# Ligand-Controlled Oxidant-Free Gold(I)/(III)-Catalyzed Synthesis of Benzocyclobutenes via [2 + 2] Annulation

**DOI:** 10.1038/s41467-026-74372-7

**Published:** 2026-06-10

**Authors:** Pengcheng Gao, Jinzhao Wang, Wenchao Chu, Tongliang Zhou, Elwira Bisz, Błażej Dziuk, Roger Lalancette, Roman Szostak, Dongju Zhang, Michal Szostak

**Affiliations:** 1 Department of Pharmacological Sciences, Icahn School of Medicine at Mount Sinai, New York, NY 10029 USA; 2 Department of Chemistry, Rutgers University, 73 Warren Street, Newark, NJ 07102 USA; 3 Key Lab of Colloid and Interface Chemistry, Ministry of Education, Institute of Theoretical Chemistry, School of Chemistry and Chemical Engineering, Shandong University, Jinan, 250100 China; 4 Department of Chemistry, Opole University, 48 Oleska Street, Opole, 45-052 Poland; 5 Department of Chemistry, University of Science and Technology, Norwida 4/6, Wroclaw, 50-373 Poland; 6 Department of Chemistry, Wroclaw University, F. Joliot-Curie 14, Wroclaw, 50-383 Poland

**Keywords:** Catalyst synthesis, Synthetic chemistry methodology

## Abstract

Benzocyclobutenes (BCBs) represent a highly strained, privileged structural motif with significant applications in drug discovery, medicinal chemistry, organic synthesis, polymers and materials science, yet efficient synthetic methods remain limited. Here, we report an oxidant-free Au(I)/Au(III)-catalyzed [2 + 2] annulation strategy for the synthesis of BCBs leveraging a unique class of rationally designed hemilabile C,N-bidentate N-heterocyclic carbene ligand (NHC). This approach employs readily available aryl halides and olefins under mild conditions, demonstrating broad functional group compatibility and the applications in late-stage functionalization. The ligand tunes the electronic and steric environment of gold center to promote selective migratory insertion while suppressing undesired β-hydride elimination. Density functional theory (DFT) calculations elucidate the reaction pathway and underscore the critical role of electron-withdrawing substituents within the C,N-carbene framework. This work establishes an efficient strategy for BCBs synthesis and highlights the potential of NHC ligands in advancing oxidant-free gold catalysis.

## Abstract (web summary)

Benzocyclobutenes (BCBs) represent a highly strained, privileged structural motif with significant applications in drug discovery, medicinal chemistry, organic synthesis, polymers and materials science, yet efficient synthetic methods remain limited. Here, the authors report an oxidant-free Au(I)/Au(III)-catalyzed [2 + 2] annulation strategy for the synthesis of BCBs leveraging a unique class of rationally designed hemilabile C,N-bidentate N-heterocyclic carbene ligand.

## Introduction

**Fig. 1:**
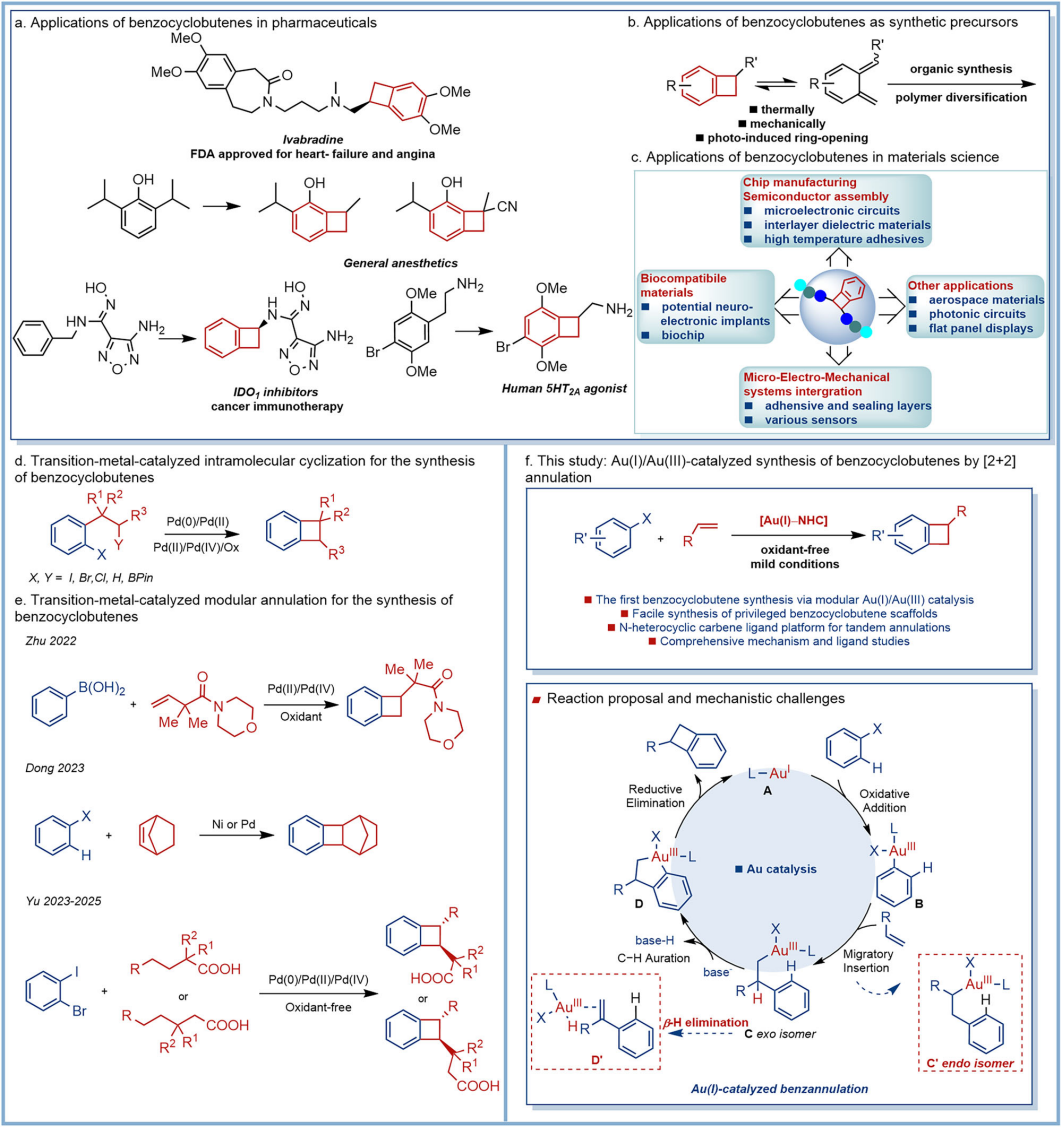
Context of this work. **a** Applications of benzocyclobutenes in pharmaceuticals. **b** Applications of benzocyclobutenes as synthetic precursors. **c** Applications of benzocyclobutenes in materials science. **d** Transition-metal-catalyzed intramolecular cyclization for the synthesis of benzocyclobutenes. **e** Transition-metal-catalyzed modular annulation for the synthesis of benzocyclobutenes. **f** This study: Au(I)/Au(III)-catalyzed synthesis of benzocyclobutenes by [2 + 2] annulation.

Benzocyclobutenes (BCBs) have been widely employed as a key class of compounds with applications throughout medicinal chemistry, drug discovery, organic synthesis, polymers and materials science^1^. Benzocyclobutanes feature a privileged structural scaffold with a thermodynamically stable benzene ring fused to a dynamic cyclobutane moiety^2–5^. As a rigid, three-dimensional pharmacophore, BCBs are found in natural products and bioactive molecules as exemplified by the marketed medicine ivabradine for the treatment of heart failure and angina (Fig. 1a)^2^. Importantly, as the surrogate of benzylic functional group, replacement with BCB results in improved potency and selectivity, higher metabolic stability and improved pharmacokinetic profiles in drug discovery, cancer immunotherapy and neural signaling modulation^3–5^.

Separately, the unique properties of BCBs to undergo electrocyclic ring opening induced by heat, light or mechanical force renders them highly valuable as precursors for organic synthesis. The resulting reactive intermediates *o*-quinodimethanes have been widely used in pericyclic reactions in natural product synthesis and preparation of cross-linked polymers (Fig. 1b)^6,7^. Most importantly, BCB-based polymers show unique properties such as low dielectric performance, minimal thermal expansion and reduced moisture absorption, which altogether make BCB-based polymers supreme materials in various fields such as semiconductor industry, biomedical implants, micro-electro-mechanical systems integration and advanced aerospace material engineering (Fig. 1c)^7^.

Given the significance of BCBs, methods for the synthesis of BCBs have been developed, including cycloaddition, rearrangements, photo-induced radical processes, and transition-metal catalysis^8,9^. Among these approaches, Pd catalysis is a promising strategy for constructing BCB scaffolds (Fig. 1d)^10–15^. Recently, Zhu group reported an inventive intermolecular synthetic coupling of aryl boronic acids and alkenes via a Pd(II)/Pd(IV) cycle (Fig. 1e)^16^. The reaction employs a specialized substitution for alkene substrates by installing amide directing group and disubstitution at the α-carbon of amide carbonyl. Later, the Yang group reported an asymmetric [2 + 2] annulation for synthesis of chiral BCBs using similar strategy^17^. The Dong group elegantly explored the Catellani-type annulation for constructing BCB derivatives for the synthesis of norbornene-fused BCBs (Fig. 1e)^18^. Most remarkably, Yu group introduced a Pd catalyzed regiocontrolled [2 + 2] benzannulation of aryl halides and aliphatic carboxylic acids via double C(sp^3^)–H activation of 2,2-disubstituted aliphatic acids (Fig. 1e)^19,20^. This approach requires 1,2-disubstituted aryl halides and specific aliphatic acids.

Meanwhile, as an emerging alternative to Pd(0/II) and Pd(II/IV) catalysis, Au(I)/Au(III) redox cycle has garnered tremendous attention in synthetic community^21–23^. In particular, the long-debated oxidative addition of Au(I) has been achieved by rational design of bidentate P,N and C,N ligands. Crucially, beyond the Au(I)/Au(III) catalytic cycle, gold catalysis has been long recognized to exhibit unique carbophilic π-activation properties toward unsaturated carbon-carbon bonds, clearly distinguishing this catalysis manifold from palladium and further expanding its utility in the construction of complex functional molecules^24^.

Inspired by our previous studies in N-heterocyclic carbenes (NHCs) for Au(I)/Au(III) catalysis^22,25,26^, most specifically ImPy (ImPy = imidazo[1,5-a]pyridin-3-ylidene) ligands, herein, we report an oxidant-free gold(I)/(III)-catalyzed [2 + 2] annulation for the synthesis of BCBs leveraging a unique class of rationally designed C,N-bidentate NHCs. This catalysis approach in constructing valuable BCBs advances the palladium-catalyzed methods to a gold-mediated pathway (Fig. 1f). Crucially, this approach employs readily available aryl halides and olefins under mild conditions, demonstrating broad functional group compatibility. The method is demonstrated in late-stage functionalization of complex bioactive molecules, underscoring its potential for pharmaceutical applications. Mechanistic investigations provide key insights into the reaction pathway and the key role of ligand architecture in the C,N bidentate carbene framework. Overall, this study demonstrates the synthetic utility of oxidant-free Au(I)/Au(III) catalysis and highlights the role of NHCs in unlocking unique reactivity paradigms in gold catalysis, paving the way for broad applications of benzocyclobutanes in diverse interdisciplinary chemical fields. The study advances: (1) the use of readily available aryl halides and olefins as starting materials, enabling efficient annulation protocol via Au(I)/Au(III) catalysis; (2) the synthesis of BCBs with broad scope, high efficiency, and excellent generality, expanding their application; (3) the development of a modular carbene ligand platform to spearhead the application of gold(I)/(III) catalysis.

## Results

### Design

Based on the mechanism of gold-catalyzed BCB synthesis, although the gold catalysis involved migratory insertion and C-H auration have been reported recently^27–30^, our proposed mechanism pathway toward BCBs synthesis faced formidable challenges, such as the selectivity of migratory insertion and competing β-H elimination (Fig. 1f). We proposed to tackle these significant issues by exploiting the unique steric and electronic properties of C,N-bidentate carbenes. Firstly, we introduced a C,N ligand framework ImQun (ImQun = imidazo[1,5-*a*]quinoline) which incorporates the extended ring compared with our previous ImPy C,N ligands. We proposed that ImQun scaffold could lead to shorter Au-NMe_2_ distance which was validated by our X-ray study (Supplementary Fig. S1), thus providing better stabilizing effect toward gold center during the catalysis. The shorter Au–N(Me)₂ distance increases steric congestion around the gold center, which may favor selective migratory insertion while restricting the conformational flexibility required for the undesired β-hydride elimination. Importantly, we hypothesized that the introduction of electron-withdrawing groups on the ImQun C,N ligands would increase the electrophilicity of the Au(III) intermediates, thereby favoring aromatic C–H auration over the undesired β-hydride elimination. In addition, such electronic modulation may enhance the electronic dissymmetry of the C,N coordination mode, which could further promote selective migratory insertion^31^.

### Ligand development and reaction optimization

With our hypothesis of reaction pathway, we set out to explore various gold catalysts for BCBs synthesis. To identify an optimal catalyst, we conducted a systematic evaluation of bidentate C,N carbene ligands which stabilize both Au(I) and Au(III) intermediates in the proposed Au(I)/Au(III) cycle. The screening results are summarized in Table 1 (entry 1-8). The model reaction was conducted under optimized conditions following screening of solvents, silver salts, bases and catalyst loading for Au(I)/Au(III) catalysis (SI, Tables S1–S5). Aryl halide **2** and olefin **1a** were reacted in DCE at 80 °C in the presence of AgNTf₂ as a halide scavenger and K₃PO₄ as the base. To our delight, ImPyNMe_2_DippAuCl (**L1AuCl**) afforded the desired BCB product in 28% yield. Interestingly, the bulkier ImPyNpipDippAuCl (**L2AuCl**) failed to promote the reaction. Next, switching the ImPy to ImPhen (imidazo[1,5-*a*][1,10]phenanthroline) framework (**L3AuCl**) resulted in no detectable product formation. Then we turned our attention to a ligand framework featuring imidazo[1,5-a]quinoline^25^. Interestingly, ImQunNMe2DippAuCl (**L4AuCl**) showed a higher yield (33%) than its less sterically demanding congener ImQunNMe2MesAuCl (**L5AuCl**) (16%). Considering the challenges of migratory insertion and C–H activation steps, we introduced an electron-withdrawing C6-nitro group at the carbene framework (**L6AuCl**) and at the C4-wingtip aryl (**L7AuCl**). Pleasingly, although **L7AuCl** gave a moderate yield, **L6AuCl** showed excellent catalytic efficiency, providing the desired product in 79%. Furthermore, we tested the most widely used MeDalphosAuCl gold catalyst in Au(I)/Au(III) cycle^21,24,30^ and no desired product **3a** was detected. We observed that **L4AuCl** and **L6AuCl** afforded negligible amounts of byproduct **3a′**, whereas **L1AuCl,** **L5AuCl,** **L7AuCl**, and **L8AuCl** produced byproduct **3a**’ in 6–12% yield, arising from undesired β-hydride elimination. These results indicate that both the electronic and steric properties of the ligands influence the reaction pathway. Overall, all results indicate the unique properties of C,N bidentate carbenes playing a crucial role in facilitating the gold-catalyzed [2 + 2] annulation and offering opportunities in the powerful gold(I)/(III) redox cycle.

**Table 1:** Ligand Screening and Reactivity Studies^*a*^


Entry	[Au–NHC]	3a yield (%)		3a’ yield (%)
1	L1AuCl		28	9
2	L2AuCl		0	0
3	L3AuCl		0	0
4	L4AuCl		33	trace
5	L5AuCl		16	12
6	L6AuCl		79	trace
7	L7AuCl		30	6
8	L8AuCl		0	11

^*a*^Conditions: **1a** (2.0 equiv), **2** (1.0 equiv), [NHC–Au–Cl] (10 mol%), AgNTf_2_ (1.2 equiv), K_3_PO_4_ (0.5 equiv), DCE (0.125 M), 80 °C, 16 h.

### Substrate scope

**Fig. 2:**
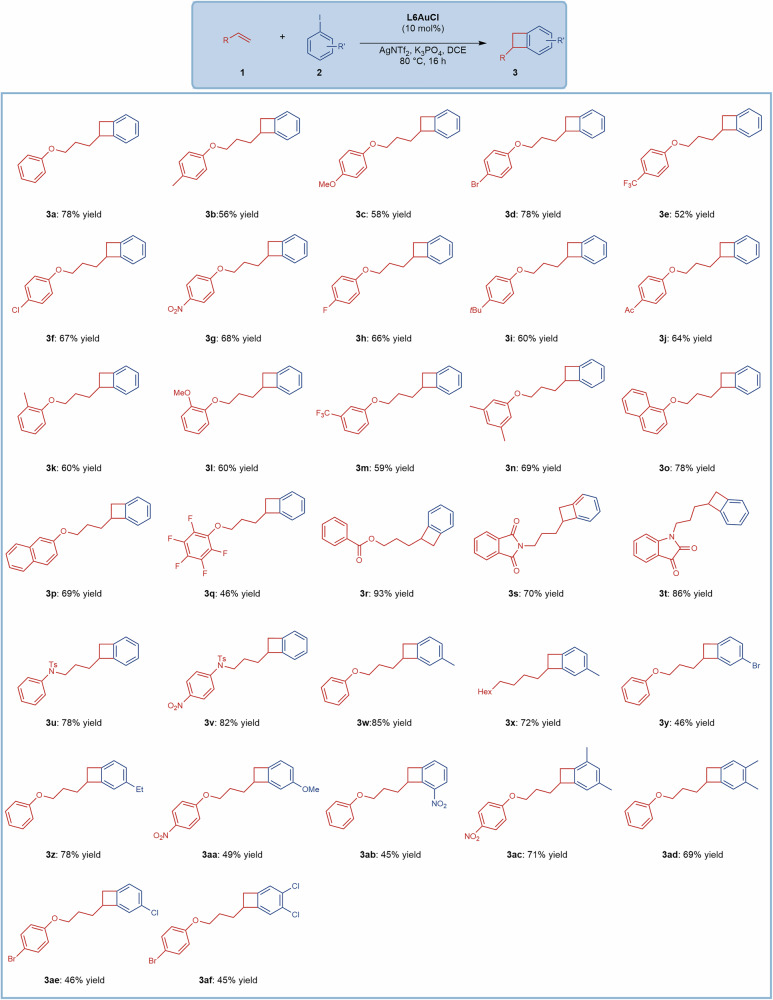
Substrate scope. Conditions: **1** (2.0 equiv), **2** (1.0 equiv), L6AuCl (10 mol%), AgNTf_2_ (1.2 equiv), K_3_PO_4_ (0.5 equiv), DCE (0.125 M), 80 °C, 16 h. Isolated yields are reported.

With the optimal gold–NHC catalyst in hand (**L6AuCl)**, we next investigated the substrate scope of olefins and aryl halides in this gold-catalyzed annulation to form BCBs. As demonstrated in Fig. 2, this protocol is characterized by broad applicability for the synthesis of valuable BCB products with high efficiency. Olefins bearing electron-neutral (-Me, -*t*Bu), electron-donating (-OMe), and electron-withdrawing substituents (-CF_3_, -NO_2_, -Ac) are well-compatible with this protocol. Importantly, different halide substituents (-F, -Cl, -Br) are well tolerated, providing functionalized BCB products (**3a**-**3n,3q**) with handles for the conventional Pd- and Ni-cross-coupling. Further, naphthalene derived olefins are tolerated and afford the desired BCBs **3o** (78%) and **3p** (69%). Pleasingly, olefins bearing ester (**3r**, 93%), amide (**3s**-**3t**, 70%–86%), and sulfonamide (**3u**-**3v**, 78-82%) functional groups provide valuable BCBs in good yields, underscoring the functional group tolerance of this gold-catalyzed annulative coupling. Moreover, different arene substitution patterns have been demonstrated in this gold-catalyzed BCBs synthesis. Notably, the *meta*-substituted iodobenzenes bearing 3-Me (**3x**, 72%), 3-Br (**3 y**, 46%), 3-Et (**3z**, 78%), 3-OMe (**3aa**, 49%), and 3-Cl (**3ae**, 46%) and *ortho*-substituted iodobenzene (**3ab**, 45%) smoothly and regioselectively provided the desired BCBs. Importantly, any other potential regioisomers via *endo* migratory insertion were not detected (Fig. 1f). We hypothesize that this unique regioselectivity is controlled by the umbrella-shaped steric hindrance of N-wingtip of NHC ligand (*vide infra*). Finally, disubstituted iodobenzenes also yield the desired BCB products with good efficiency (**3ac**, 71%; **3ad**, 69%; **3af**, 45%), demonstrating the robustness of this transformation. These results also indicate an impactful role Au(I)–NHC complexes in the discovery of [2 + 2] annulations for organic synthesis.

### Late-Stage Functionalization

**Fig. 3:**
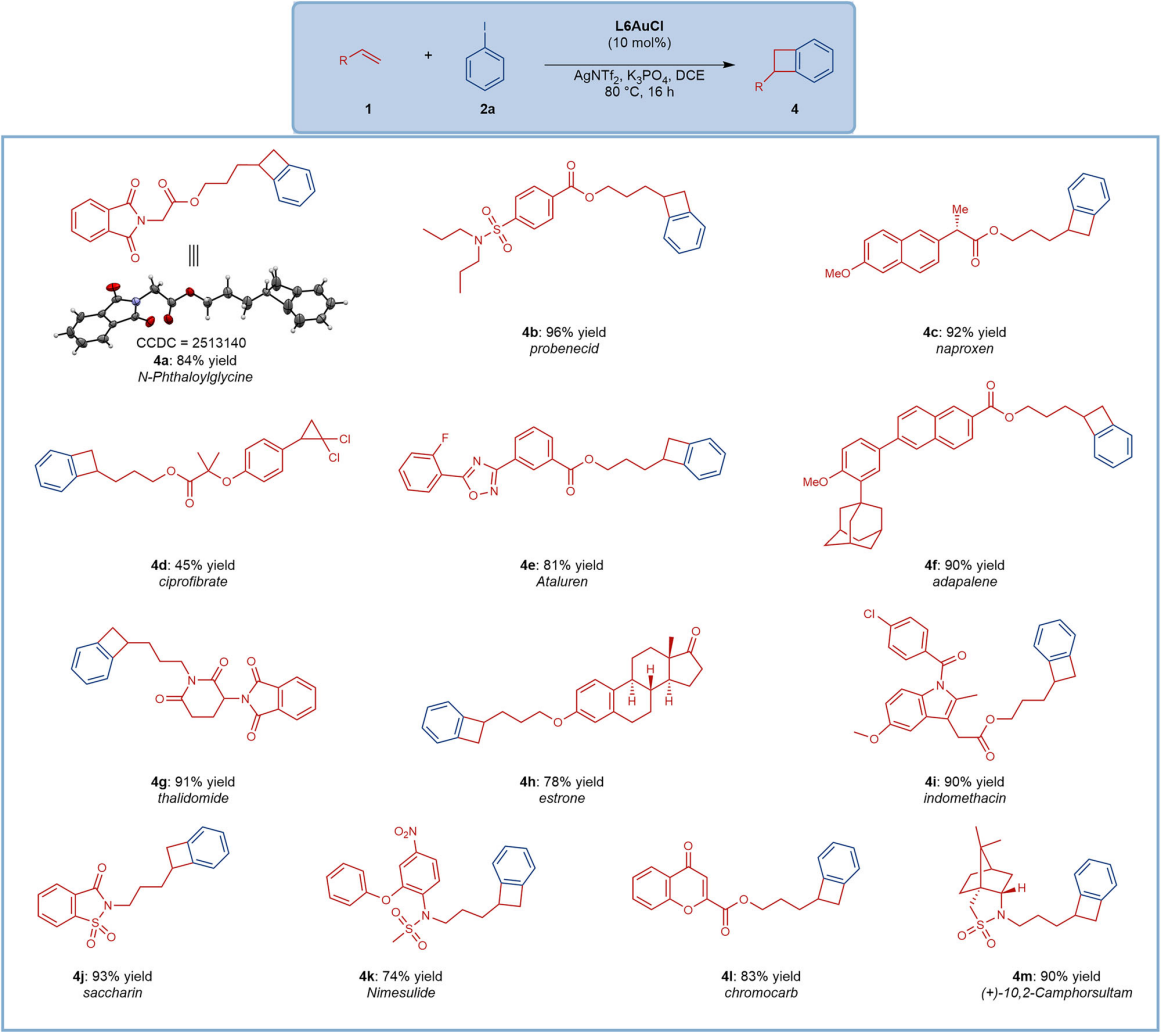
Late-stage functionalization. Conditions: **1** (2.0 equiv), **2a** (1.0 equiv), L6AuCl (10 mol%), AgNTf_2_ (1.2 equiv), K_3_PO_4_ (0.5 equiv), DCE (0.125 M), 80 °C, 16 h. Isolated yields are reported.

To further highlight the synthetic utility and functional group tolerance of this gold-catalyzed annulation, we explored late-stage functionalization of structurally complex pharmaceutical molecules. As depicted in Fig. 3, a broad array of bioactive compounds and drug-derived molecules could be successfully annulated, demonstrating the robustness and versatility of this gold-catalyzed BCB synthesis. Specifically, olefins of *Pht-Gly-OH*, *Probenecid*, *Naproxen*, *Ciprofibrate*, *Ataluren*, *Adapalene*, *Thalidomide*, *Estrone*, *Indomethacin*, *Saccharin*, *Nimesulide*, *Chromocarb* and (+)−10,2-*Camphorsultam* underwent efficient [2 + 2] annulation to afford the desired products with exceptional functional group tolerance **4a** (84%), **4b** (96%), **4c** (92%), **4 d** (45%), **4e** (81%), **4 f** (90%), **4 g** (91%), **4 h** (78%), **4i** (90%), **4j** (93%), **4k** (74%), **4 l** (83%) and **4 m** (90%). It is worth noting that all late-stage functionalizations were conducted on a 0.2 mmol scale. Furthermore, a large-scale reaction (1.0 mmol) was also demonstrated for product **3a**, affording a 68% isolated yield. These results further underscore the efficiency of Au(I)/(III)–NHC catalysis, showcasing the mild conditions and functional group tolerance, and indicate a potential for broad application of this protocol for modification of medicinally significant molecules.

### Mechanism studies

**Fig. 4:**
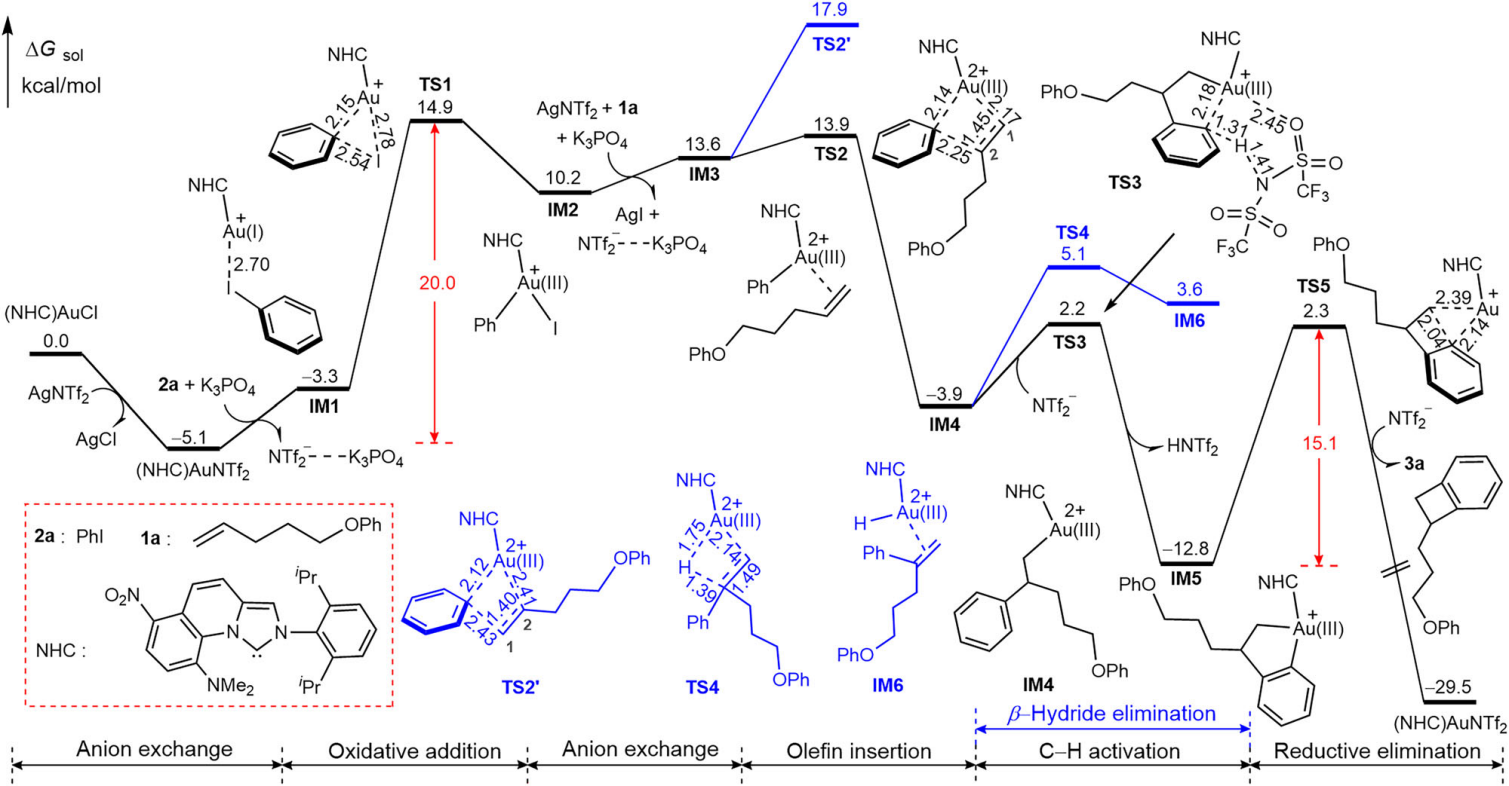
**Computational studies**. Calculated Gibbs free energy profile for the Au(I)-catalyzed annulation of iodobenzene (**2a**) with olefin (**1a**) Using ligand **L6**. Bond distances are given in Å.

To elucidate the mechanism of Au(I)/Au(III) catalysis in the annulation of aryl halides and olefins, we conducted density functional theory (DFT) calculations on a representative example using olefin **1a** and iodobenzene **2** as reactants and **L6** as the ligand. The resulting Gibbs free energy profile is depicted in Fig. 4. The Cartesian coordinates of all optimized structures are provided in the Source Data file.

Initially, the precatalyst (NHC)AuCl undergoes counter-anion exchange with AgNTf_2_, releasing 5.1 kcal/mol of energy and generating the catalytically active (NHC)AuNTf_2_ species. The NTf_2_^−^ anion in (NHC)AuNTf_2_ is then displaced by iodobenzene **2**, assisted by K_3_PO_4_ which stabilizes the departing NTf_2_^−^ anion. This substitution affords the iodine-coordinated Au(I) σ-complex **IM1**. From **IM1,** **2** undergoes oxidative addition to the Au center via transition state **TS1**, leading to the formation of the Au(III)-aryl intermediate **IM2**. The overall barrier for the formation of **IM2** is calculated to be 20.0 kcal/mol.

Subsequently, **IM2** undergoes halide abstraction by AgNTf_2_ with concomitant release of AgI, followed by K_3_PO_4_-facilitated dissociation of NTf_2_^−^⊡ This sequence enables coordination of **1a** to the metal center, yielding the olefin-coordinated intermediate **IM3**. The next step involves insertion of the olefin into the Au−C bond to form the Au(III)-alkyl species. Two possible insertion modes (1,2- and 2,1-insertions) of the asymmetric olefin (**1a**) into the Au−C bond were considered. The calculations indicate that the 1,2-insertion pathway via **TS2** is energetically more favorable than the 2,1-insertion pathway via **TS2’** by 4.0 kcal/mol.

**Fig. 5:**
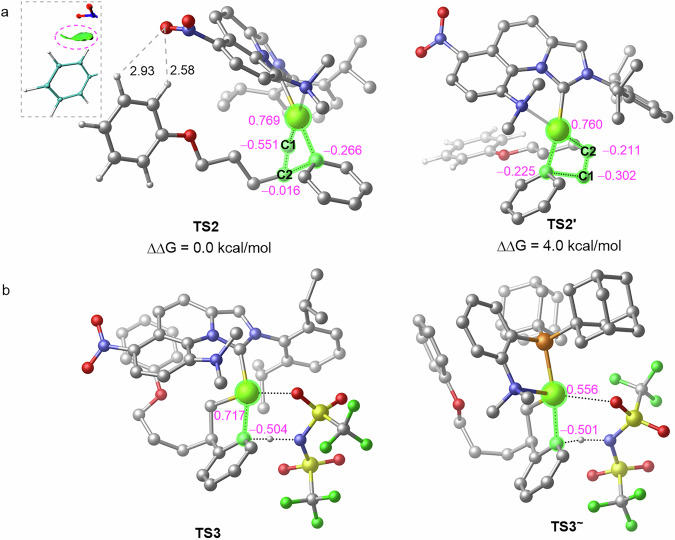
**Computational analysis of optimized transition-state geometries**. **a** The regioselectivity-determining transition states **TS2** and **TS2’** with ligand **L6**, with inserts showing the isodensity surfaces derived from IGMH analysis. **b** The C−H activation transition states **TS3** (with **L6**) and **TS3**^**~**^ (with **L8**), with selected NPA charges (in *e*) indicated. Hydrogen atoms irrelevant to noncovalent interactions or C–H activation are omitted for clarity.

To gain insight into the origin of this regioselectivity, we performed natural population analysis (NPA) and independent gradient model based on Hirshfeld partitioning (IGMH) analysis on transition states **TS2** and **TS2’** with the results presented in Fig. 5a. The calculated NPA charges suggest that **TS2** is favored over **TS2’** due to a stronger electrostatic interaction between the Au center and C1 in **TS2**, which promotes the 1,2-insertion pathway. This enhanced interaction arises from reduced steric repulsion and better stability of the electron distribution of C1 and C2. On the other hand, IGMH analysis reveals a pronounced C−H···O hydrogen bond interaction between the -NO_2_ group of the ligand and the phenyl substituent of **1a** in **TS2**. In contrast, this interaction is absent in **TS2’** due to a larger spatial separation between these two groups. The combined effects of the stronger electrostatic interaction and the additional hydrogen bonding in **TS2** over **TS2’** cooperatively favor the 1,2-insertion pathway.

The Au(III)-alkyl species **IM4**, formed via olefin insertion, undergoes C−H activation facilitated by NTf_2_^−^ through transition state **TS3**, with an activation barrier of 6.1 kcal/mol, affording the five-membered metallacycle **IM5**. Finally, C−C reductive elimination occurs from **IM5** via **TS5** with a barrier of 15.1 kcal/mol, resulting in the product **3a** and regenerating the catalytically active (NHC)AuNTf_2_ species. Additionally, we evaluated a possible *β*-hydride elimination process from **IM4**. However, the calculated energy barrier for this process is 2.9 kcal/mol higher than that of the C−H activation pathway. Therefore, the *β*-hydride elimination mechanism can be ruled out from further consideration. To provide experimental support for the proposed mechanism, HRMS experiments were performed to detect potential intermediates. Signals corresponding to the oxidative addition intermediate and intermediate IM5 were identified, supporting the proposed reaction pathway derived from our calculations.

Furthermore, we performed DFT calculations on the catalytic system employing the MeDalphos ligand **L8** to rationalize the superior reactivity of ligand **L6**, which features an electron-withdrawing -NO_2_-substituted C, N-bidentate carbene. The calculated results are presented in Supplementary Fig. S3. Notably, the energy barrier for the C−H activation step via **TS3**^**~**^ is 3.5 kcal/mol higher than that for the competing *β*-hydride elimination pathway via **TS4**^**~**^. Consequently, the reaction preferentially proceeds through *β*-hydride elimination, leading to the formation of the undesired olefin product **P’**. These theoretical findings are consistent with experimental observations, where no product **3a** is obtained when using the MeDalphos ligand **L8**.

The enhanced C−H activation ability observed with ligand **L6**, relative to **L8**, can be rationalized based on the calculated NPA charges for **TS3** and **TS3**^**~**^ (Fig. 5b). In **TS3** (with **L6**), the Au center bears a significantly higher positive charge than in **TS3**^**~**^ (with **L8**), with values of 0.717 and 0.556 *e*, respectively. This increased electrophilicity strengthens the electrostatic interaction between the Au center and the carbon atom of the aryl substrate, thereby favoring the C–H activation. The elevated positive charge on the Au center in **TS3** can be attributed to the strong electron-withdrawing inductive effect of the -NO_2_ substituent on the ligand. Furthermore, NPA charges on the Au centers of **L1AuCl**–**L8AuCl** were calculated and compared based on Natural Bond Orbital (NBO) analysis (Supplementary Fig. S4). **L8AuCl** (0.152 *e*) displays the lowest electrophilicity, whereas **L6AuCl** (0.241 *e*) exhibits the highest. The slightly increased electrophilicity of **L6AuCl** (0.241 *e*) relative to **L4AuCl** (0.239 *e*) is attributed to the electron-withdrawing –NO₂ substituent on the ImQun framework. Overall, we propose that the electrophilicity of the gold center is a key factor in the C–H auration and reductive elimination steps, which are crucial for the formation of BCB products.

### Crystallographic characterization

**Fig. 6:**
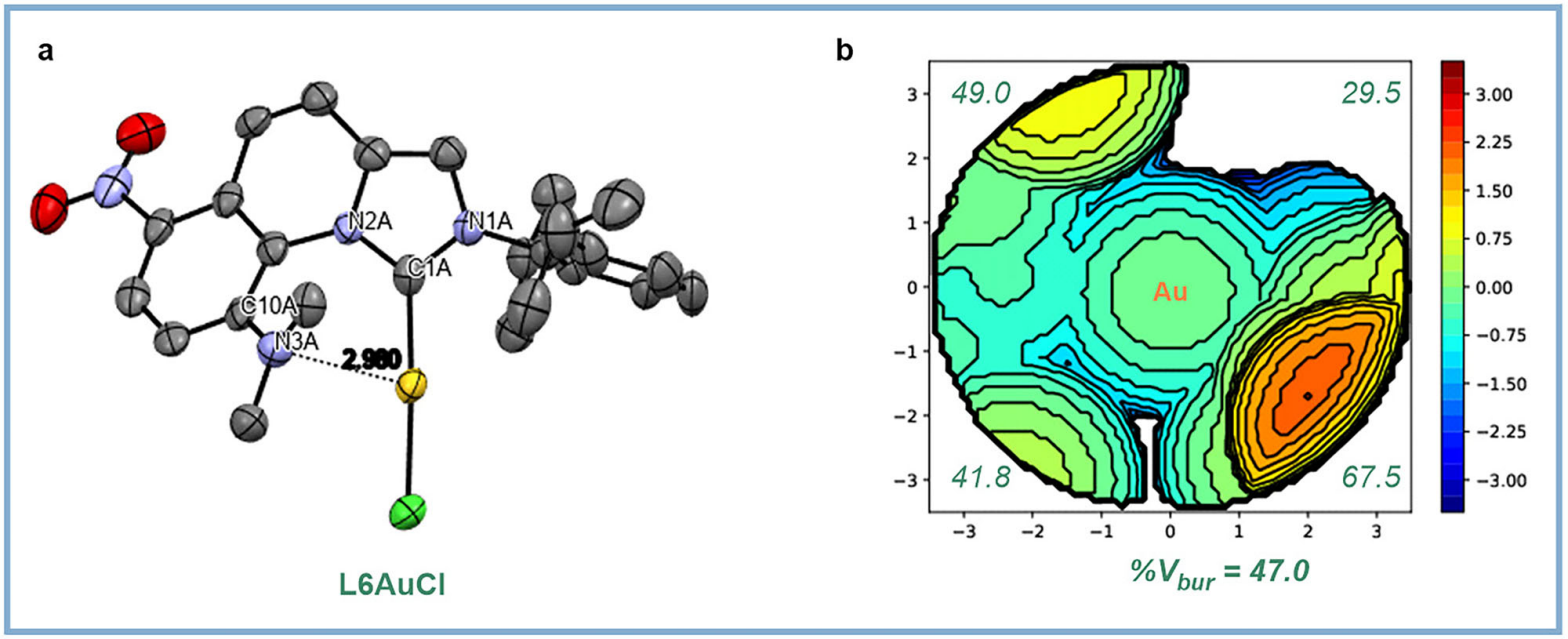
Crystallographic studies. **a** X-ray crystal structure of L6AuCl. Hydrogen atoms have been omitted for clarity. Selected bond lengths [Å] and angles [°]. CCDC 2513141, L6AuCl: Au1A–C1A, 1.978(8); Au1A–Cl1A, 2.276(2); C1A–N1A, 1.37(1); C1A–N2A, 1.37(1); Au1A-N3A, 2.980; C10A-N3A, 1.38(9); Cl1A–Au1A–C1A, 176.1(2); N1A–C1A–N2A, 103.8(6). **b** Topographical steric map of L6AuCl showing %V_*bur*_ per quadrant.

We have also investigated the structural features of the optimal gold catalyst **L6AuCl** by crystallographic analysis (Fig. 6a). It should be noted that the complex is air- and moisture-stable. As shown in Fig. 6a, **L6AuCl** gold complex, which features the ImQun–NHC scaffold, is characterized by a short Au···NMe_2_ distance (<3.0 Å), indicating a non-bonding interaction between Au(I) and a hemilabile NMe_2_ group that enables catalyst stabilization. **L6AuCl** exhibits a shorter Au···NMe₂ distance than its ImPy congener **L1AuCl** (2.980 Å vs. 3.141 Å)^22^, reflecting the enhanced electron-donating ability of the NMe₂ group in **L6AuCl**. The aryl C–N bond (C10A–N3A) in **L6AuCl** is slightly shorter than the corresponding bond in **L1AuC**l (1.380 Å vs. 1.390 Å), which is characteristic of the electron-withdrawing NO₂ substituent at the C6 position of the ImQun scaffold, indicating enhanced conjugation between the NMe₂ group and the ImQun framework. The complex displays a slight deviation from the linear C_(carbene)_–Au(I)–Cl geometry (176.1°).

We applied the X-ray based-steric map analysis developed by Nolan, Cavallo and co-workers to determine the %buried volume (%V_*bur*_)^32,33^, reflecting the steric environment of this ImQun–NHC–Au complex (Fig. 6b). The steric map of L6AuCl indicates a significant buried volume around the gold center (%V_*bur*_ of 47.0%) with the characteristic unsymmetrical steric pattern with the most bulk quadrant near NMe_2_ (67.5%) and one unencumbered quadrant near the ImQun core (29.5%). This steric distribution around the carbene centre together with the electronic properties of the scaffold and the hemilabile stabilization by the donor amino group provide key differentiation of this ligand platform from other known carbene and phosphine ligands. These features are crucial for promoting the [2 + 2] annulation mechanism.

## Discussion

In summary, we have developed an Au(I)/Au(III)-catalyzed [2 + 2] benzannulation that enables efficient and highly regioselective synthesis of challenging BCB scaffolds from readily available aryl halides and olefins. The success of this transformation can be attributed to redox Au(I)/Au(III) catalysis enabled by the development of an Au(I)–NHC ligand platform. By exploiting C, N-bidentate carbene ligands, we effectively addressed the key challenges in BCB synthesis, such as the selectivity of migratory insertion and undesired β-elimination. The broad substrate scope, excellent functional group tolerance, and late-stage modification of bioactive molecules highlight the potential impact of this methodology in furthering the utility of BCBs across chemical research. Compared with the previous synthetic methods, this protocol advances Pd catalysis in the synthesis of BCB to the versatile Au(I)/(III) cycle, showcasing the avoidance of directing groups, lack of external oxidants, and mild conditions, while marking the application of gold catalysis for the synthesis of valuable BCB scaffolds. The mechanistic DFT studies provide valuable insight into the reaction pathway, demonstrating the key role of C, N-bidentate carbene ligands and paving the way for advancements in redox gold catalysis. Considering the significant interest in gold-mediated transformations, we anticipate innovations that will unlock catalytic reactivity and expand the synthetic toolbox for the construction of highly complex and challenging molecules.

## Methods

General procedure for the synthesis of benzocyclobutenes: An oven-dried microwave vial equipped with a stir bar was charged with iodoarene (0.2 mmol, 1.0 equiv), alkene (0.40 mmol, 2.0 equiv), L6AuCl (0.02 mmol, 10 mol%), AgNTf_2_ (0.24 mmol, 1.2 equiv), K_3_PO_4_ (0.1 mmol, 0.5 equiv), DCE (0.125 M) at room temperature. Then, the reaction mixture was placed in a preheated oil bath at 80 °C, and stirred for 16 h at 80 °C. After the indicated time, the reaction mixture was cooled down to room temperature, diluted with CH_2_Cl_2_ (10 mL), filtered, and concentrated. The residue was analyzed by ^1^H NMR (CDCl_3_, 500 MHz) and GC-MS using an internal standard. Purification by chromatography on silica gel (EtOAc/hexanes or DCM/MeOH) or reverse phase chromatography to afford the title product.

## Supplementary information

Supplementary Information

Transparent Peer Review file

## Source data

Source Data

## Data Availability

Experimental procedures and characterization data are available within this article are provided in the Supplementary Information/Source Data file. Crystallographic data for the structures reported in this article have been deposited at the Cambridge Crystallographic Data Centre, under deposition numbers CCDC 2513140 (4a) and CCDC 2513141 (L6AuCl). Source data for the Cartesian coordinates of all optimized structures are provided with this paper. All data are available from the corresponding author upon request. Source data are provided with this paper.
