# Supplementary material for: Ligand-Controlled Oxidant-Free Gold(I)/(III)-Catalyzed Synthesis of Benzocyclobutenes via [2 + 2] Annulation
Source: Nat Commun. 2026 Jun 10;17:7368. doi: 10.1038/s41467-026-74372-7 (PMC13402704; doi:10.1038/s41467-026-74372-7)
Supplement: Supplementary file 1 — Supplementary Information [file 41467_2026_74372_MOESM1_ESM.pdf]

Supplementary Information

# Ligand-Controlled Oxidant-Free Gold(I)/(III)-Catalyzed Synthesis of Benzocyclobutenes via [2+2] Annulation

Pengcheng Gao *et al.*

*Corresponding author. Email: pg459@scarletmail.rutgers.edu; zhangdj@sdu.edu.cn; michal.szostak@rutgers.edu

**Table of Contents** S1

Known Compounds/General Methods S2
Experimental Procedures and Characterization Data S4
Synthesis of Ligands and Gold Complexes S4
Additional Optimization Studies S13
General Procedure and Product Characterization S17
HRMS Studies for Possible Intermediates S42
1 mmol Scale Synthesis S44
Crystallographic Studies S45
NMR Spectra S49
Computational Studies S118
References S124

## Known Compounds/General Methods

All starting materials reported in the manuscript have been previously described in literature and prepared by the method reported previously unless state otherwise. All experiments were performed using standard Schlenk techniques under nitrogen or argon unless stated otherwise. All solvents were purchased at the highest commercial grade and used as received or after purification by passing through activated alumina columns or distillation from sodium/benzophenone under nitrogen. All solvents were deoxygenated prior to use. All other chemicals were purchased at the highest commercial grade and used as received. Reaction glassware was oven-dried at 140 °C for at least 24 h or flame-dried prior to use, allowed to cool under vacuum and purged with argon (three cycles). All products were identified using $^{1}$H NMR analysis and comparison with authentic samples. GC and/or GC/MS analysis was used for volatile products. All yields refer to yields determined by $^{1}$H NMR and/or GC or GC/MS using an internal standard (optimization) and isolated yields (preparative runs) unless stated otherwise. $^{1}$H NMR and $^{13}$C NMR spectra were recorded in $CDCl_3$ on Bruker spectrometers at 500 ($^{1}$H NMR) and 125 MHz ($^{13}$C NMR). All shifts are reported in parts per million (ppm) relative to residual $CHCl_3$ peak (7.26 and 77.2 ppm, $^{1}$H NMR and $^{13}$C NMR, respectively). All coupling constants (J) are reported in hertz (Hz). Abbreviations are: s, singlet; d, doublet; t, triplet; q, quartet; brs, broad singlet. GC-MS chromatography was performed using Agilent HP6890 GC System and Agilent 5973A inert XL EI/CI MSD using helium as the carrier gas at a flow rate of 1 mL/min and an initial oven temperature of 50 °C. The injector temperature was 250 °C. The detector temperature was 250 °C. For runs with the initial oven temperature of 50 °C, temperature was increased with a 10 °C/min ramp after 50 °C hold for 3 min to a final temperature of 220 °C, then hold at 220 °C for 15 min (splitless mode of injection, total run time of 22.0 min). High-resolution mass spectra (HRMS) were measured on a 7T Bruker Daltonics FT-MS instrument. All flash chromatography was performed using silica gel, 60 Å, 300 mesh. TLC analysis was carried out on glass plates coated with silica gel 60 F254, 0.2 mm thickness. The plates were visualized using a 254 nm ultraviolet lamp or aqueous potassium permanganate solutions. $^{1}$H NMR and $^{13}$C NMR data are given for all compounds

in the SI for characterization purposes. $^{1}$H NMR, $^{13}$C NMR, and HRMS data are given for all new compounds. All products have been previously reported, unless stated otherwise.

# Experimental Procedures and Characterization Data

## Synthesis of Ligands and Gold Complexes.

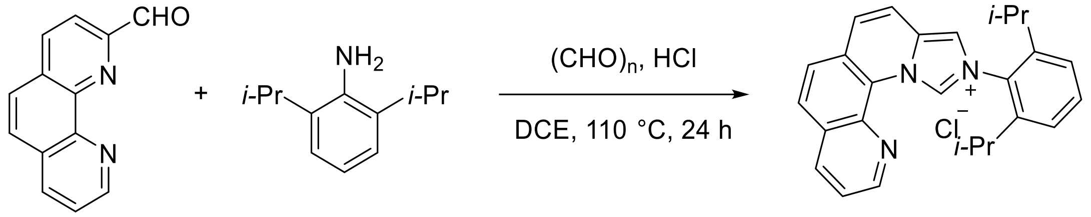


**10-(2,6-Diisopropylphenyl)imidazo[1,5-*a*][1,10]phenanthrolin-10-ium chloride.** An oven-dried 100 mL pressure vessel equipped with a stir bar was charged with 1,10-phenanthroline-2-carbaldehyde (2.08 g, 10 mmol, 1.0 equiv),[1] 2,6-diisopropylaniline (1.77 g, 10 mmol, 1.0 equiv), paraformaldehyde (450 mg, 15 mmol, 1.5 equiv) and DCE (25 mL). HCl (4.0 M in dioxane, 5.0 mL, 2.0 equiv) was added dropwise into the reaction at room temperature. The mixture was stirred at 110 °C for 24 hours. After the indicated time, the reaction mixture was cooled down to room temperature and concentrated. The residue was purified by chromatography on silica gel ($CH_2Cl_2$/MeOH = 20/1) to afford the title product (3.45 g, 83%). $^1$H NMR (500 MHz, $CDCl_3$) δ 11.77 (s, 1H), 9.31 (d, $J$ = 1.8 Hz, 1H), 9.16 (dd, $J$ = 4.3, 1.7 Hz, 1H), 9.05 (d, $J$ = 9.4 Hz, 1H), 8.59 (dd, $J$ = 8.2, 1.8 Hz, 1H), 8.23 (d, $J$ = 8.6 Hz, 1H), 8.12 (d, $J$ = 8.6 Hz, 1H), 7.93 (d, $J$ = 9.5 Hz, 1H), 7.79 (dd, $J$ = 8.2, 4.4 Hz, 1H), 7.64 (t, $J$ = 7.9 Hz, 1H), 7.41 (d, $J$ = 8.0 Hz, 2H), 2.26 (p, $J$ = 6.8 Hz, 2H), 1.24 (d, $J$ = 6.8 Hz, 6H), 1.17 (d, $J$ = 6.8 Hz, 6H). $^{13}$C NMR (126 MHz, CDCl3) δ 149.82, 144.31, 138.72, 136.68, 131.12, 130.24, 130.08, 129.35, 128.47, 128.09, 126.76, 126.50, 125.95, 123.75, 123.36, 122.02, 120.03, 118.22, 27.82, 23.74, 23.19. HRMS calcd for $C_{26}H_{26}N_3^+$ (M – $Cl^-$) 380.2121, found 380.2139.

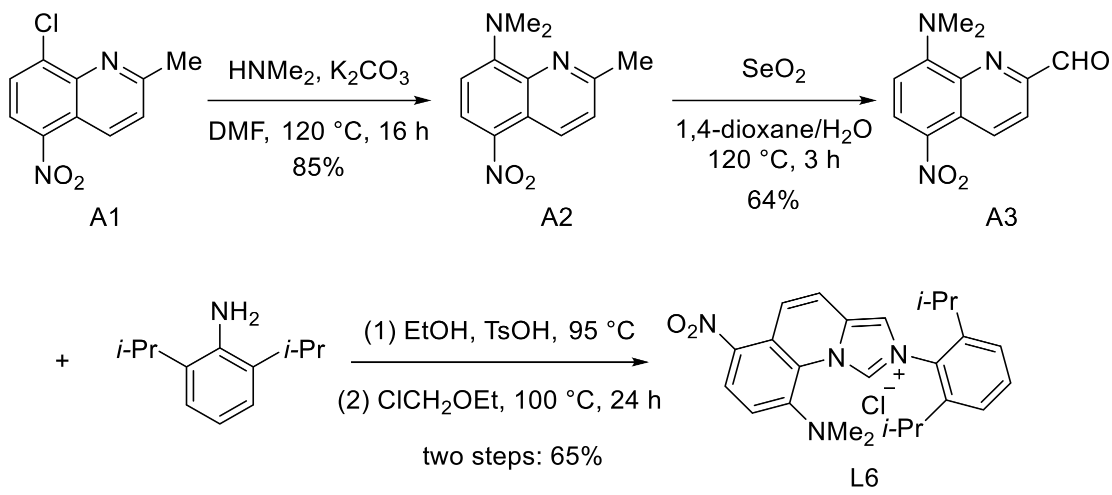


***N,N*,2-Trimethyl-5-nitroquinolin-8-amine (A2).** An oven-dried 200 mL pressure vessel equipped with a stir bar was charged with 8-chloro-2-methyl-5-nitroquinoline (8.91 g, 40 mmol, 1.0 equiv),[2] $K_2CO_3$ (11.04 g, 80 mmol, 2.0 equiv), dimethylamine solution (200 mmol, 5.0 equiv, 40 wt% in $H_2O$) and DMF (50 mL). The mixture was heated at 120 °C until the starting material was fully consumed (24 hours). Then, the mixture was cooled down to room temperature and diluted with DCM, washed with $H_2O$ and brine. The organic layers were collected, dried over $Na_2SO_4$, filtered, and concentrated. The resulting brown solid was used directly in the next step (7.86 g, 85%).$^1$H NMR (500 MHz, $CDCl_3$) δ 9.21 (d, *J* = 8.9 Hz, 1H), 8.40 (d, *J* = 9.0 Hz, 1H), 7.46 (d, *J* = 8.9 Hz, 1H), 6.82 (d, *J* = 9.1 Hz, 1H), 3.38 (s, 6H), 2.74 (s, 3H). $^{13}$C NMR (126 MHz, $CDCl_3$) δ 155.62, 155.25, 139.53, 134.94, 132.89, 127.32, 124.52, 122.36, 109.88, 44.40, 25.10. HRMS calcd for $C_{12}H_{14}N_3O_2^+$ (M +H$^+$) 232.1081, found 232.1082.

**8-(Dimethylamino)-5-nitroquinoline-2-carbaldehyde (A3).** An oven-dried flask equipped with a stir bar was charged with *N,N*,2-trimethyl-5-nitroquinolin-8-amine (2.31 g, 10 mmol, 1.0 equiv), $SeO_2$ (1.66 g, 15 mmol, 1.5 equiv) and dioxane/$H_2O$ (30 mL/3 ml). The mixture was refluxed at 120 °C for 2.5 hours. Then, the mixture was added second portion of $SeO_2$ (1.11 g, 10 mmol, 1.0 equiv). After 2 hours, the mixture was cooled down to room temperature and diluted with DCM, filtered, and concentrated. The residue was purified by chromatography on silica gel (DCM/hexane) to afford the title product (1.57 g, 64%).$^1$H NMR (500 MHz, $CDCl_3$) δ 10.12 (d, *J* = 0.9 Hz, 1H), 9.50 (dd, *J* = 9.1, 0.9 Hz, 1H), 8.56 (d, *J* = 9.2 Hz, 1H),

8.15 (d, $J$ = 9.1 Hz, 1H), 6.84 (d, $J$ = 9.3 Hz, 1H), 3.55 (s, 6H). $^{13}$C NMR (126 MHz, $CDCl_3$) δ 192.24, 155.22, 148.51, 138.66, 133.93, 130.70, 126.72, 120.14, 109.31, 44.85. HRMS calcd for $C_{12}H_{12}N_3O_3^+$ (M +H$^+$) 246.0873, found 246.0878.

**2-(2,6-Diisopropylphenyl)-9-(dimethylamino)-6-nitroimidazo[1,5-*a*]quinolin-2-ium chloride (L6)**. An oven-dried 100 mL pressure vessel equipped with a stir bar was charged with 8-(dimethylamino)-5-nitroquinoline-2-carbaldehyde (2.45 g, 10 mmol, 1.0 equiv), 2,6-diisopropylaniline (1.77 g, 10 mmol, 1.0 equiv), *p*-toluenesulfonic acid (344 mg, 2.0 mmol, 0.2 equiv) and EtOH (25 mL). The mixture was stirred at 95 °C for 24 hours. After the indicated time, the reaction mixture was cooled down to room temperature and concentrated. The residue was dissolved with $ClCH_2OEt$ (15 ml). The mixture was stirred at 100 °C for 24 hours. After the indicated time, the reaction mixture was cooled down to room temperature and concentrated. The residue was purified by chromatography on silica gel ($CH_2Cl_2$/MeOH = 20/1) to afford the title product (2.94 g, 65%). $^1$H NMR (500 MHz, $CDCl_3$) δ 10.78 (d, $J$ = 1.7 Hz, 1H), 8.99 (d, $J$ = 1.8 Hz, 1H), 8.61 (d, $J$ = 10.1 Hz, 1H), 8.29 (d, $J$ = 9.0 Hz, 1H), 8.08 (d, $J$ = 10.2 Hz, 1H), 7.79 (d, $J$ = 9.0 Hz, 1H), 7.62 (t, $J$ = 7.9 Hz, 1H), 7.38 (d, $J$ = 7.9 Hz, 2H), 2.90 (s, 6H), 2.25 (p, $J$ = 6.8 Hz, 2H), 1.21 (dd, $J$ = 6.9, 3.7 Hz, 12H). $^{13}$C NMR (126 MHz, $CDCl_3$) δ 150.26, 145.21, 142.88, 132.37, 130.98, 130.48, 130.13, 126.40, 124.83, 123.77, 122.01, 121.33, 121.14, 120.77, 119.57, 44.09, 28.87, 24.51, 24.42. HRMS calcd for $C_{25}H_{29}N_4O_2^+$ (M – Cl$^-$) 417.2285, found 417.2303.

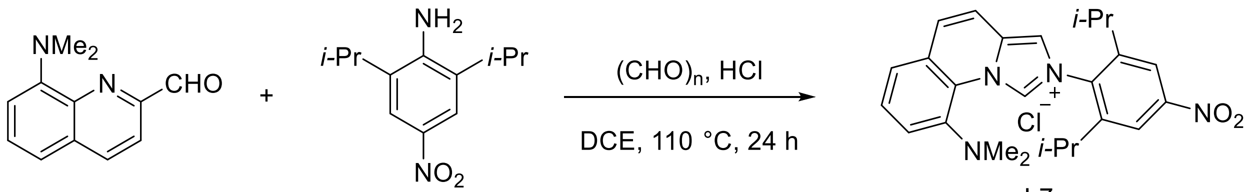

L7

**2-(2,6-Diisopropyl-4-nitrophenyl)-9-(dimethylamino)imidazo[1,5-*a*]quinolin-2-ium chloride (L7)**. An oven-dried 100 mL pressure vessel equipped with a stir bar was charged with 8-(dimethylamino)quinoline-2-carbaldehyde (2.00 g, 10 mmol, 1.0 equiv), 2,6-diisopropyl-4-nitroaniline (2.22 g, 10 mmol, 1.0 equiv), paraformaldehyde (450 mg, 15 mmol,

1.5 equiv) and DCE (25 mL). HCl (4.0 M in dioxane, 5.0 mL, 2.0 equiv) was added dropwise into the reaction at room temperature. The mixture was stirred at 110 °C for 24 hours. After the indicated time, the reaction mixture was cooled down to room temperature and concentrated. The residue was purified by chromatography on silica gel ($CH_2Cl_2$/MeOH = 20/1) to afford the title product (3.31 g, 73%). $^1$H NMR (500 MHz, $CDCl_3$) δ 11.04 (d, $J$ = 1.9 Hz, 1H), 9.38 (d, $J$ = 1.9 Hz, 1H), 8.36 (d, $J$ = 9.6 Hz, 1H), 8.17 (s, 2H), 7.71 (d, $J$ = 1.8 Hz, 3H), 7.60 (d, $J$ = 9.6 Hz, 1H), 2.82 (s, 6H), 2.39 (p, $J$ = 6.8 Hz, 2H), 1.30 (dd, $J$ = 19.8, 6.8 Hz, 12H). $^{13}$C NMR (126 MHz, $CDCl_3$) δ 150.00, 148.22, 146.03, 135.68, 131.41, 130.03, 129.87, 127.96, 127.63, 126.37, 124.35, 123.38, 119.86, 118.80, 117.25, 44.73, 29.40, 24.32, 24.13. HRMS calcd for $C_{25}H_{29}N_4O_2^+$ (M – $Cl^-$) 417.2285, found 417.2299.

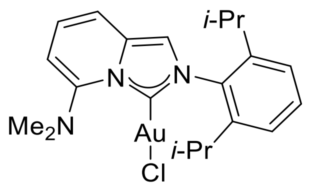


L1AuCl

**L1AuCl.** An oven-dried flask equipped with a stir bar was charged with the corresponding NHC·HCl salt (107 mg, 0.3 mmol, 1.0 equiv),[3] AuClSMe2 (90 mg, 0.3 mmol, 1.0 equiv) and finely powdered K2CO3 (83 mg, 0.6 mmol, 2.0 equiv). The reaction mixture was placed under a positive pressure of argon and subjected to three evacuation/backfilling cycles under high vacuum. Acetone (6.0 ml, 0.05 M) was added and the reaction mixture was stirred at 60 °C for 16 h. After the indicated time, the reaction mixture was diluted with CH2Cl2 (10 mL) and filtered. The solution was collected and concentrated. The title product was obtained by trituration from hexanes as a white solid (150 mg, 90%). Analytical data are consistent with that previously reported in the literature.[3]

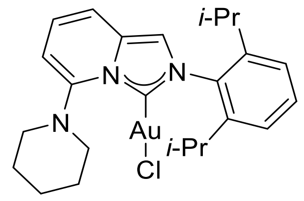


L2AuCl

**L2AuCl.** An oven-dried flask equipped with a stir bar was charged with the corresponding NHC·HCl salt (119 mg, 0.3 mmol, 1.0 equiv),[3] $AuClSMe_2$ (90 mg, 0.3 mmol, 1.0 equiv) and finely powdered $K_2CO_3$ (83 mg, 0.6 mmol, 2.0 equiv). The reaction mixture was placed under a positive pressure of argon and subjected to three evacuation/backfilling cycles under high vacuum. Acetone (6.0 ml, 0.05 M) was added and the reaction mixture was stirred at 60 °C for 16 h. After the indicated time, the reaction mixture was diluted with $CH_2Cl_2$ (200 mL) and filtered. The solution was collected and concentrated. The title product was obtained by trituration from hexanes as a white solid (151 mg, 85%). Analytical data are consistent with that previously reported in the literature.[3]

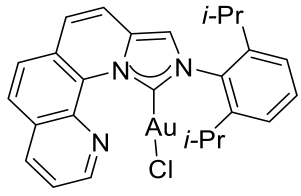


L3AuCl

**L3AuCl.** An oven-dried flask equipped with a stir bar was charged with the corresponding NHC·HCl salt (107 mg, 0.3 mmol, 1.0 equiv), $AuClSMe_2$ (90 mg, 0.3 mmol, 1.0 equiv) and finely powdered $K_2CO_3$ (83 mg, 0.6 mmol, 2.0 equiv). The reaction mixture was placed under a positive pressure of argon and subjected to three evacuation/backfilling cycles under high vacuum. Acetone (6.0 ml, 0.05 M) was added and the reaction mixture was stirred at 60 °C for 16 h. After the indicated time, the reaction mixture was diluted with $CH_2Cl_2$ (10 mL) and filtered. The solution was collected and concentrated. The title product was obtained by trituration from hexanes as a white solid (110 mg, 60%). $^1$H NMR (500 MHz, $CDCl_3$) δ 9.19 (dd, $J$ = 4.2, 1.8 Hz, 1H), 8.24 (dd, $J$ = 8.2, 1.8 Hz, 1H), 7.90 (d, $J$ = 8.4 Hz, 1H), 7.76 (d, $J$ = 8.4 Hz, 1H), 7.57 (dd, $J$ = 8.2, 4.1 Hz, 1H), 7.52 (dd, $J$ = 8.6, 7.4 Hz, 2H), 7.49 (s, 1H), 7.39

(d, $J$ = 9.3 Hz, 1H), 7.31 (d, $J$ = 7.8 Hz, 2H), 2.40 (p, $J$ = 6.8 Hz, 2H), 1.38 (d, $J$ = 6.8 Hz, 6H), 1.14 (d, $J$ = 6.9 Hz, 6H). $^{13}$C NMR (126 MHz, $CDCl_3$) δ 176.46, 150.22, 145.49, 139.81, 135.92, 135.76, 131.58, 130.57, 130.16, 129.47, 127.40, 127.05, 126.48, 125.42, 124.17, 122.57, 117.62, 114.26, 28.59, 24.55, 24.17. HRMS calcd for $C_{26}H_{26}AuClN_3^+$ (M + $H^+$) 612.1475, found 612.1491.

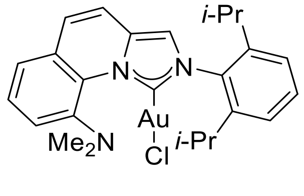


L4AuCl

**L4AuCl.** An oven-dried flask equipped with a stir bar was charged with the corresponding NHC·HCl salt (122 mg, 0.3 mmol, 1.0 equiv),[4] $Ag_2O$ (83.4 mg, 0.36 mmol, 1.2 equiv), NaCl (35 mg, 0.6 mmol, 2.0 equiv), KCl (45 mg, 0.6 mmol, 2.0 equiv) and 4Å MS (240 mg). The reaction mixture was placed under a positive pressure of argon and subjected to three evacuation/backfilling cycles under high vacuum. Anhydrous THF (4.0 ml) was added and the reaction mixture was stirred at 90 °C for 16 h. After the indicated time, the reaction mixture was diluted with $CH_2Cl_2$ (10 mL) and filtered. The solution was collected and concentrated. The NHCAgCl intermediate was obtained by trituration from hexanes as a brown solid (125 mg, 81%). An oven-dried flask equipped with a stir bar was charged with the above NHCAgCl (154 mg, 0.3 mmol, 1.0 equiv), DCM (4 ml) and $AuClSMe_2$ (90 mg, 0.3 mmol, 1.0 equiv) was added. The mixture was stirred at room temperature for 16 hours. The solution was filtered and concentrated. The residue was purified by chromatography on silica gel to afford the title product (172 mg, 95%). Analytical data are consistent with that previously reported in the literature.[4]

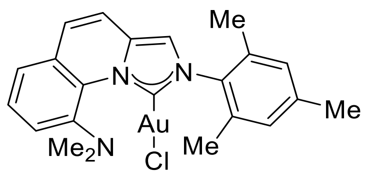


L5AuCl

**L5AuCl.** An oven-dried flask equipped with a stir bar was charged with the corresponding NHC·HCl salt (110 mg, 0.3 mmol, 1.0 equiv),[4] $Ag_2O$ (83.4 mg, 0.36 mmol, 1.2 equiv), NaCl (35 mg, 0.6 mmol, 2.0 equiv), KCl (45 mg, 0.6 mmol, 2.0 equiv) and 4Å MS (240 mg). The reaction mixture was placed under a positive pressure of argon and subjected to three evacuation/backfilling cycles under high vacuum. Anhydrous THF (4.0 ml) was added and the reaction mixture was stirred at 90 °C for 16 h. After the indicated time, the reaction mixture was diluted with $CH_2Cl_2$ (10 mL) and filtered. The solution was collected and concentrated. The title product was obtained by trituration from hexanes as a brown solid (121 mg, 85%). An oven-dried flask equipped with a stir bar was charged with the corresponding NHCAgCl salt (142 mg, 0.3 mmol, 1.0 equiv), DCM (4 ml) and $AuClSMe_2$ (90 mg, 0.3 mmol, 1.0 equiv) was added. The mixture was stirred at room temperature for 16 hours. The solution was filtered and concentrated. The residue was purified by chromatography on silica gel to afford the title product (145 mg, 86%). Analytical data are consistent with that previously reported in the literature.[4]

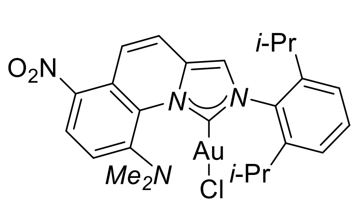


L6AuCl

**L6AuCl.** An oven-dried flask equipped with a stir bar was charged with the corresponding NHC·HCl salt (107 mg, 0.3 mmol, 1.0 equiv), $AuClSMe_2$ (90 mg, 0.3 mmol, 1.0 equiv) and finely powdered KO*t*Bu (41 mg, 0.36 mmol, 1.2 equiv). The reaction mixture was placed under a positive pressure of argon and subjected to three evacuation/backfilling cycles under high vacuum. THF (6.0 ml, 0.05 M) was added and the reaction mixture was stirred at room

temperature for 16 h. After the indicated time, the reaction mixture was diluted with $CH_2Cl_2$ (10 mL) and filtered. The solution was collected and concentrated. The title product was obtained by chromatography on silica gel to afford the title product (88 mg, 45%). $^1$H NMR (500 MHz, $CDCl_3$) δ 8.24 (d, $J$ = 10.0 Hz, 1H), 8.18 (d, $J$ = 9.3 Hz, 1H), 7.53 (t, $J$ = 7.8 Hz, 1H), 7.44 (d, $J$ = 10.0 Hz, 1H), 7.37 (s, 1H), 7.30 (d, $J$ = 7.9 Hz, 2H), 7.21 (d, $J$ = 9.3 Hz, 1H), 2.88 (s, 6H), 2.29 (p, $J$ = 6.8 Hz, 2H), 1.33 (d, $J$ = 6.9 Hz, 6H), 1.14 (d, $J$ = 6.9 Hz, 6H). $^{13}$C NMR (126 MHz, $CDCl_3$) δ 174.72, 149.50, 145.37, 137.53, 134.84, 130.98, 130.21, 125.46, 124.38, 121.08, 120.66, 118.15, 116.62, 114.94, 42.21, 28.59, 24.73, 24.12. HRMS calcd for $C_{25}H_{28}AuClKN_4O_2^+$ (M + $K^+$) 687.1198, found 687.1232.

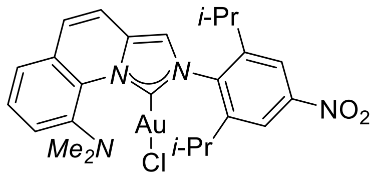


L7AuCl

**L7AuCl.** An oven-dried flask equipped with a stir bar was charged with the corresponding NHC·HCl salt (107 mg, 0.3 mmol, 1.0 equiv), $AuClSMe_2$ (90 mg, 0.3 mmol, 1.0 equiv) and finely powdered $K_2CO_3$ (83 mg, 0.6 mmol, 2.0 equiv). The reaction mixture was placed under a positive pressure of argon and subjected to three evacuation/backfilling cycles under high vacuum. Acetone (6.0 ml, 0.05 M) was added and the reaction mixture was stirred at 60 °C for 16 h. After the indicated time, the reaction mixture was diluted with $CH_2Cl_2$ (10 mL) and filtered. The solution was collected and concentrated. The title product was obtained by chromatography on silica gel to afford the title product (155 mg, 80%). $^1$H NMR (500 MHz, $CDCl_3$) δ 8.11 (s, 2H), 7.40 (t, $J$ = 7.8 Hz, 1H), 7.33 – 7.28 (m, 1H), 7.25 – 7.19 (m, 2H), 7.19 (s, 2H), 2.65 (s, 6H), 2.42 (p, $J$ = 6.9 Hz, 2H), 1.35 (d, $J$ = 6.8 Hz, 6H), 1.14 (d, $J$ = 6.9 Hz, 6H). $^{13}$C NMR (126 MHz, $CDCl_3$) δ 174.42, 149.44, 148.24, 145.52, 140.50, 131.94, 128.37, 127.67, 127.37, 124.55, 122.19, 120.89, 119.67, 114.46, 113.10, 42.88, 29.06, 24.41, 23.77. HRMS calcd for $C_{25}H_{29}AuClN_4O_2^+$ (M + $H^+$) 649.1639, found 649.1669.

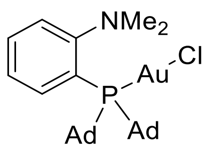


L8AuCl

**L8AuCl.** In a glovebox, Me-Dalphos (421.6 mg, 1.0 mmol) and $Me_2SAuCl$ (294.6 mg, 1.0 mmol) were added to a 25 mL round-bottom flask, followed by $CH_2Cl_2$ (5 mL). The reaction mixture was stirred at room temperature in the dark for 2 h. The flask was then removed from the glovebox, and the reaction mixture was filtered through a pad of Celite and washed with $CH_2Cl_2$. The combined filtrates were concentrated under reduced pressure at room temperature to afford the crude product. Recrystallization from $CH_2Cl_2$/hexanes afforded L8AuCl as a white solid (569.0 mg, 87% yield). Analytical data are consistent with that previously reported in the literature.[5]

## Additional Optimization Studies

**Table S1.** Effect of Solvents in L6AuCl-catalyzed Synthesis of Benzocyclobutenes.[a]

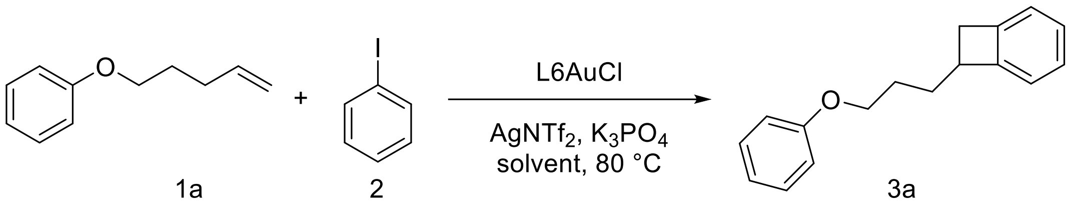


| **Entry** | **Solvent** | **yield of 3a (%)** |
|---|---|---|
| 1 | DCE | 79 |
| 2 | dioxane | 0 |
| 3 | DCM | 56 |
| 4 | THF | 0 |
| 5 | MeOH | 0 |
| 6 | acetone | 0 |
| 7 | toluene | 0 |
| 8 | EA | 0 |

[a]Conditions: **1a** (2.0 equiv), **2** (1.0 equiv), L6AuCl (10 mol%), $AgNTf_2$ (1.2 equiv), $K_3PO_4$ (0.5 equiv), solvent (0.125 M), 80 °C, 16 h.

**Table S2.** Effect of silver salts in L6AuCl-catalyzed Synthesis of Benzocyclobutenes.[a]

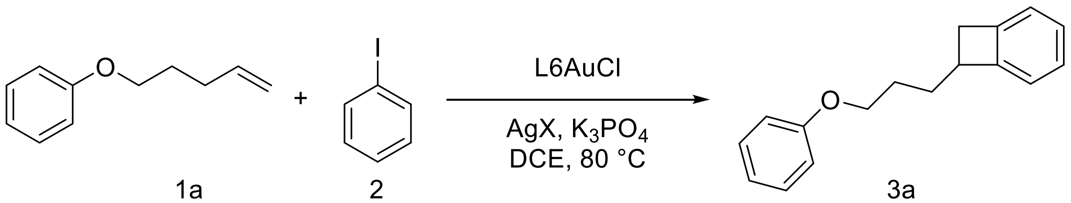


1a 2 3a

| Entry | Ag salt | yield of 3a (%) |
|---|---|---|
| 1 | $AgNTf_2$ | 79 |
| 2 | $AgSbF_6$ | 0 |
| 3 | AgOTf | 66 |
| 4 | AgOAc | 0 |
| 5 | AgF | 0 |
| 6 | $Ag_2O$ | 0 |

[a]Conditions: **1a** (2.0 equiv), **2** (1.0 equiv), L6AuCl (10 mol%), AgX (1.2 equiv), $K_3PO_4$ (0.5 equiv), DCE (0.125 M), 80 °C, 16 h.

**Table S3.** Effect of base in L6AuCl-catalyzed Synthesis of Benzocyclobutenes.[a]

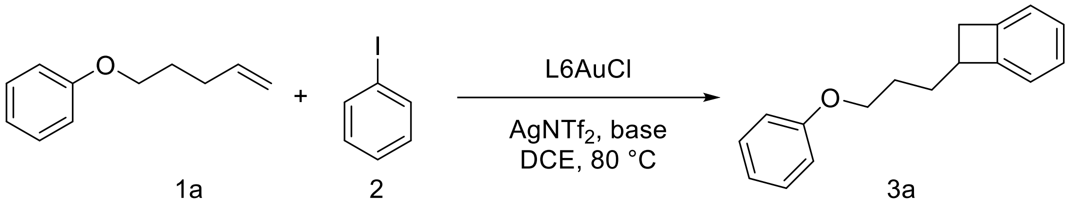


1a 2 3a

| Entry | Base | yield of 3a (%) |
|---|---|---|
| 1 | $K_3PO_4$ | 79 |
| 2 | pyridine | 0 |
| 3 | $NEt_3$ | 39 |
| 4 | $K_2CO_3$ | 0 |
| 5 | $Cs_2CO_3$ | 0 |
| 6 | $NaHCO_3$ | 45 |
| 7 | DIEA | 0 |
| 8 | NaO*t*Bu | 0 |

[a]Conditions: **1a** (2.0 equiv), **2** (1.0 equiv), L6AuCl (10 mol%), $AgNTf_2$ (1.2 equiv), base (0.5 equiv), DCE (0.125 M), 80 °C, 16 h.

**Table S4.** Effect of equivalent of **1a** in L6AuCl-catalyzed Synthesis of Benzocyclobutenes.[a]

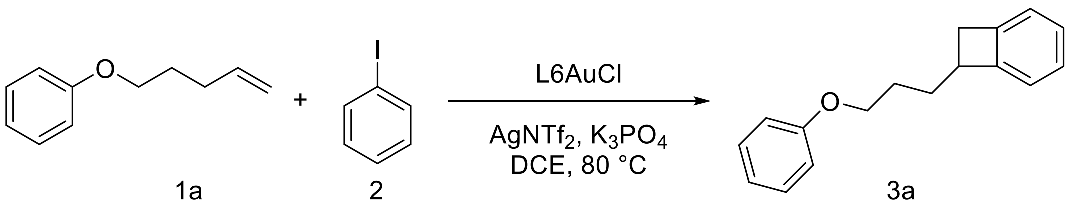


| Entry | 1a (equiv) | yield of 3a (%) |
|---|---|---|
| 1 | 1.0 | 63 |
| 2 | 1.5 | 68 |
| 3 | 2.0 | 79 |
| 4 | 3.0 | 76 |

[a]Conditions: **1a** (x equiv), **2** (1.0 equiv), L6AuCl (10 mol%), $AgNTf_2$ (1.2 equiv), $K_3PO_4$ (0.5 equiv), DCE (0.125 M), 80 °C, 16 h.

**Table S5.** Effect of Catalyst Loading in L6AuCl-catalyzed Synthesis of Benzocyclobutenes.[a]

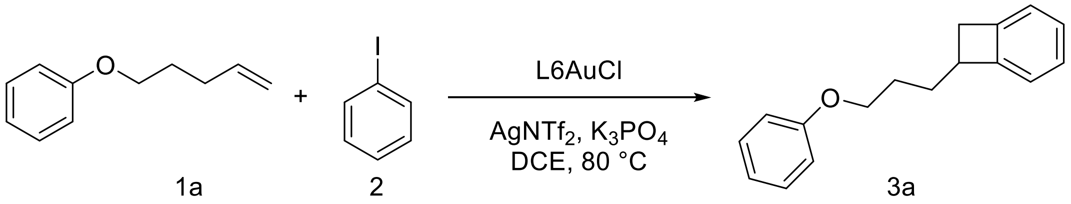


| Entry | L6AuCl (mol%) | yield of 3a (%) |
|---|---|---|
| 1 | 5 | 72 |
| 2 | 10 | 79 |

[a]Conditions: **1a** (2.0 equiv), **2** (1.0 equiv), L6AuCl (x mol%), $AgNTf_2$ (1.2 equiv), $K_3PO_4$ (0.5 equiv), DCE (0.125 M), 80 °C, 16 h.

**General Procedure and Product Characterization.**

An oven-dried microwave vial equipped with a stir bar was charged with iodoarene (0.2 mmol, 1.0 equiv), alkene (0.40 mmol, 2.0 equiv), L6AuCl (0.02 mmol, 10 mol%), $AgNTf_2$ (0.24 mmol, 1.2 equiv), $K_3PO_4$ (0.1 mmol, 0.5 equiv), DCE (0.125 M) at room temperature. Then, the reaction mixture was placed in a preheated oil bath at 80 °C, and stirred for 16 hours at 80 °C. After the indicated time, the reaction mixture was cooled down to room temperature, diluted with $CH_2Cl_2$ (10 mL), filtered, and concentrated. The residue was analyzed by $^1H$ NMR ($CDCl_3$, 500 MHz) and GC-MS using internal standard. Purification by chromatography on silica gel (EtOAc/hexanes or DCM/MeOH) or reverse phase chromatography to afford the title product.

**7-(3-Phenoxypropyl)bicyclo[4.2.0]octa-1,3,5-triene (3a).**

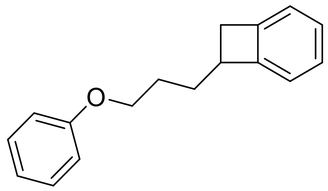


According to the general procedure, the reaction afforded compound **3a** in 78% yield (37.2 mg). $^1H$ NMR (500 MHz, $CDCl_3$) δ 7.30 – 7.26 (m, 2H), 7.23 – 7.14 (m, 2H), 7.14 – 7.05 (m, 2H), 6.92 (dd, *J* = 20.1, 7.7 Hz, 3H), 4.02 (td, *J* = 6.3, 1.9 Hz, 2H), 3.54 (dtd, *J* = 7.5, 5.0, 2.6 Hz, 1H), 3.36 (dd, *J* = 14.0, 5.3 Hz, 1H), 2.79 (dd, *J* = 14.0, 2.5 Hz, 1H), 2.07 – 1.84 (m, 4H). $^{13}C$ NMR (126 MHz, $CDCl_3$) δ 159.04, 149.50, 143.79, 129.45, 127.20, 126.64, 123.08, 121.95, 120.57, 114.49, 67.71, 43.19, 36.16, 30.91, 28.01 HRMS calcd for $C_{17}H_{19}O^+$ (M + $H^+$) 239.1430, found 239.1428.

**(5-Phenoxypent-2-en-1-yl)benzene (3a').**

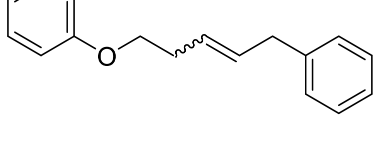

According to the Table 1, the byproduct **3a'** ($E$:$Z$ = 5.4:1) was characterized as following. $^1$H NMR (400 MHz, $CDCl_3$) δ 7.32 – 7.26 (m, 4H, for both isomers), 7.23 – 7.16 (m, 3H, for both isomers), 6.97 – 6.86 (m, 3H, for both isomers), 5.82 – 5.67 (m, 1H, for both isomers), 5.66 – 5.54 (m, 1H, for both isomers), 4.01 (dt, $J$ = 9.2, 6.8 Hz, 2H, for both isomers), 3.46 (d, $J$ = 7.4 Hz, 0.3H, for $Z$-isomer), 3.37 (d, $J$ = 6.6 Hz, 1.63H, for $E$-isomer), 2.71 – 2.62 (m, 0.3H, for $Z$-isomer), 2.55 – 2.50 (m, 1.63H, for $E$-isomer). $^{13}$C NMR (101 MHz, $CDCl_3$) δ 158.91, 140.60, 131.66, 129.47, 129.45, 128.53, 128.48, 128.41, 127.27, 126.02, 125.98, 120.63, 114.56, 114.51, 67.46, 39.11, 32.52. HRMS calcd for $C_{17}H_{19}O^+$ (M + $H^+$) 239.1430, found 239.1429.

**7-(3-(*p*-Tolyloxy)propyl)bicyclo[4.2.0]octa-1,3,5-triene (3b).**

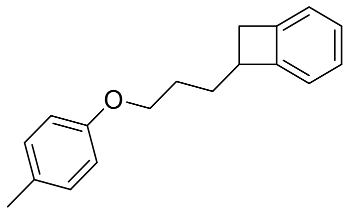


According to the general procedure, the reaction afforded compound **3b** in 56% yield (28.3 mg). $^1$H NMR (500 MHz, $CDCl_3$) δ 7.23 – 7.15 (m, 2H), 7.12 – 7.04 (m, 4H), 6.80 (d, $J$ = 8.6 Hz, 2H), 3.99 (td, $J$ = 6.4, 1.9 Hz, 2H), 3.53 (dq, $J$ = 10.2, 3.8 Hz, 1H), 3.35 (dd, $J$ = 14.0, 5.2 Hz, 1H), 2.78 (dd, $J$ = 14.0, 2.5 Hz, 1H), 2.29 (s, 3H), 2.01 – 1.85 (m, 4H). $^{13}$C NMR (126 MHz, $CDCl_3$) δ 156.92, 149.53, 143.79, 129.88, 129.76, 127.18, 126.63, 123.07, 121.95, 114.35, 67.88, 43.20, 36.16, 30.91, 28.05, 20.48. HRMS calcd for $C_{18}H_{21}O^+$ (M + $H^+$) 253.1587, found 253.1589.

**7-(3-(4-Methoxyphenoxy)propyl)bicyclo[4.2.0]octa-1,3,5-triene (3c)**.

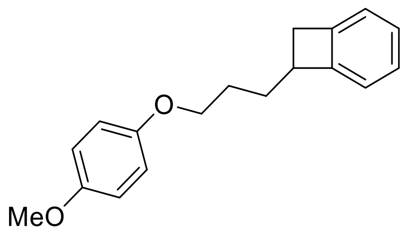

According to the general procedure, the reaction afforded compound **3c** in 58% yield (31.1 mg). $^{1}$H NMR (500 MHz, $CDCl_3$) δ 7.23 – 7.15 (m, 2H), 7.12 – 7.05 (m, 2H), 6.83 (s, 4H), 3.97 (td, *J* = 6.3, 1.8 Hz, 2H), 3.77 (s, 3H), 3.56 – 3.49 (m, 1H), 3.35 (dd, *J* = 14.0, 5.2 Hz, 1H), 2.78 (dd, *J* = 14.0, 2.5 Hz, 1H), 2.03 – 1.83 (m, 4H). $^{13}$C NMR (126 MHz, $CDCl_3$) δ 153.77, 153.23, 149.51, 143.77, 127.17, 126.62, 123.05, 121.92, 115.45, 114.67, 68.51, 55.76, 43.20, 36.14, 30.90, 28.09. HRMS calcd for $C_{18}H_{20}NaO_2^+$ (M + $Na^+$) 291.1356, found 291.1358.

**7-(3-(4-Bromophenoxy)propyl)bicyclo[4.2.0]octa-1,3,5-triene (3d)**.

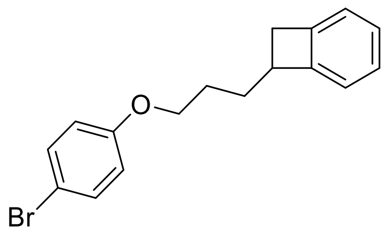

According to the general procedure, the reaction afforded compound **3d** in 78% yield (47.8 mg). $^{1}$H NMR (500 MHz, $CDCl_3$) δ 7.36 (d, *J* = 8.9 Hz, 2H), 7.24 – 7.15 (m, 2H), 7.09 (t, *J* = 6.1 Hz, 2H), 6.78 (d, *J* = 8.6 Hz, 2H), 4.05 – 3.92 (m, 2H), 3.53 (dq, *J* = 10.1, 3.8 Hz, 1H), 3.36 (dd, *J* = 14.0, 5.2 Hz, 1H), 2.78 (dd, *J* = 14.0, 2.5 Hz, 1H), 2.07 – 1.82 (m, 4H). $^{13}$C NMR (126 MHz, $CDCl_3$) δ 158.16, 149.37, 143.74, 132.24, 127.25, 126.67, 123.11, 121.92, 116.29, 112.69, 68.09, 43.12, 36.14, 30.84, 27.88. HRMS calcd for $C_{17}H_{17}BrNaO^+$ (M + $Na^+$) 339.0355, found 339.0356.

**7-(3-(4-(Trifluoromethyl)phenoxy)propyl)bicyclo[4.2.0]octa-1,3,5-triene (3e)**.

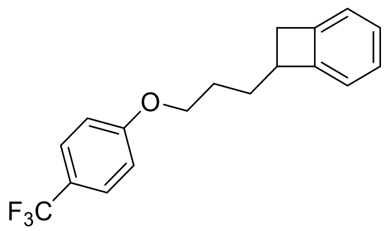


According to the general procedure, the reaction afforded compound **3e** in 52% yield (31.9 mg). $^{1}H$ NMR (500 MHz, $CDCl_3$) δ 7.53 (d, *J* = 8.6 Hz, 2H), 7.24 – 7.14 (m, 2H), 7.09 (t, *J* = 6.2 Hz, 2H), 6.95 (d, *J* = 8.4 Hz, 2H), 4.06 (td, *J* = 6.3, 1.4 Hz, 2H), 3.54 (dtd, *J* = 7.6, 5.1, 2.5 Hz, 1H), 3.36 (dd, *J* = 14.0, 5.3 Hz, 1H), 2.79 (dd, *J* = 13.9, 2.5 Hz, 1H), 2.09 – 1.82 (m, 4H). $^{13}C$ NMR (126 MHz, $CDCl_3$) δ 160.45, 148.26, 142.68, 126.24, 125.88, 125.85, 125.82, 125.79, 125.64, 124.52, 122.37, 122.08, 121.80, 121.55, 120.86, 113.37, 67.04, 42.04, 35.09, 29.77, 26.76. $^{19}F$ NMR (471 MHz, $CDCl_3$) δ -61.45. HRMS calcd for $C_{18}H_{18}F_3O^+$ (M + $H^+$) 307.1304, found 307.1308.

**7-(3-(4-Chlorophenoxy)propyl)bicyclo[4.2.0]octa-1,3,5-triene (3f)**.

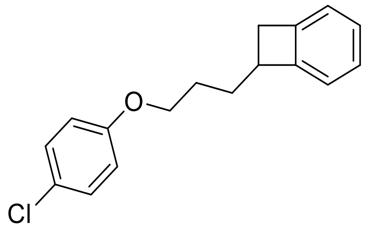


According to the general procedure, the reaction afforded compound **3f** in 67% yield (36.5 mg). $^{1}H$ NMR (500 MHz, $CDCl_3$) δ 7.24 – 7.15 (m, 4H), 7.09 (t, *J* = 6.4 Hz, 2H), 6.82 (d, *J* = 8.9 Hz, 2H), 3.98 (td, *J* = 6.3, 1.5 Hz, 2H), 3.53 (tdd, *J* = 7.6, 5.2, 2.5 Hz, 1H), 3.35 (dd, *J* = 14.0, 5.3 Hz, 1H), 2.78 (dd, *J* = 13.9, 2.5 Hz, 1H), 2.04 – 1.84 (m, 4H). $^{13}C$ NMR (126 MHz, $CDCl_3$) δ 157.67, 149.37, 143.72, 129.28, 127.23, 126.65, 125.42, 123.07, 121.88, 115.77, 68.18, 43.12, 36.12, 30.83, 27.88. HRMS calcd for $C_{17}H_{17}ClNaO^+$ (M + $Na^+$) 295.0860, found 295.0865.

**7-(3-(4-Nitrophenoxy)propyl)bicyclo[4.2.0]octa-1,3,5-triene (3g).**

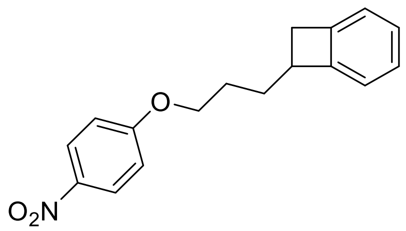

According to the general procedure, the reaction afforded compound **3g** in 68% yield (38.5 mg). $^{1}$H NMR (500 MHz, $CDCl_3$) δ 8.20 (d, *J* = 9.2 Hz, 2H), 7.24 – 7.15 (m, 2H), 7.12 – 7.06 (m, 2H), 6.94 (d, *J* = 9.3 Hz, 2H), 4.11 (t, *J* = 6.1 Hz, 2H), 3.54 (tdd, *J* = 7.6, 5.2, 2.5 Hz, 1H), 3.37 (dd, *J* = 14.0, 5.2 Hz, 1H), 2.79 (dd, *J* = 14.0, 2.5 Hz, 1H), 2.10 – 1.85 (m, 4H). $^{13}$C NMR (126 MHz, $CDCl_3$) δ 164.11, 149.14, 143.68, 141.43, 127.35, 126.73, 125.96, 123.16, 121.88, 114.40, 68.74, 43.00, 36.11, 30.74, 29.72, 27.68. HRMS calcd for $C_{17}H_{18}NO_3^+$ (M + $H^+$) 284.1281, found 284.1282.

**7-(3-(4-Fluorophenoxy)propyl)bicyclo[4.2.0]octa-1,3,5-triene (3h)**.

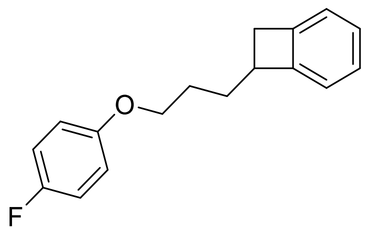

According to the general procedure, the reaction afforded compound **3h** in 66% yield (33.8 mg). $^{1}$H NMR (500 MHz, $CDCl_3$) δ 7.24 – 7.16 (m, 2H), 7.15 – 7.06 (m, 2H), 6.97 (t, *J* = 8.7 Hz, 2H), 6.88 – 6.78 (m, 2H), 3.98 (td, *J* = 6.3, 1.6 Hz, 2H), 3.53 (tdd, *J* = 7.5, 5.2, 2.5 Hz, 1H), 3.36 (dd, *J* = 14.0, 5.3 Hz, 1H), 2.78 (dd, *J* = 14.0, 2.5 Hz, 1H), 2.04 – 1.83 (m, 4H). $^{13}$C NMR (151 MHz, cdcl$_3$) δ 160.58, 159.00, 157.79, 157.77, 152.05, 146.38, 129.85, 129.28, 125.72, 124.55, 118.46, 118.31, 118.04, 117.99, 71.08, 45.77, 38.76, 33.49, 30.61. $^{19}$F NMR (471 MHz, $CDCl_3$) δ -124.32, -124.33, -124.34. HRMS calcd for $C_{17}H_{18}FO^+$ (M + $H^+$) 257.1336, found 257.1339.

**7-(3-(4-(*Tert*-butyl)phenoxy)propyl)bicyclo[4.2.0]octa-1,3,5-triene (3i)**.

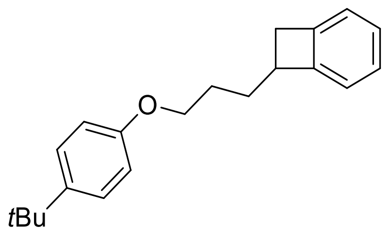


According to the general procedure, the reaction afforded compound **3i** in 60% yield (35.3 mg). $^1$H NMR (500 MHz, $CDCl_3$) δ 7.30 (d, *J* = 8.8 Hz, 2H), 7.24 – 7.16 (m, 2H), 7.13 – 7.06 (m, 2H), 6.85 (d, *J* = 8.8 Hz, 2H), 4.01 (td, *J* = 6.4, 2.2 Hz, 2H), 3.54 (dt, *J* = 7.6, 5.1 Hz, 1H), 3.36 (dd, *J* = 14.0, 5.3 Hz, 1H), 2.79 (dd, *J* = 13.9, 2.5 Hz, 1H), 2.03 – 1.87 (m, 4H). $^{13}$C NMR (126 MHz, $CDCl_3$) δ 156.80, 149.54, 143.80, 143.24, 127.18, 126.63, 126.21, 123.07, 121.95, 113.95, 67.80, 43.21, 36.17, 34.07, 31.56, 30.94, 28.07. HRMS calcd for $C_{21}H_{27}O^+$ (M + $H^+$) 295.2056, found 295.2058.

**1-(4-(3-(Bicyclo[4.2.0]octa-1,3,5-trien-7-yl)propoxy)phenyl)ethan-1-one (3j).**

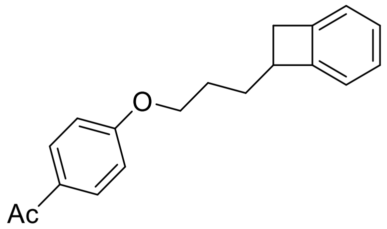


According to the general procedure, the reaction afforded compound **3j** in 64% yield (35.8 mg). $^1$H NMR (500 MHz, $CDCl_3$) δ 7.93 (d, *J* = 8.8 Hz, 2H), 7.20 (dd, *J* = 7.5, 5.5 Hz, 2H), 7.09 (t, *J* = 6.5 Hz, 2H), 6.96 – 6.85 (m, 2H), 4.08 (t, *J* = 6.1 Hz, 2H), 3.54 (dq, *J* = 7.5, 2.5 Hz, 1H), 3.36 (dd, *J* = 14.0, 5.3 Hz, 1H), 2.79 (dd, *J* = 14.0, 2.5 Hz, 1H), 2.56 (s, 3H), 2.09 – 1.83 (m, 4H). $^{13}$C NMR (126 MHz, $CDCl_3$) δ 196.82, 163.01, 149.30, 143.72, 130.62, 130.23, 127.28, 126.69, 123.12, 121.91, 114.14, 68.10, 43.09, 36.13, 30.82, 27.81, 26.35. HRMS calcd for $C_{19}H_{20}NaO_2^+$ (M + $Na^+$) 303.1356, found 303.1357.

**7-(3-(*O*-Tolyloxy)propyl)bicyclo[4.2.0]octa-1,3,5-triene (3k).**

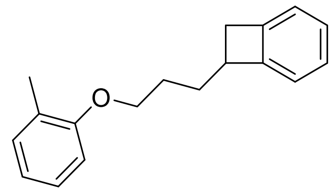

According to the general procedure, the reaction afforded compound **3k** in 60% yield (30.2 mg). $^{1}H$ NMR (500 MHz, $CDCl_3$) δ 7.23 – 7.05 (m, 5H), 6.88 – 6.77 (m, 2H), 4.02 (tt, *J* = 6.8, 3.5 Hz, 2H), 3.55 (dq, *J* = 10.0, 4.1 Hz, 1H), 3.36 (dd, *J* = 13.9, 5.3 Hz, 1H), 2.79 (dd, *J* = 13.9, 2.5 Hz, 1H), 2.23 (s, 3H), 2.07 – 1.86 (m, 4H). $^{13}C$ NMR (126 MHz, $CDCl_3$) δ 157.16, 149.55, 143.79, 130.61, 127.18, 126.84, 126.72, 126.63, 123.08, 121.92, 120.16, 110.87, 67.74, 43.22, 36.18, 31.03, 28.10, 16.28. HRMS calcd for $C_{18}H_{21}O^+$ (M + $H^+$) 253.1587, found 253.1591.

**7-(3-(2-Methoxyphenoxy)propyl)bicyclo[4.2.0]octa-1,3,5-triene (3l).**

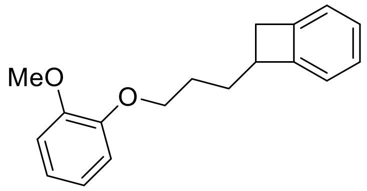


According to the general procedure, the reaction afforded compound **3l** in 60% yield (32.2 mg). $^{1}H$ NMR (500 MHz, $CDCl_3$) δ 7.23 – 7.14 (m, 2H), 7.08 (dd, *J* = 8.9, 7.0 Hz, 2H), 6.90 (d, *J* = 3.2 Hz, 4H), 4.08 (td, *J* = 6.8, 2.5 Hz, 2H), 3.86 (s, 3H), 3.57 – 3.49 (m, 1H), 3.35 (dd, *J* = 14.0, 5.3 Hz, 1H), 2.78 (dd, *J* = 13.9, 2.5 Hz, 1H), 2.04 (ddq, *J* = 20.8, 13.8, 6.8 Hz, 2H), 1.88 (q, *J* = 7.8 Hz, 2H). $^{13}C$ NMR (126 MHz, $CDCl_3$) δ 149.48, 148.59, 143.76, 127.16, 126.61, 123.02, 121.93, 121.01, 120.88, 113.38, 112.03, 68.96, 55.98, 43.19, 36.11, 30.75, 27.94. HRMS calcd for $C_{18}H_{21}O_2^+$ (M + $H^+$) 269.1536, found 269.1545.

**7-(3-(3-(Trifluoromethyl)phenoxy)propyl)bicyclo[4.2.0]octa-1,3,5-triene (3m).**

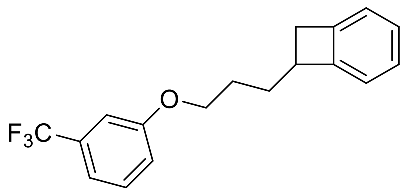

According to the general procedure, the reaction afforded compound **3m** in 59% yield (36.1 mg). $^{1}$H NMR (500 MHz, $CDCl_3$) δ 7.38 (t, *J* = 8.0 Hz, 1H), 7.19 (td, *J* = 7.2, 5.0 Hz, 3H), 7.15 – 7.01 (m, 4H), 4.10 – 4.00 (m, 2H), 3.54 (dtd, *J* = 7.6, 5.2, 2.6 Hz, 1H), 3.37 (dd, *J* = 14.0, 5.3 Hz, 1H), 2.79 (dd, *J* = 14.0, 2.5 Hz, 1H), 2.08 – 1.85 (m, 4H). $^{13}$C NMR (126 MHz, $CDCl_3$) δ 158.12, 148.29, 142.69, 130.91, 130.65, 128.89, 126.22, 125.64, 124.05, 122.07, 121.89, 120.87, 116.95, 116.22, 116.19, 110.18, 110.15, 67.09, 42.05, 35.08, 29.79, 26.79. $^{19}$F NMR (471 MHz, $CDCl_3$) δ -62.70. HRMS calcd for $C_{18}H_{18}F_3O^+$ (M + $H^+$) 307.1304, found 307.1318.

**7-(3-(3,5-Dimethylphenoxy)propyl)bicyclo[4.2.0]octa-1,3,5-triene (3n).**

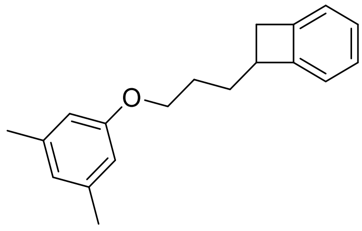


According to the general procedure, the reaction afforded compound **3n** in 69% yield (36.7 mg). $^{1}$H NMR (500 MHz, $CDCl_3$) δ 7.24 – 7.17 (m, 2H), 7.13 – 7.06 (m, 2H), 6.60 (s, 1H), 6.54 (s, 2H), 4.00 (td, *J* = 6.4, 1.8 Hz, 2H), 3.54 (dtd, *J* = 7.6, 5.1, 2.6 Hz, 1H), 3.36 (dd, *J* = 14.0, 5.2 Hz, 1H), 2.79 (dd, *J* = 13.9, 2.5 Hz, 1H), 2.29 (s, 6H), 2.05 – 1.84 (m, 4H). $^{13}$C NMR (126 MHz, $CDCl_3$) δ 159.11, 149.55, 143.80, 139.18, 127.18, 126.63, 123.07, 122.36, 121.96, 112.27, 67.61, 43.19, 36.17, 30.93, 28.06, 21.47. HRMS calcd for $C_{19}H_{23}O^+$ (M + $H^+$) 267.1743, found 267.1745.

**1-(3-(Bicyclo[4.2.0]octa-1,3,5-trien-7-yl)propoxy)naphthalene (3o)**.

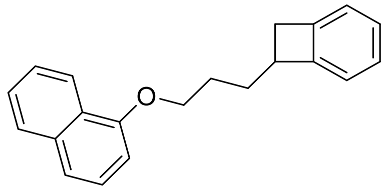

According to the general procedure, the reaction afforded compound **3o** in 78% yield (44.9 mg). $^1$H NMR (500 MHz, $CDCl_3$) δ 8.32 – 8.24 (m, 1H), 7.80 (dd, *J* = 7.5, 1.8 Hz, 1H), 7.52 – 7.34 (m, 4H), 7.25 – 7.18 (m, 2H), 7.15 – 7.12 (m, 1H), 7.12 – 7.08 (m, 1H), 6.82 (d, *J* = 7.4 Hz, 1H), 4.21 (tt, *J* = 6.3, 3.2 Hz, 2H), 3.61 (tdd, *J* = 7.7, 5.1, 2.5 Hz, 1H), 3.39 (dd, *J* = 14.0, 5.3 Hz, 1H), 2.83 (dd, *J* = 14.0, 2.5 Hz, 1H), 2.20 – 1.97 (m, 4H). $^{13}$C NMR (126 MHz, $CDCl_3$) δ 154.81, 149.52, 143.81, 134.52, 127.44, 127.23, 126.68, 126.36, 125.91, 125.72, 125.12, 123.11, 122.09, 121.96, 120.07, 104.52, 67.99, 43.26, 36.21, 31.14, 28.07. HRMS calcd for $C_{21}H_{21}O^+$ (M + $H^+$) 289.1587, found 289.1588.

**2-(3-(Bicyclo[4.2.0]octa-1,3,5-trien-7-yl)propoxy)naphthalene (3p)**.

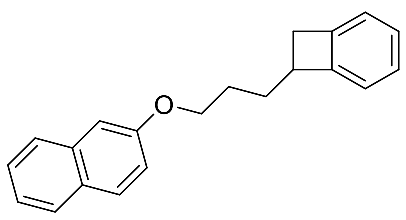


According to the general procedure, the reaction afforded compound **3p** in 69% yield (39.7 mg). $^1$H NMR (500 MHz, $CDCl_3$) δ 7.79 – 7.69 (m, 3H), 7.46 – 7.40 (m, 1H), 7.36 – 7.31 (m, 1H), 7.24 – 7.18 (m, 2H), 7.17 – 7.07 (m, 4H), 4.15 (td, *J* = 6.4, 1.6 Hz, 2H), 3.58 (tdd, *J* = 7.7, 5.2, 2.5 Hz, 1H), 3.38 (dd, *J* = 14.0, 5.3 Hz, 1H), 2.81 (dd, *J* = 13.9, 2.5 Hz, 1H), 2.14 – 1.90 (m, 4H). $^{13}$C NMR (126 MHz, $CDCl_3$) δ 157.02, 149.50, 143.80, 134.62, 129.37, 128.93, 127.66, 127.23, 126.71, 126.67, 126.33, 123.52, 123.10, 121.97, 119.01, 106.56, 67.86, 43.21, 36.19, 30.97, 27.98. HRMS calcd for $C_{21}H_{21}O^+$ (M + $H^+$) 289.1587, found 289.1589.

**7-(3-(Perfluorophenoxy)propyl)bicyclo[4.2.0]octa-1,3,5-triene (3q).**

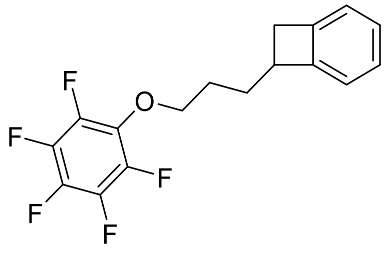

According to the general procedure, the reaction afforded compound **3q** in 46% yield (30.1 mg). $^1$H NMR (500 MHz, $CDCl_3$) δ 7.20 (dq, *J* = 8.7, 6.8 Hz, 2H), 7.11 – 7.05 (m, 2H), 4.21 (t, *J* = 6.2 Hz, 2H), 3.52 (tdd, *J* = 7.5, 5.2, 2.5 Hz, 1H), 3.36 (dd, *J* = 14.0, 5.3 Hz, 1H), 2.78 (dd, *J* = 13.9, 2.5 Hz, 1H), 2.07 – 1.84 (m, 4H). $^{13}$C NMR (126 MHz, $CDCl_3$) δ 148.10, 142.64, 141.80, 139.84, 138.10, 137.17, 135.97, 135.23, 132.70, 126.26, 125.65, 122.08, 120.83, 74.65, 41.89, 35.04, 29.31, 27.48. $^{19}$F NMR (471 MHz, $CDCl_3$) δ -156.83, -156.84, -156.85, -156.88, -156.89, -163.44, -163.45, -163.46, -163.49, -163.50, -163.70, -163.74. HRMS calcd for $C_{17}H_{13}F_5NaO^+$ (M + $Na^+$) 351.0779, found 351.0787.

**3-(Bicyclo[4.2.0]octa-1(6),2,4-trien-7-yl)propyl benzoate (3r)**.

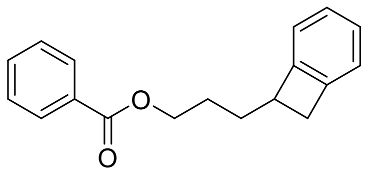

According to the general procedure, the reaction afforded compound **3r** in 93% yield (49.5 mg). $^1$H NMR (500 MHz, $CDCl_3$) δ 8.05 (d, *J* = 7.7 Hz, 2H), 7.56 (t, *J* = 7.4 Hz, 1H), 7.44 (t, *J* = 7.6 Hz, 2H), 7.20 (p, *J* = 7.4 Hz, 2H), 7.10 (dd, *J* = 11.9, 6.6 Hz, 2H), 4.39 (t, *J* = 6.5 Hz, 2H), 3.58 – 3.48 (m, 1H), 3.37 (dd, *J* = 14.0, 5.2 Hz, 1H), 2.79 (dd, *J* = 14.0, 2.5 Hz, 1H), 2.05 – 1.82 (m, 4H). $^{13}$C NMR (126 MHz, $CDCl_3$) δ 166.68, 149.29, 143.72, 132.88, 130.43, 129.58, 128.36, 127.27, 126.69, 123.11, 121.94, 65.01, 43.08, 36.15, 30.91, 27.50. HRMS calcd for $C_{18}H_{19}O_2^+$ (M + $H^+$) 267.1380, found 267.1383.

**2-(3-(Bicyclo[4.2.0]octa-1,3,5-trien-7-yl)propyl)isoindoline-1,3-dione (3s).**

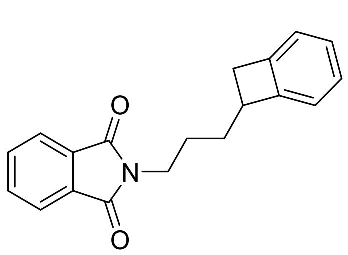

According to the general procedure, the reaction afforded compound **3s** in 70% yield (40.8 mg). $^{1}H$ NMR (500 MHz, $CDCl_3$) δ 7.84 (dd, *J* = 5.4, 3.1 Hz, 2H), 7.71 (dd, *J* = 5.5, 3.0 Hz, 2H), 7.17 (p, *J* = 7.4 Hz, 2H), 7.09 (d, *J* = 6.2 Hz, 1H), 7.05 (d, *J* = 6.5 Hz, 1H), 3.76 (t, *J* = 7.2 Hz, 2H), 3.49 (dq, *J* = 10.1, 3.8 Hz, 1H), 3.32 (dd, *J* = 14.0, 5.3 Hz, 1H), 2.74 (dd, *J* = 13.9, 2.4 Hz, 1H), 1.87 (dp, *J* = 13.6, 6.6 Hz, 2H), 1.76 (q, *J* = 7.8 Hz, 2H). $^{13}C$ NMR (126 MHz, $CDCl_3$) δ 168.46, 149.18, 143.68, 133.90, 132.18, 127.21, 126.65, 123.21, 123.02, 122.05, 42.90, 38.01, 36.09, 31.59, 27.27. HRMS calcd for $C_{19}H_{18}NO_2^+$ (M + $H^+$) 292.1332, found 292.1335.

**1-(3-(Bicyclo[4.2.0]octa-1,3,5-trien-7-yl)propyl)indoline-2,3-dione (3t).**

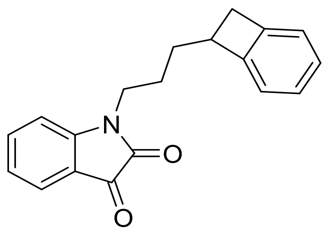


According to the general procedure, the reaction afforded compound **3t** in 86% yield (50.1 mg). $^{1}H$ NMR (500 MHz, $CDCl_3$) δ 7.63 – 7.55 (m, 2H), 7.23 – 7.14 (m, 2H), 7.12 (t, *J* = 7.5 Hz, 1H), 7.06 (t, *J* = 7.3 Hz, 2H), 6.87 (d, *J* = 7.9 Hz, 1H), 3.78 (h, *J* = 7.2 Hz, 2H), 3.51 (q, *J* = 7.3 Hz, 1H), 3.34 (dd, *J* = 14.0, 5.3 Hz, 1H), 2.75 (dd, *J* = 14.0, 2.4 Hz, 1H), 1.91 (qt, *J* = 14.5, 6.5 Hz, 2H), 1.81 (p, *J* = 7.2 Hz, 2H). $^{13}C$ NMR (126 MHz, $CDCl_3$) δ 183.55, 158.17, 150.98, 148.83, 143.60, 138.33, 127.39, 126.76, 125.55, 123.68, 123.14, 121.93, 117.65, 110.07, 42.80, 40.28, 36.03, 31.48, 25.84. HRMS calcd for $C_{19}H_{18}NO_2^+$ (M + $H^+$) 292.1332, found 292.1333.

***N*-(3-(Bicyclo[4.2.0]octa-1(6),2,4-trien-7-yl)propyl)-4-methyl-*N*-phenylbenzenesulfonamide (3u).**

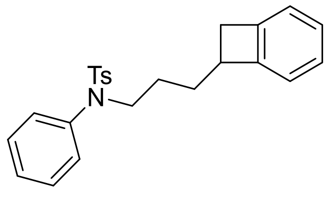

According to the general procedure, the reaction afforded compound **3u** in 78% yield (61.0 mg). $^1$H NMR (500 MHz, $CDCl_3$) δ 7.49 (d, *J* = 7.9 Hz, 2H), 7.36 – 7.32 (m, 3H), 7.27 (t, *J* = 7.0 Hz, 2H), 7.17 (dt, *J* = 22.9, 7.5 Hz, 2H), 7.11 – 7.03 (m, 3H), 6.95 (d, *J* = 7.1 Hz, 1H), 3.61 (t, *J* = 7.0 Hz, 2H), 3.46 – 3.37 (m, 1H), 3.29 (dd, *J* = 14.0, 5.3 Hz, 1H), 2.68 (dd, *J* = 14.0, 2.4 Hz, 1H), 2.45 (s, 3H), 1.76 (tq, *J* = 13.8, 7.2 Hz, 2H), 1.70 – 1.53 (m, 3H). $^{13}$C NMR (126 MHz, $CDCl_3$) δ 149.28, 143.68, 143.30, 139.13, 135.32, 129.38, 129.01, 128.84, 127.88, 127.74, 127.15, 126.57, 123.01, 121.87, 50.47, 42.85, 36.06, 31.16, 26.81, 21.56. HRMS calcd for $C_{24}H_{26}NO_2S^+$ (M + H$^+$) 392.1679, found 392.1678.

***N*-(3-(Bicyclo[4.2.0]octa-1(6),2,4-trien-7-yl)propyl)-4-methyl-*N*-(4-nitrophenyl)benzenesulfonamide (3v).**

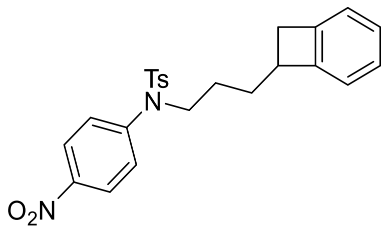


According to the general procedure, the reaction afforded compound **3v** in 82% yield (71.6 mg). $^1$H NMR (500 MHz, $CDCl_3$) δ 8.18 (d, *J* = 8.7 Hz, 2H), 7.43 (d, *J* = 7.9 Hz, 2H), 7.26 (d, *J* = 8.4 Hz, 4H), 7.15 (dt, *J* = 28.1, 7.5 Hz, 2H), 7.04 (d, *J* = 7.2 Hz, 1H), 6.91 (d, *J* = 7.2 Hz, 1H), 3.64 (t, *J* = 7.0 Hz, 2H), 3.38 (t, *J* = 7.6 Hz, 1H), 3.27 (dd, *J* = 14.0, 5.3 Hz, 1H), 2.65 (dd, *J* = 13.9, 2.4 Hz, 1H), 2.43 (s, 3H), 1.79 – 1.68 (m, 2H), 1.68 – 1.56 (m, 2H). $^{13}$C NMR (126 MHz, $CDCl_3$) δ 148.91, 146.42, 145.20, 144.16, 143.56, 134.51, 129.74, 128.53, 127.52, 127.31, 126.65, 124.37, 123.11, 121.74, 49.91, 42.66, 36.02, 31.09, 26.64, 21.59. HRMS calcd for $C_{24}H_{25}N_2O_4S^+$ (M + H$^+$) 437.1530, found 437.1530.

**3-Methyl-8-(3-phenoxypropyl)bicyclo[4.2.0]octa-1,3,5-triene (3w).**

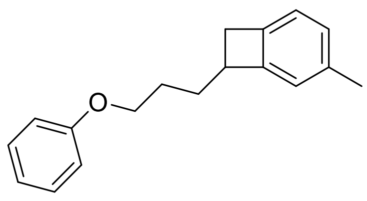

According to the general procedure, the reaction afforded compound **3w** in 85% yield (42.9 mg). $^1$H NMR (500 MHz, $CDCl_3$) δ 7.30 (dd, *J* = 8.7, 7.2 Hz, 2H), 7.05 (d, *J* = 7.5 Hz, 1H), 7.00 – 6.90 (m, 5H), 4.04 (td, *J* = 6.4, 1.4 Hz, 2H), 3.51 (dq, *J* = 10.2, 3.9 Hz, 1H), 3.32 (dd, *J* = 13.8, 5.3 Hz, 1H), 2.75 (dd, *J* = 13.8, 2.4 Hz, 1H), 2.36 (s, 3H), 2.08 – 1.85 (m, 4H). $^{13}$C NMR (126 MHz, $CDCl_3$) δ 159.07, 149.45, 140.41, 136.29, 129.46, 127.93, 122.82, 122.71, 120.57, 114.51, 67.74, 42.83, 35.62, 30.99, 28.05, 22.06. HRMS calcd for $C_{18}H_{21}O^+$ ($M + H^+$) 253.1587, found 253.1590.

**8-Decyl-3-methylbicyclo[4.2.0]octa-1,3,5-triene (3x).**

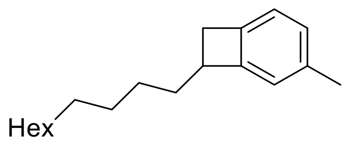


According to the general procedure, the reaction afforded compound **3x** in 72% yield (39.2 mg). $^1$H NMR (500 MHz, $CDCl_3$) δ 7.06 (d, *J* = 7.5 Hz, 1H), 7.00 (d, *J* = 7.5 Hz, 1H), 6.96 (s, 1H), 3.45 (tdd, *J* = 7.8, 5.1, 2.4 Hz, 1H), 3.30 (dd, *J* = 13.8, 5.3 Hz, 1H), 2.77 – 2.67 (m, 1H), 2.38 (s, 3H), 1.72 (dddd, *J* = 8.7, 7.8, 6.2, 2.5 Hz, 2H), 1.56 – 1.47 (m, 2H), 1.36 (dq, *J* = 12.0, 6.8 Hz, 11H), 0.97 – 0.90 (m, 3H). $^{13}$C NMR (126 MHz, $CDCl_3$) δ 150.10, 140.56, 136.11, 127.70, 122.72, 122.66, 43.28, 35.67, 34.62, 31.96, 29.77, 29.67, 29.38, 28.36, 22.74, 22.06, 14.16. HRMS calcd for $C_{19}H_{30}K^+$ ($M + K^+$) 297.1979, found 297.1976.

**3-Bromo-8-(3-phenoxypropyl)bicyclo[4.2.0]octa-1,3,5-triene (3y).**

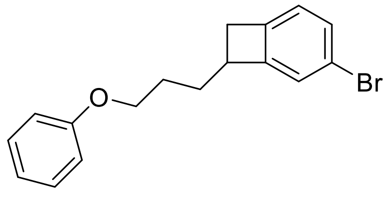


According to the general procedure, the reaction afforded compound **3y** in 46% yield (29.1 mg). $^1$H NMR (500 MHz, $CDCl_3$) δ 7.35 (dd, *J* = 7.9, 1.6 Hz, 1H), 7.28 (dd, *J* = 8.7, 7.2 Hz, 2H), 7.24 (d, *J* = 1.6 Hz, 1H), 6.98 – 6.85 (m, 4H), 4.01 (td, *J* = 6.2, 2.5 Hz, 2H), 3.59 – 3.47 (m, 1H), 3.28 (dd, *J* = 14.2, 5.3 Hz, 1H), 2.71 (dd, *J* = 14.2, 2.5 Hz, 1H), 2.06 – 1.80 (m, 4H). $^{13}$C NMR (126 MHz, $CDCl_3$) δ 158.95, 150.96, 142.20, 130.41, 129.46, 125.41, 124.99, 120.63, 120.51, 114.46, 67.54, 43.03, 35.58, 30.73, 27.84. HRMS calcd for $C_{17}H_{18}BrO^+$ (M + $H^+$) 317.0536, found 317.0540.

**3-Ethyl-8-(3-phenoxypropyl)bicyclo[4.2.0]octa-1,3,5-triene (3z).**

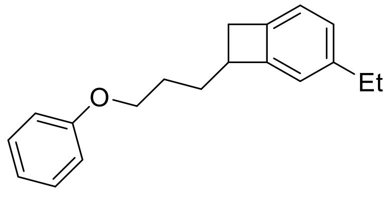


According to the general procedure, the reaction afforded compound **3z** in 78% yield (41.5 mg). $^1$H NMR (500 MHz, $CDCl_3$) δ 7.29 (dd, *J* = 8.7, 7.2 Hz, 2H), 7.06 (d, *J* = 7.4 Hz, 1H), 7.01 (d, *J* = 7.5 Hz, 1H), 6.99 – 6.89 (m, 4H), 4.03 (td, *J* = 6.4, 1.5 Hz, 2H), 3.51 (dq, *J* = 10.1, 3.8 Hz, 1H), 3.32 (dd, *J* = 13.8, 5.2 Hz, 1H), 2.75 (dd, *J* = 13.8, 2.5 Hz, 1H), 2.63 (q, *J* = 7.6 Hz, 2H), 2.05 – 1.87 (m, 4H), 1.24 (t, *J* = 7.6 Hz, 3H). $^{13}$C NMR (126 MHz, $CDCl_3$) δ 159.06, 149.45, 142.98, 140.74, 129.46, 126.90, 122.88, 121.54, 120.57, 114.51, 67.75, 42.87, 35.67, 30.95, 29.58, 28.08, 16.26. HRMS calcd for $C_{19}H_{23}O^+$ (M + $H^+$) 267.1743, found 267.1746.

**3-Methoxy-8-(3-(4-nitrophenoxy)propyl)bicyclo[4.2.0]octa-1,3,5-triene (3aa).**

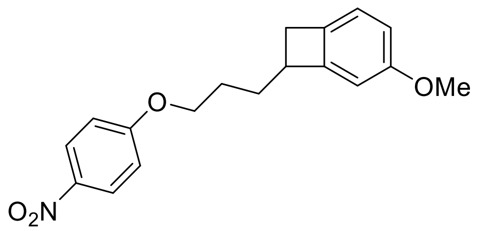

According to the general procedure, the reaction afforded compound **3aa** in 49% yield (30.7 mg). $^1$H NMR (400 MHz, $CDCl_3$) δ 8.20 (d, *J* = 9.2 Hz, 2H), 7.00 (d, *J* = 8.0 Hz, 1H), 6.97 – 6.90 (m, 2H), 6.77 – 6.69 (m, 2H), 4.10 (t, *J* = 6.4 Hz, 2H), 3.78 (s, 3H), 3.46 (tdd, *J* = 7.6, 5.2, 2.5 Hz, 1H), 3.31 (dd, *J* = 14.1, 5.2 Hz, 1H), 2.72 (dd, *J* = 14.0, 2.4 Hz, 1H), 2.09 – 1.81 (m, 4H). $^{13}$C NMR (101 MHz, $CDCl_3$) δ 164.10, 159.70, 144.39, 141.35, 140.72, 125.95, 122.88, 114.37, 113.13, 109.12, 68.73, 55.45, 42.03, 35.49, 31.00, 27.59. HRMS calcd for $C_{18}H_{20}NO_4^+$ (M + $H^+$) 314.1387, found 314.1391.

**2-Nitro-8-(3-phenoxypropyl)bicyclo[4.2.0]octa-1,3,5-triene (3ab).**

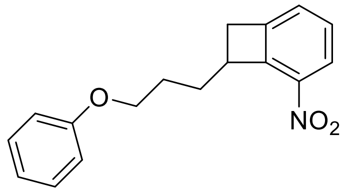

According to the general procedure, the reaction afforded compound **3ab** in 45% yield (25.5 mg). $^1$H NMR (500 MHz, $CDCl_3$) δ 7.87 (d, *J* = 8.2 Hz, 1H), 7.45 (t, *J* = 7.6 Hz, 1H), 7.35 (t, *J* = 7.9 Hz, 1H), 7.26 (s, 1H), 7.21 (d, *J* = 7.7 Hz, 1H), 6.94 (t, *J* = 7.4 Hz, 1H), 6.88 (d, *J* = 8.1 Hz, 2H), 3.96 (q, *J* = 7.1 Hz, 2H), 3.66 (d, *J* = 6.6 Hz, 1H), 2.92 (dd, *J* = 13.5, 7.0 Hz, 1H), 2.72 (dd, *J* = 13.2, 5.6 Hz, 1H), 2.00 – 1.76 (m, 2H), 1.63 (s, 2H). $^{13}$C NMR (151 MHz, $CDCl_3$) δ 161.58, 152.55, 136.48, 136.12, 135.16, 132.04, 129.99, 127.25, 123.15, 117.10, 79.11, 70.30, 40.27, 33.51, 27.68. HRMS calcd for $C_{17}H_{18}NO_3^+$ (M + $H^+$) 284.1281, found 284.1284.

**2,4-Dimethyl-7-(3-(4-nitrophenoxy)propyl)bicyclo[4.2.0]octa-1,3,5-triene (3ac).**

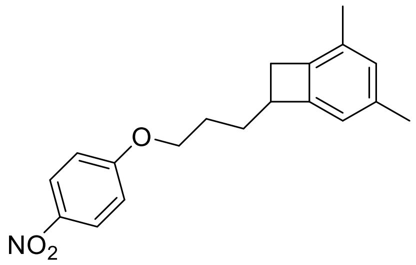


According to the general procedure, the reaction afforded compound **3ac** in 71% yield (44.2 mg). $^1$H NMR (500 MHz, $CDCl_3$) δ 8.20 (d, *J* = 9.0 Hz, 2H), 6.97 – 6.92 (m, 2H), 6.85 (s, 1H), 6.76 (s, 1H), 4.11 (t, *J* = 6.6 Hz, 2H), 3.45 (d, *J* = 6.8 Hz, 1H), 3.24 (dd, *J* = 14.0, 5.1 Hz, 1H), 2.66 (d, *J* = 13.7 Hz, 1H), 2.31 (s, 3H), 2.16 (s, 3H), 2.01 (ddt, *J* = 22.4, 14.0, 7.0 Hz, 2H), 1.91 – 1.82 (m, 2H). $^{13}$C NMR (126 MHz, $CDCl_3$) δ 164.15, 148.66, 141.41, 138.93, 136.99, 132.78, 128.94, 125.95, 119.87, 114.41, 68.79, 41.89, 34.36, 30.82, 27.75, 21.96, 16.53. HRMS calcd for $C_{19}H_{22}NO_3^+$ (M + $H^+$) 312.1594, found 312.1596.

**3,4-Dimethyl-7-(3-phenoxypropyl)bicyclo[4.2.0]octa-1,3,5-triene (3ad).**

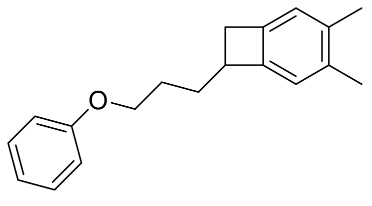


According to the general procedure, the reaction afforded compound **3ad** in 69% yield (36.7 mg). $^1$H NMR (500 MHz, $CDCl_3$) δ 7.33 – 7.29 (m, 2H), 6.99 – 6.85 (m, 5H), 4.07 – 4.00 (m, 2H), 3.51 (d, *J* = 6.8 Hz, 1H), 3.32 (dd, *J* = 13.8, 5.1 Hz, 1H), 2.74 (d, *J* = 13.8 Hz, 1H), 2.27 (s, 6H), 1.99 (m, 2H), 1.87 (m, 2H). $^{13}$C NMR (126 MHz, $CDCl_3$) δ 159.04, 147.00, 141.12, 135.55, 134.90, 129.44, 124.23, 123.17, 120.53, 114.48, 67.74, 42.81, 35.81, 31.18, 28.05, 20.38, 20.33. HRMS calcd for $C_{19}H_{23}O^+$ (M + $H^+$) 267.1743, found 267.1753.

**8-(3-(4-Bromophenoxy)propyl)-3-chlorobicyclo[4.2.0]octa-1,3,5-triene (3ae).**

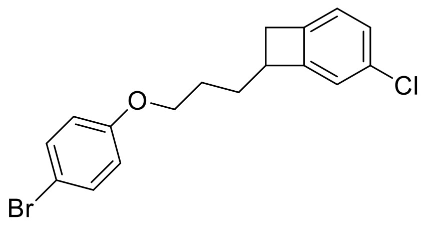


According to the general procedure, the reaction afforded compound **3ae** in 46% yield (32.3 mg). $^1$H NMR (500 MHz, $CDCl_3$) δ 7.37 (d, *J* = 8.9 Hz, 2H), 7.21 – 7.16 (m, 1H), 7.09 (s, 1H), 6.99 (d, *J* = 7.8 Hz, 1H), 6.80 – 6.73 (m, 2H), 3.97 (t, *J* = 6.3 Hz, 2H), 3.54 – 3.47 (m, 1H), 3.30 (dd, *J* = 14.2, 5.3 Hz, 1H), 2.72 (dd, *J* = 14.1, 2.5 Hz, 1H), 2.00 – 1.81 (m, 4H). $^{13}$C NMR (126 MHz, $CDCl_3$) δ 158.09, 150.43, 141.67, 132.49, 132.26, 127.66, 124.57, 122.55, 116.27, 112.77, 67.95, 42.76, 35.46, 30.65, 27.72. HRMS calcd for $C_{17}H_{17}BrClO^+$ (M + $H^+$) 351.0146, found 351.0113.

**7-(3-(4-Bromophenoxy)propyl)-3,4-dichlorobicyclo[4.2.0]octa-1,3,5-triene (3af).**

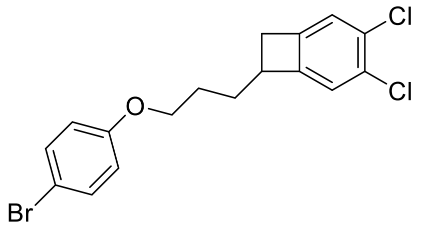


According to the general procedure, the reaction afforded compound **3af** in 45% yield (34.7 mg). $^1$H NMR (500 MHz, $CDCl_3$) δ 7.40 – 7.34 (m, 2H), 7.17 (d, *J* = 5.8 Hz, 2H), 6.80 – 6.70 (m, 2H), 3.97 (t, *J* = 6.1 Hz, 2H), 3.49 (dd, *J* = 7.6, 5.2 Hz, 1H), 3.31 (dd, *J* = 14.4, 5.3 Hz, 1H), 2.75 (dd, *J* = 14.3, 2.6 Hz, 1H), 1.98 – 1.80 (m, 4H). $^{13}$C NMR (126 MHz, $CDCl_3$) δ 158.03, 148.66, 143.13, 132.27, 131.36, 130.93, 125.63, 124.46, 116.25, 112.83, 67.83, 42.68, 35.58, 30.59, 27.58. HRMS calcd for $C_{17}H_{15}BrCl_2KO^+$ (M + $K^+$) 422.9315, found 422.9306.

**3-(Bicyclo[4.2.0]octa-1(6),2,4-trien-7-yl)propyl 2-(1,3-dioxoisoindolin-2-yl)acetate (4a).**

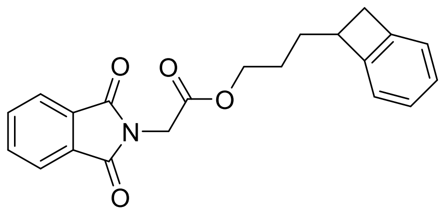

According to the general procedure, the reaction afforded compound **4a** in 84% yield (58.6 mg). $^{1}$H NMR (500 MHz, $CDCl_3$) δ 7.87 (dd, *J* = 5.7, 3.1 Hz, 2H), 7.74 (dd, *J* = 5.8, 3.1 Hz, 2H), 7.18 (p, *J* = 7.7 Hz, 2H), 7.06 (t, *J* = 5.3 Hz, 2H), 4.45 (s, 2H), 4.23 (t, *J* = 6.4 Hz, 2H), 3.50 – 3.41 (m, 1H), 3.31 (dd, *J* = 14.0, 5.3 Hz, 1H), 2.72 (dd, *J* = 14.0, 2.4 Hz, 1H), 1.84 (ddt, *J* = 18.3, 13.5, 6.6 Hz, 2H), 1.77 – 1.69 (m, 2H). $^{13}$C NMR (126 MHz, $CDCl_3$) δ 167.52, 167.30, 149.12, 143.64, 134.25, 132.03, 127.25, 126.68, 123.62, 123.08, 121.90, 65.90, 42.88, 38.96, 36.05, 30.60, 27.15. HRMS calcd for $C_{21}H_{20}NO_4^+$ (M + $H^+$) 350.1387, found 350.1388.

**3-(Bicyclo[4.2.0]octa-1(6),2,4-trien-7-yl)propyl 4-(*N*,*N*-dipropylsulfamoyl)benzoate (4b).**

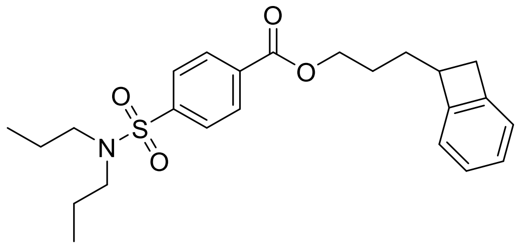

According to the general procedure, the reaction afforded compound **4b** in 96% yield (82.3 mg). $^{1}$H NMR (500 MHz, $CDCl_3$) δ 8.15 (d, *J* = 8.0 Hz, 2H), 7.87 (d, *J* = 8.1 Hz, 2H), 7.25 – 7.16 (m, 2H), 7.09 (t, *J* = 7.0 Hz, 2H), 4.42 (t, *J* = 6.5 Hz, 2H), 3.54 (q, *J* = 6.6 Hz, 1H), 3.37 (dd, *J* = 14.0, 5.3 Hz, 1H), 3.13 – 3.07 (m, 4H), 2.79 (dd, *J* = 13.9, 2.4 Hz, 1H), 2.07 – 1.83 (m, 4H), 1.55 (h, *J* = 7.4 Hz, 4H), 0.87 (t, *J* = 7.4 Hz, 6H). $^{13}$C NMR (126 MHz, $CDCl_3$) δ 165.31, 149.14, 144.22, 143.67, 133.68, 130.21, 127.34, 127.02, 126.73, 123.15, 121.91, 65.67, 49.95, 43.01, 36.14, 30.86, 27.41, 21.96, 11.18. HRMS calcd for $C_{24}H_{32}NO_4S^+$ (M + $H^+$) 430.2047, found 430.2043.

**3-(Bicyclo[4.2.0]octa-1(6),2,4-trien-7-yl)propyl (2*S*)-2-(6-methoxynaphthalen-2-yl)propanoate (4c).**

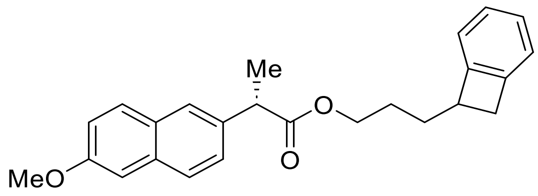


According to the general procedure, the reaction afforded compound **4c** in 92% yield (68.8 mg). $^{1}$H NMR (500 MHz, $CDCl_3$) δ 7.67 (td, *J* = 7.0, 3.4 Hz, 3H), 7.40 (dd, *J* = 8.9, 4.1 Hz, 1H), 7.22 – 7.12 (m, 3H), 7.11 (s, 1H), 7.04 (t, *J* = 6.5 Hz, 1H), 6.98 (d, *J* = 7.0 Hz, 1H), 4.14 (t, *J* = 6.4 Hz, 2H), 3.92 (s, 3H), 3.85 (q, *J* = 7.1 Hz, 1H), 3.40 – 3.32 (m, 1H), 3.23 (dt, *J* = 14.1, 4.9 Hz, 1H), 2.62 (ddd, *J* = 14.2, 8.6, 2.4 Hz, 1H), 1.84 – 1.69 (m, 2H), 1.67 – 1.61 (m, 2H), 1.58 (d, *J* = 7.0 Hz, 3H). $^{13}$C NMR (126 MHz, $CDCl_3$) δ 174.75, 157.63, 149.27, 143.66, 135.80, 133.69, 129.28, 128.94, 127.17, 127.12, 126.60, 126.29, 126.24, 125.98, 125.94, 123.03, 121.87, 121.85, 118.97, 105.61, 64.78, 64.75, 55.33, 45.55, 45.53, 42.92, 42.91, 36.03, 30.64, 27.28, 18.47, 18.45. HRMS calcd for $C_{25}H_{27}O_3^+$ (M + $H^+$) 375.1955, found 375.1956.

**3-(Bicyclo[4.2.0]octa-1(6),2,4-trien-7-yl)propyl 2-(4-(2,2-dichlorocyclopropyl)phenoxy)-2-methylpropanoate (4d).**

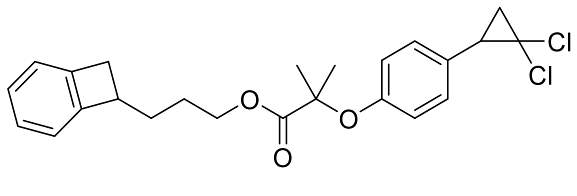


According to the general procedure, the reaction afforded compound **4d** in 45% yield (38.9 mg). $^{1}$H NMR (500 MHz, $CDCl_3$) δ 7.18 (p, *J* = 7.5 Hz, 2H), 7.09 – 7.04 (m, 3H), 7.02 (d, *J* = 7.0 Hz, 1H), 6.80 (d, *J* = 8.3 Hz, 2H), 4.21 (td, *J* = 6.5, 2.2 Hz, 2H), 3.40 (q, *J* = 7.7 Hz, 1H), 3.29 (dd, *J* = 14.0, 5.3 Hz, 1H), 2.80 (dd, *J* = 10.7, 8.3 Hz, 1H), 2.67 (d, *J* = 13.9 Hz, 1H), 1.92 (dd, *J* = 10.7, 7.4 Hz, 1H), 1.87 – 1.78 (m, 1H), 1.78 – 1.69 (m, 2H), 1.64 (q, *J* = 5.6 Hz, 2H), 1.60 (s, 6H). $^{13}$C NMR (126 MHz, $CDCl_3$) δ 174.33, 155.01, 149.14, 143.64, 129.63, 128.05,

127.26, 126.66, 123.09, 123.07, 121.87, 118.43, 79.16, 65.50, 60.88, 42.88, 36.05, 34.82, 30.58, 27.16, 25.84, 25.51, 25.48, 25.46, 25.43. HRMS calcd for $C_{24}H_{27}Cl_2O_3^+$ (M + $H^+$) 433.1332, found 433.1334.

**3-(Bicyclo[4.2.0]octa-1(6),2,4-trien-7-yl)propyl 3-(5-(2-fluorophenyl)-1,2,4-oxadiazol-3-yl)benzoate (4e).**

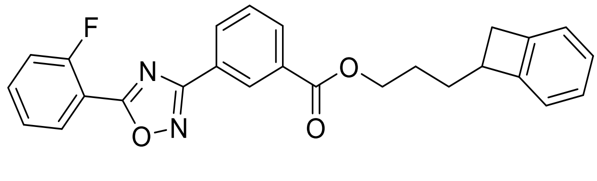

According to the general procedure, the reaction afforded compound **4e** in 81% yield (69.3 mg). $^1$H NMR (500 MHz, $CDCl_3$) δ 8.85 (d, *J* = 1.9 Hz, 1H), 8.38 (dd, *J* = 7.9, 1.5 Hz, 1H), 8.28 – 8.18 (m, 2H), 7.62 (dt, *J* = 12.5, 7.1 Hz, 2H), 7.39 – 7.26 (m, 2H), 7.26 – 7.16 (m, 2H), 7.12 (d, *J* = 6.4 Hz, 1H), 7.10 – 7.05 (m, 1H), 4.45 (t, *J* = 6.6 Hz, 2H), 3.60 – 3.52 (m, 1H), 3.38 (dd, *J* = 14.0, 5.3 Hz, 1H), 2.81 (dd, *J* = 13.9, 2.5 Hz, 1H), 2.10 – 1.93 (m, 2H), 1.89 (q, *J* = 7.7 Hz, 2H). $^{13}$C NMR (126 MHz, $CDCl_3$) δ 173.09, 173.06, 168.12, 165.95, 161.87, 159.80, 149.24, 143.71, 134.79, 134.72, 132.25, 131.76, 131.31, 131.01, 129.08, 128.73, 127.27, 127.24, 126.70, 124.78, 124.75, 123.10, 121.96, 117.30, 117.14, 112.79, 112.70, 65.40, 43.05, 36.14, 30.84, 27.46. $^{19}$F NMR (471 MHz, $CDCl_3$) δ -108.15, -108.16. HRMS calcd for $C_{26}H_{21}FN_2NaO_3^+$ (M + $Na^+$) 451.1428, found 451.1433.

**3-(Bicyclo[4.2.0]octa-1(6),2,4-trien-7-yl)propyl 6-(3-((3r,5r,7r)-adamantan-1-yl)-4-methoxyphenyl)-2-naphthoate (4f).**

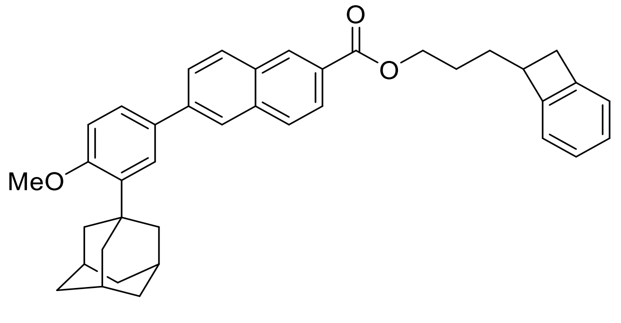

According to the general procedure, the reaction afforded compound **4f** in 90% yield (100.0 mg). $^{1}H$ NMR (500 MHz, $CDCl_3$) δ 8.61 (s, 1H), 8.08 (d, *J* = 8.6 Hz, 1H), 8.04 – 7.97 (m, 2H), 7.92 (d, *J* = 8.5 Hz, 1H), 7.80 (d, *J* = 8.5 Hz, 1H), 7.61 (d, *J* = 2.3 Hz, 1H), 7.55 (dd, *J* = 8.4, 2.3 Hz, 1H), 7.25 – 7.18 (m, 2H), 7.14 (d, *J* = 6.5 Hz, 1H), 7.10 (d, *J* = 6.4 Hz, 1H), 7.00 (d, *J* = 8.3 Hz, 1H), 4.46 (t, *J* = 6.5 Hz, 2H), 3.91 (s, 3H), 3.58 (q, *J* = 7.1 Hz, 1H), 3.39 (dd, *J* = 14.0, 5.3 Hz, 1H), 2.82 (dd, *J* = 14.0, 2.5 Hz, 1H), 2.20 (d, *J* = 2.9 Hz, 6H), 2.12 (s, 3H), 2.04 (tt, *J* = 13.9, 6.9 Hz, 2H), 1.93 (q, *J* = 7.6 Hz, 2H), 1.81 (s, 6H). $^{13}C$ NMR (126 MHz, $CDCl_3$) δ 166.91, 158.94, 149.32, 143.75, 141.37, 139.03, 135.96, 132.59, 131.27, 130.78, 129.72, 128.22, 127.28, 127.18, 126.71, 126.47, 126.00, 125.75, 125.62, 124.75, 123.13, 121.98, 112.13, 65.14, 55.19, 43.13, 40.63, 37.24, 37.15, 36.18, 30.95, 29.14, 27.57. HRMS calcd for $C_{39}H_{41}O_3^+$ (M + $H^+$) 557.3050, found 557.3058.

**2-(1-(3-(Bicyclo[4.2.0]octa-1(6),2,4-trien-7-yl)propyl)-2,6-dioxopiperidin-3-yl)isoindoline-1,3-dione (4g).**

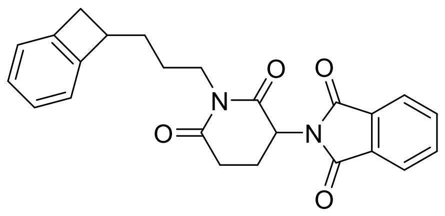

According to the general procedure, the reaction afforded compound **4g** in 91% yield (73.2 mg). $^{1}H$ NMR (500 MHz, $CDCl_3$) δ 7.94 – 7.88 (m, 2H), 7.79 (dd, *J* = 5.5, 3.1 Hz, 2H), 7.19 (t, *J* = 5.5 Hz, 2H), 7.12 (d, *J* = 6.7 Hz, 1H), 7.07 (d, *J* = 6.5 Hz, 1H), 5.01 (dd, *J* = 12.4, 5.4 Hz, 1H), 3.90 (t, *J* = 7.4 Hz, 2H), 3.50 (dt, *J* = 5.9, 3.1 Hz, 1H), 3.34 (dd, *J* = 14.1, 5.2 Hz, 1H), 3.04 – 2.97 (m, 1H), 2.86 – 2.69 (m, 3H), 2.18 – 2.10 (m, 1H), 1.81 – 1.70 (m, 4H). $^{13}C$ NMR (126 MHz, $CDCl_3$) δ 170.84, 168.45, 167.45, 167.43, 149.38, 149.36, 143.75, 143.72, 134.43, 134.40, 131.82, 127.11, 126.61, 123.76, 123.72, 122.97, 122.10, 122.09, 50.19, 42.98, 42.93, 40.68, 36.14, 32.07, 31.60, 31.58, 26.45, 22.07. HRMS calcd for $C_{24}H_{23}N_2O_4^+$ (M + $H^+$) 403.1652, found 403.1641.

**(8*R*,9*S*,13*S*,14*S*)-3-(3-(Bicyclo[4.2.0]octa-1(6),2,4-trien-7-yl)propoxy)-13-methyl-6,7,8,9,11,12,13,14,15,16-decahydro-17*H*-cyclopenta[*a*]phenanthren-17-one (4h).**

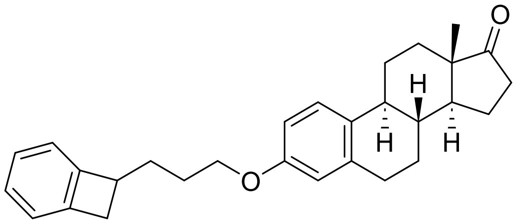


According to the general procedure, the reaction afforded compound **4h** in 78% yield (64.6 mg). $^{1}$H NMR (500 MHz, $CDCl_3$) δ 7.24 – 7.15 (m, 3H), 7.09 (dd, *J* = 9.2, 6.7 Hz, 2H), 6.72 (dd, *J* = 8.5, 2.7 Hz, 1H), 6.65 (d, *J* = 2.7 Hz, 1H), 4.00 (t, *J* = 6.4 Hz, 2H), 3.57 – 3.49 (m, 1H), 3.35 (dd, *J* = 14.0, 5.3 Hz, 1H), 2.97 – 2.84 (m, 2H), 2.82 – 2.75 (m, 1H), 2.51 (dd, *J* = 19.0, 8.7 Hz, 1H), 2.44 – 2.33 (m, 1H), 2.26 (td, *J* = 10.7, 4.4 Hz, 1H), 2.15 (dt, *J* = 18.5, 8.8 Hz, 1H), 2.09 – 1.83 (m, 7H), 1.66 – 1.42 (m, 6H), 0.91 (s, 3H). $^{13}$C NMR (126 MHz, $CDCl_3$) δ 157.09, 149.52, 143.79, 137.74, 131.95, 127.19, 126.63, 126.33, 123.08, 121.94, 114.56, 112.11, 67.77, 50.44, 48.04, 44.01, 43.18, 38.41, 36.17, 35.90, 31.61, 30.91, 29.68, 28.05, 26.59, 25.95, 21.61, 13.88. HRMS calcd for $C_{29}H_{35}O_2^+$ (M + $H^+$) 415.2632, found 415.2632.

**3-(Bicyclo[4.2.0]octa-1(6),2,4-trien-7-yl)propyl 2-(1-(4-chlorobenzoyl)-5-methoxy-2-methyl-1*H*-indol-3-yl)acetate (4i).**

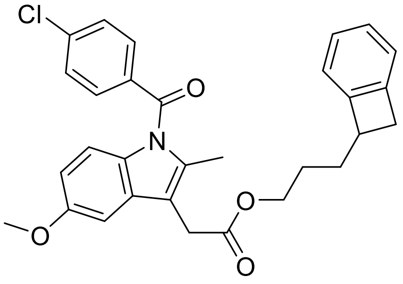


According to the general procedure, the reaction afforded compound **4i** in 90% yield (90.3 mg). $^{1}$H NMR (500 MHz, $CDCl_3$) δ 7.64 (d, *J* = 8.1 Hz, 2H), 7.46 (d, *J* = 8.1 Hz, 2H), 7.18 (dt, *J* = 16.0, 7.5 Hz, 2H), 7.04 (dd, *J* = 18.1, 7.0 Hz, 2H), 6.97 (d, *J* = 2.4 Hz, 1H), 6.86 (d, *J* = 9.0 Hz, 1H), 6.67 (dd, *J* = 9.2, 2.5 Hz, 1H), 4.17 (t, *J* = 6.3 Hz, 2H), 3.81 (s, 3H), 3.66 (s, 2H), 3.42 (d,

*J* = 7.1 Hz, 1H), 3.29 (dd, *J* = 14.0, 5.3 Hz, 1H), 2.67 (dt, *J* = 14.0, 1.6 Hz, 1H), 2.38 (s, 3H), 1.88 – 1.73 (m, 2H), 1.71 (ddd, *J* = 13.8, 8.7, 5.7 Hz, 2H). $^{13}$C NMR (126 MHz, $CDCl_3$) δ 170.94, 168.29, 156.06, 149.14, 143.62, 139.25, 135.91, 133.94, 131.17, 130.82, 130.67, 129.13, 127.26, 126.67, 123.10, 121.83, 114.98, 112.70, 111.66, 101.33, 65.06, 55.69, 42.91, 36.07, 30.75, 30.46, 27.32, 13.38. HRMS calcd for $C_{30}H_{29}ClNO_4^+$ (M + $H^+$) 502.1780, found 502.1778.

**2-(3-(Bicyclo[4.2.0]octa-1,3,5-trien-7-yl)propyl)benzo[*d*]isothiazol-3(2*H*)-one 1,1-dioxide (4j).**

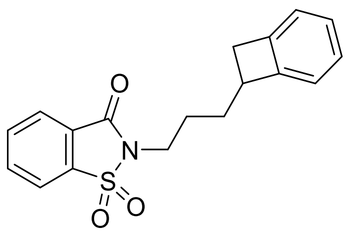


According to the general procedure, the reaction afforded compound **4j** in 93% yield (60.8 mg). $^{1}$H NMR (500 MHz, $CDCl_3$) δ 8.06 (d, *J* = 7.4 Hz, 1H), 7.92 (d, *J* = 7.5 Hz, 1H), 7.85 (dt, *J* = 18.9, 7.4 Hz, 2H), 7.18 (q, *J* = 6.3 Hz, 2H), 7.10 (d, *J* = 6.5 Hz, 1H), 7.08 – 7.04 (m, 1H), 3.85 (td, *J* = 7.4, 2.8 Hz, 2H), 3.52 (dq, *J* = 10.1, 3.8 Hz, 1H), 3.34 (dd, *J* = 14.0, 5.3 Hz, 1H), 2.78 (dd, *J* = 14.0, 2.4 Hz, 1H), 2.11 – 1.98 (m, *J* = 6.6 Hz, 2H), 1.83 (q, *J* = 7.8 Hz, 2H). $^{13}$C NMR (126 MHz, $CDCl_3$) δ 158.98, 149.05, 143.66, 137.73, 134.70, 134.31, 127.47, 127.27, 126.69, 125.17, 123.05, 122.03, 120.92, 42.76, 39.37, 36.09, 31.50, 27.13. HRMS calcd for $C_{18}H_{18}NO_3S^+$ (M + $H^+$) 328.1002, found 328.0998.

***N*-(3-(Bicyclo[4.2.0]octa-1(6),2,4-trien-7-yl)propyl)-*N*-(4-nitro-2-phenoxyphenyl)methanesulfonamide (4k).**

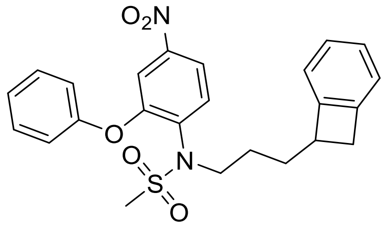

According to the general procedure, the reaction afforded compound **4k** in 74% yield (66.9 mg). $^{1}$H NMR (500 MHz, $CDCl_3$) δ 7.97 (d, *J* = 8.8 Hz, 1H), 7.66 (s, 1H), 7.61 (d, *J* = 8.6 Hz, 1H), 7.48 (t, *J* = 7.8 Hz, 2H), 7.33 (t, *J* = 7.7 Hz, 1H), 7.22 (t, *J* = 7.6 Hz, 1H), 7.16 (t, *J* = 7.5 Hz, 1H), 7.08 (t, *J* = 9.0 Hz, 3H), 6.96 (d, *J* = 7.2 Hz, 1H), 3.83 (t, *J* = 7.2 Hz, 2H), 3.45 (q, *J* = 7.1 Hz, 1H), 3.33 (dd, *J* = 13.9, 5.4 Hz, 1H), 3.05 (s, 3H), 2.72 (d, *J* = 14.0 Hz, 1H), 1.77 (dh, *J* = 29.0, 7.5 Hz, 4H). $^{13}$C NMR (126 MHz, $CDCl_3$) δ 155.53, 154.16, 148.95, 148.09, 143.61, 134.33, 134.25, 130.82, 127.36, 126.69, 125.99, 123.16, 121.80, 119.90, 117.89, 112.40, 50.04, 42.81, 39.70, 36.11, 31.26, 27.58. HRMS calcd for $C_{24}H_{25}N_2O_5S^+$ (M + $H^+$) 453.1479, found 453.1479.

**3-(Bicyclo[4.2.0]octa-1(6),2,4-trien-7-yl)propyl 4-oxo-4*H*-chromene-2-carboxylate (4l).**

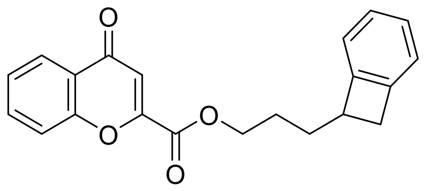

According to the general procedure, the reaction afforded compound **4l** in 83% yield (55.4 mg). $^{1}$H NMR (500 MHz, $CDCl_3$) δ 8.20 (dd, *J* = 8.0, 1.6 Hz, 1H), 7.78 – 7.71 (m, 1H), 7.61 (d, *J* = 8.5 Hz, 1H), 7.46 (t, *J* = 7.6 Hz, 1H), 7.20 (h, *J* = 7.6 Hz, 2H), 7.14 – 7.06 (m, 3H), 4.47 (t, *J* = 6.6 Hz, 2H), 3.54 (q, *J* = 7.2 Hz, 1H), 3.38 (dd, *J* = 14.0, 5.3 Hz, 1H), 2.79 (dd, *J* = 14.0, 2.4 Hz, 1H), 2.00 (ddp, *J* = 21.0, 14.0, 7.1 Hz, 2H), 1.86 (q, *J* = 7.7 Hz, 2H). $^{13}$C NMR (126 MHz, $CDCl_3$) δ 178.48, 160.61, 156.02, 152.16, 148.93, 143.61, 134.77, 127.39, 126.76, 125.95, 125.78, 124.46, 123.17, 121.91, 118.84, 114.86, 66.95, 42.86, 36.10, 30.64, 27.18. HRMS calcd for $C_{21}H_{19}O_4^+$ (M + $H^+$) 335.1278, found 335.1278.

**(3a*R*,6*R*,7a*S*)-1-(3-(Bicyclo[4.2.0]octa-1(6),2,4-trien-7-yl)propyl)-8,8-dimethylhexahydro-3*H*-3a,6-methanobenzo[*c*]isothiazole 2,2-dioxide (4m).**

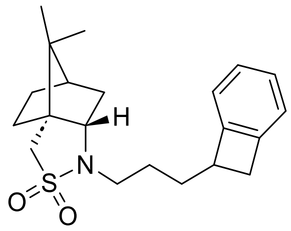


According to the general procedure, the reaction afforded compound **4m** in 90% yield (64.7 mg). $^{1}$H NMR (500 MHz, $CDCl_3$) δ 7.18 (p, *J* = 7.4 Hz, 2H), 7.10 – 7.04 (m, 2H), 3.47 (q, *J* = 7.0 Hz, 1H), 3.32 (dd, *J* = 14.0, 5.3 Hz, 1H), 3.21 – 3.05 (m, 4H), 2.86 – 2.71 (m, 2H), 1.99 (td, *J* = 12.1, 3.5 Hz, 1H), 1.93 – 1.75 (m, 6H), 1.69 (tt, *J* = 13.3, 7.4 Hz, 2H), 1.44 (t, *J* = 9.4 Hz, 1H), 1.33 – 1.23 (m, 1H), 1.15 (d, *J* = 10.3 Hz, 3H), 0.92 (d, *J* = 3.0 Hz, 3H). $^{13}$C NMR (126 MHz, $CDCl_3$) δ 149.36, 149.31, 143.78, 143.72, 127.20, 127.17, 126.63, 126.60, 123.07, 123.05, 121.94, 121.91, 67.83, 67.74, 49.86, 49.79, 47.57, 47.55, 44.65, 44.63, 44.01, 43.93, 42.96, 36.18, 36.14, 35.75, 35.69, 32.25, 32.18, 32.16, 26.91, 26.65, 26.51, 20.42, 20.03. HRMS calcd for $C_{21}H_{30}NO_2S^+$ (M + $H^+$) 360.1992, found 360.1991.

**HRMS Studies for Possible Intermediates**

An oven-dried vial equipped with a stir bar was charged with iodobenzene (0.1 mmol, 1.0 equiv), L6AuCl (0.1 mmol, 1.0 equiv), $AgNTf_2$ (0.12 mmol, 1.2 equiv). Then, the reaction mixture was stirred at room temperature for 10 minutes. After the indicated time, the reaction mixture was analysed by HRMS. The signal peak of oxidative addition and iodide-chloride exchanges intermediate[6] was identified. HRMS calcd for the oxidative addition intermediate [L6AuClPh]+ ($C_{31}H_{33}Au_{Cl}N_4O_2^+$) 725.1952, found 725.1958.

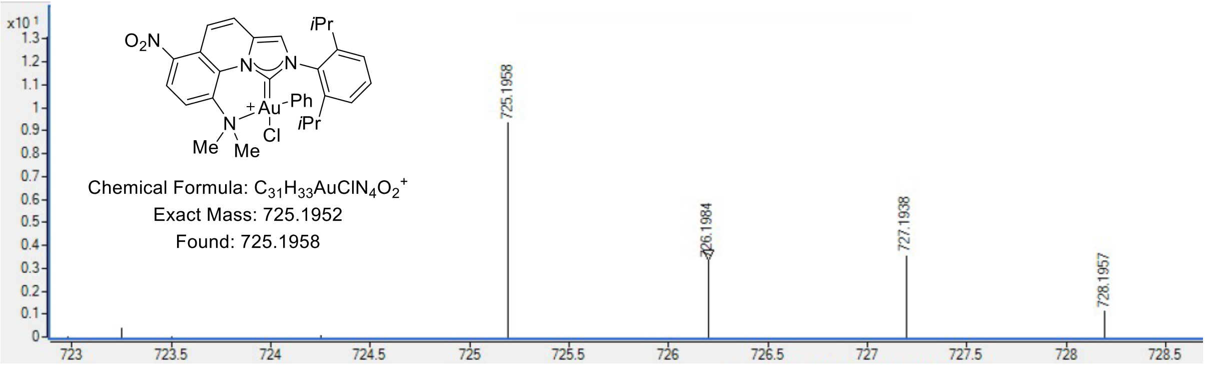

An oven-dried vial equipped with a stir bar was charged with **1a** (0.2 mmol, 2.0 equiv), iodobenzene (0.1 mmol, 1.0 equiv), L6AuCl (0.01 mmol, 10 mol%), $AgNTf_2$ (0.12 mmol, 1.2 equiv), $K_3PO_4$ (0.05 mmol, 0.5 equiv), DCE (0.125 M) at room temperature. Then, the reaction mixture was placed in a preheated oil bath at 80 °C, and stirred for 30 minutes at 80 °C. After the indicated time, the reaction mixture was analysed by HRMS. The signal peak of intermediate IM5 (Fig. 4) was identified. HRMS calcd for the intermediate IM5 ($C_{42}H_{46}AuN_4O_3^+$) 851.3236, found 851.3239.

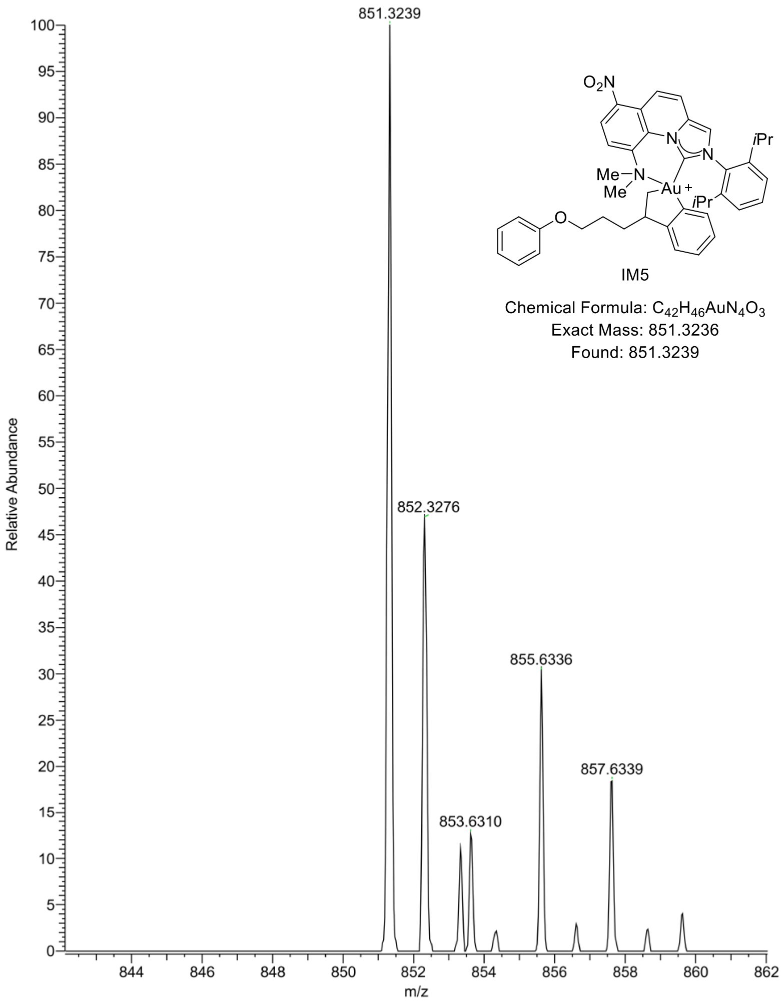

**1 mmol Scale Synthesis**

An oven-dried vial equipped with a stir bar was charged with **1a** (2.0 mmol, 2.0 equiv), iodobenzene (1.0 mmol, 1.0 equiv), L6AuCl (0.1 mmol, 10 mol%), $AgNTf_2$ (1.2 mmol, 1.2 equiv), $K_3PO_4$ (0.5 mmol, 0.5 equiv), DCE (0.125 M) at room temperature. Then, the reaction mixture was placed in a preheated oil bath at 80 °C, and stirred for 16 hours at 80 °C. After the indicated time, the reaction mixture was cooled down to room temperature, diluted with $CH_2Cl_2$ (20 mL), filtered, and concentrated. The residue was analyzed by $^1H$ NMR ($CDCl_3$, 500 MHz) and GC-MS using internal standard. Purification by chromatography on silica gel (EtOAc/hexanes) afforded the product **3a** in 68% yield (162.0 mg).

## Crystallographic Studies

**Table S6.** *Crystal Data and Structure Refinement Summaries for* ***L6AuCl***.

| Compound | **L6AuCl** |
|---|---|
| Chemical formula | $C_{25}H_{28}AuClN_4O_2$ |
| $M_r$ | 648.93 |
| Crystal system, space group | Monoclinic, *P*21/*c* |
| Temperature (K) | 100 |
| *a*, *b*, *c* (Å) | 13.3293 (3), 19.8905 (3), 20.6552 (4) |
| β (°) | 107.936 (2) |
| *V* (Å$^3$) | 5210.10 (18) |
| *Z* | 8 |
| Radiation type | Cu Kα |
| μ (mm$^{-1}$) | 11.78 |
| Crystal size (mm) | 0.38 × 0.25 × 0.12 |
| No. of measured, independent and observed [$I > 2\sigma(I)$] reflections | 74770, 10489, 9892 |
| $R_{int}$ | 0.030 |
| $(\sin \theta/\lambda)_{max}$ (Å$^{-1}$) | 0.628 |
| $R[F^2 > 2\sigma(F^2)]$, $wR(F^2)$, $S$ | 0.051, 0.112, 1.15 |
| No. of reflections | 10489 |
| No. of parameters | 645 |
| H-atom treatment | H-atom parameters constrained |
| $\Delta\rho_{max}$, $\Delta\rho_{min}$ (e Å$^{-3}$) | 1.61, −1.69 |

Computer programs: *CrysAlis PRO* 1.171.43.105a (Rigaku OD, 2024), *SHELXL* 2014/7 (Sheldrick, 2015), Olex2 1.3 (Dolomanov *et al.*, 2009).

**Table S7.** *Crystal Data and Structure Refinement Summaries for* ***4a****.*

| Compound | **4a** |
|---|---|
| Chemical formula | $C_{14}H_{12}NO_4{\cdot}C_7H_{3.58}{\cdot}4.737(H)$ |
| $M_r$ | 350.70 |
| Crystal system, space group | Monoclinic, $P2_1/n$ |
| Temperature (K) | 100 |
| $a$, $b$, $c$ (Å) | 13.5662 (2), 7.4675 (1), 17.0841 (2) |
| β (°) | 91.441 (1) |
| $V$ (Å$^3$) | 1730.17 (4) |
| $Z$ | 4 |
| Radiation type | Cu $K$α |
| μ (mm$^{-1}$) | 0.76 |
| Crystal size (mm) | 0.17 × 0.13 × 0.08 |
| No. of measured, independent and observed [$I > 2\sigma(I)$] reflections | 19971, 3403, 2978 |
| $R_{int}$ | 0.038 |
| $(\sin \theta/\lambda)_{max}$ (Å$^{-1}$) | 0.629 |
| $R[F^2 > 2\sigma(F^2)]$, $wR(F^2)$, $S$ | 0.057, 0.199, 1.75 |
| No. of reflections | 3403 |
| No. of parameters | 300 |
| H-atom treatment | H-atom parameters constrained |
| $\Delta\rho_{max}$, $\Delta\rho_{min}$ (e Å$^{-3}$) | 0.44, −0.50 |

Computer programs: *CrysAlis PRO* 1.171.42.58a (Rigaku OD, 2022), *SHELXL2018*/3 (Sheldrick, 2018), *SHELXTL*.

## ORTEP Structures

**Fig. S1.** *ORTEP Structure of **L6AuCl** (50% ellipsoids).* (Crystallographic data has been deposited with the Cambridge Crystallographic Data Center as supplementary publication no. CCDC 2513141).

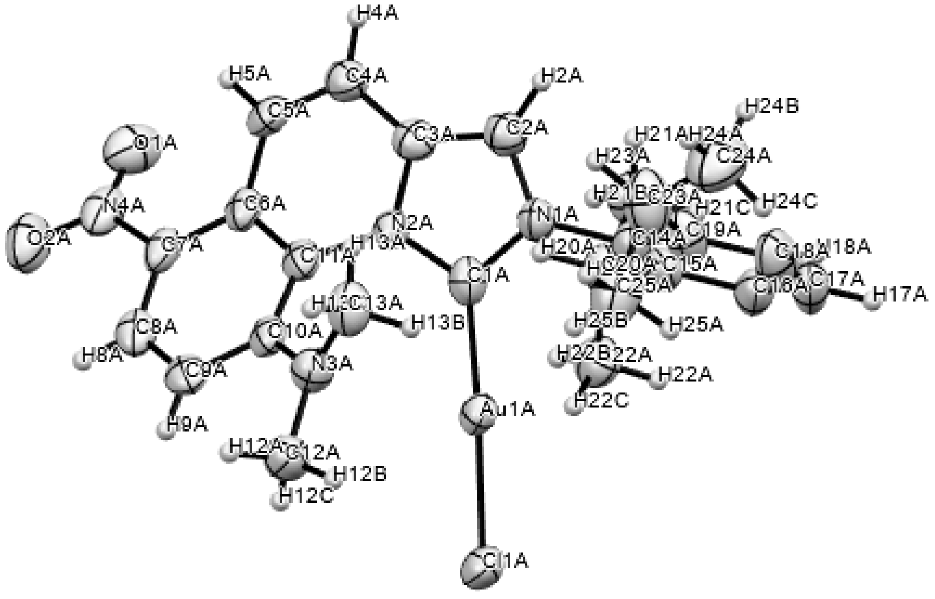


Crystals suitable for X-ray diffraction were obtained by slow diffusion of hexane into a dichloromethane solution of the compound at room temperature. The asymmetric unit contains two independent molecules of the complex, which exhibit very similar geometries. Only structure A is shown for clarity. Selected bond lengths (Å) and angles (deg), CCDC 2513141, **[L6AuCl]**: Au1A–C1A, 1.978(8); Au1A–Cl1A, 2.276(2); C1A–N1A, 1.37(1); C1A–N2A, 1.37(1); Au1A-N3A, 2.980; C10A-N3A, 1.38(9); Cl1A–Au1A–C1A, 176.1(2); N1A–C1A–N2A, 103.8(6).

**Fig. S2.** *ORTEP Structure of **4a** (50% ellipsoids).* (Crystallographic data has been deposited with the Cambridge Crystallographic Data Center as supplementary publication no. CCDC 2513140).

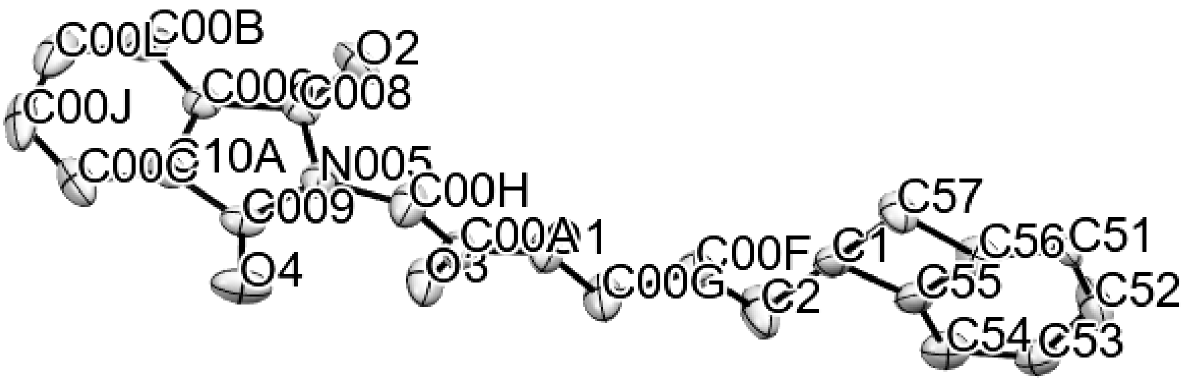


Crystals suitable for X-ray diffraction were obtained by slow diffusion of hexane into a dichloromethane solution of the compound at room temperature. The benzocyclobutene group is disordered over two orientations. Only the major component is shown in the crystallographic figures for clarity. Selected bond lengths (Å) and angles (deg), CCDC 2513140, **4a**: C1–C2, 1.398(4); C1-C57, 1.557(9); C1–C55, 1.51(1); C55–C56, 1.38(2); C57–C1–C55, 86.7(5); C1–C57–C56, 86.7(8); C1–C55–C56, 97.9(8); C57–C56–C55, 89(1).

## NMR Spectra

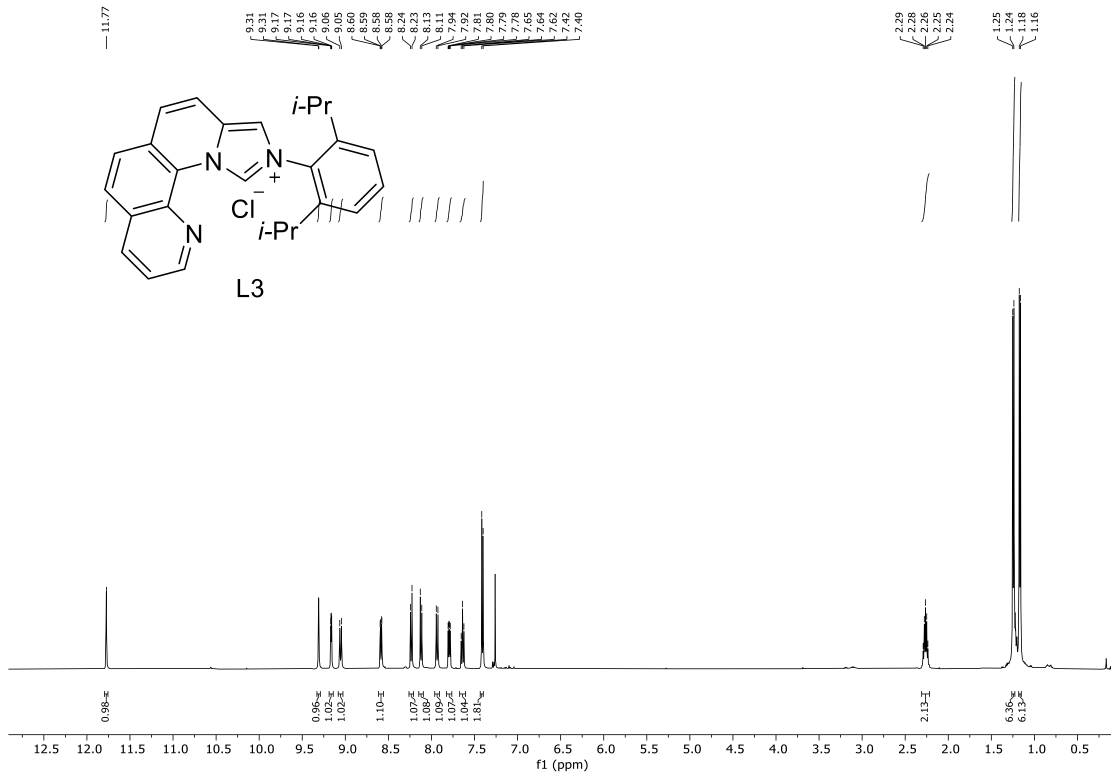


$^{1}H$ NMR (500 MHz, $CDCl_3$) Spectrum of **L3**

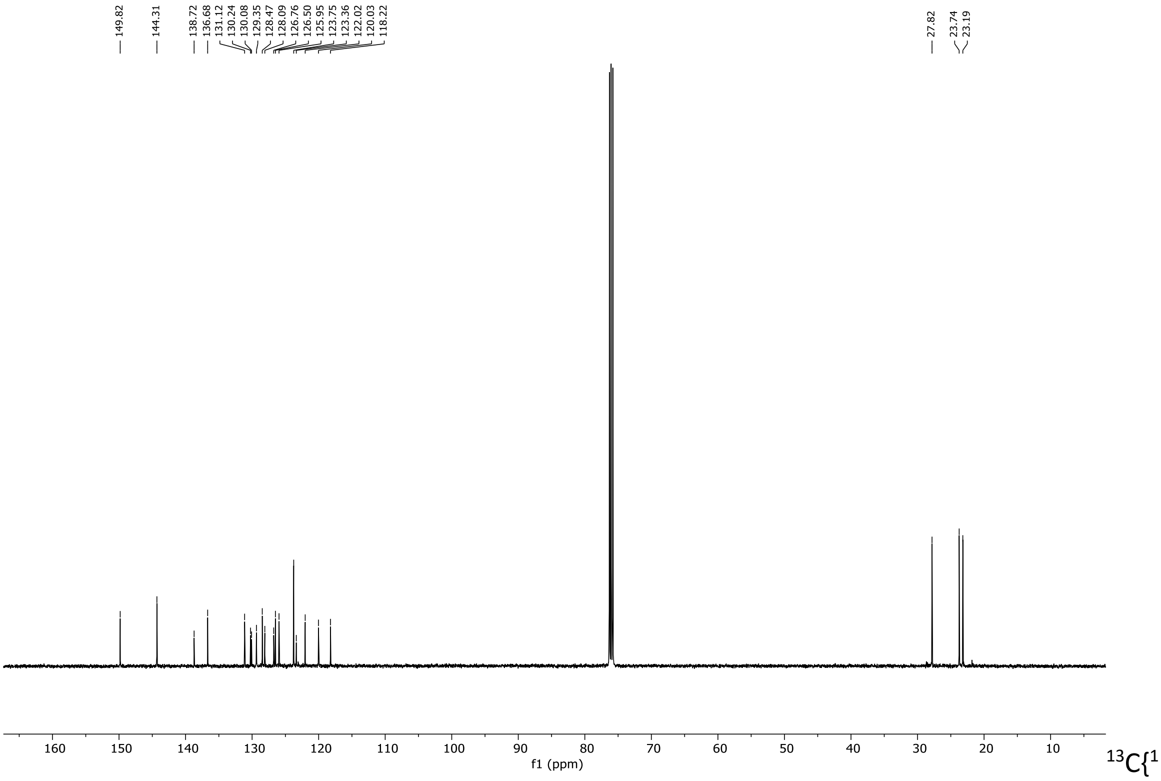


$^{13}C\{^{1}$

$^{13}C\{^{1}H\}$ NMR (125 MHz, $CDCl_3$) Spectrum of **L3**

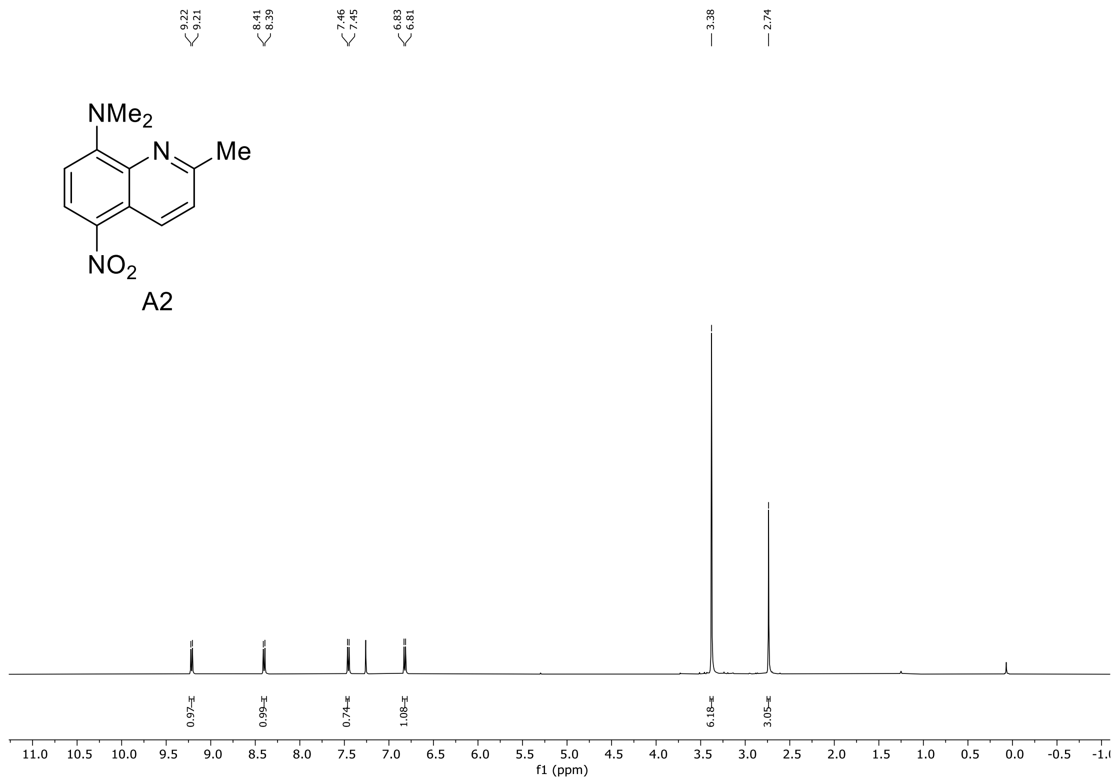


$^{1}$H NMR (500 MHz, $CDCl_3$) Spectrum of **A2**

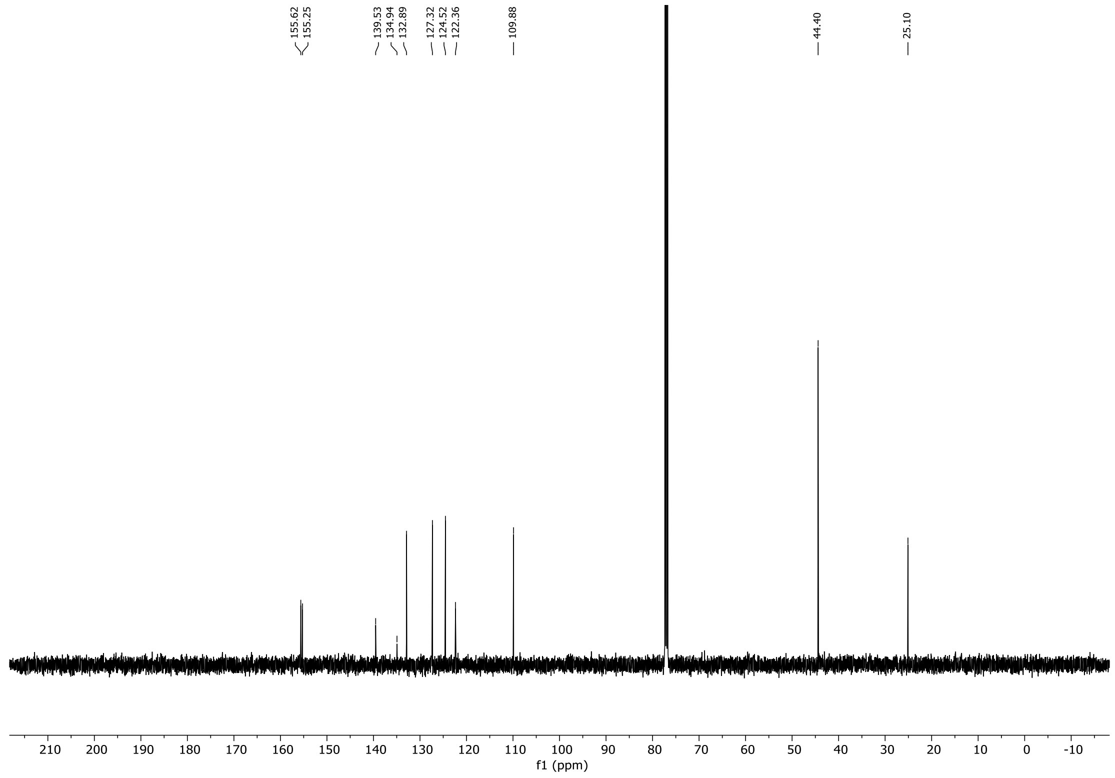


$^{13}$C{$^{1}$H} NMR (125 MHz, $CDCl_3$) Spectrum of **A2**

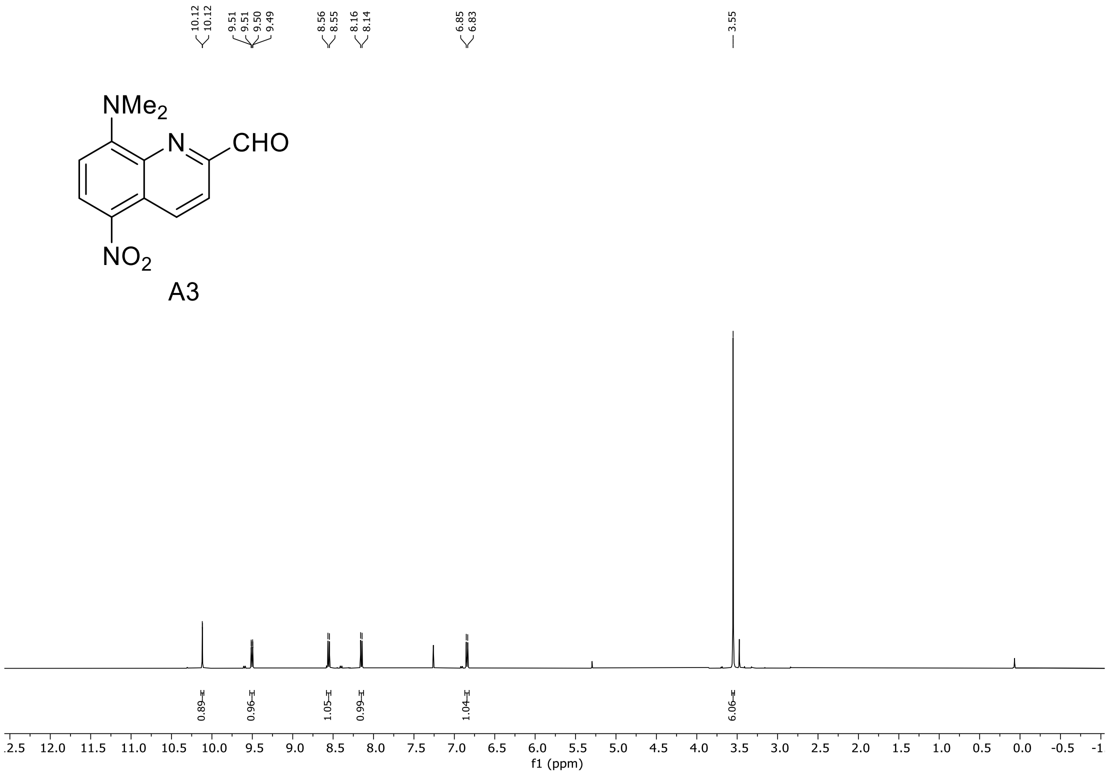


$^{1}$H NMR (500 MHz, $CDCl_3$) Spectrum of **A3**

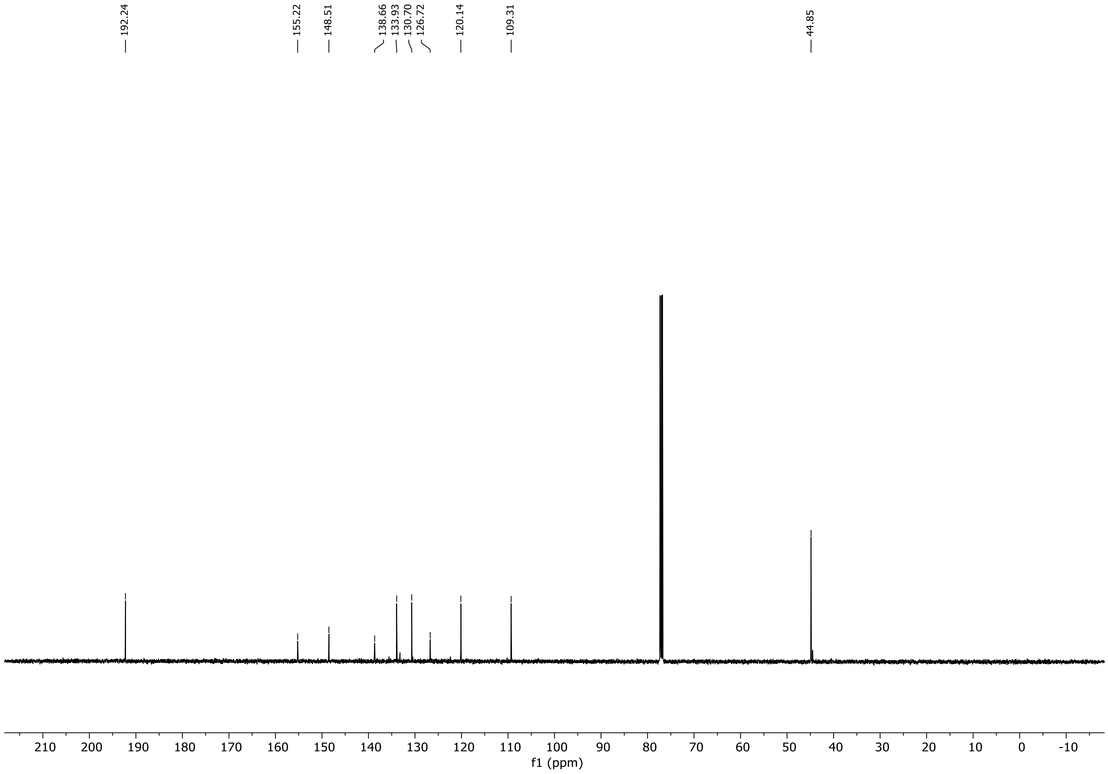


$^{13}C\{^{1}H\}$ NMR (125 MHz, $CDCl_3$) Spectrum of **A3**

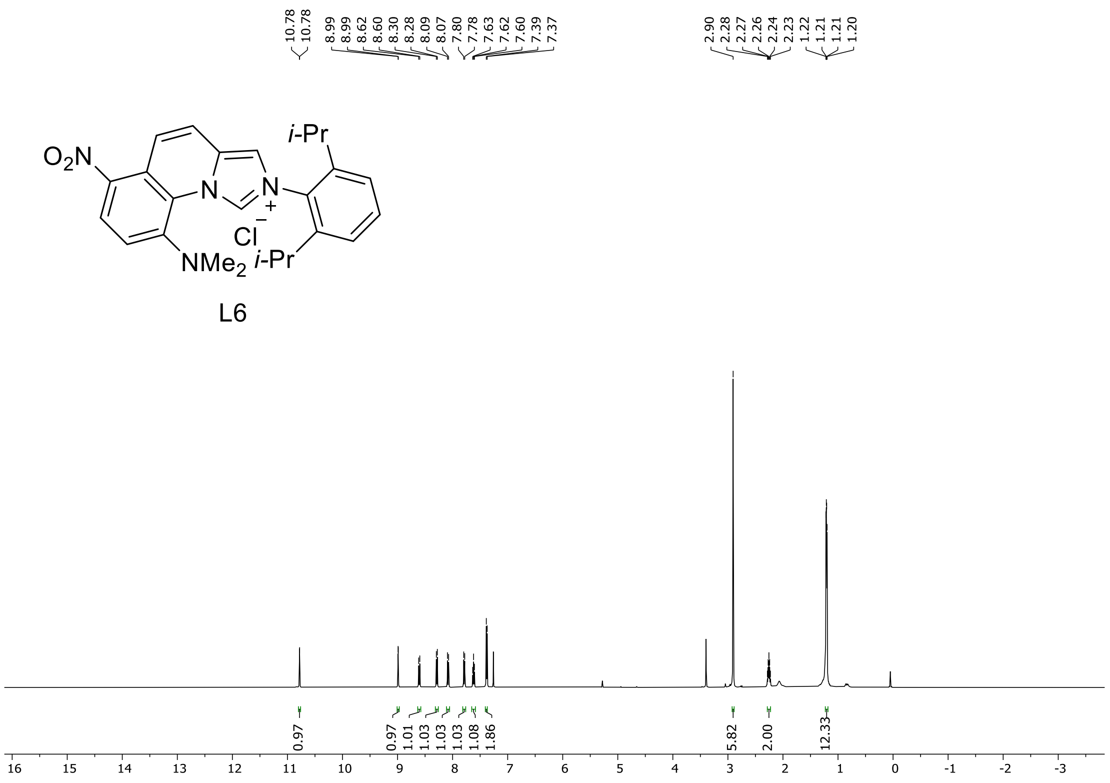


$^{1}$H NMR (500 MHz, $CDCl_3$) Spectrum of **L6**

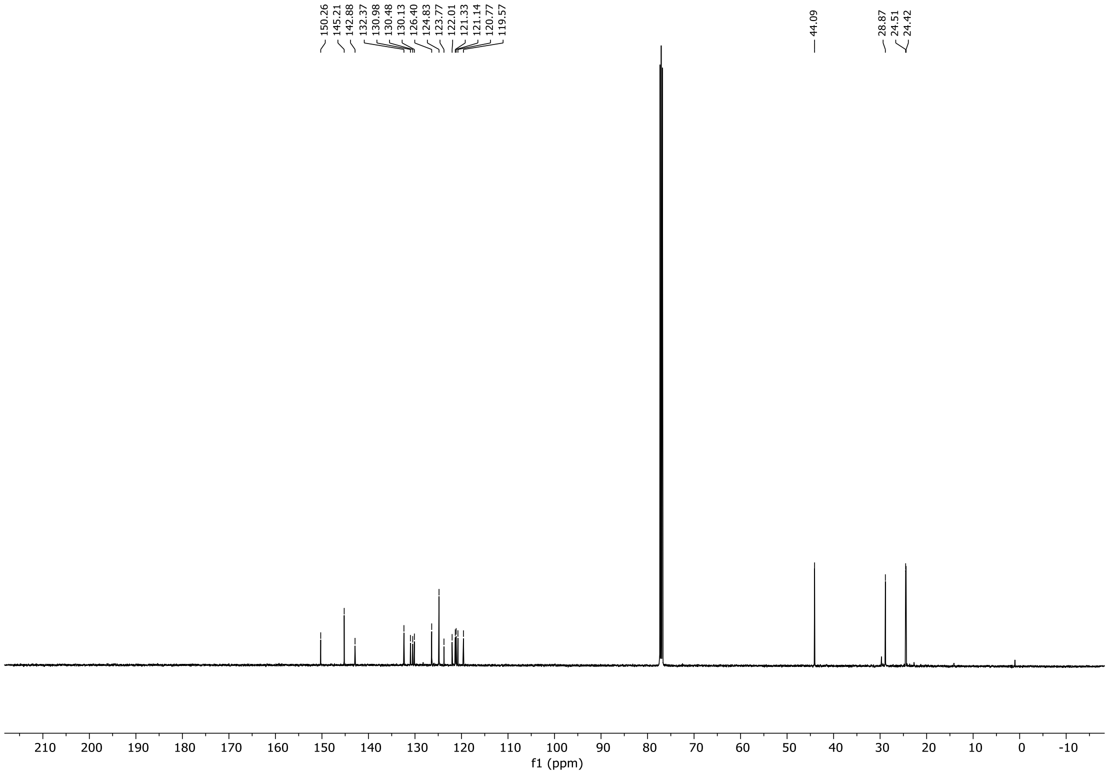


$^{13}C\{^{1}H\}$ NMR (125 MHz, $CDCl_3$) Spectrum of **L6**

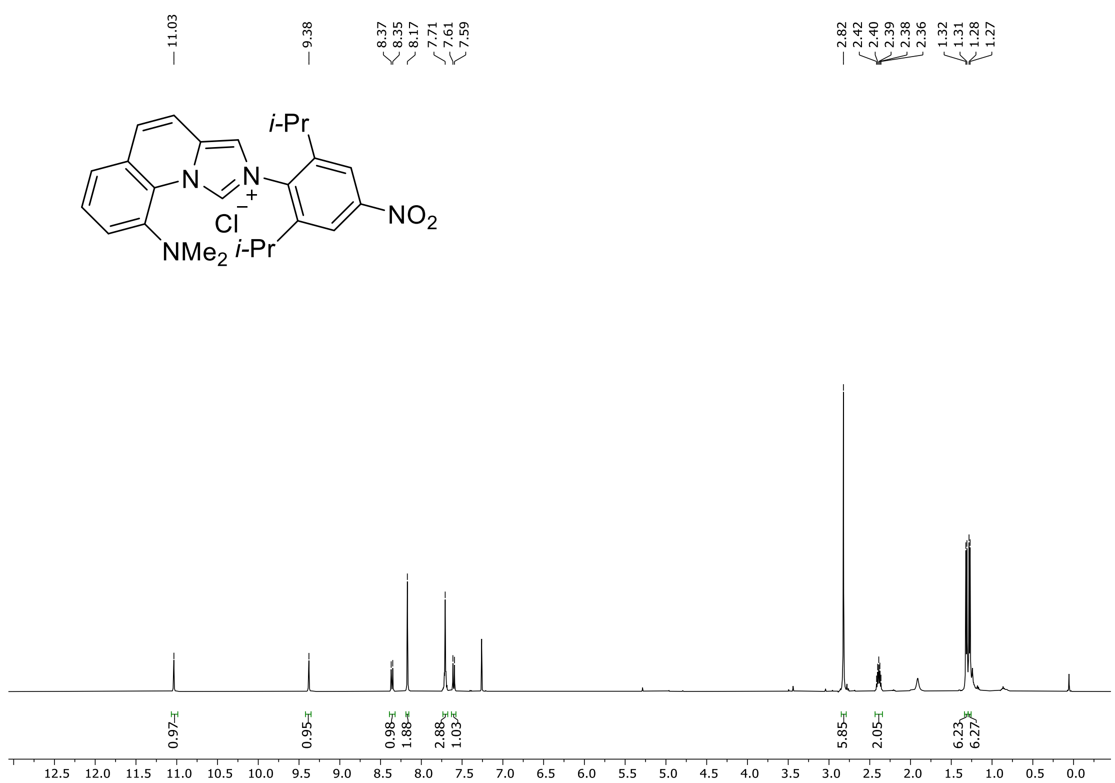


$^{1}$H NMR (500 MHz, $CDCl_3$) Spectrum of **L7**

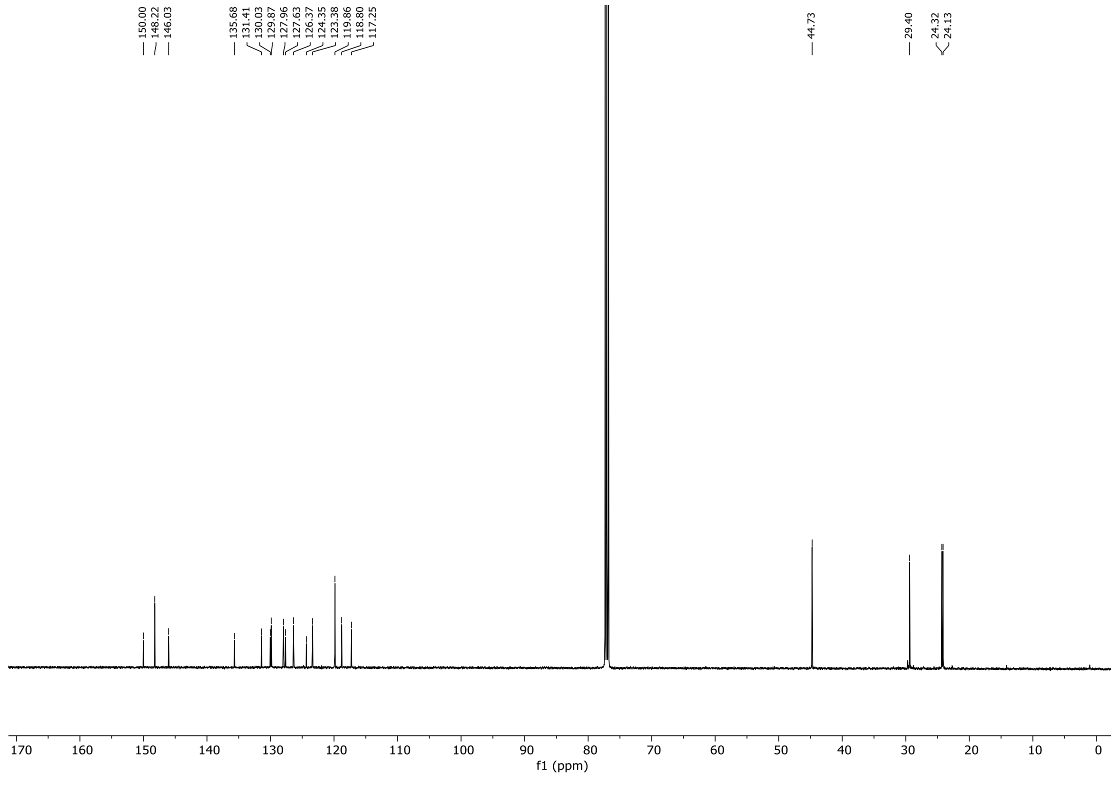


$^{13}$C{$^{1}$H} NMR (125 MHz, $CDCl_3$) Spectrum of **L7**

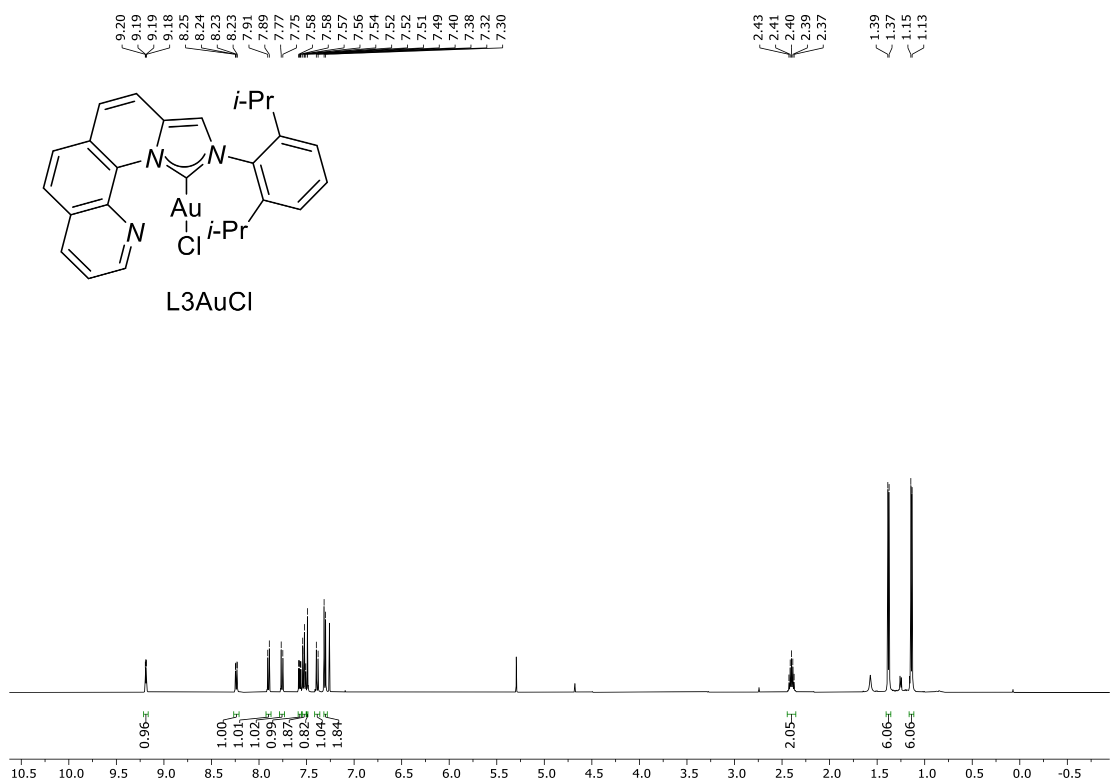


$^{1}H$ NMR (500 MHz, $CDCl_3$) Spectrum of **[L3AuCl]**

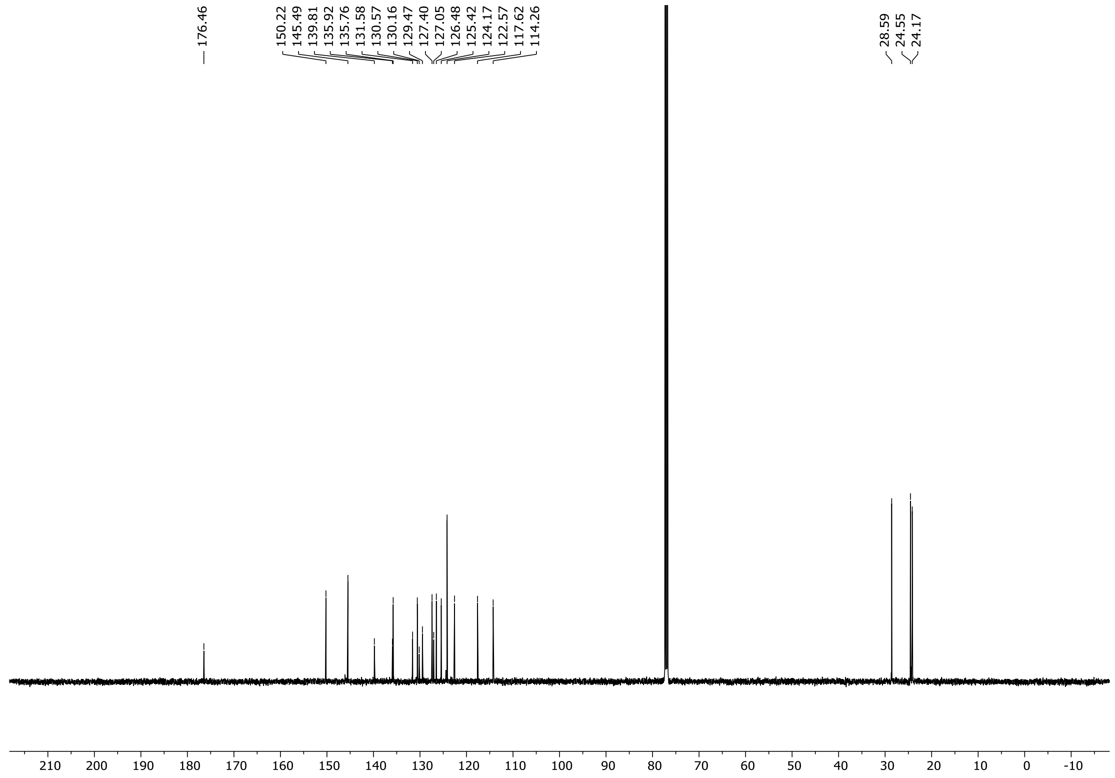


$^{13}C\{^{1}H\}$ NMR (125 MHz, $CDCl_3$) Spectrum of **[L3AuCl]**

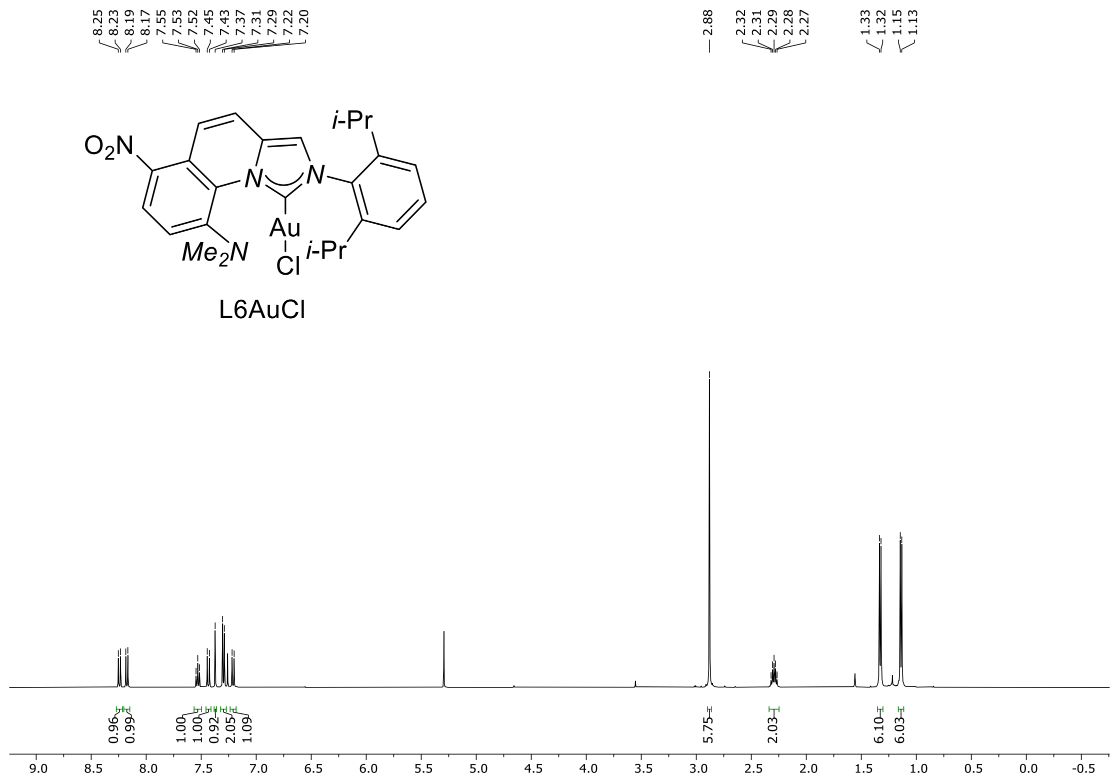

$^{1}$H NMR (500 MHz, $CDCl_3$) Spectrum of **[L6AuCl]**

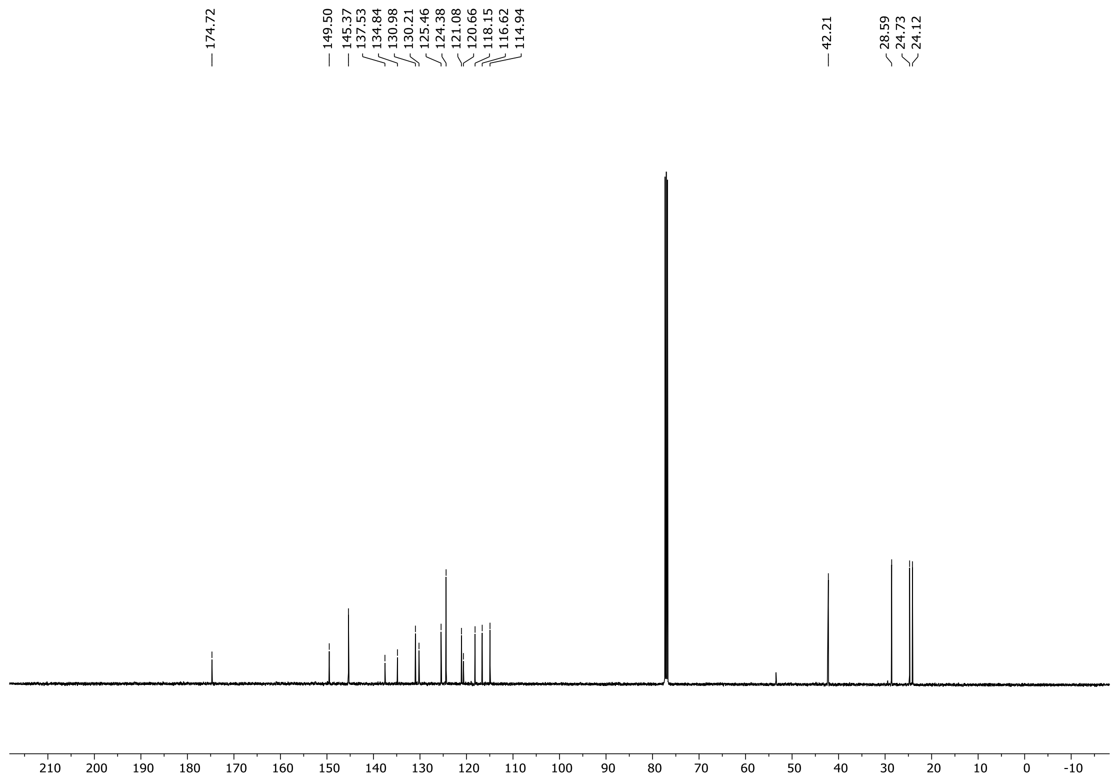

$^{13}$C{[1] H} NMR (125 MHz, $CDCl_3$) Spectrum of **[L6AuCl]**

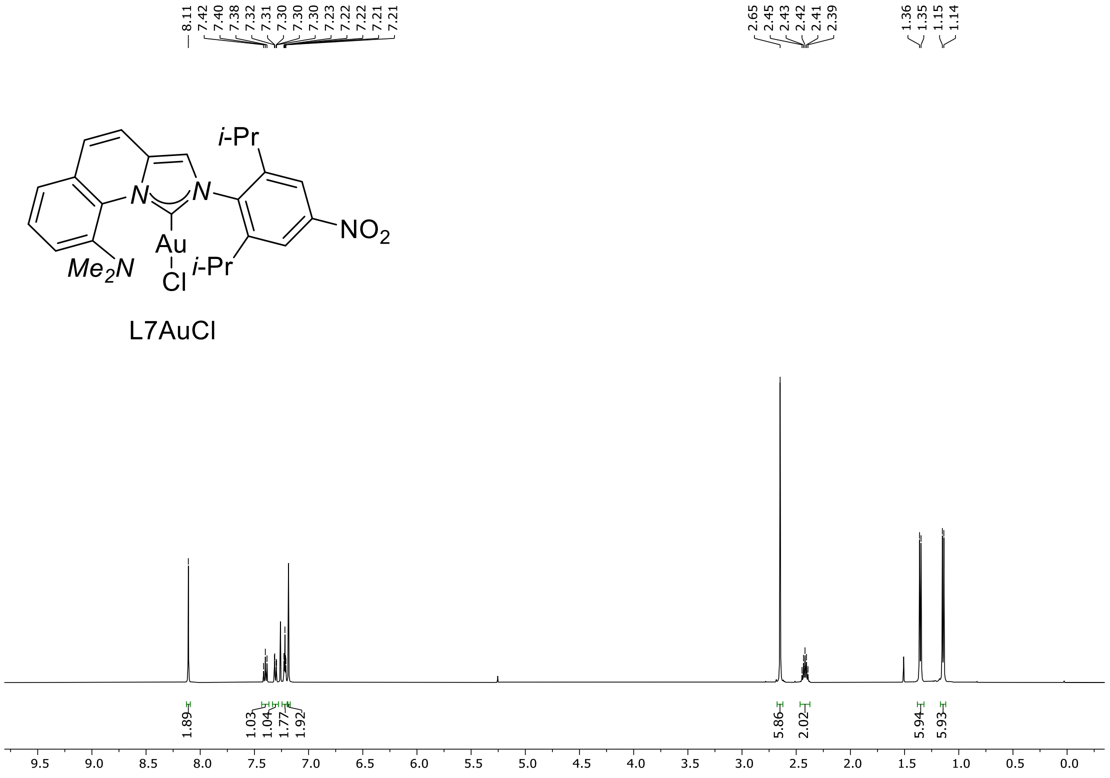


$^1$H NMR (500 MHz, $CDCl_3$) Spectrum of **[L7AuCl]**

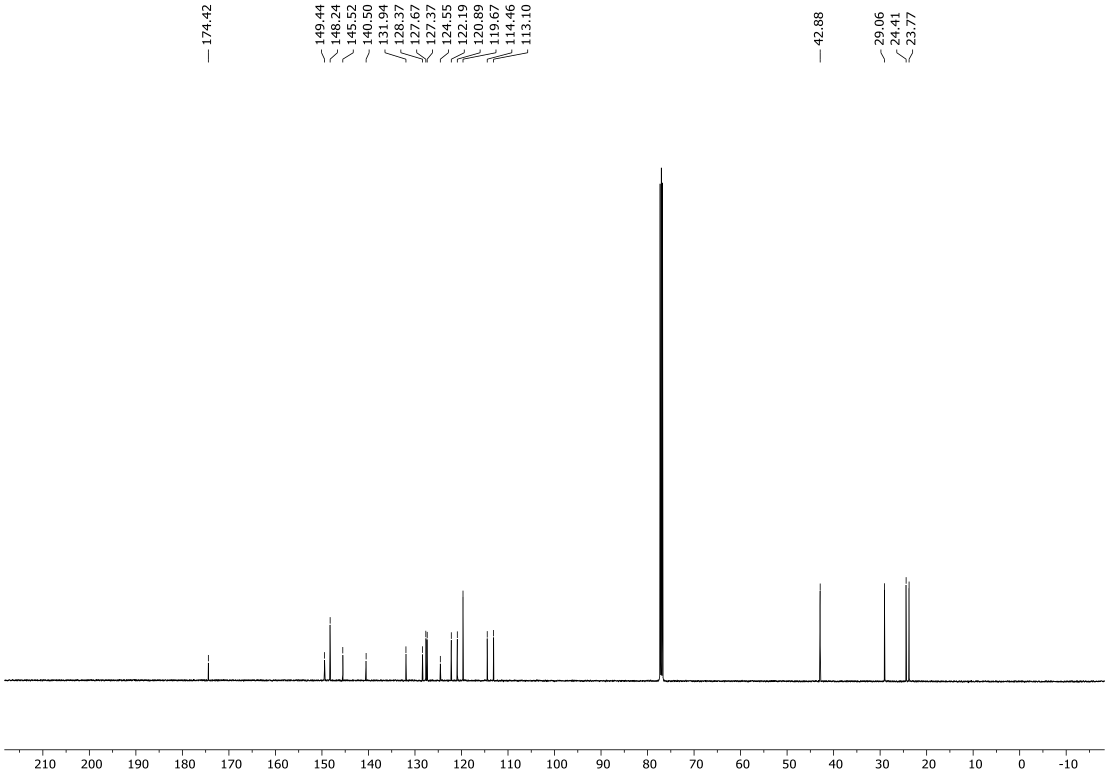


$^{13}C\{^1H\}$ NMR (125 MHz, $CDCl_3$) Spectrum of **[L7AuCl]**

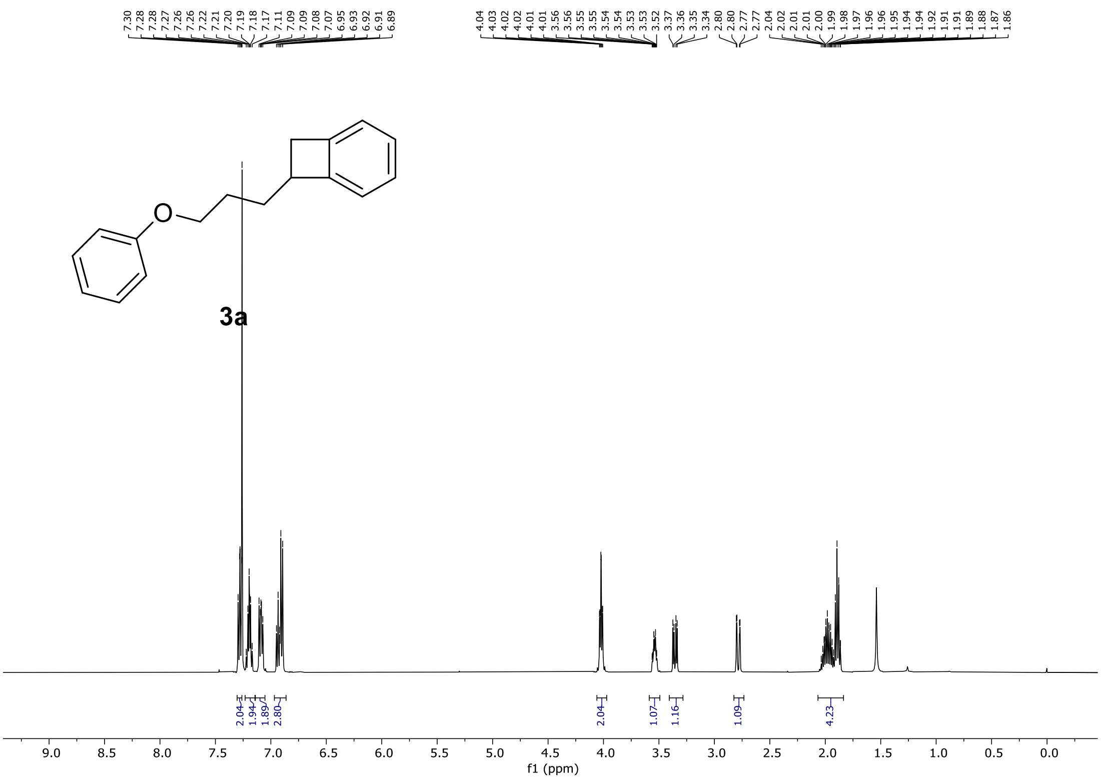

$^{1}H$ NMR (500 MHz, $CDCl_3$) Spectrum of **3a**

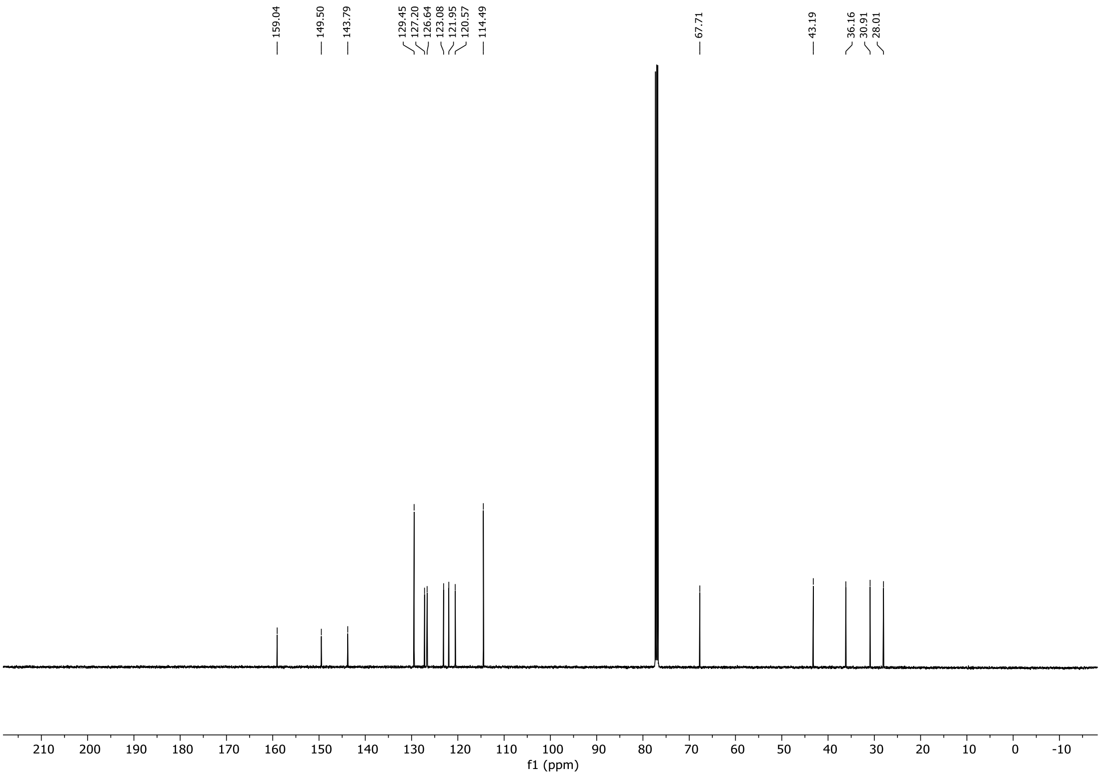

$^{13}C\{^{1}H\}$ NMR (125 MHz, $CDCl_3$) Spectrum of **3a**

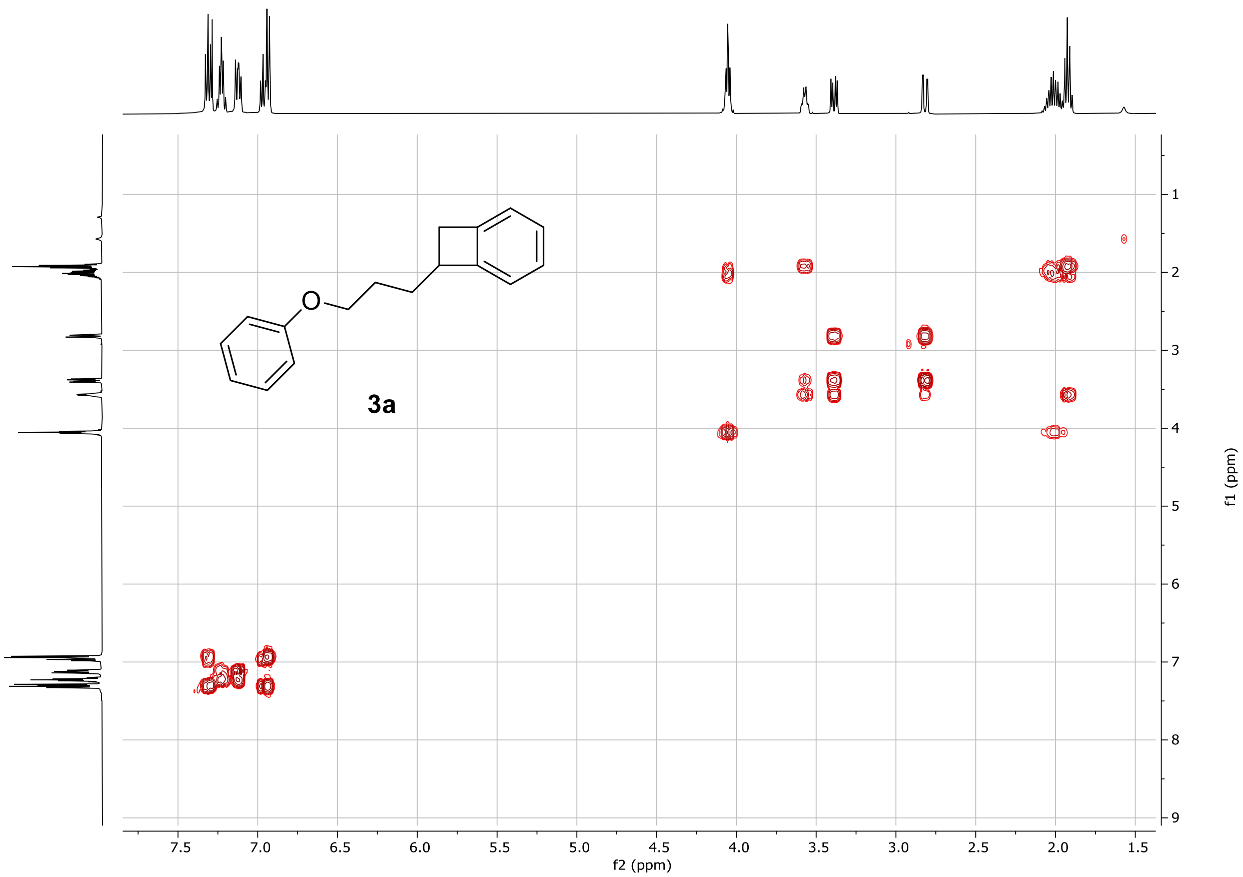


$^{1}H$–$^{1}H$ COSY (500 MHz, $CDCl_3$) Spectrum of **3a**

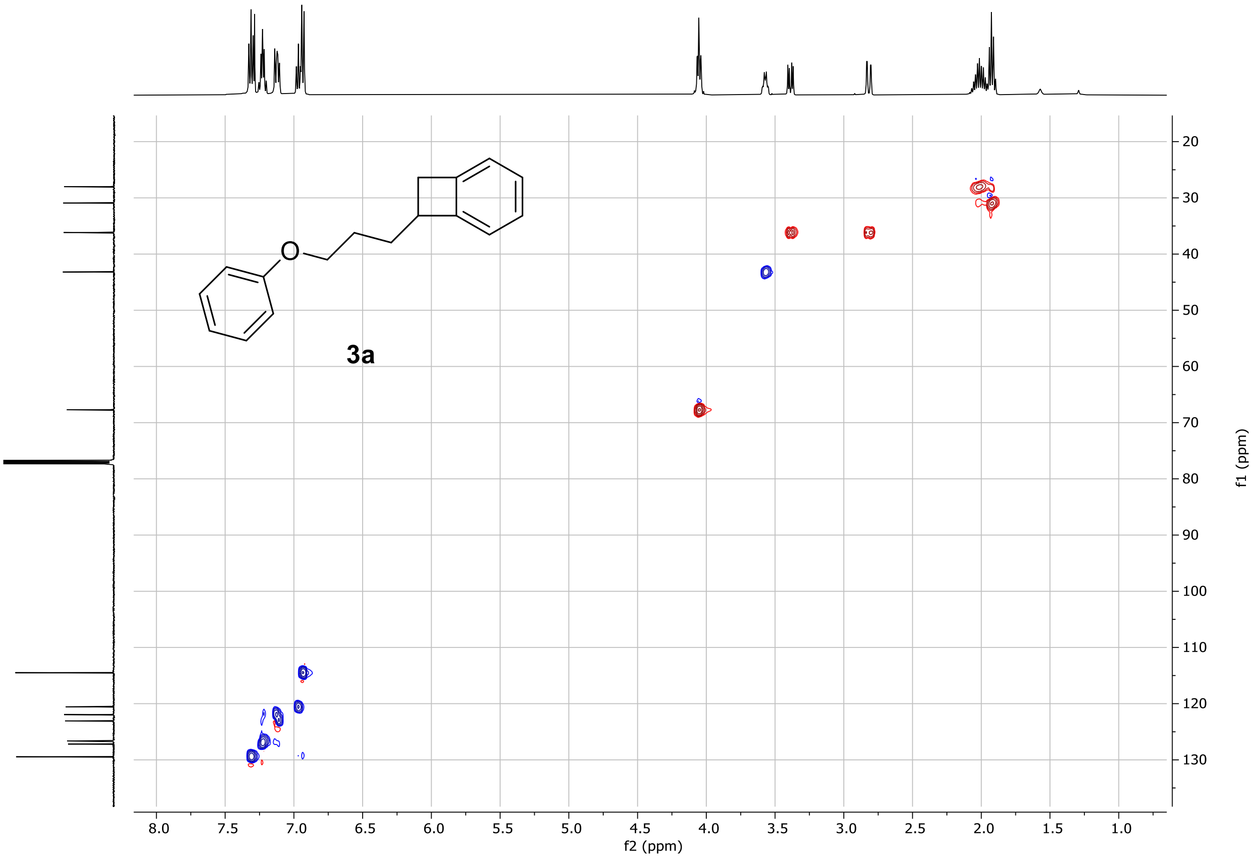


$^1$H–$^{13}$C HSQC (500/125 MHz, $CDCl_3$) Spectrum of **3a**

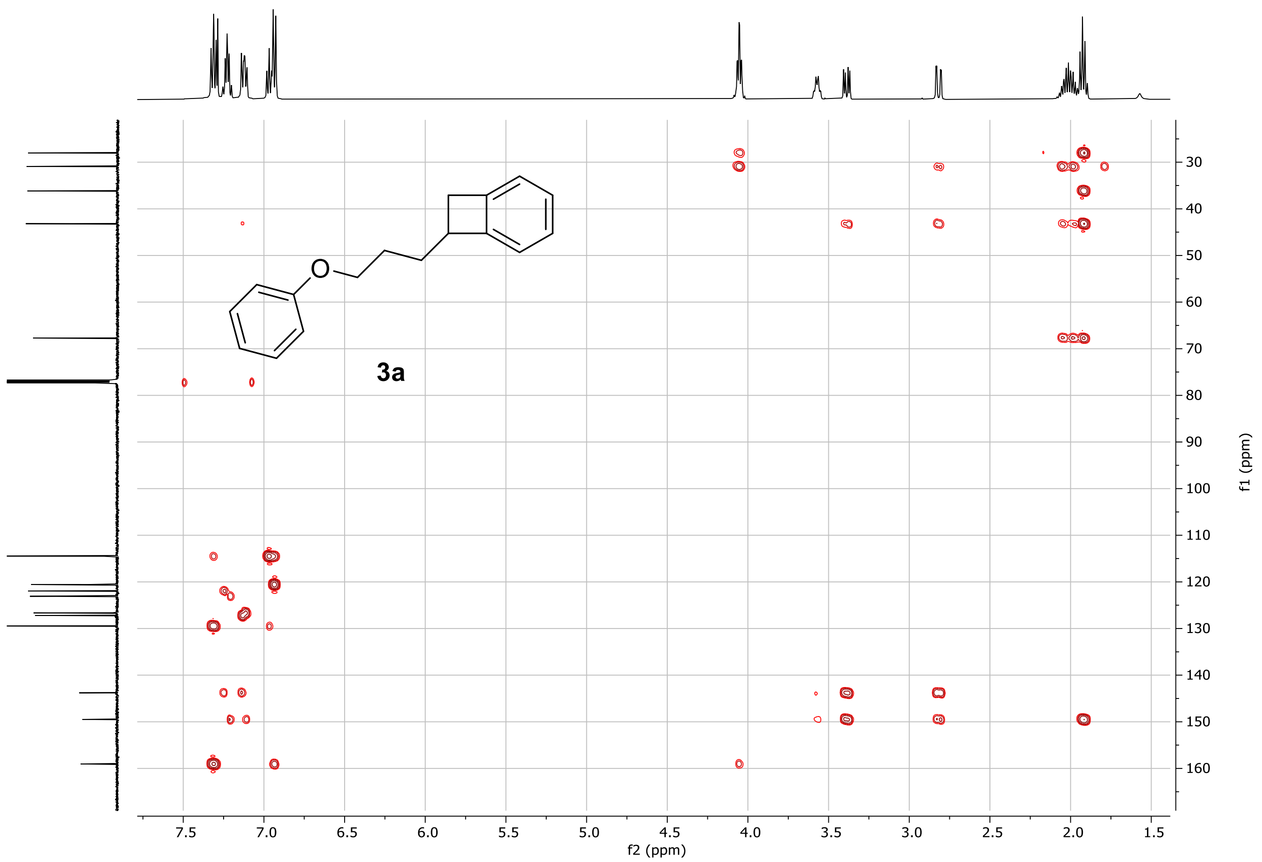


$^{1}H$–$^{13}C$ HMBC (500/125 MHz, $CDCl_3$) Spectrum of **3a**

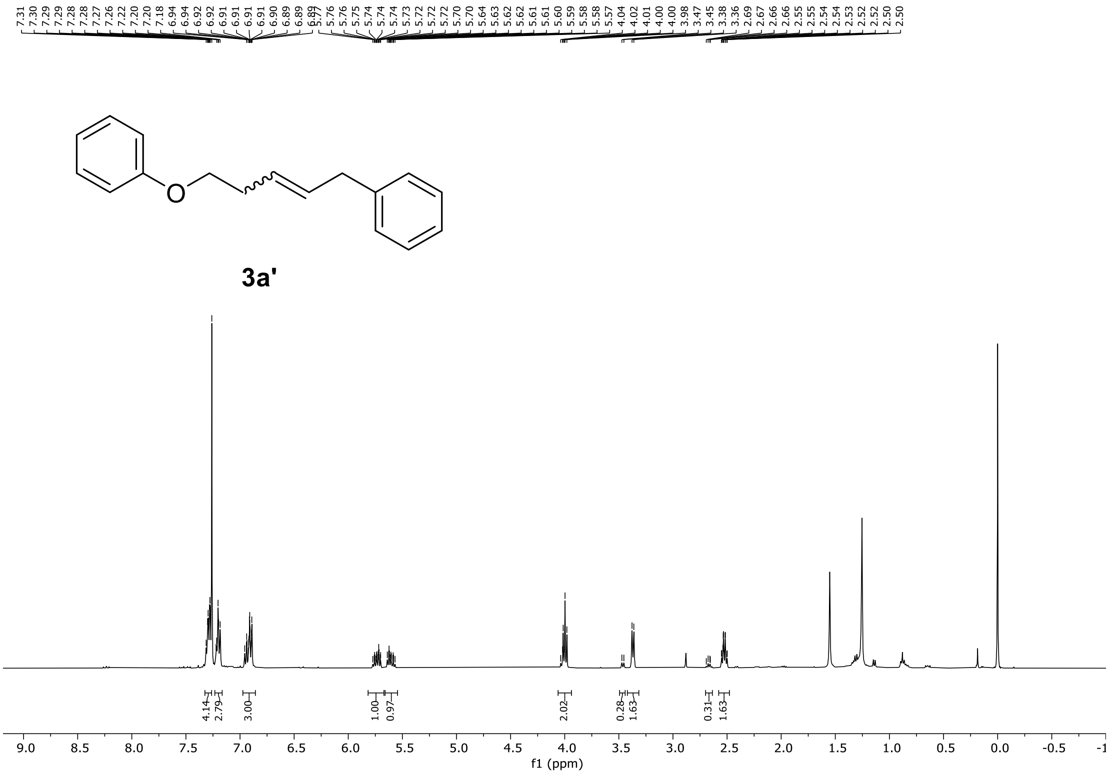


$^1$H NMR (400 MHz, $CDCl_3$) Spectrum of **3a’**

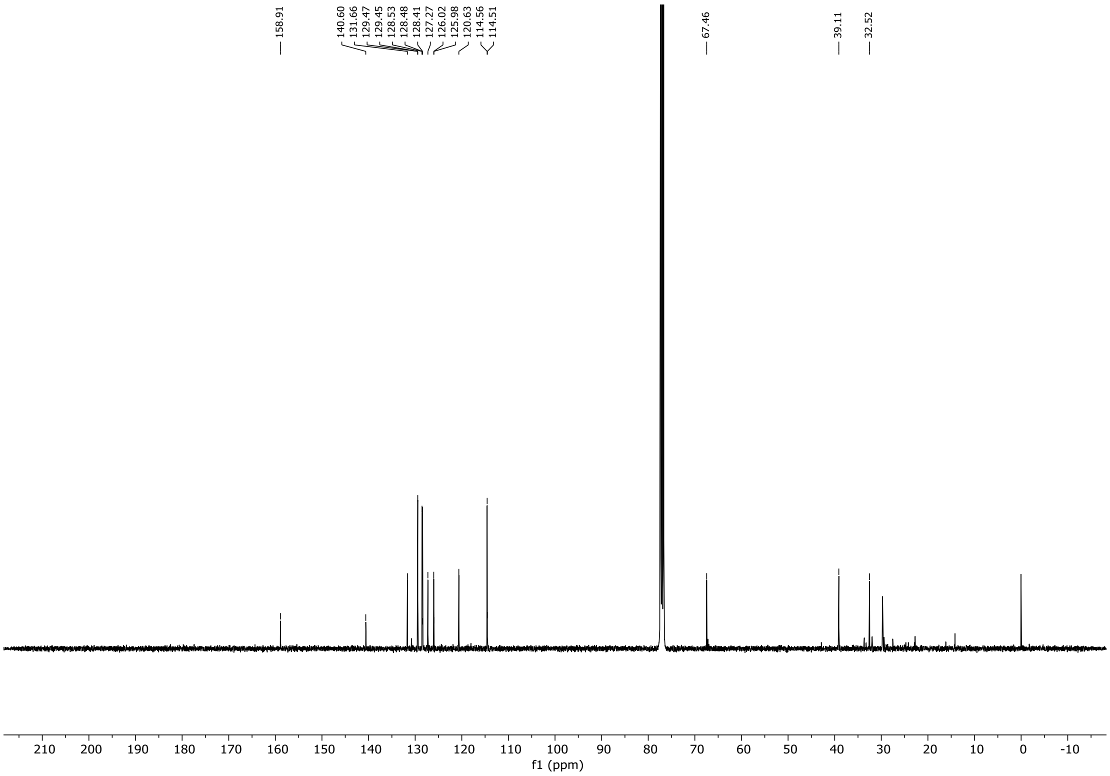


$^{13}C\{^1H\}$ NMR (101 MHz, $CDCl_3$) Spectrum of **3a’**

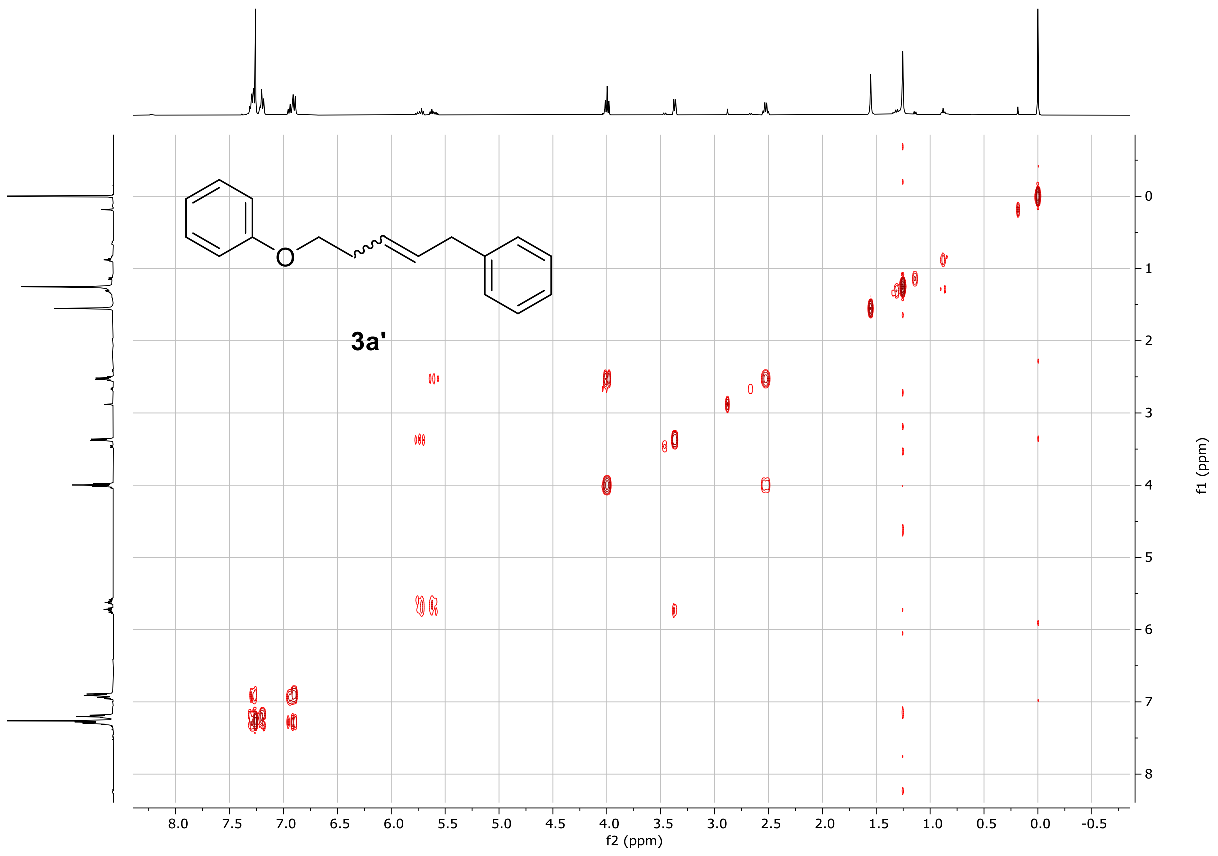


$^{1}H$–$^{1}H$ COSY (400 MHz, $CDCl_3$) Spectrum of **3a’**

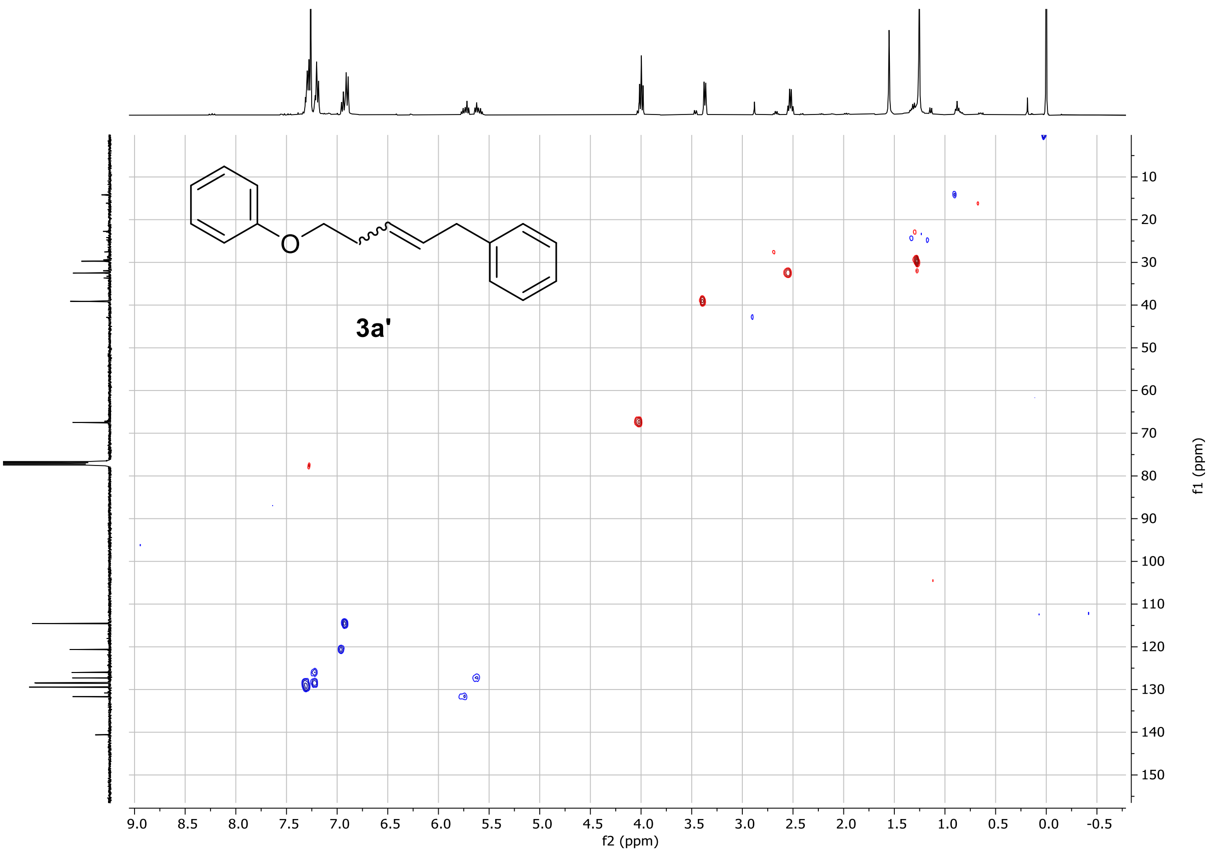


$^{1}$H–$^{13}$C HSQC (400/101 MHz, $CDCl_3$) Spectrum of **3a’**

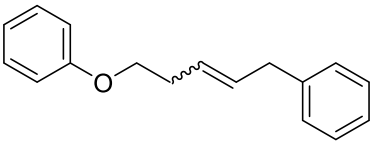

**3a'**

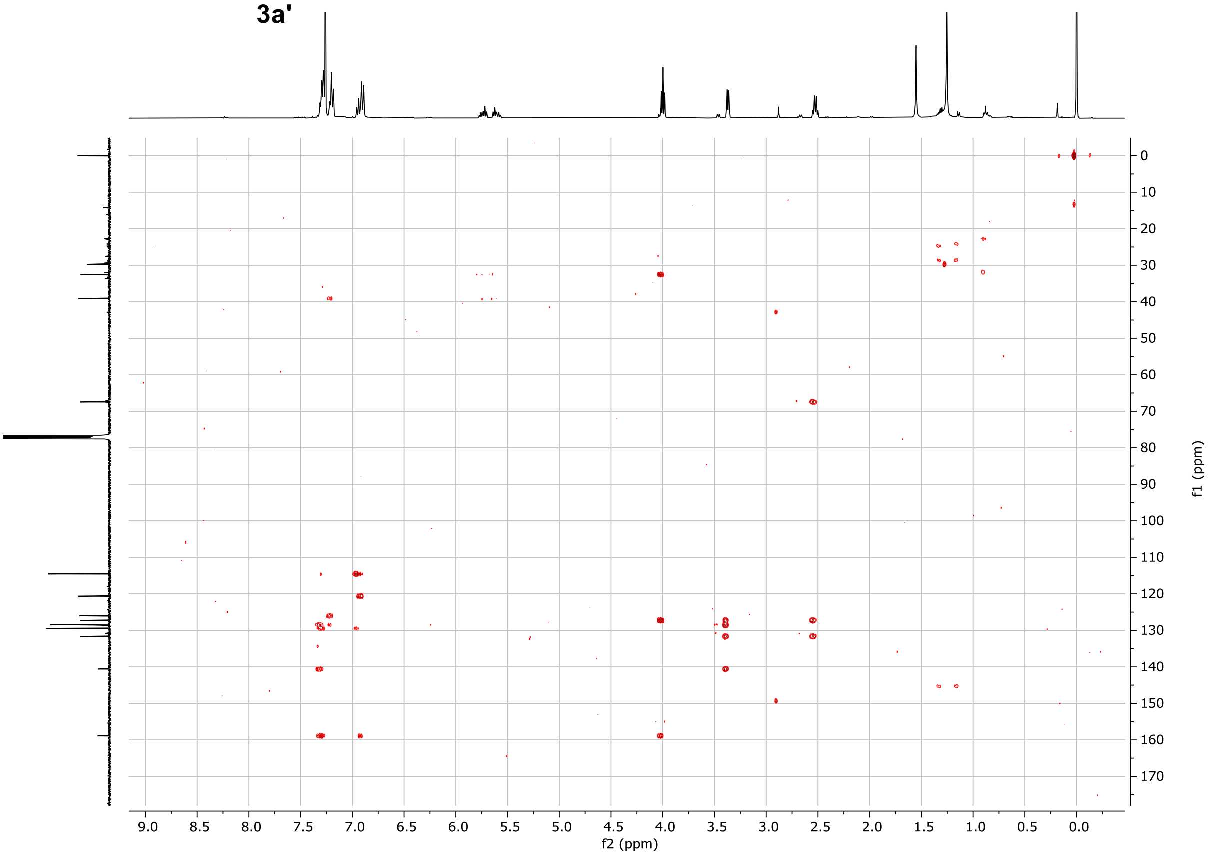


$^{1}$H–$^{13}$C HMBC (400/101 MHz, $CDCl_3$) Spectrum of **3a’**

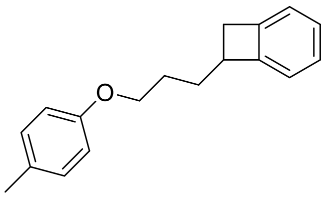

**3b**

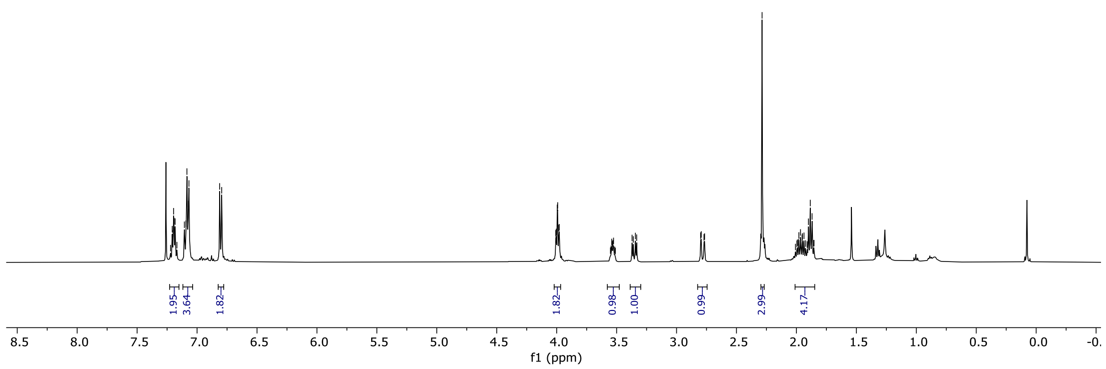


$^{1}$H NMR (500 MHz, $CDCl_3$) Spectrum of **3b**

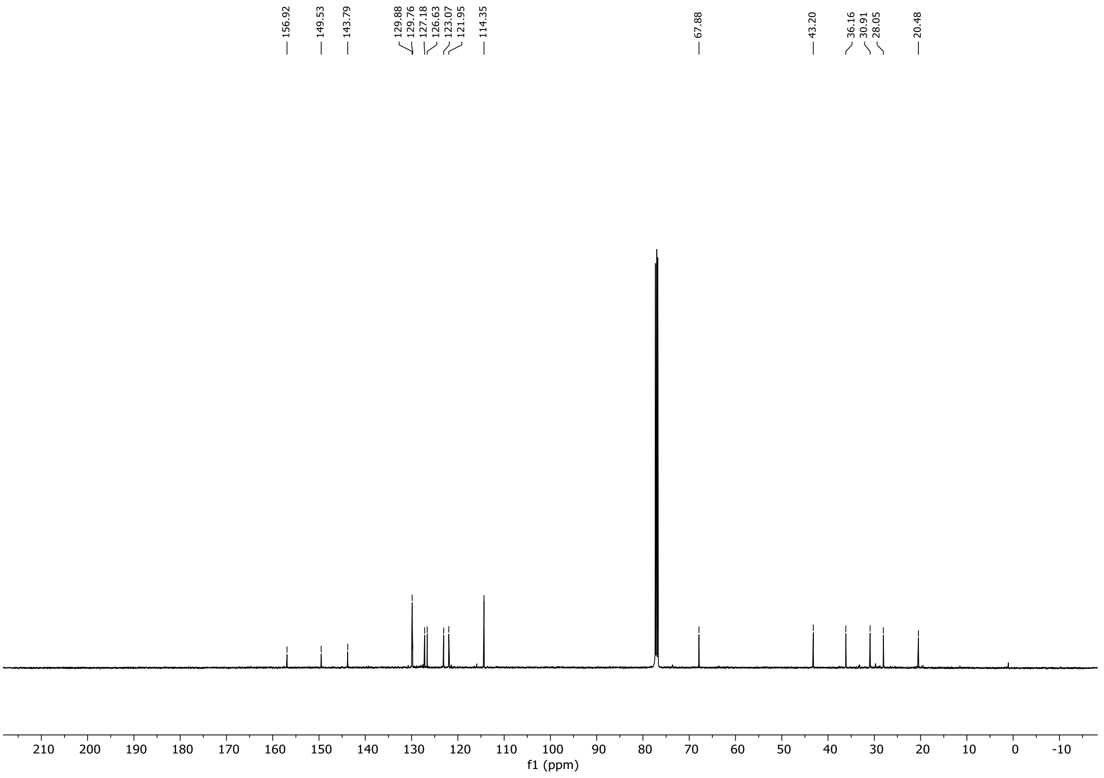


$^{13}C\{^{1}H\}$ NMR (125 MHz, $CDCl_3$) Spectrum of **3b**

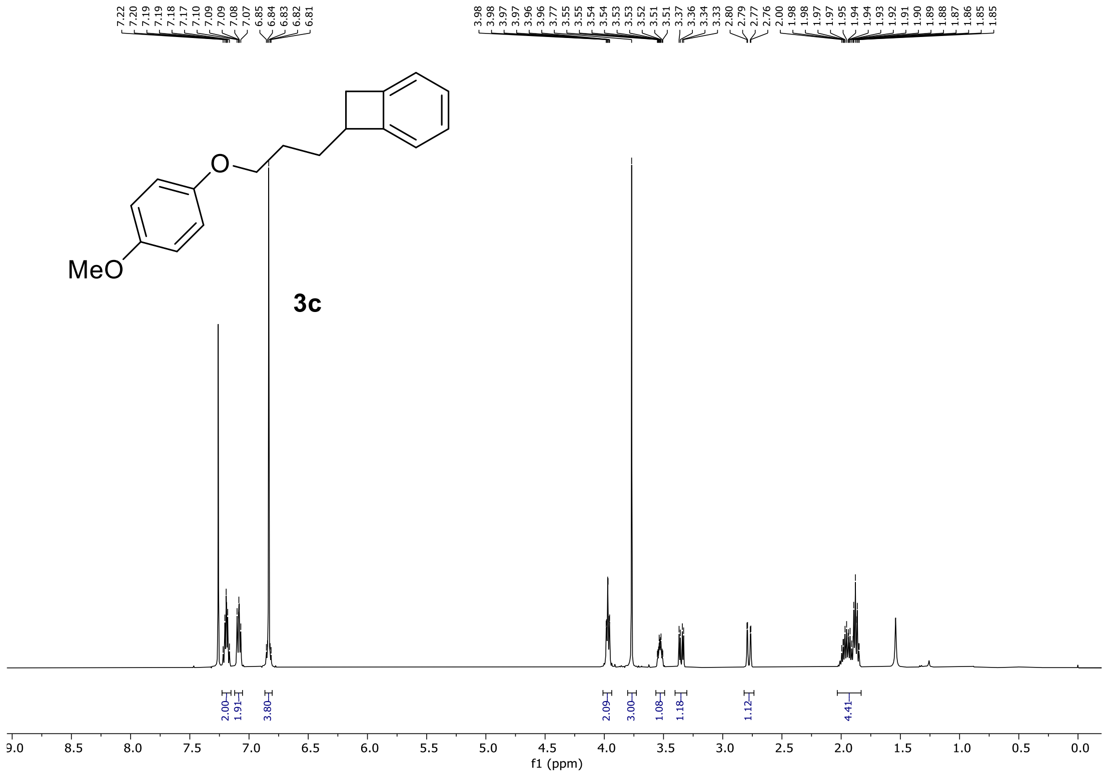


$^{1}H$ NMR (500 MHz, $CDCl_3$) Spectrum of **3c**

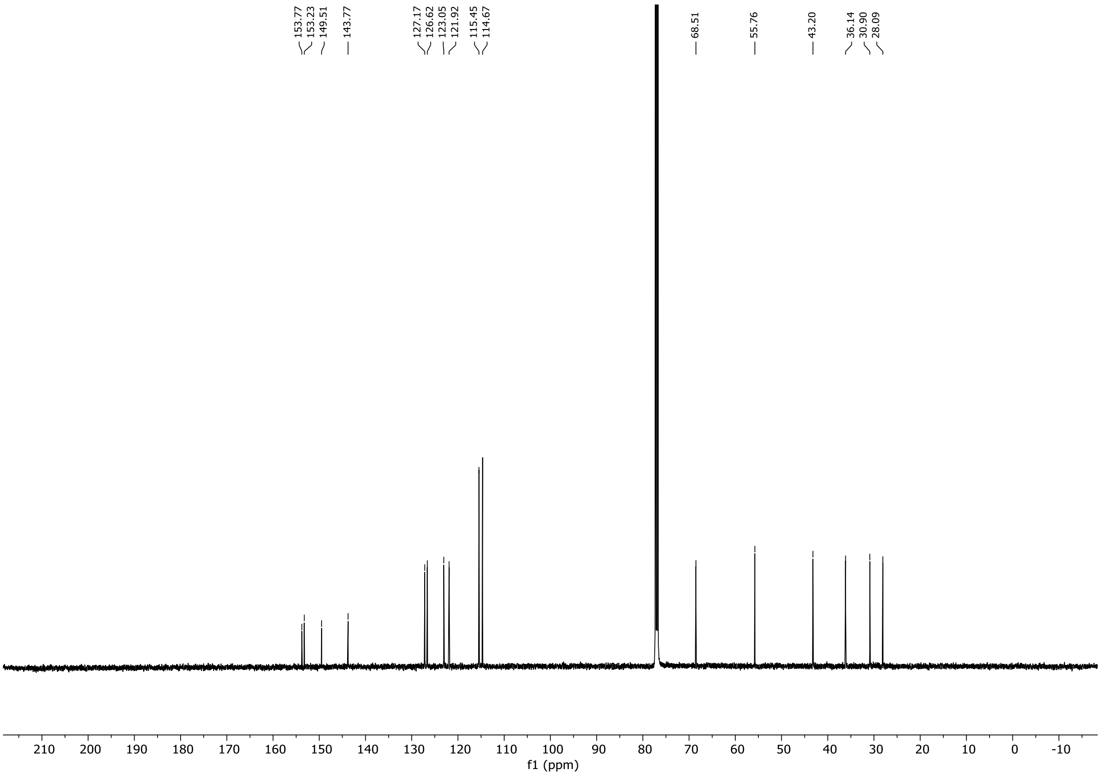


$^{13}C\{^{1}H\}$ NMR (125 MHz, $CDCl_3$) Spectrum of **3c**

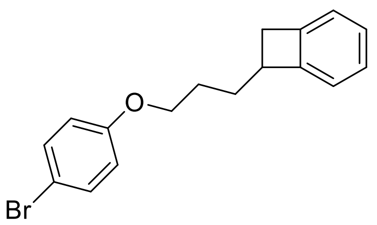


**3d**

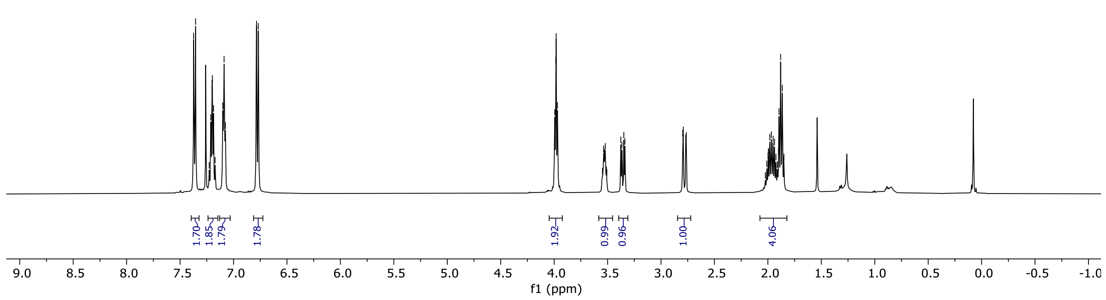


$^{1}$H NMR (500 MHz, $CDCl_3$) Spectrum of **3d**

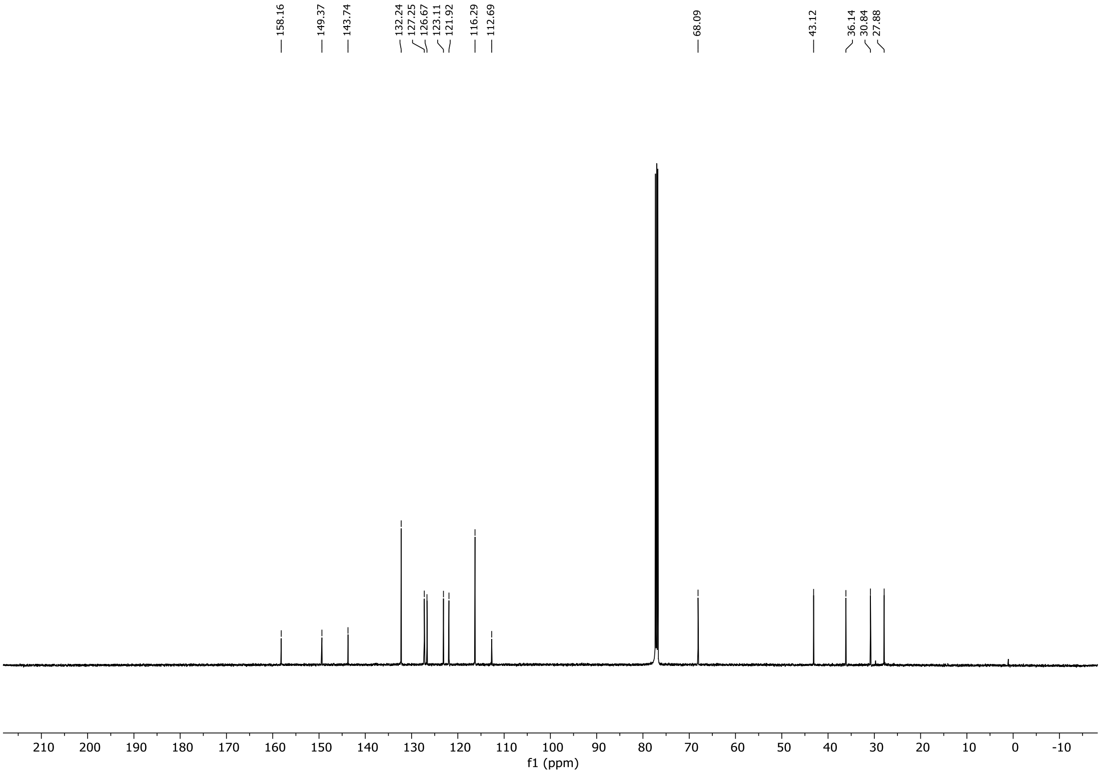


$^{13}C\{^{1}H\}$ NMR (125 MHz, $CDCl_3$) Spectrum of **3d**

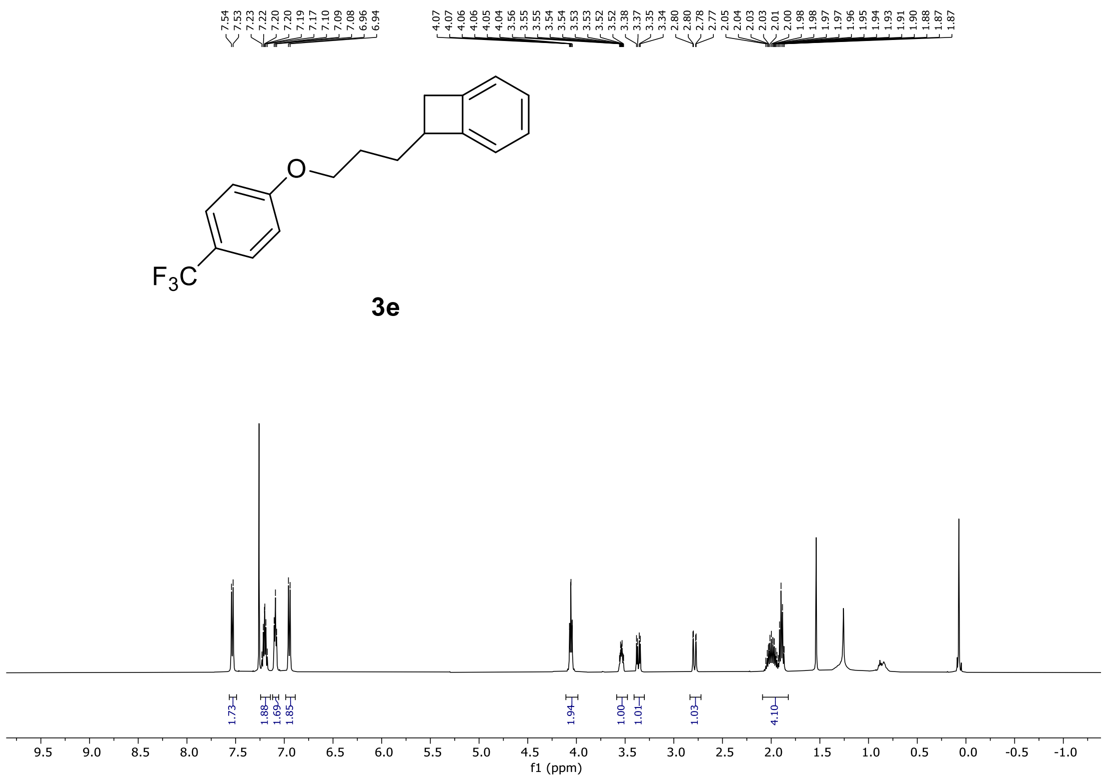


$^{1}$H NMR (500 MHz, $CDCl_3$) Spectrum of **3e**

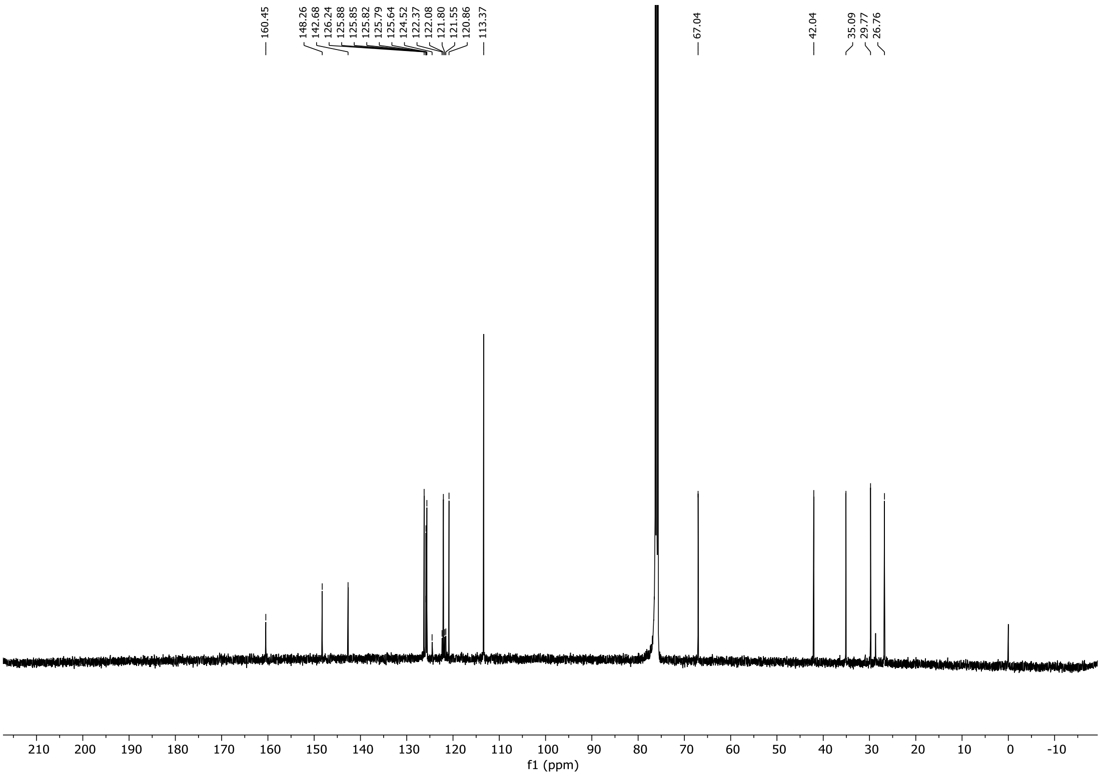


$^{13}$C{[1] H} NMR (125 MHz, $CDCl_3$) Spectrum of **3e**

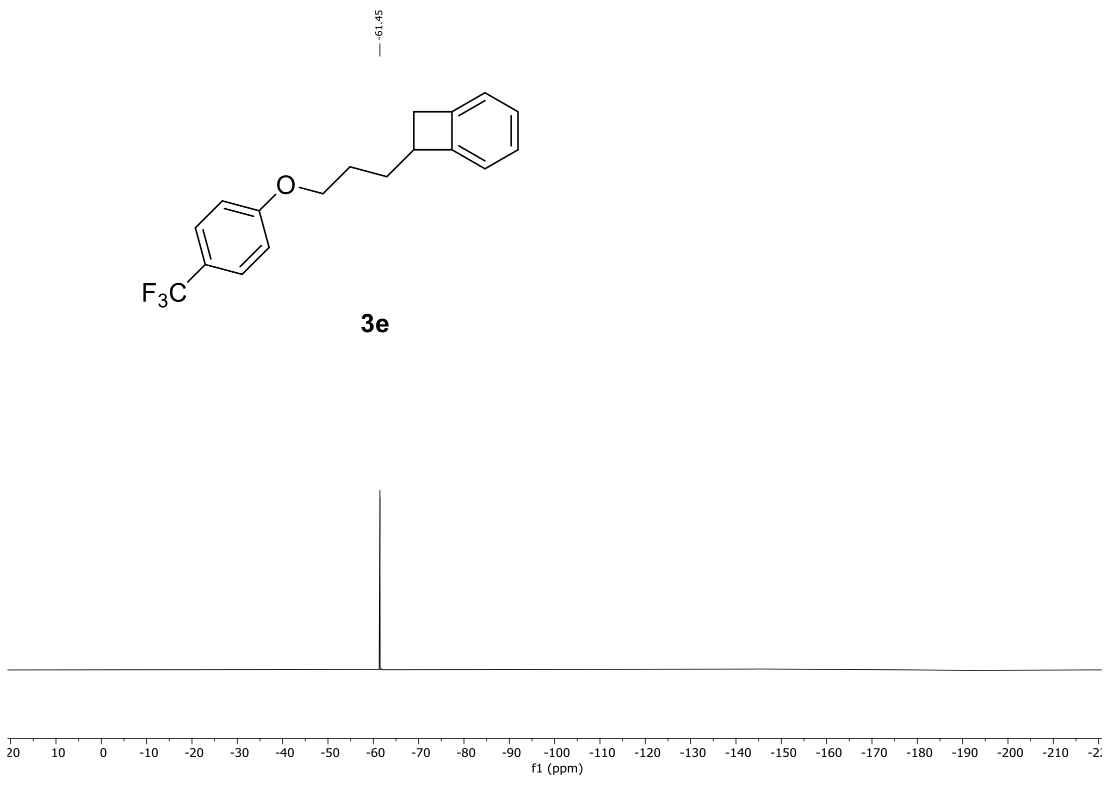


$^{19}F$ NMR (471 MHz, $CDCl_3$) Spectrum of **3e**

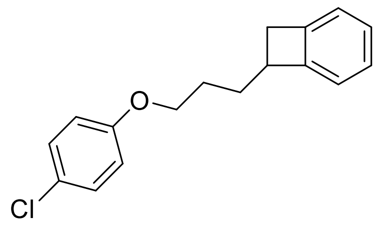

3f

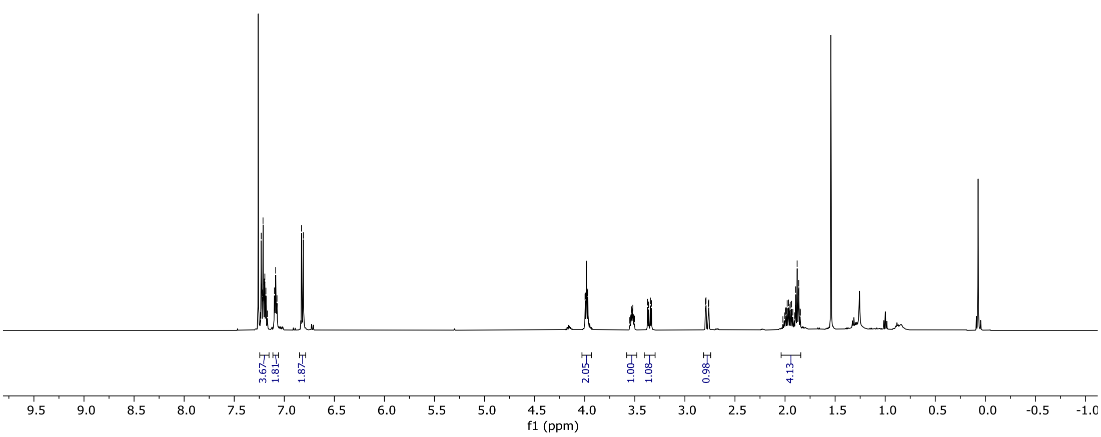


$^{1}$H NMR (500 MHz, $CDCl_3$) Spectrum of **3f**

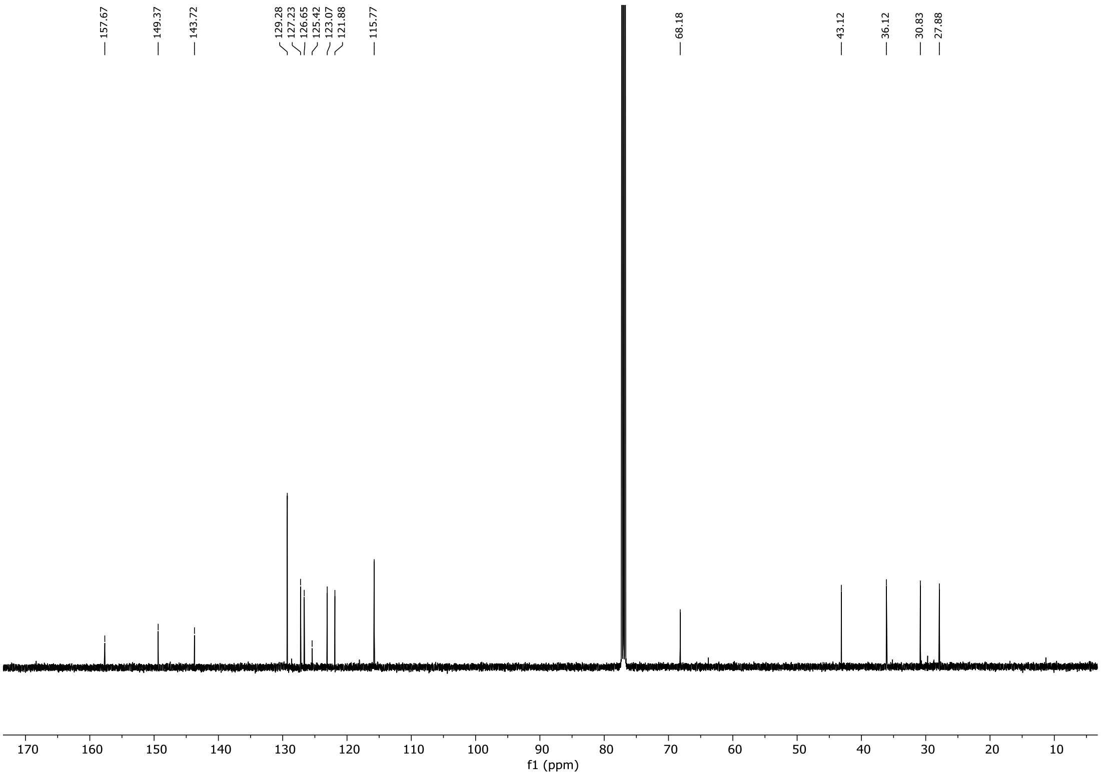


$^{13}C\{^{1}H\}$ NMR (125 MHz, $CDCl_3$) Spectrum of **3f**

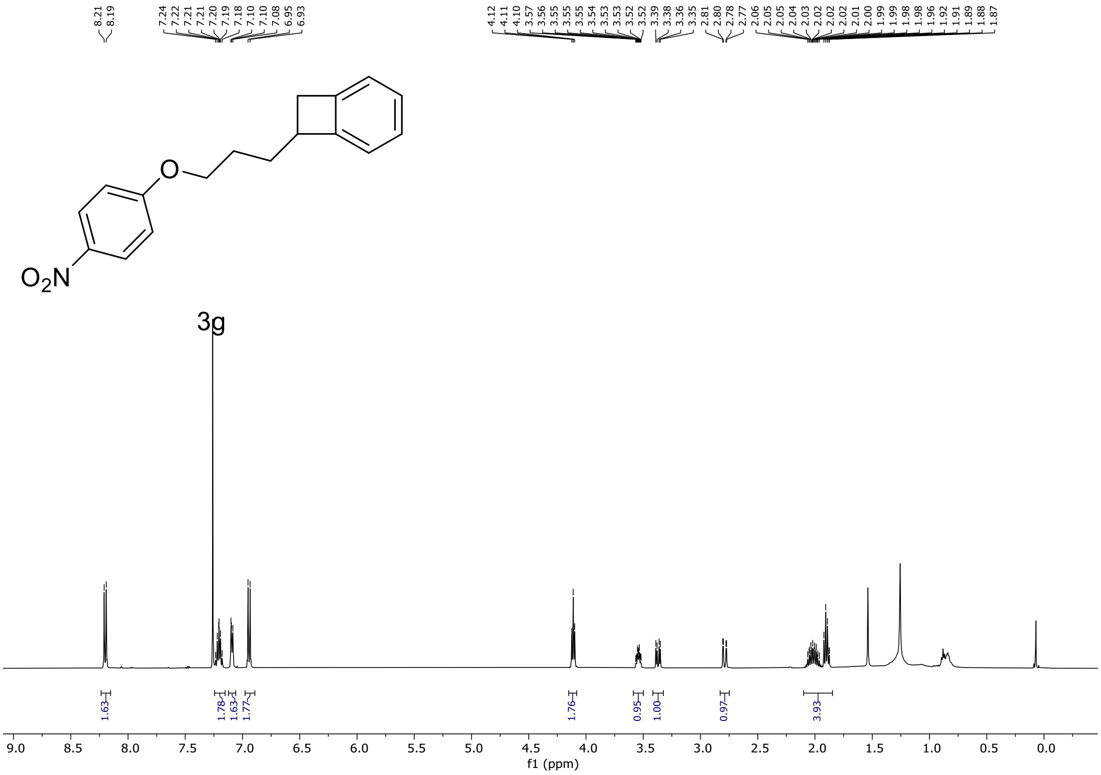


$^{1}H$ NMR (500 MHz, $CDCl_3$) Spectrum of **3g**

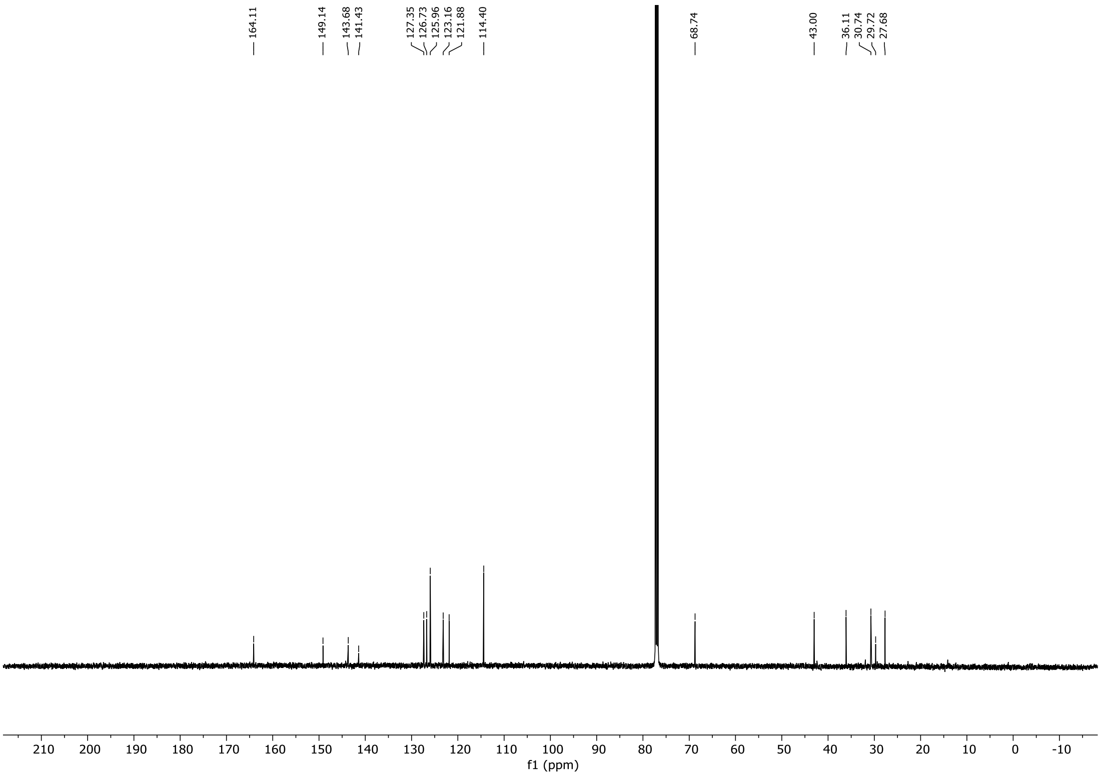


$^{13}C\{^{1}H\}$ NMR (125 MHz, $CDCl_3$) Spectrum of **3g**

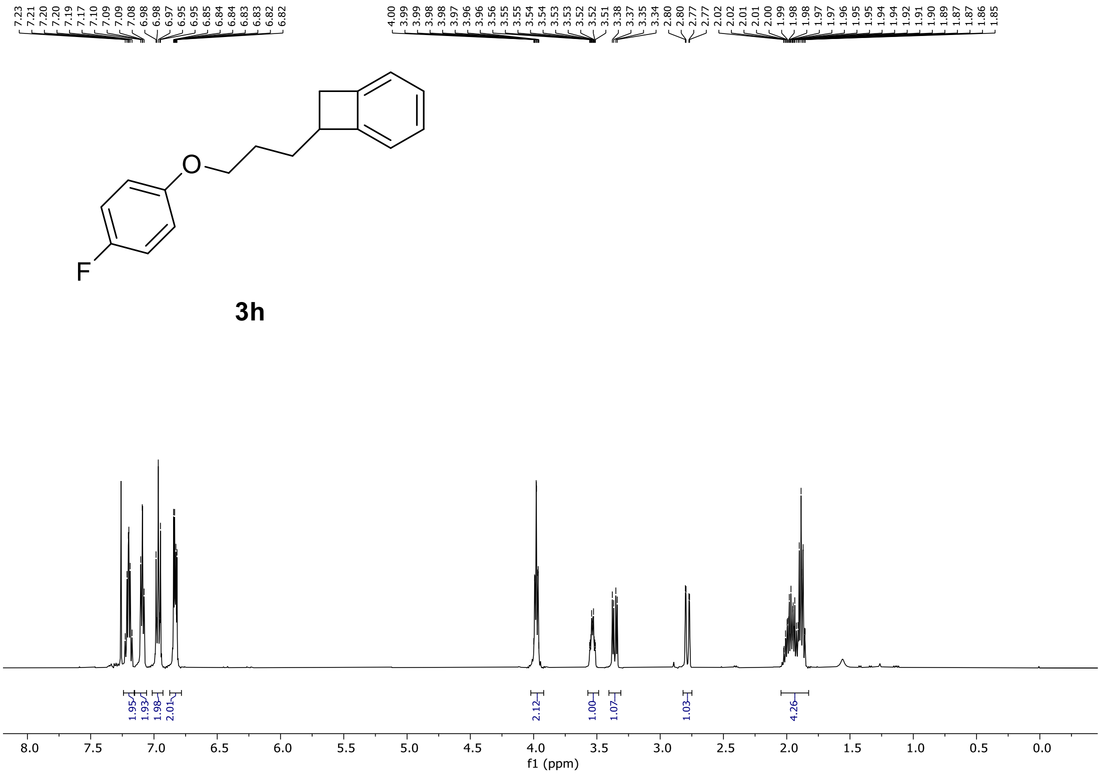


**3h**

$^{1}$H NMR (500 MHz, $CDCl_3$) Spectrum of **3h**

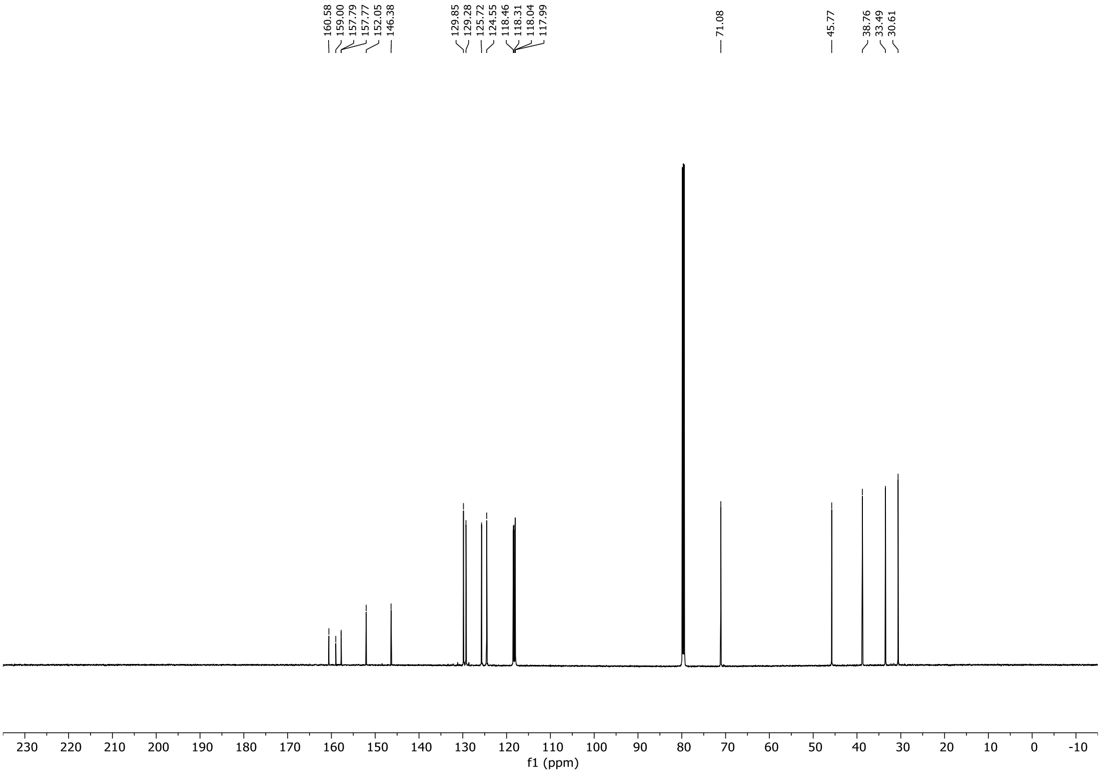


$^{13}C\{^{1}H\}$ NMR (125 MHz, $CDCl_3$) Spectrum of **3h**

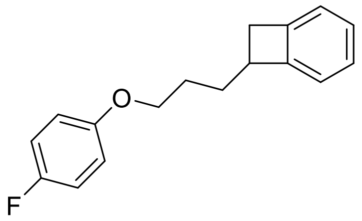

**3h**

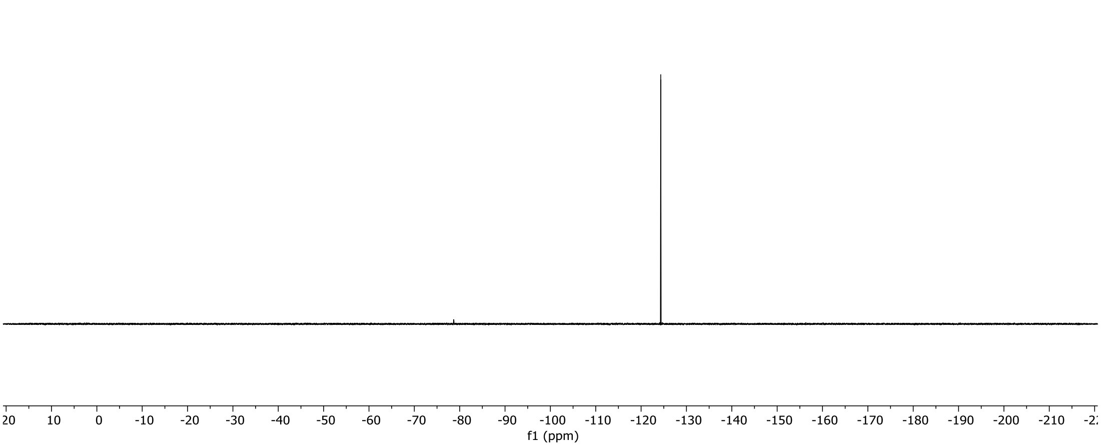


$^{19}$F NMR (471 MHz, $CDCl_3$) Spectrum of **3h**

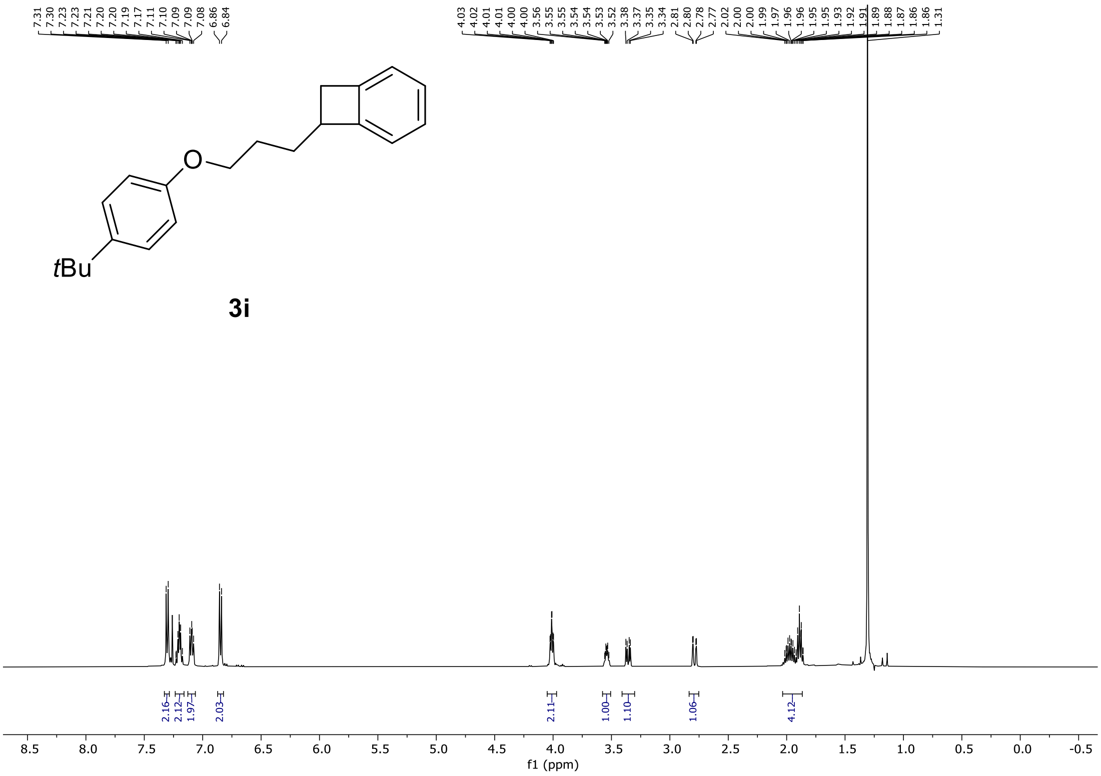


$^{1}$H NMR (500 MHz, $CDCl_3$) Spectrum of **3i**

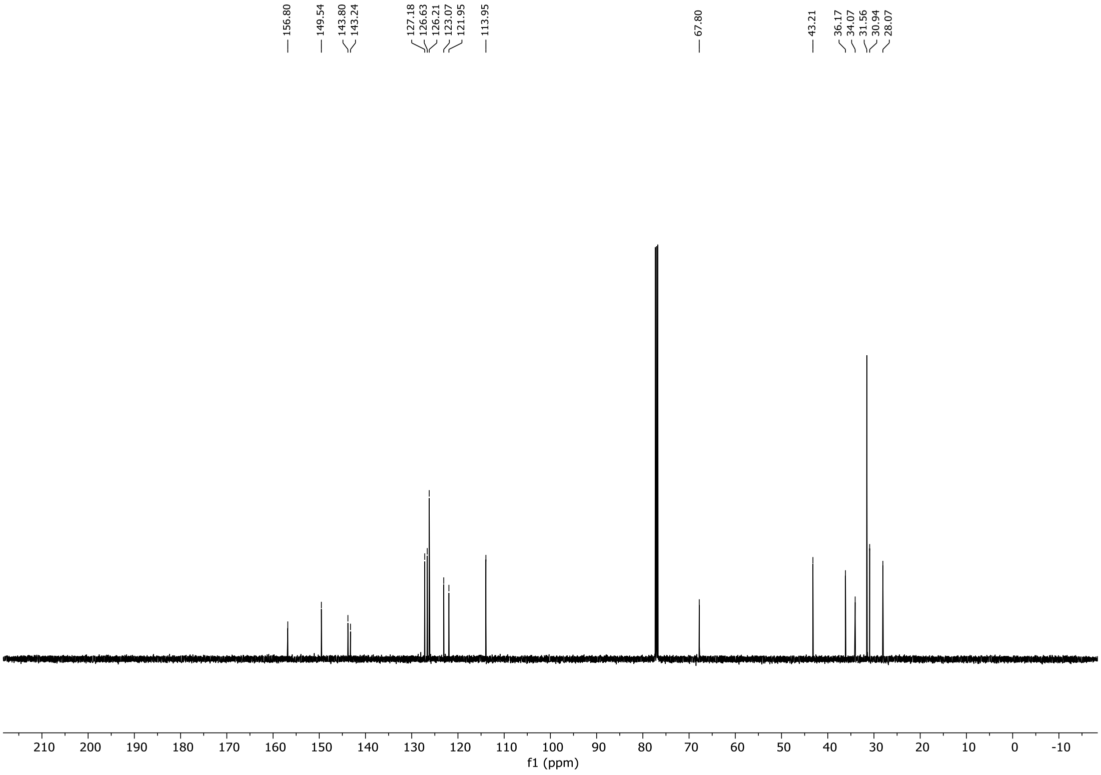


$^{13}$C{$^{1}$H} NMR (125 MHz, $CDCl_3$) Spectrum of **3i**

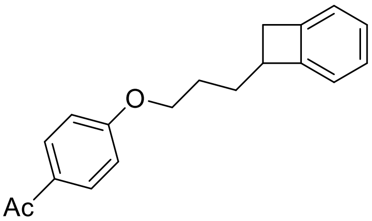


**3j**

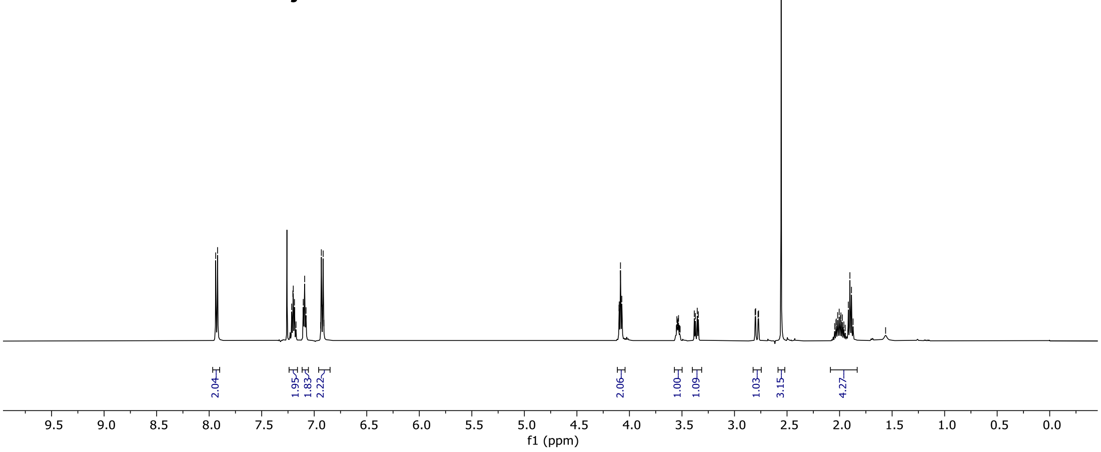


$^{1}$H NMR (500 MHz, $CDCl_3$) Spectrum of **3j**

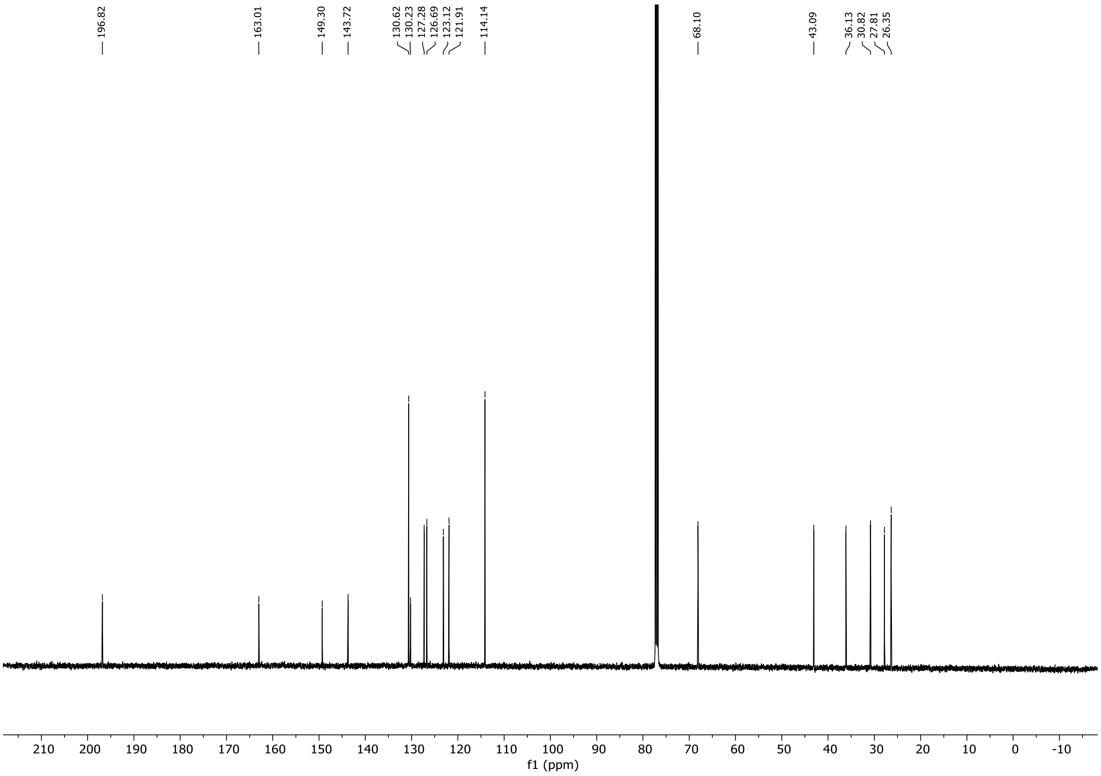


$^{13}$C{$^{1}$H} NMR (125 MHz, $CDCl_3$) Spectrum of **3j**

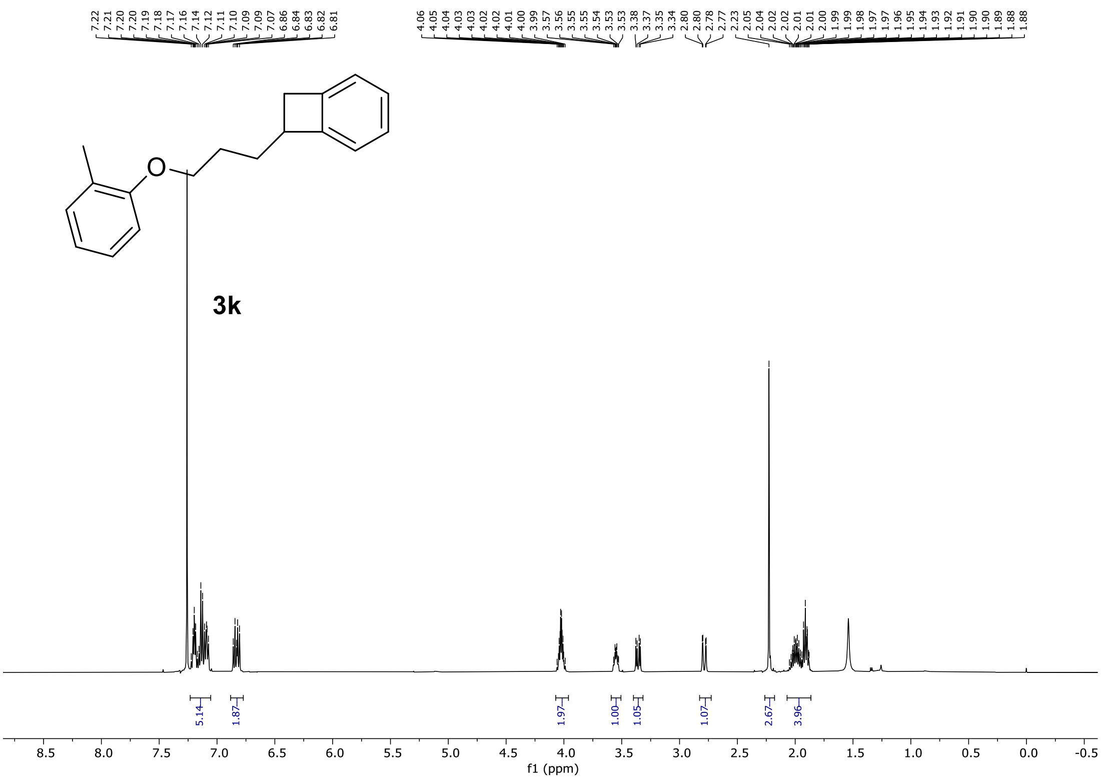


$^{1}$H NMR (500 MHz, $CDCl_3$) Spectrum of **3k**

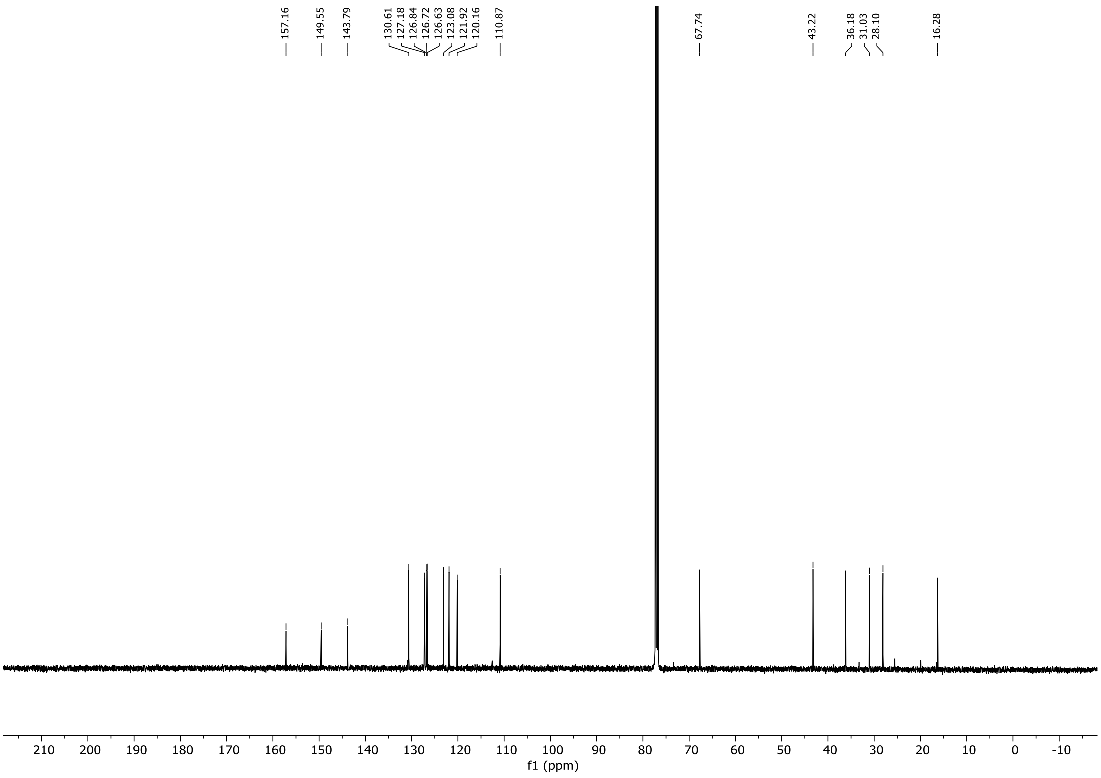


$^{13}$C{[1] H} NMR (125 MHz, $CDCl_3$) Spectrum of **3k**

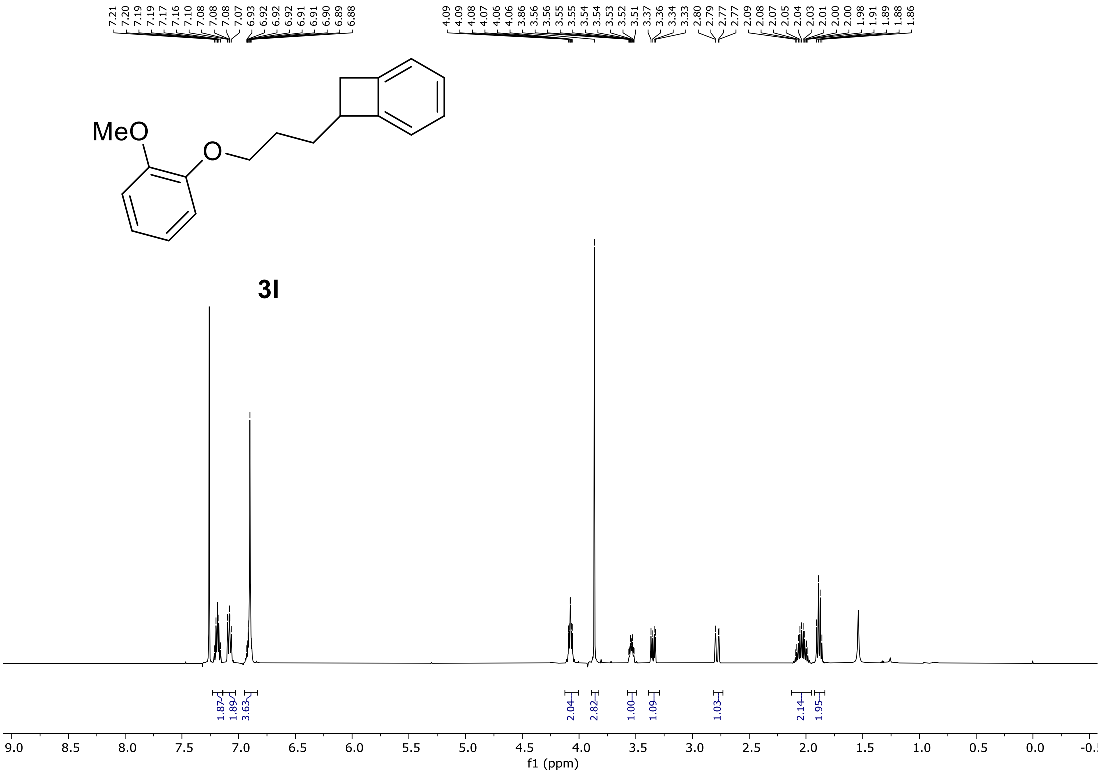


$^{1}H$ NMR (500 MHz, $CDCl_3$) Spectrum of **3l**

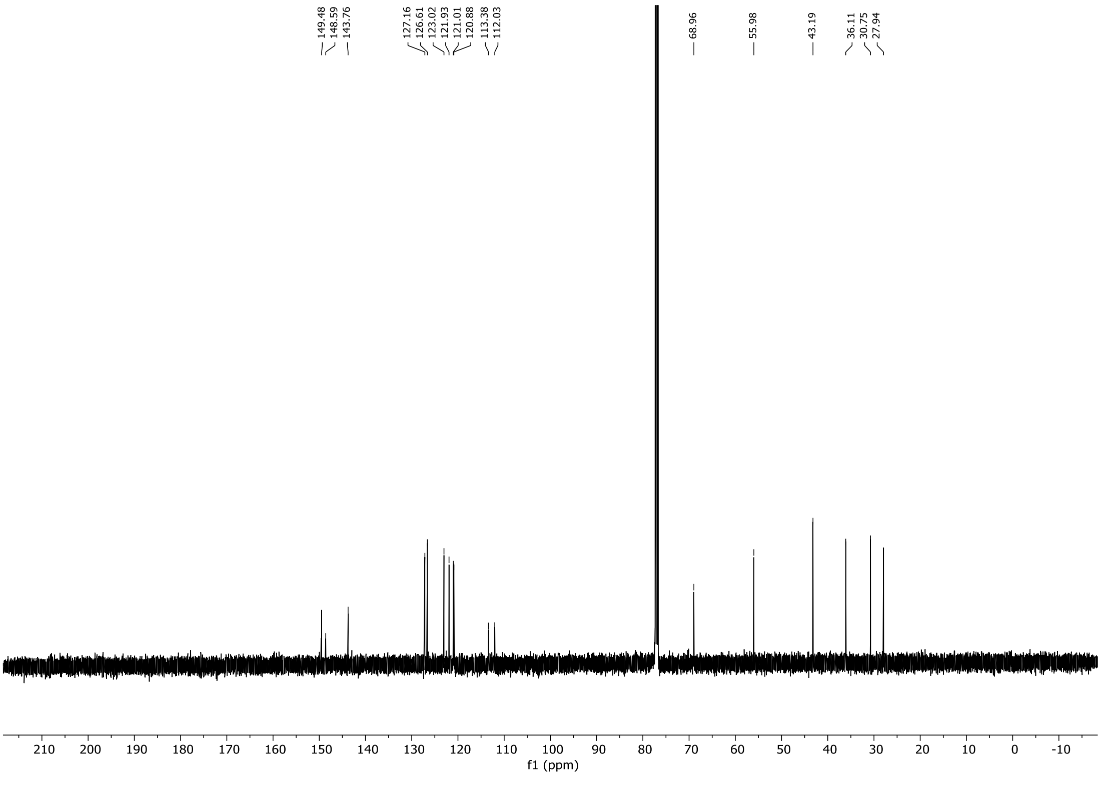


$^{13}C\{^{1}H\}$ NMR (125 MHz, $CDCl_3$) Spectrum of **3l**

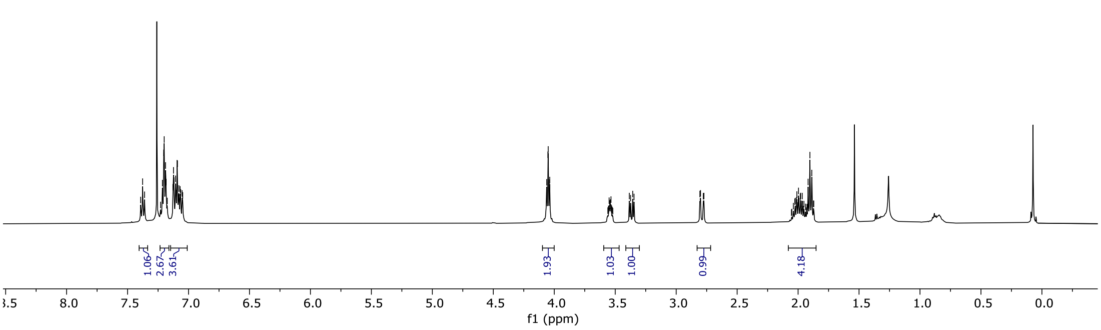

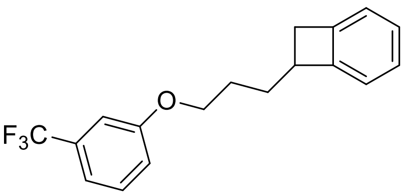


**3m**



$^{1}$H NMR (500 MHz, $CDCl_3$) Spectrum of **3m**

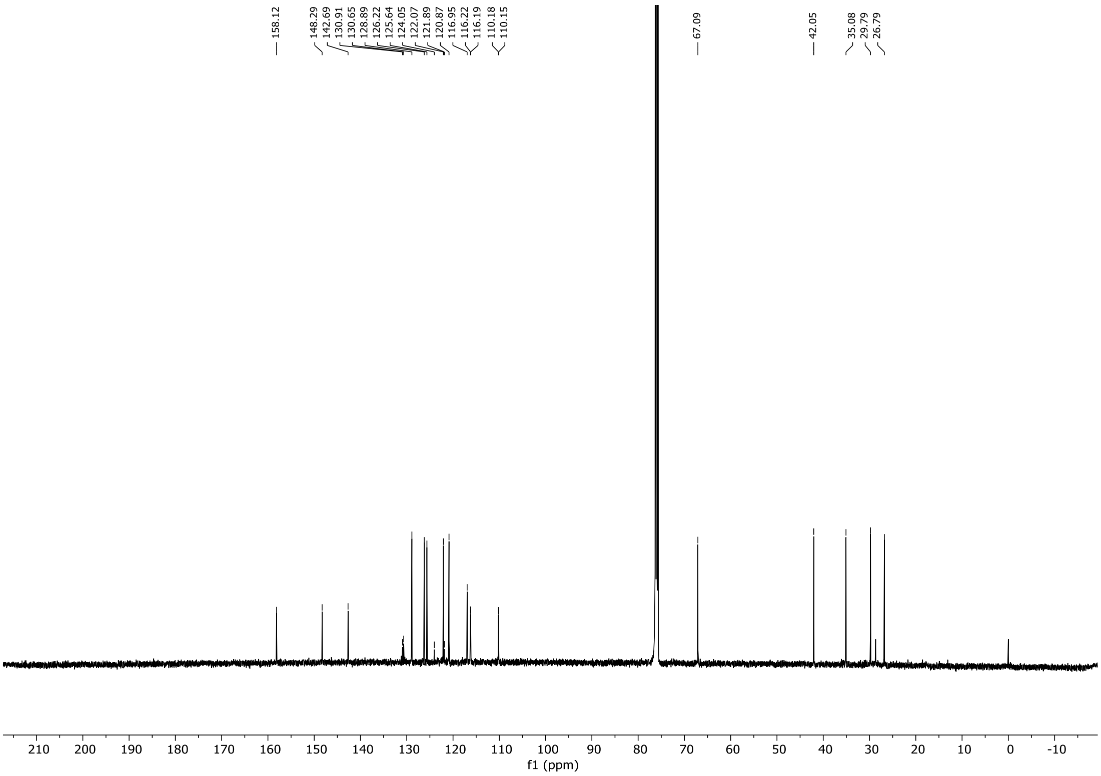


$^{13}$C{$^{1}$H} NMR (125 MHz, $CDCl_3$) Spectrum of **3m**

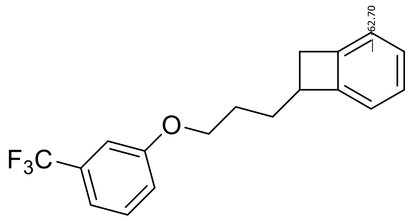


**3m**

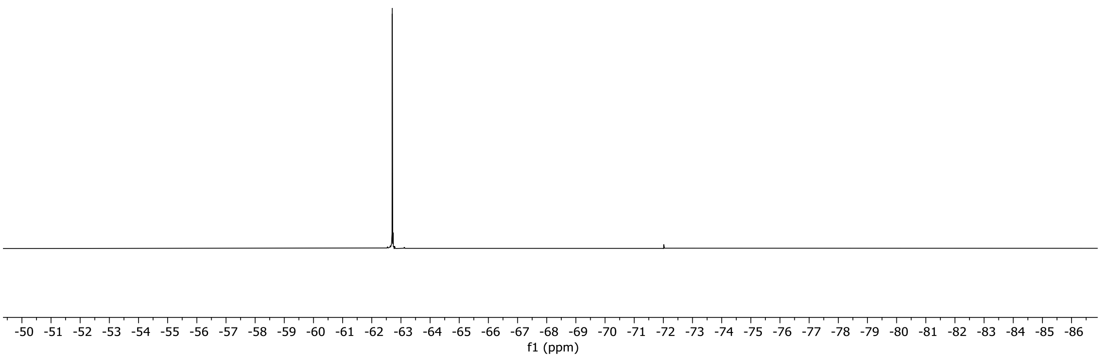


$^{19}$F NMR (471 MHz, $CDCl_3$) Spectrum of **3m**

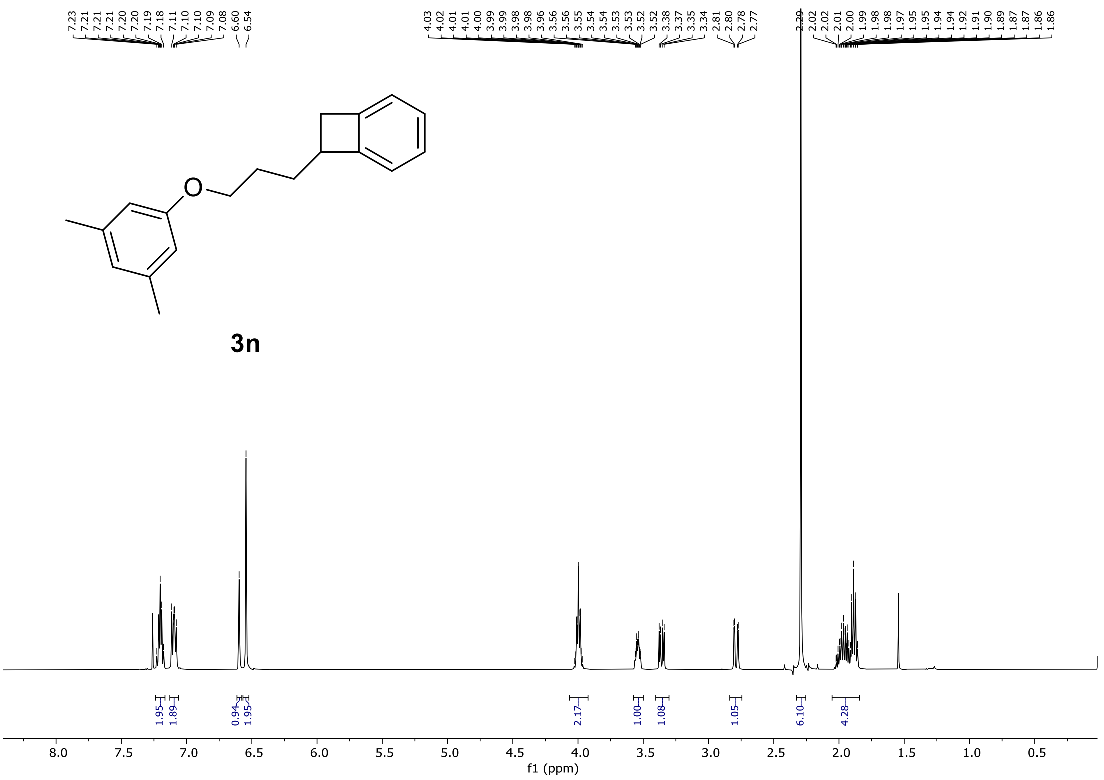


$^{1}$H NMR (500 MHz, $CDCl_3$) Spectrum of **3n**

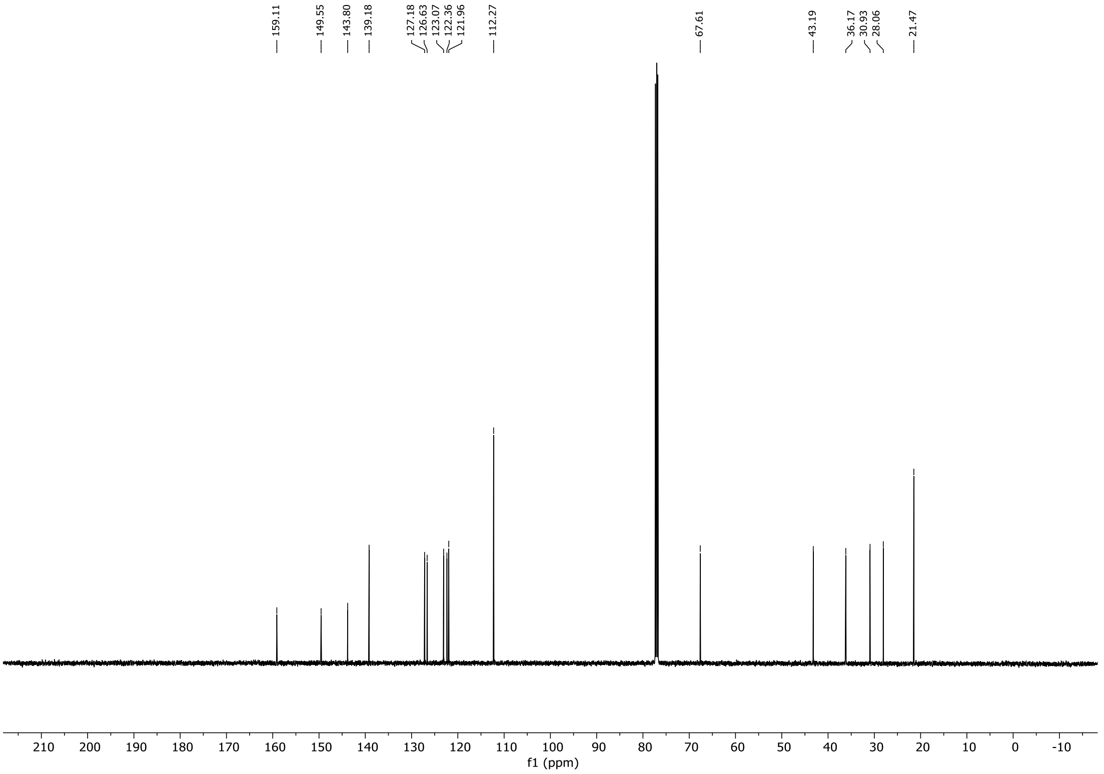


$^{13}C\{^1H\}$ NMR (125 MHz, $CDCl_3$) Spectrum of **3n**

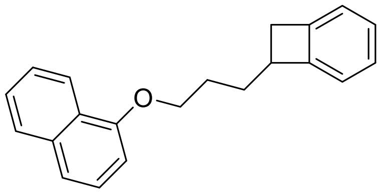

**3o**

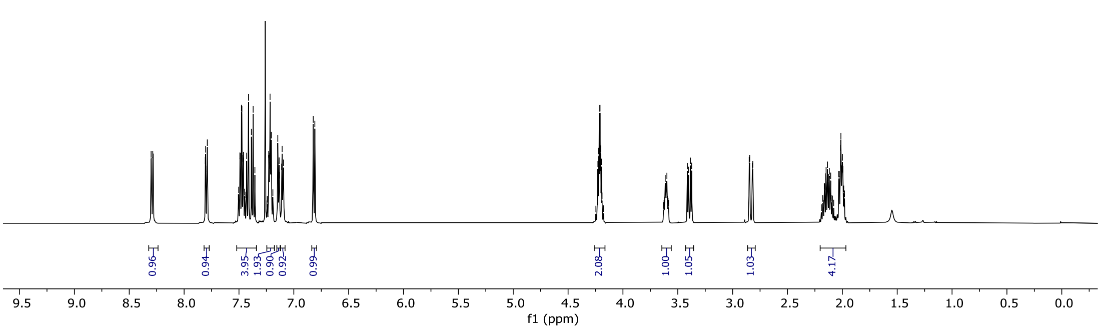

$^{1}$H NMR (500 MHz, $CDCl_3$) Spectrum of **3o**

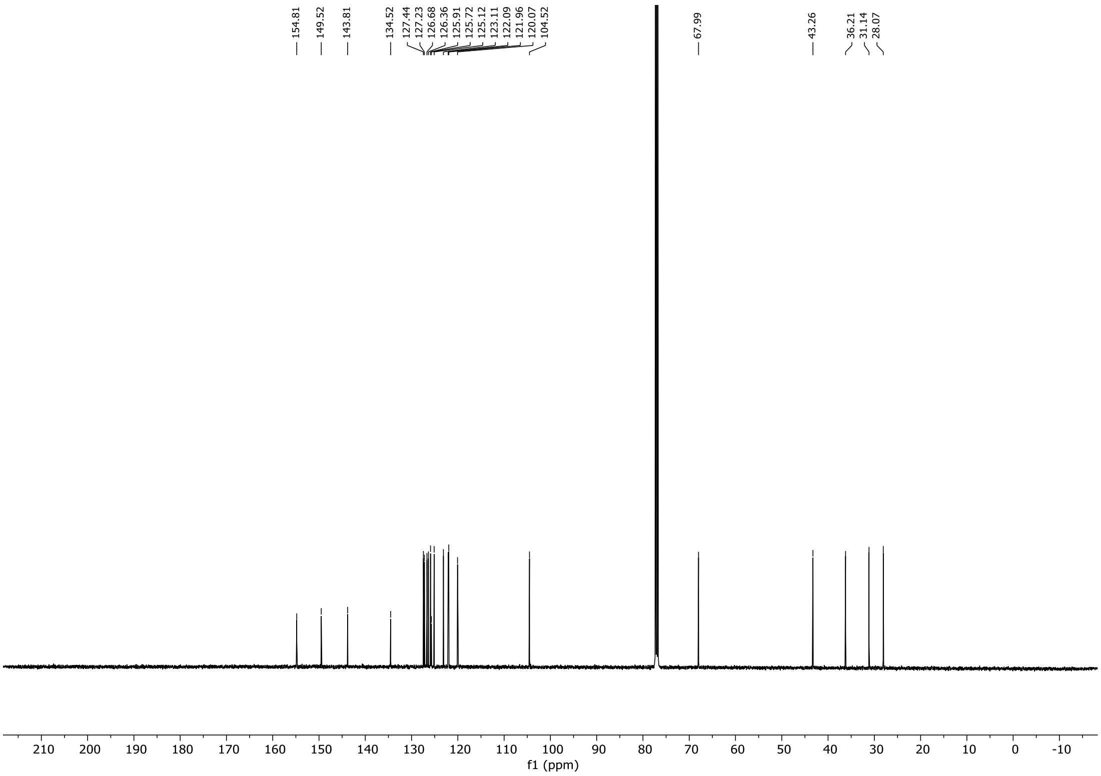

$^{13}$C{[1] H} NMR (125 MHz, $CDCl_3$) Spectrum of **3o**

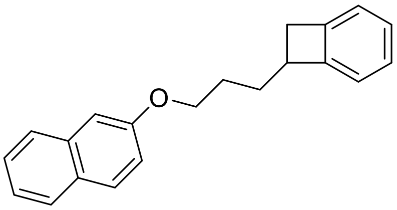

**3p**

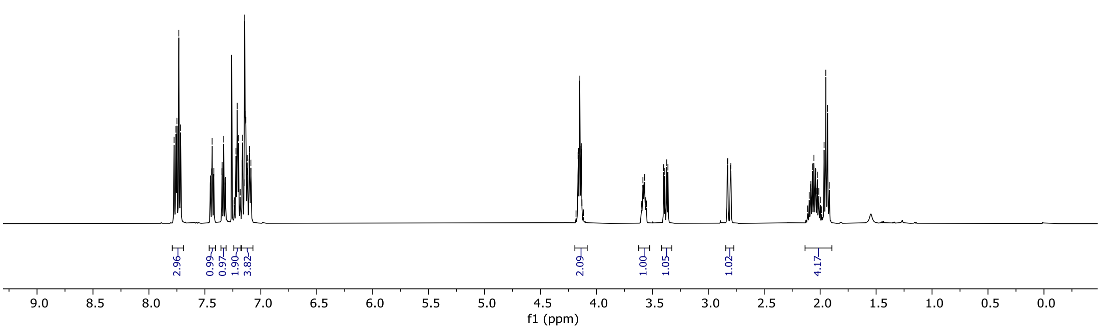


$^{1}$H NMR (500 MHz, $CDCl_3$) Spectrum of **3p**

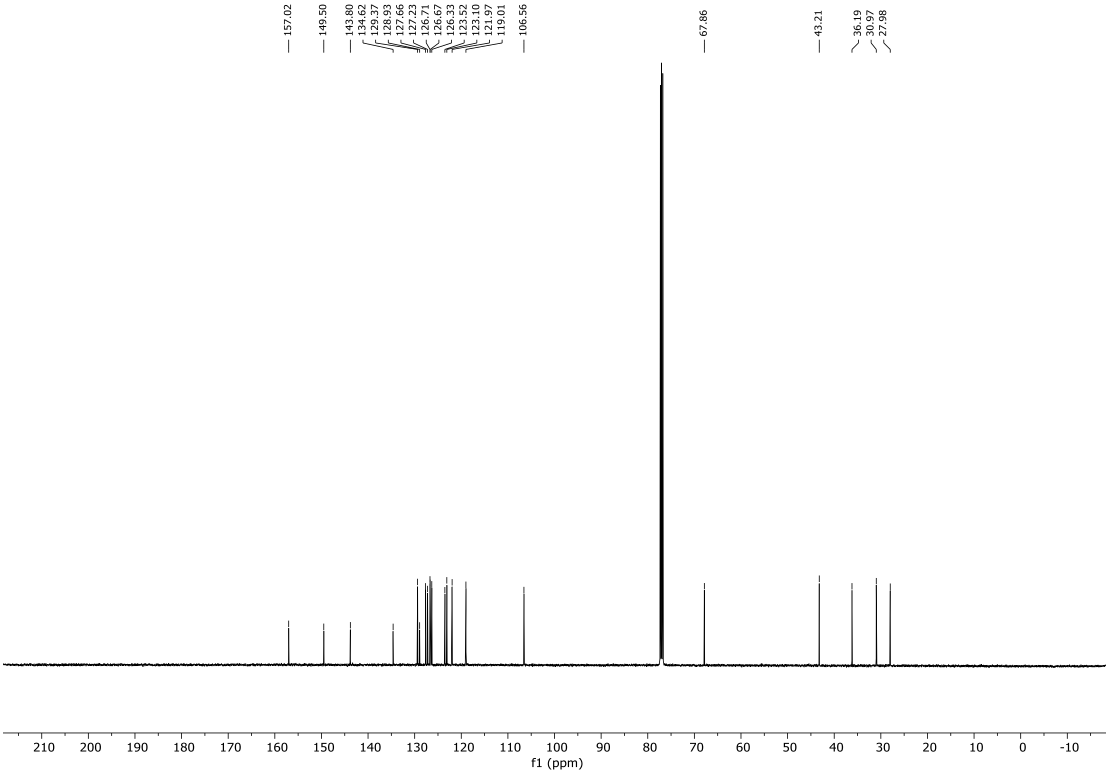


$^{13}$C{[1] H} NMR (125 MHz, $CDCl_3$) Spectrum of **3p**

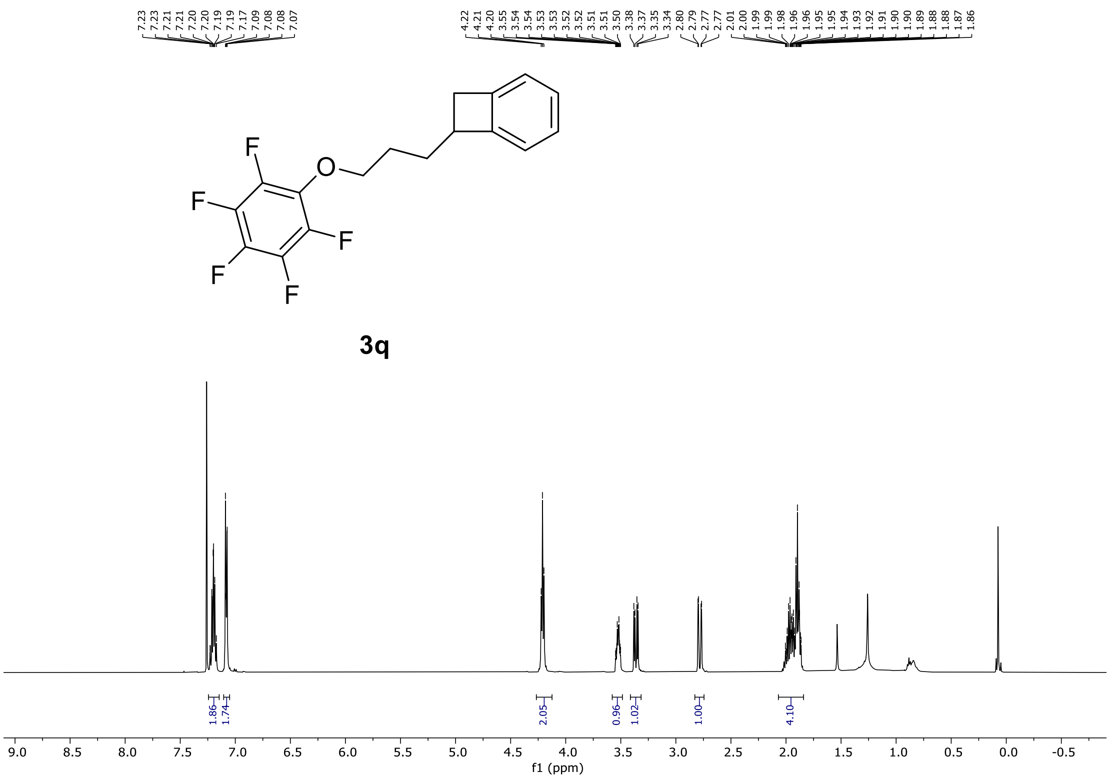


$^{1}$H NMR (500 MHz, $CDCl_3$) Spectrum of **3q**

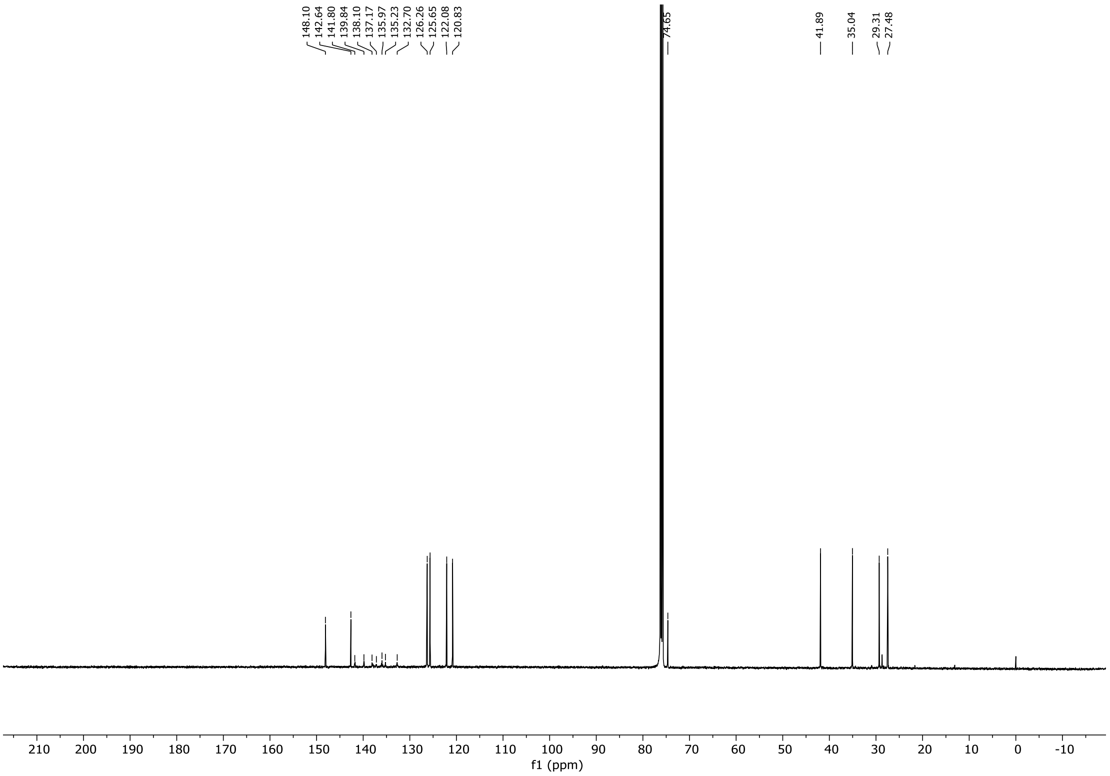


$^{13}$C{$^{1}$H} NMR (125 MHz, $CDCl_3$) Spectrum of **3q**

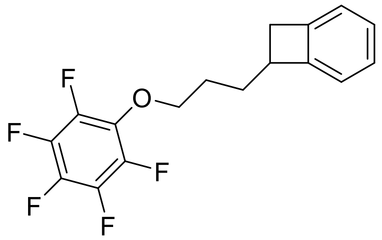

**3q**

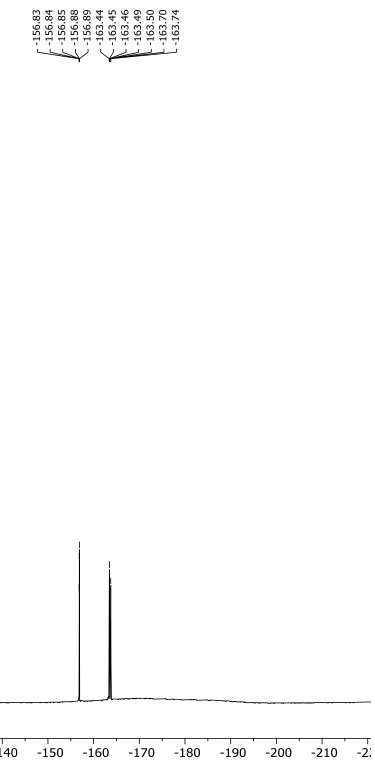


$^{19}$F NMR (471 MHz, $CDCl_3$) Spectrum of **3q**

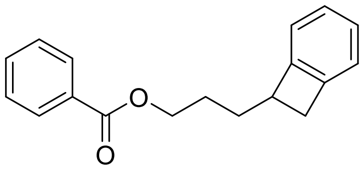

**3r**

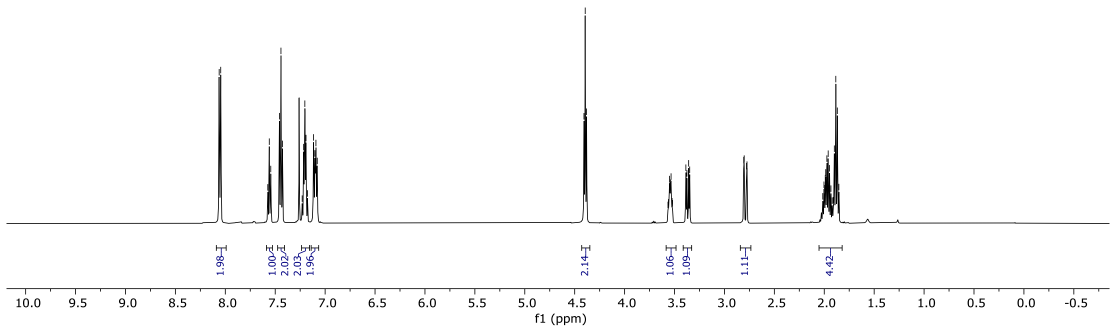


$^{1}$H NMR (500 MHz, $CDCl_3$) Spectrum of **3r**

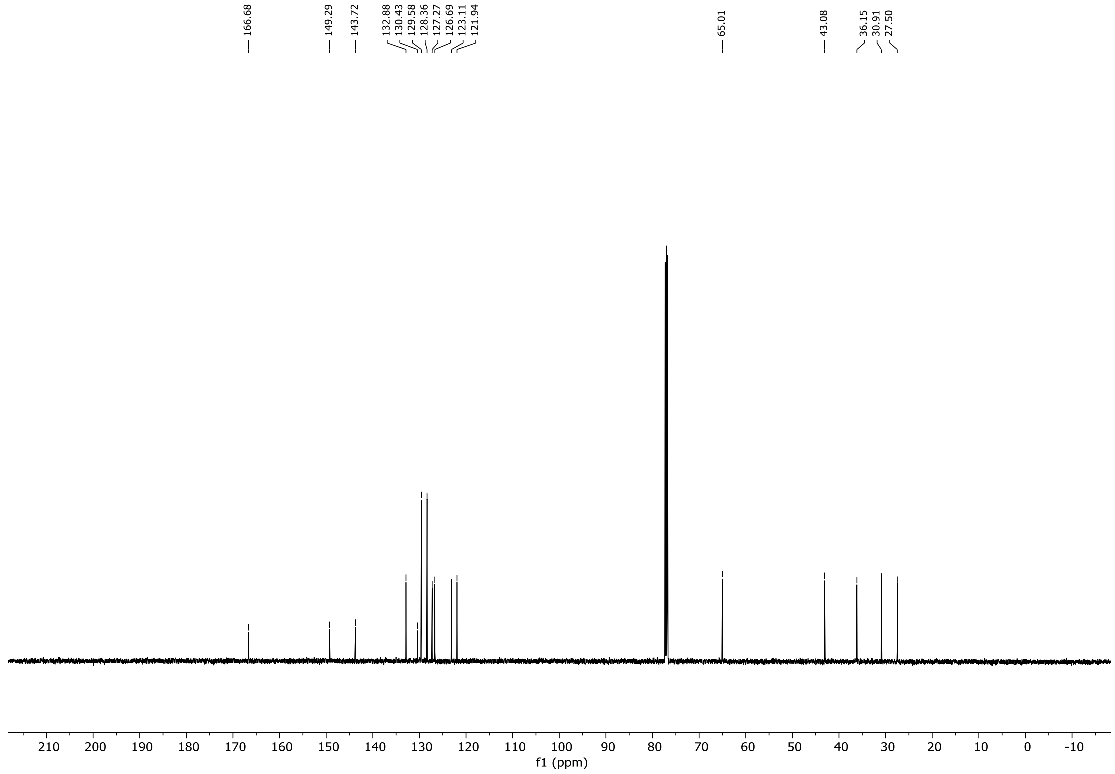


$^{13}$C{$^{1}$H} NMR (125 MHz, $CDCl_3$) Spectrum of **3r**

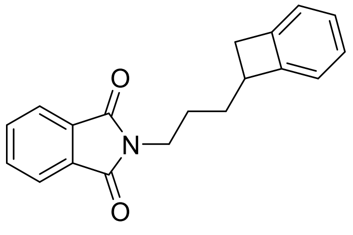


**3s**

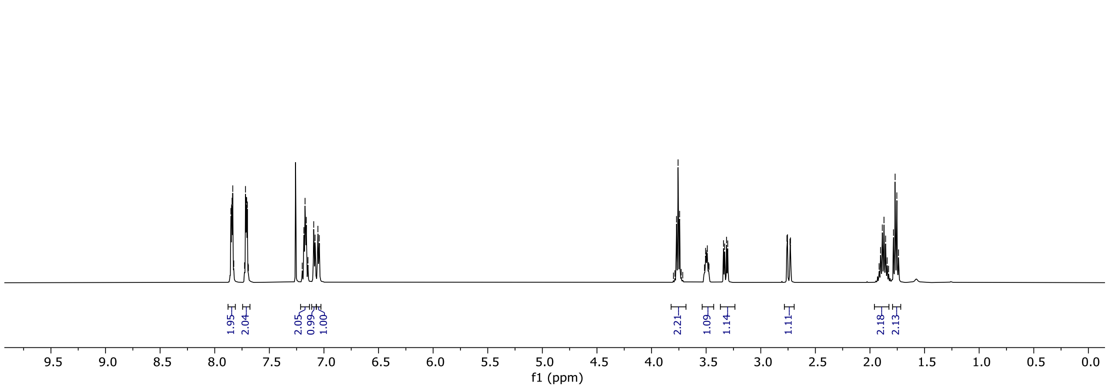


$^{1}H$ NMR (500 MHz, $CDCl_3$) Spectrum of **3s**

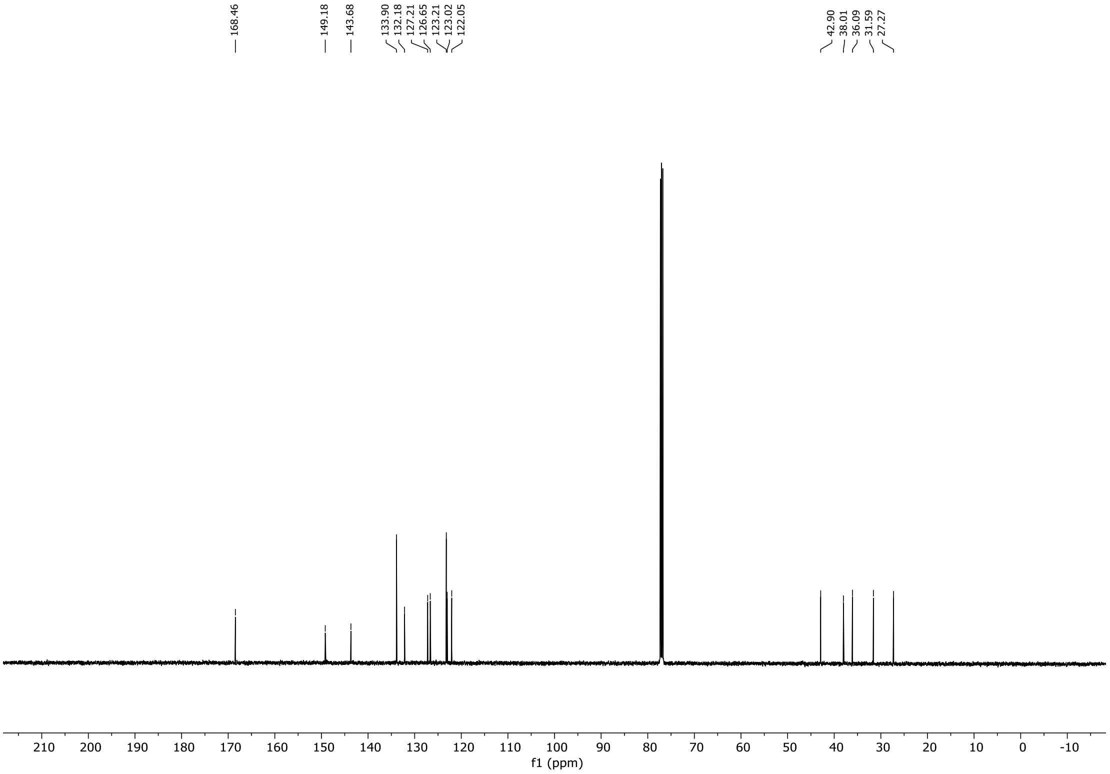


$^{13}C\{^{1}H\}$ NMR (125 MHz, $CDCl_3$) Spectrum of **3s**

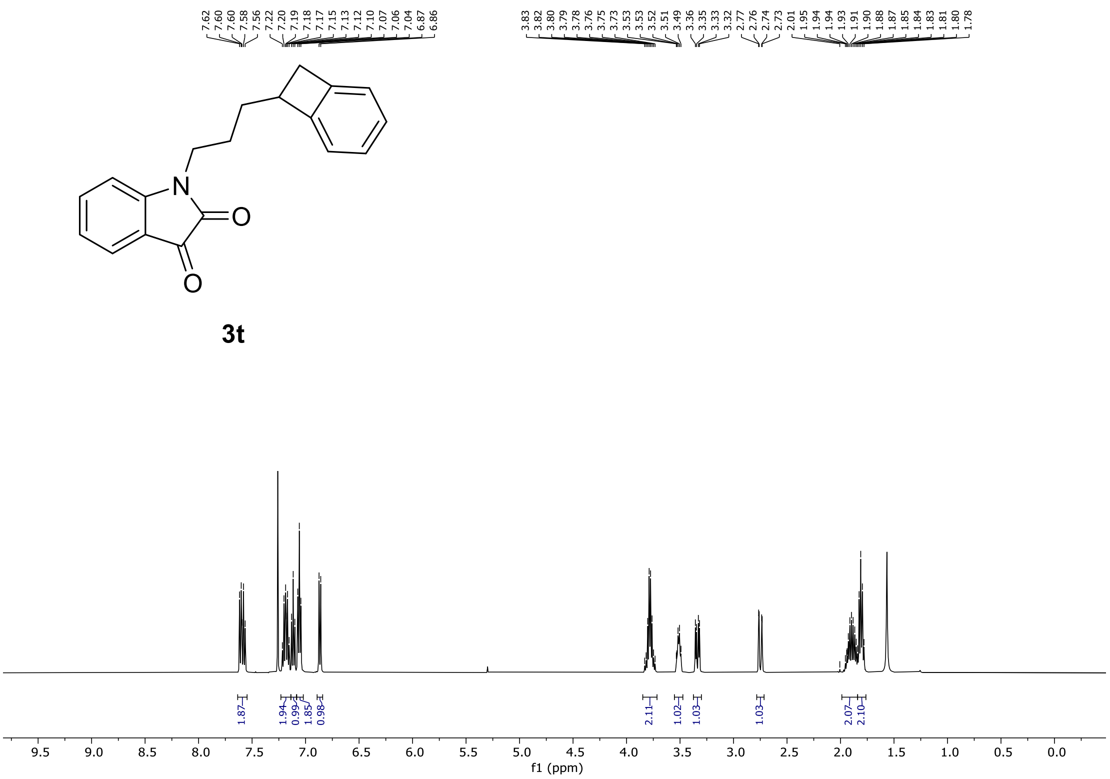


$^{1}$H NMR (500 MHz, $CDCl_3$) Spectrum of **3t**

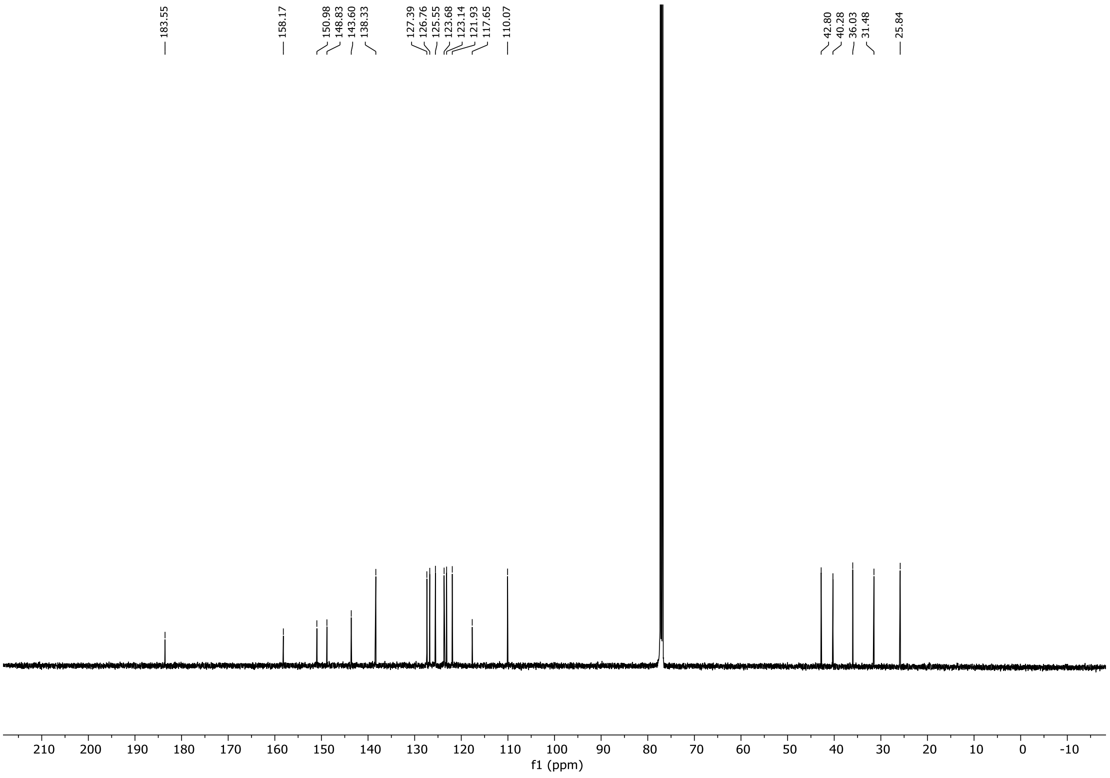


$^{13}$C{$^{1}$H} NMR (125 MHz, $CDCl_3$) Spectrum of **3t**

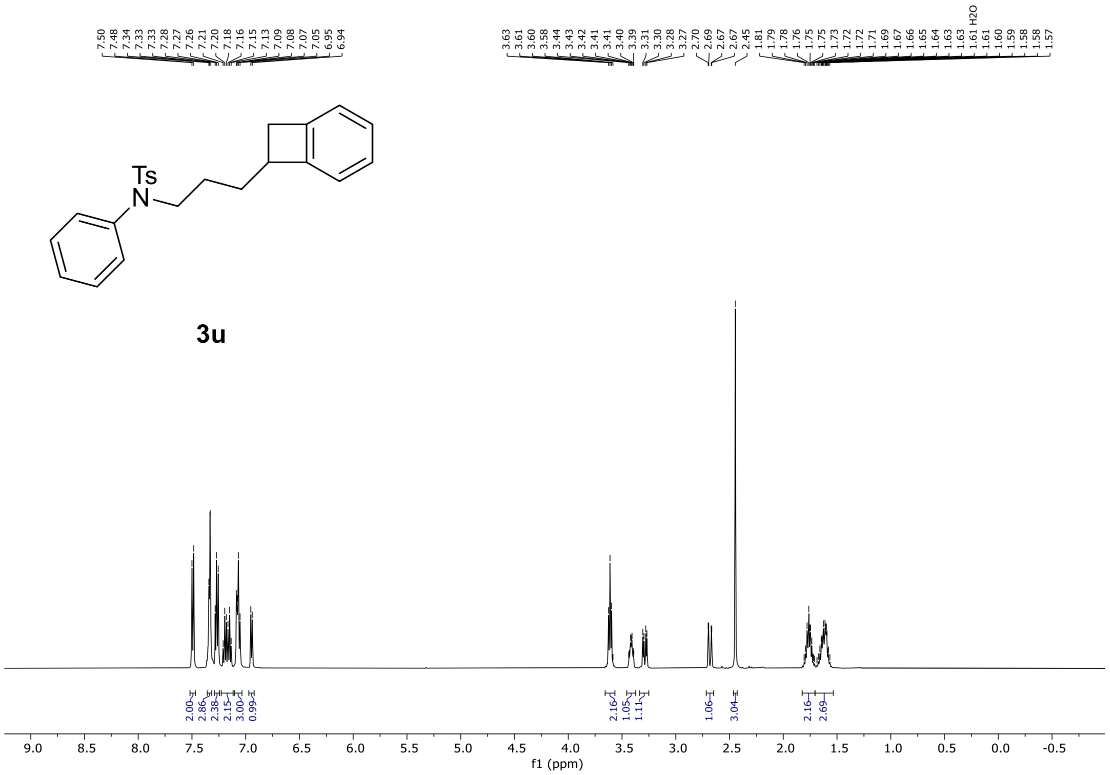


$^{1}H$ NMR (500 MHz, $CDCl_3$) Spectrum of **3u**

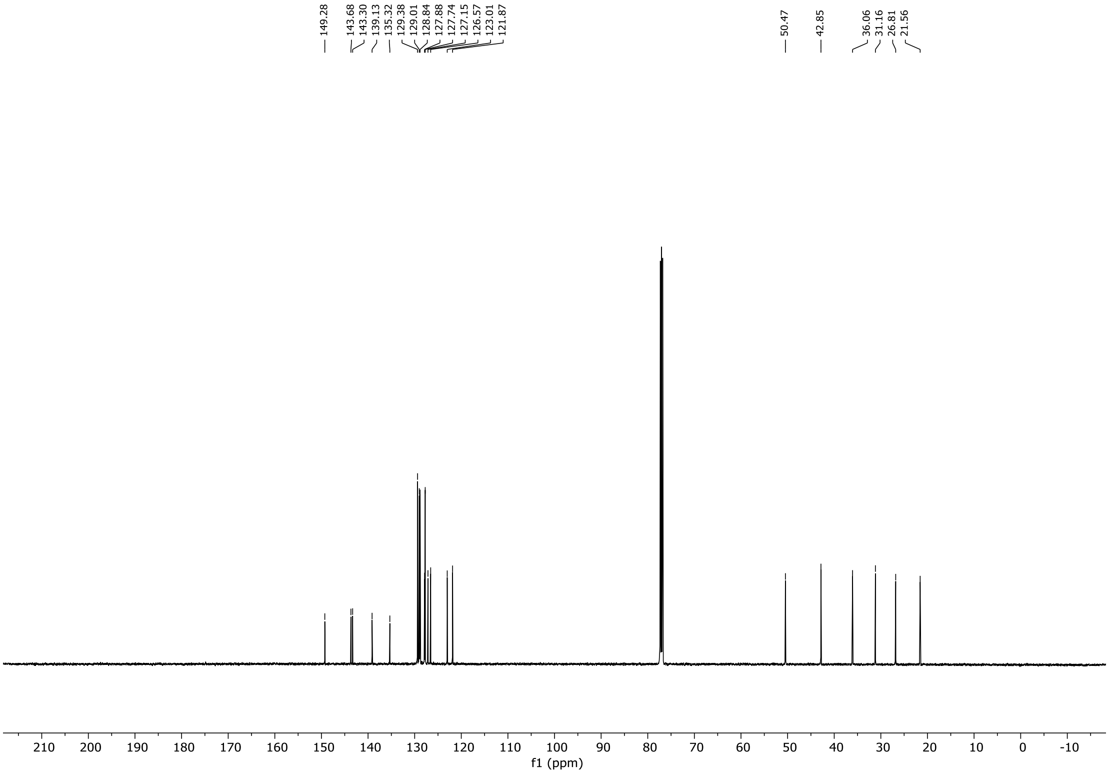


$^{13}C\{^{1}H\}$ NMR (125 MHz, $CDCl_3$) Spectrum of **3u**

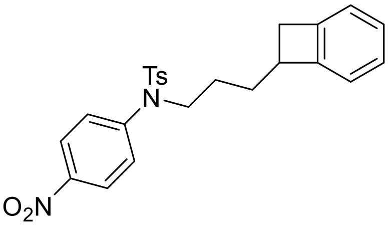

**3v**

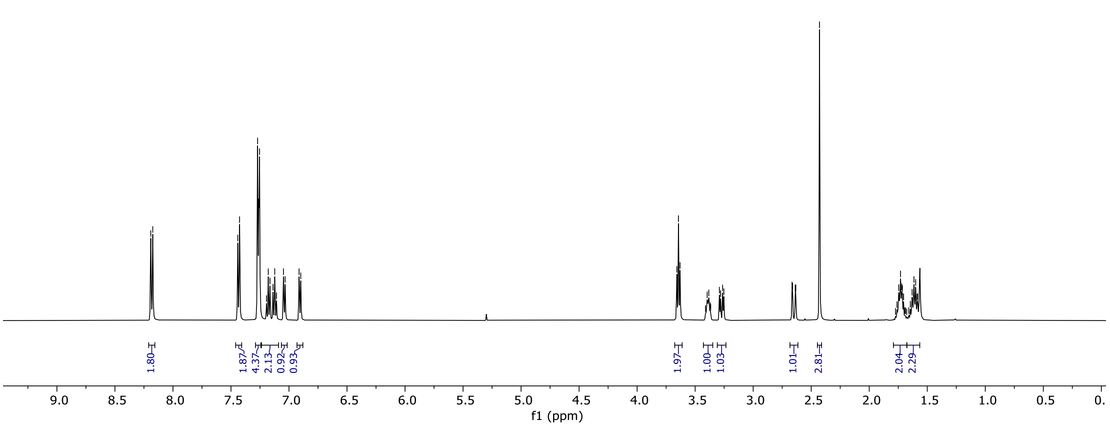


$^{1}$H NMR (500 MHz, $CDCl_3$) Spectrum of **3v**

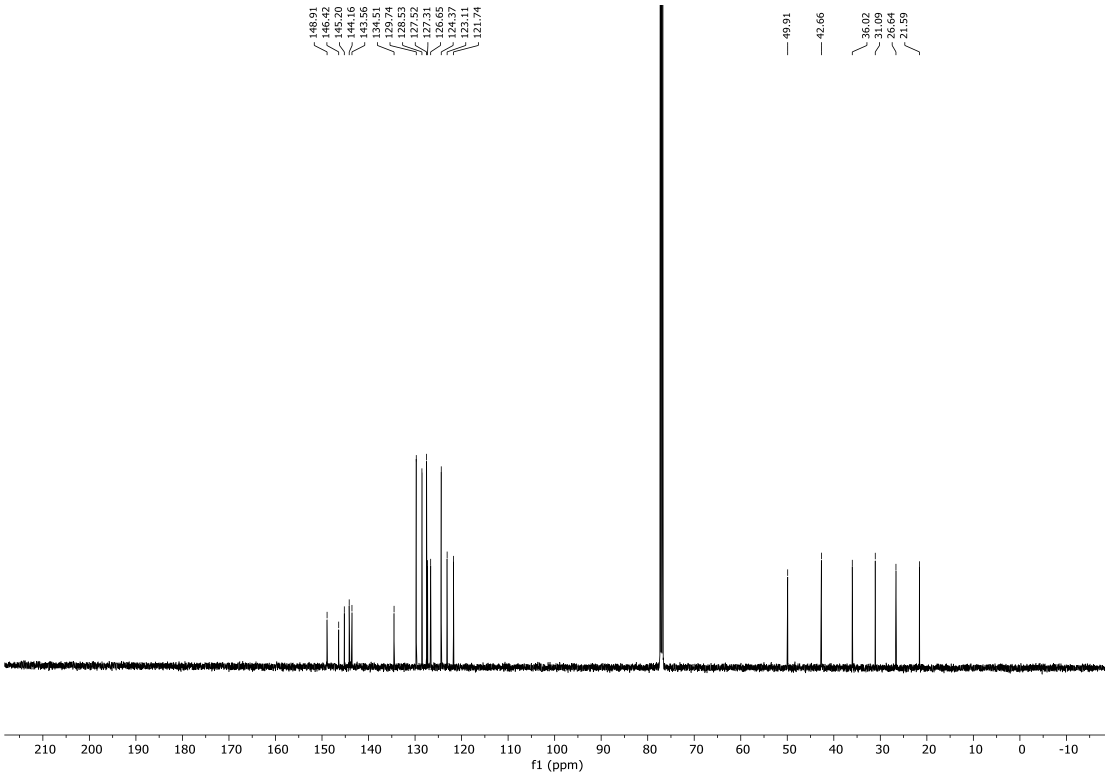


$^{13}C\{^{1}H\}$ NMR (125 MHz, $CDCl_3$) Spectrum of **3v**

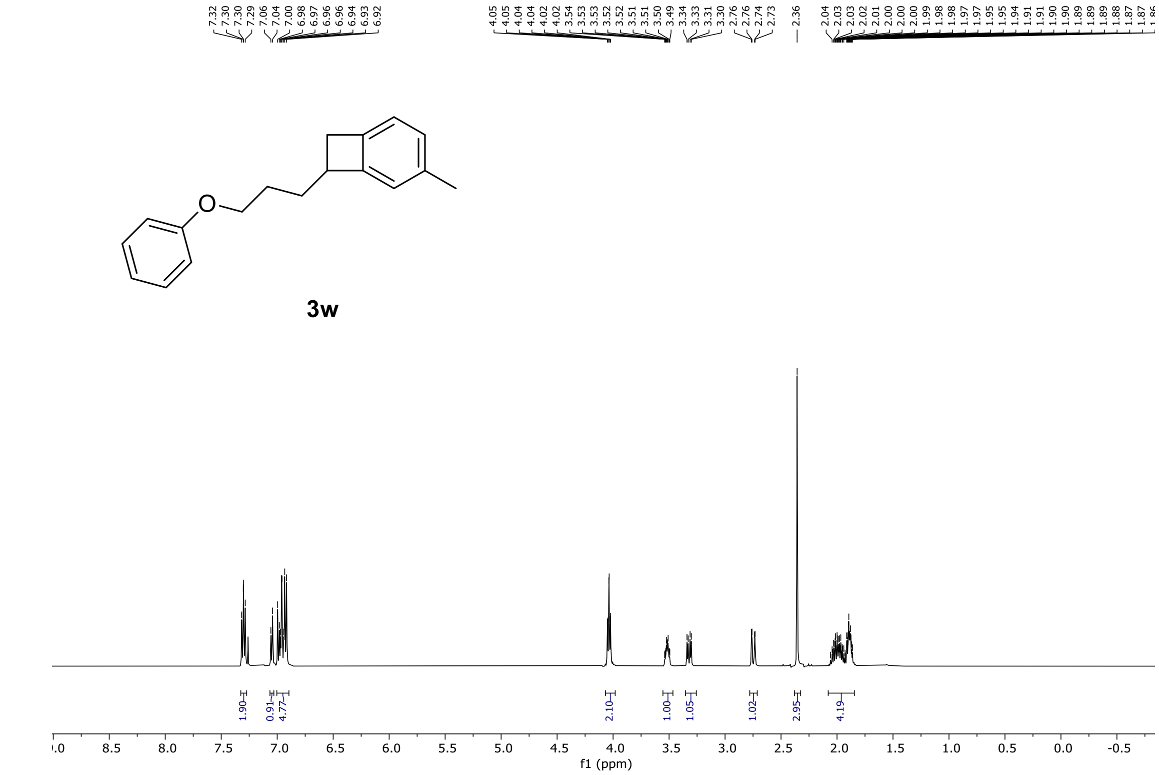


$^{1}H$ NMR (500 MHz, $CDCl_3$) Spectrum of **3w**

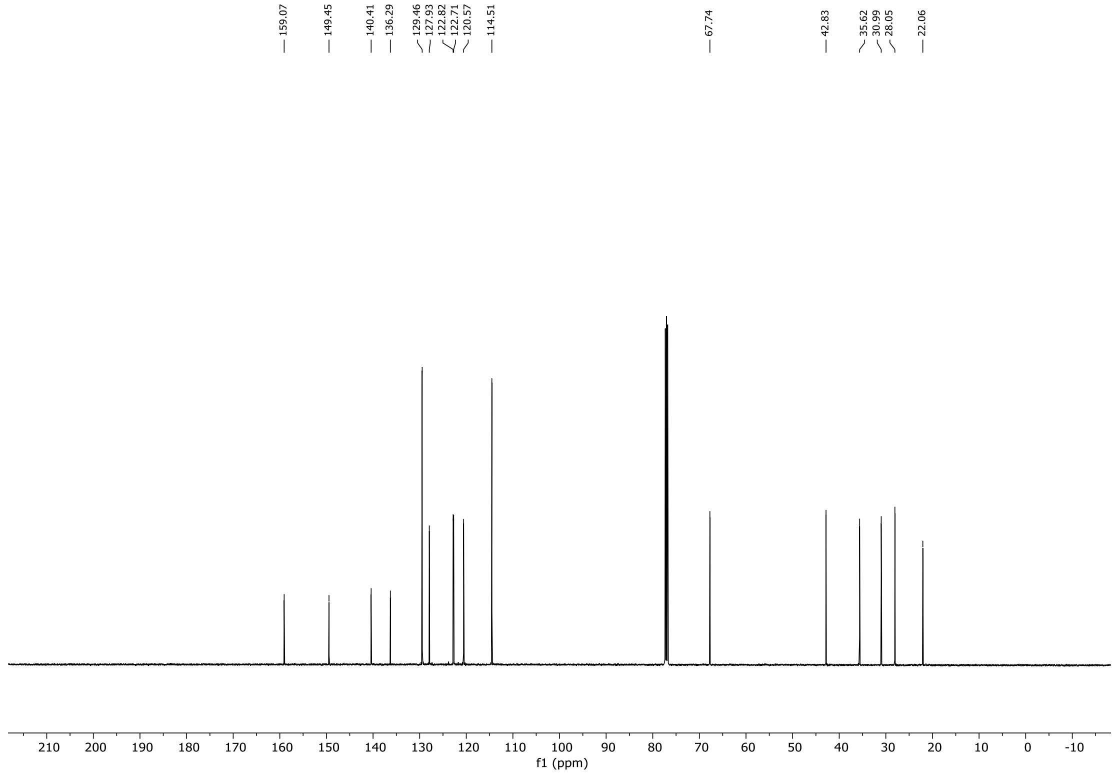


$^{13}C\{^{1}H\}$ NMR (125 MHz, $CDCl_3$) Spectrum of **3w**

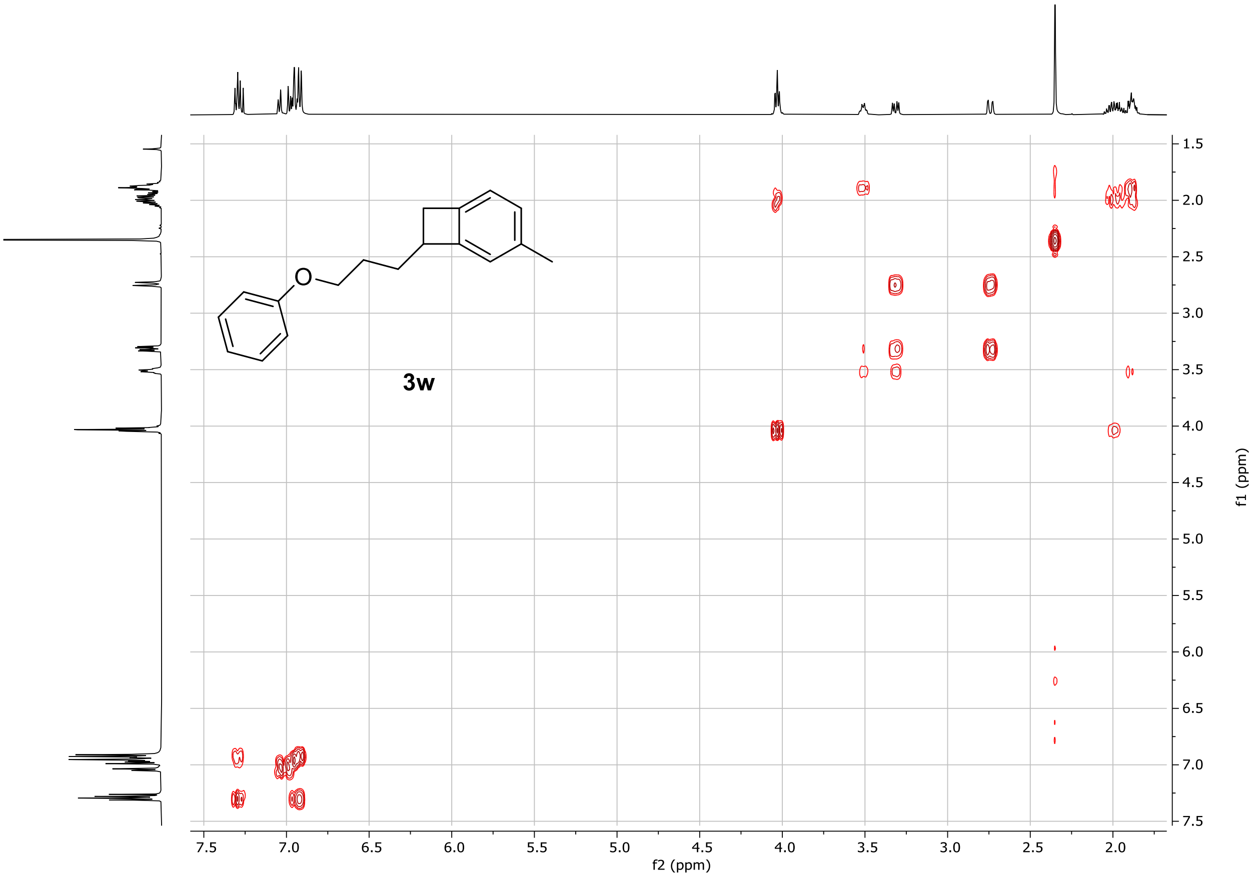


$^{1}H$–$^{1}H$ COSY (500 MHz, $CDCl_3$) Spectrum of **3w**

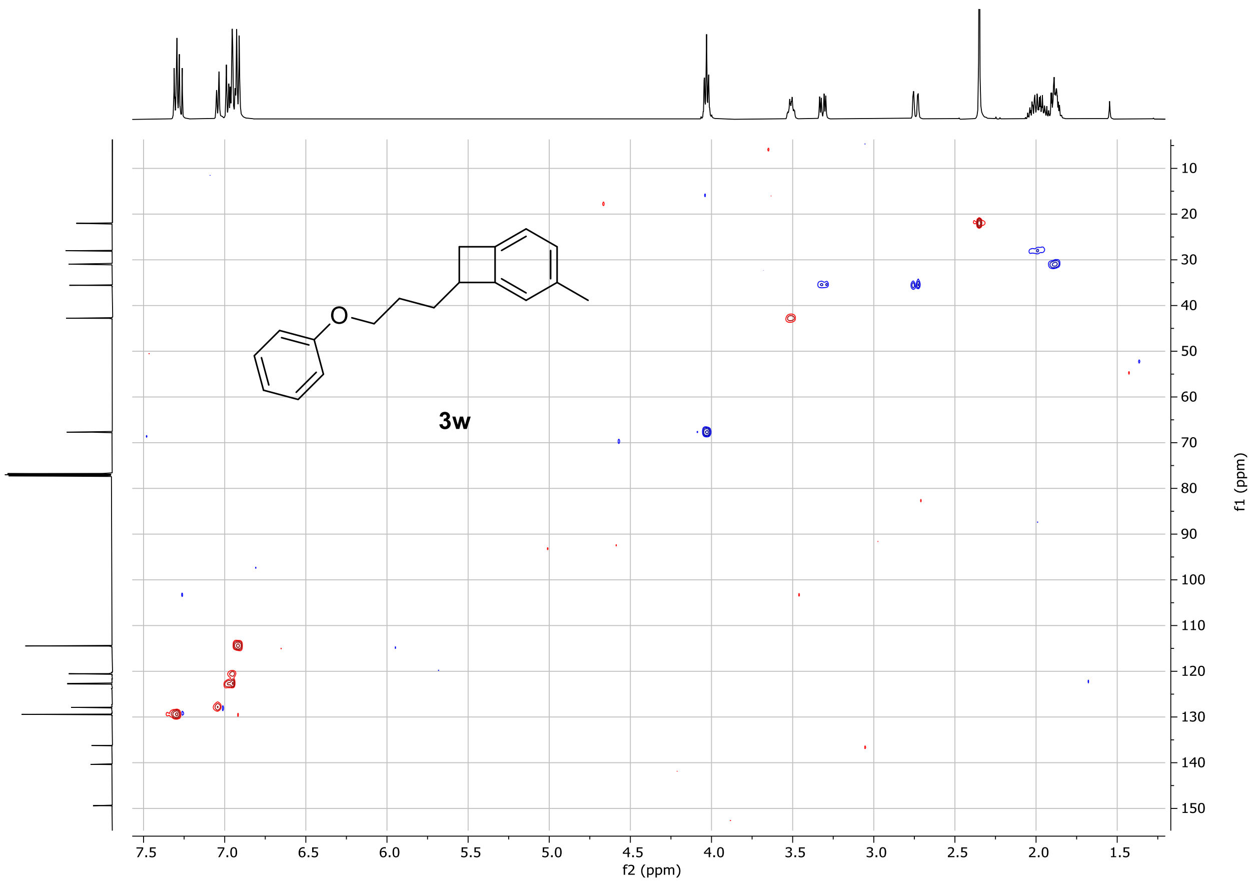


$^{1}H$–$^{13}C$ HSQC (500/125 MHz, $CDCl_3$) Spectrum of **3w**

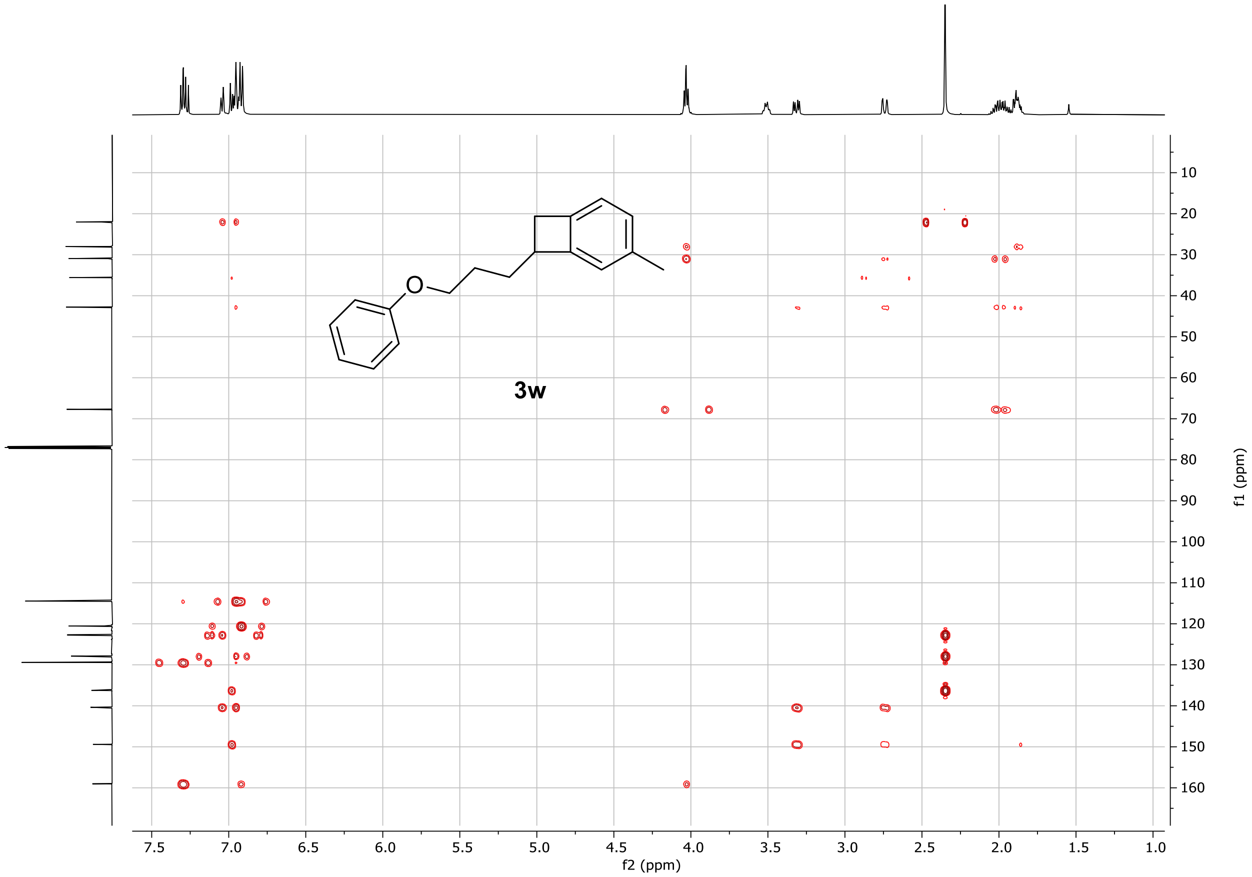


$^1$H–$^{13}$C HMBC (500/125 MHz, $CDCl_3$) Spectrum of **3w**

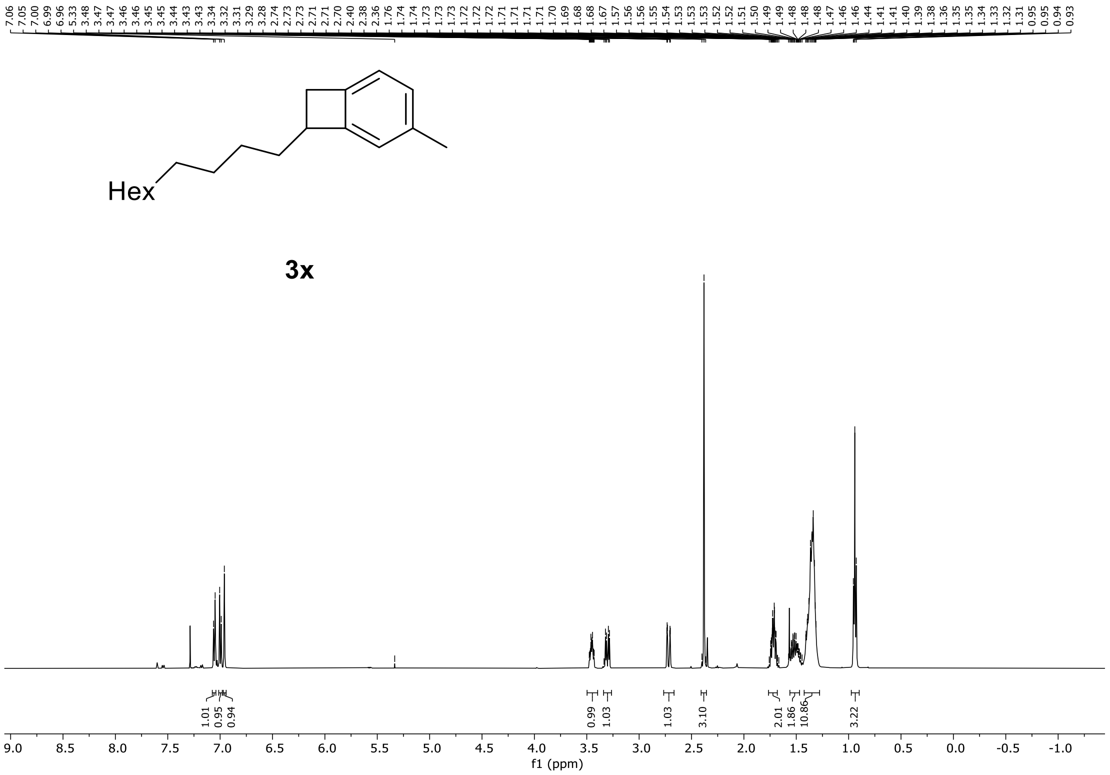


$^{1}H$ NMR (500 MHz, $CDCl_3$) Spectrum of **3x**

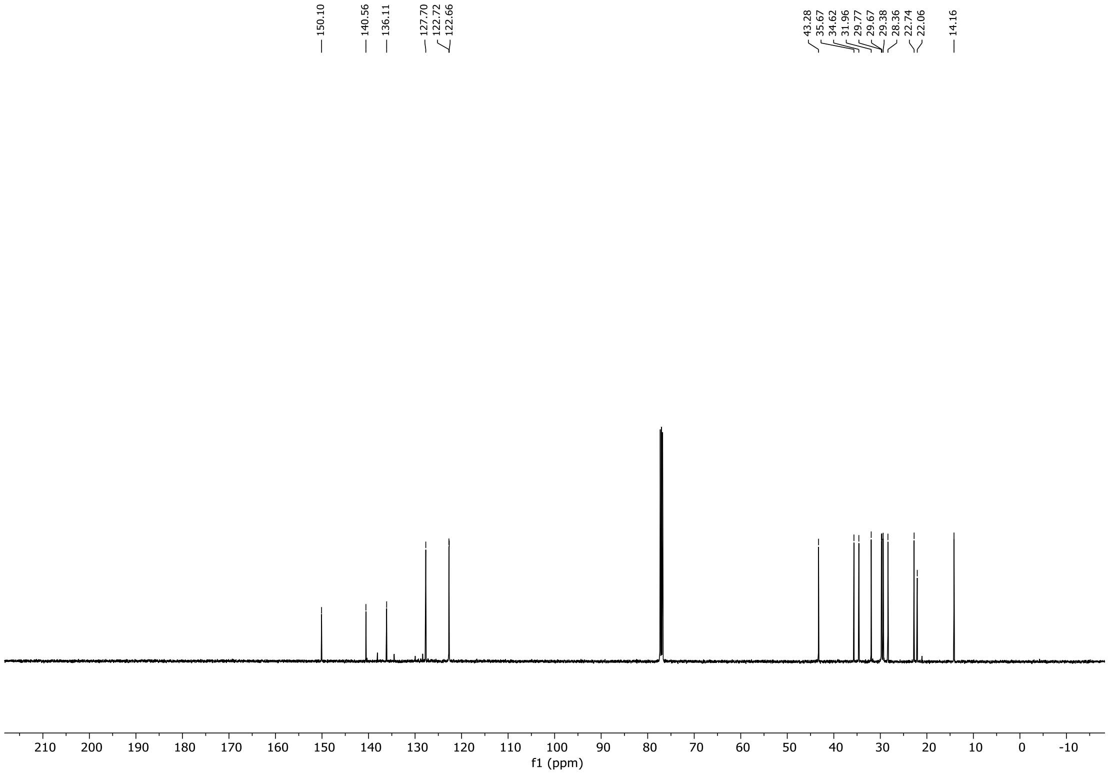


$^{13}C\{^{1}H\}$ NMR (125 MHz, $CDCl_3$) Spectrum of **3x**

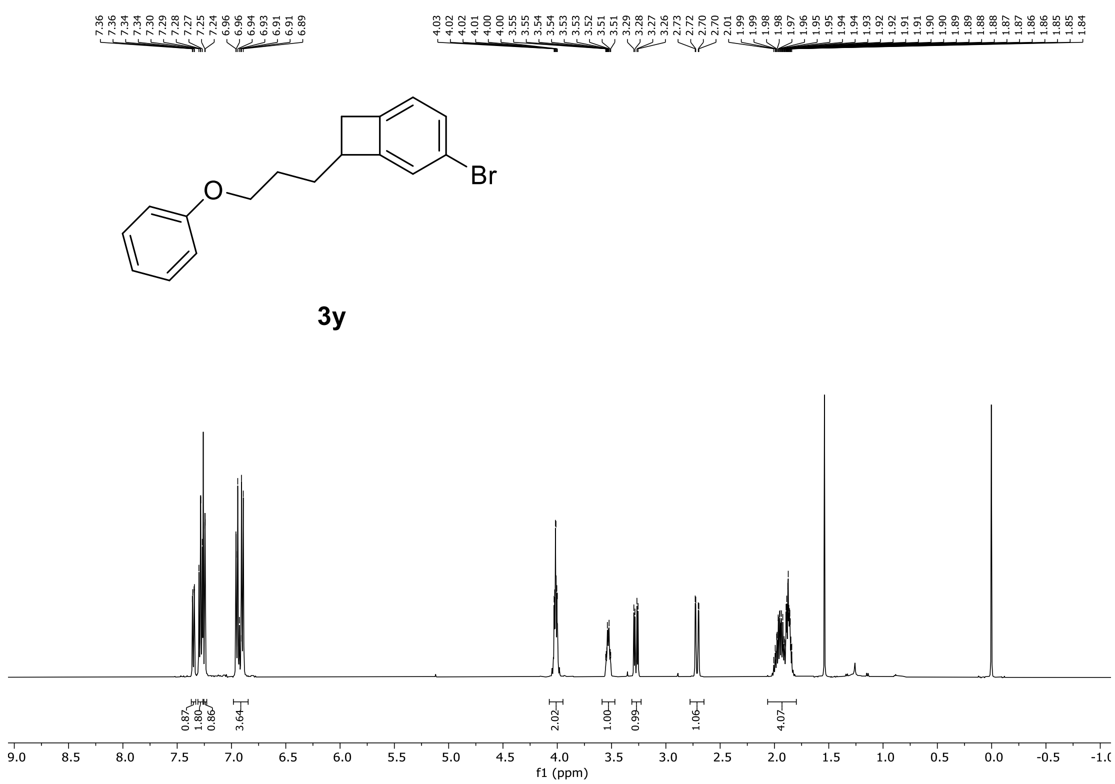


$^{1}$H NMR (500 MHz, CDCl$_3$) Spectrum of **3y**

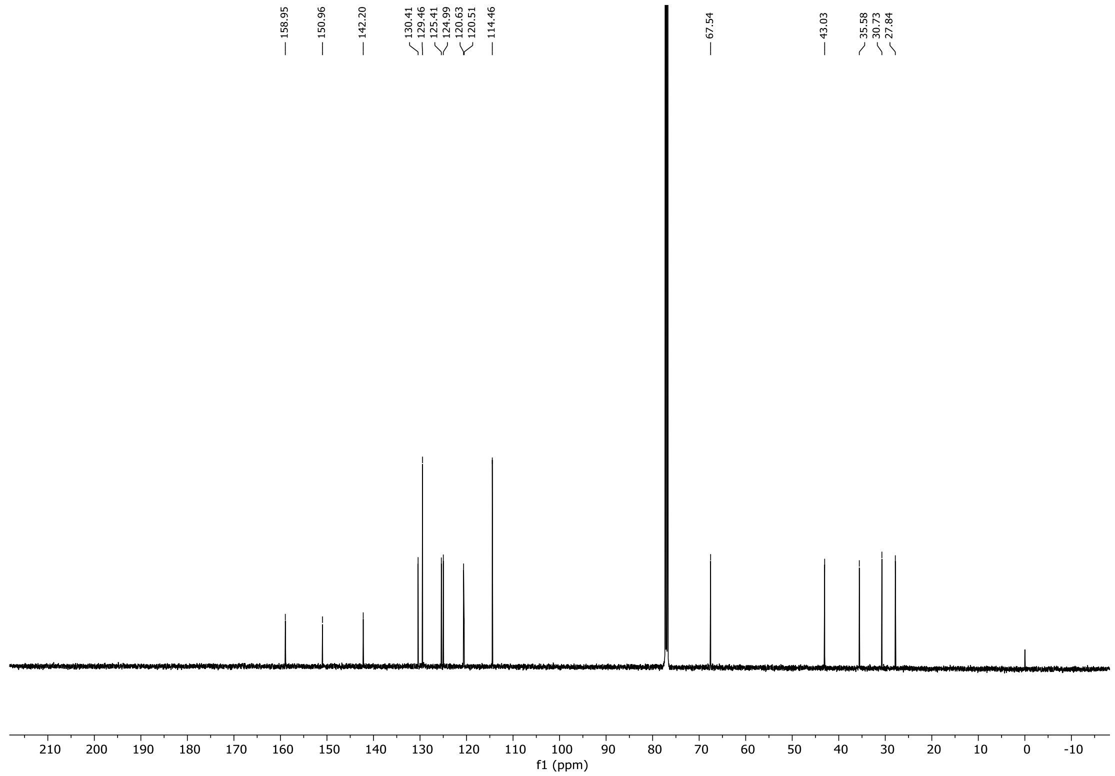


$^{13}$C{$^{1}$H} NMR (125 MHz, CDCl$_3$) Spectrum of **3y**

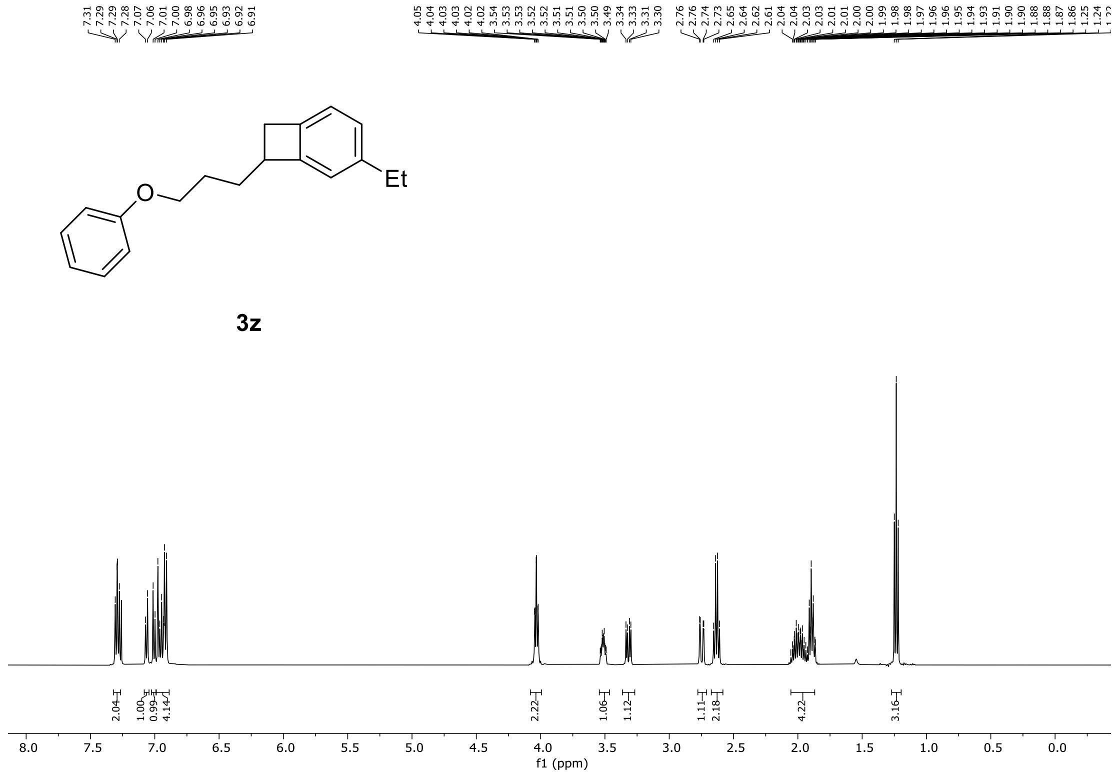


$^{1}$H NMR (500 MHz, CDCl$_3$) Spectrum of **3z**

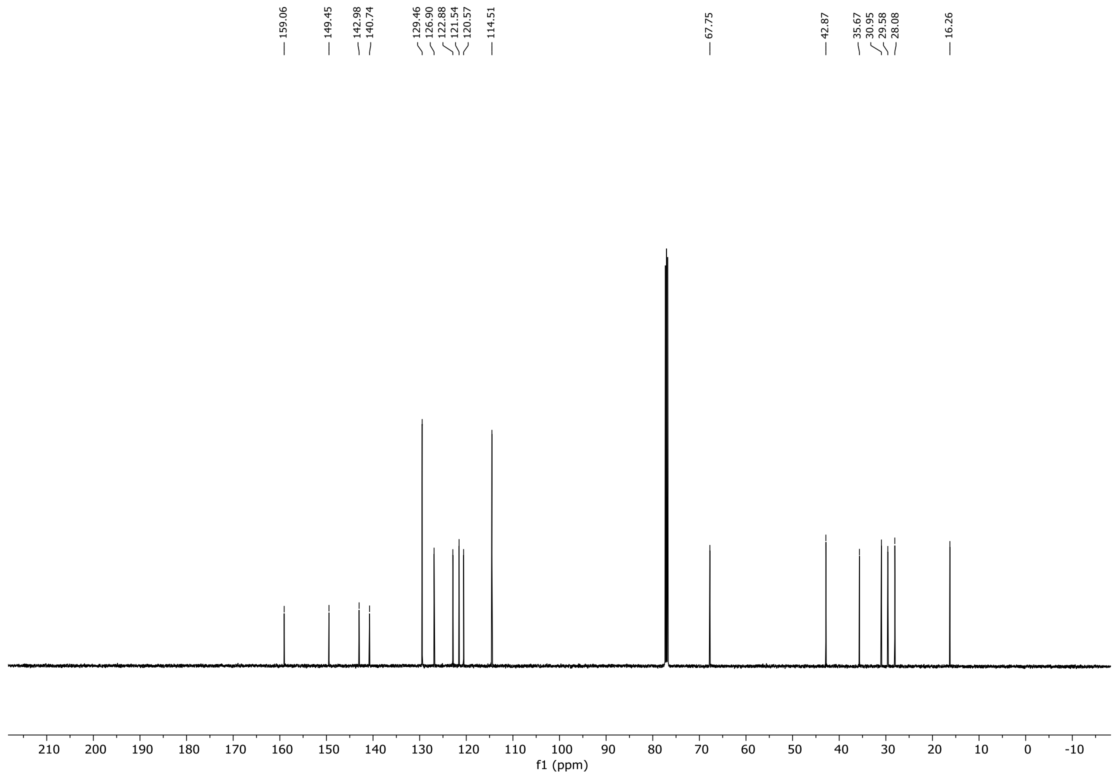


$^{13}$C{$^{1}$H} NMR (125 MHz, CDCl$_3$) Spectrum of **3z**

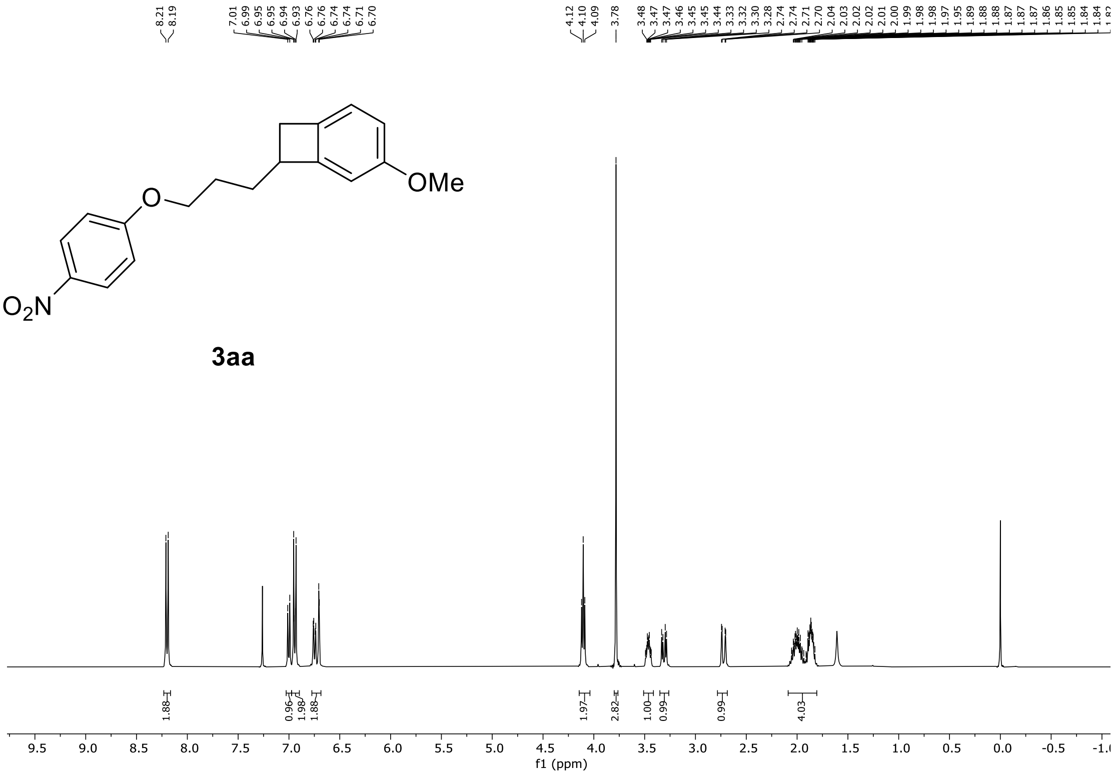


$^1$H NMR (400 MHz, $CDCl_3$) Spectrum of **3aa**

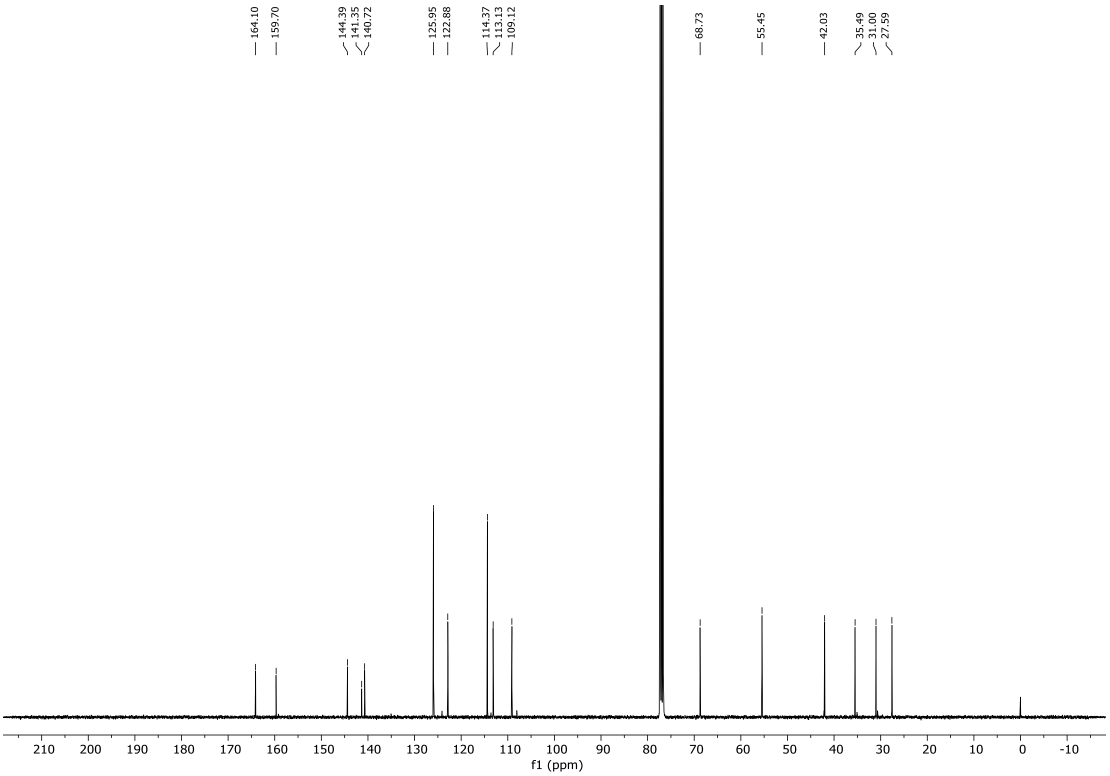


$^{13}C\{^1H\}$ NMR (101 MHz, $CDCl_3$) Spectrum of **3aa**

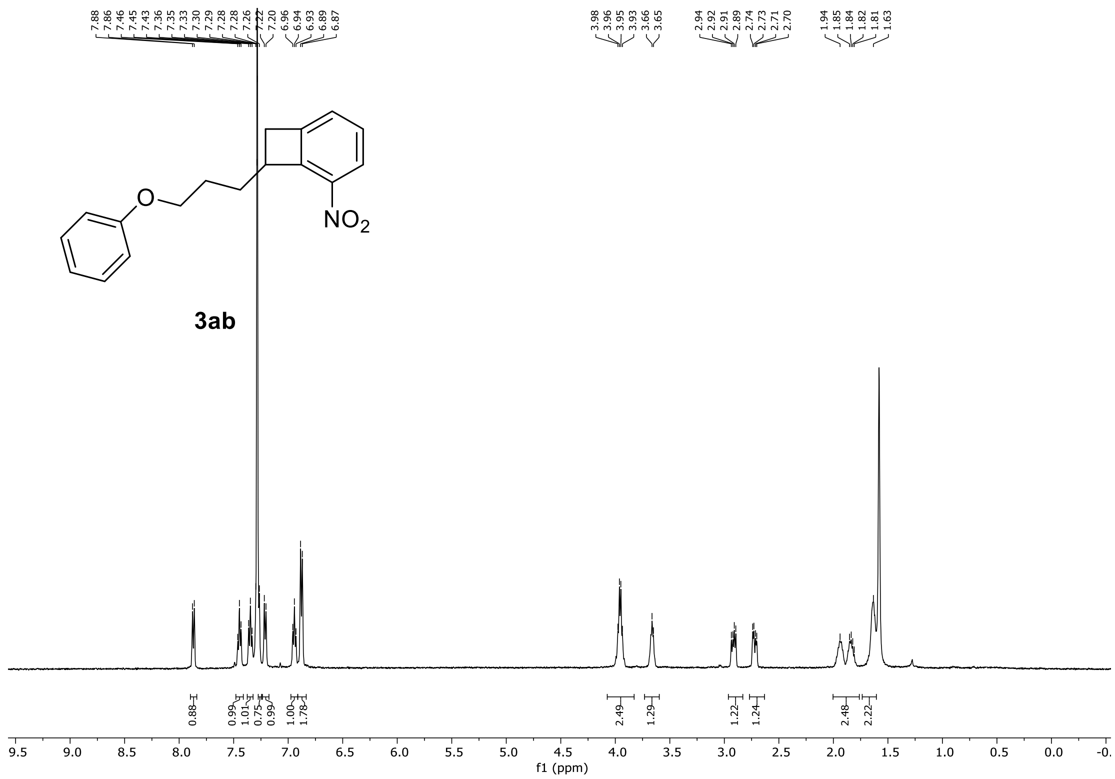

$^{1}$H NMR (500 MHz, $CDCl_3$) Spectrum of **3ab**

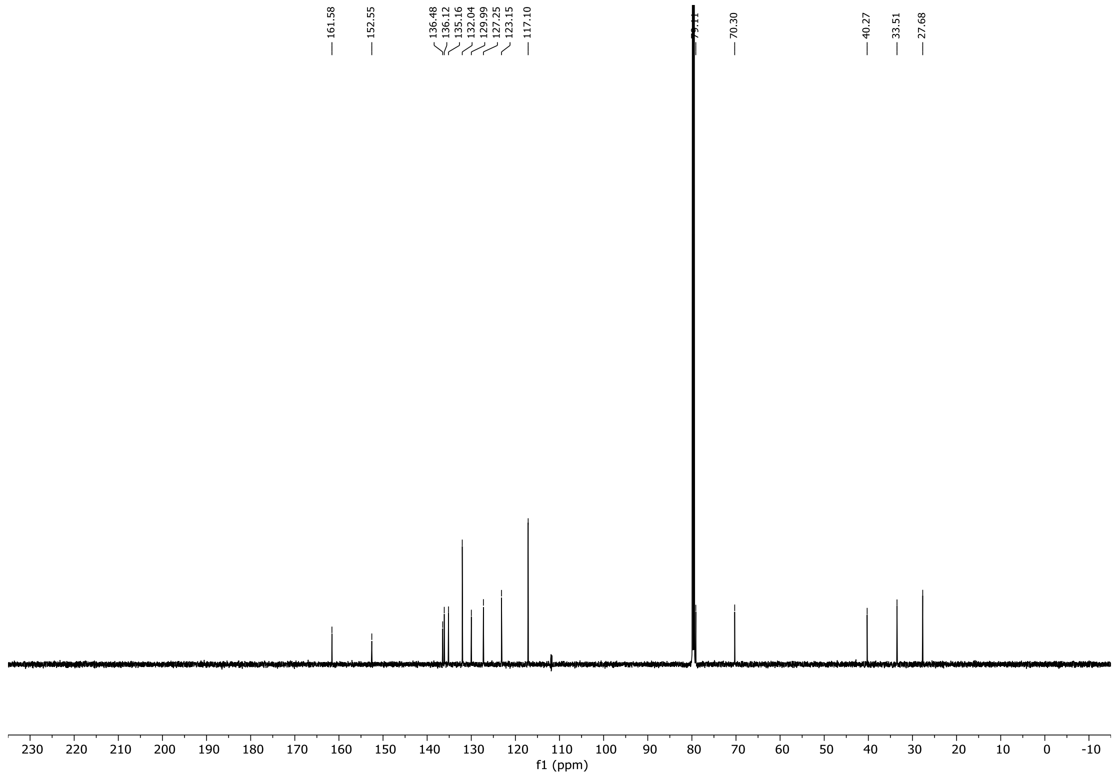

$^{13}$C{$^{1}$H} NMR (151 MHz, $CDCl_3$) Spectrum of **3ab**

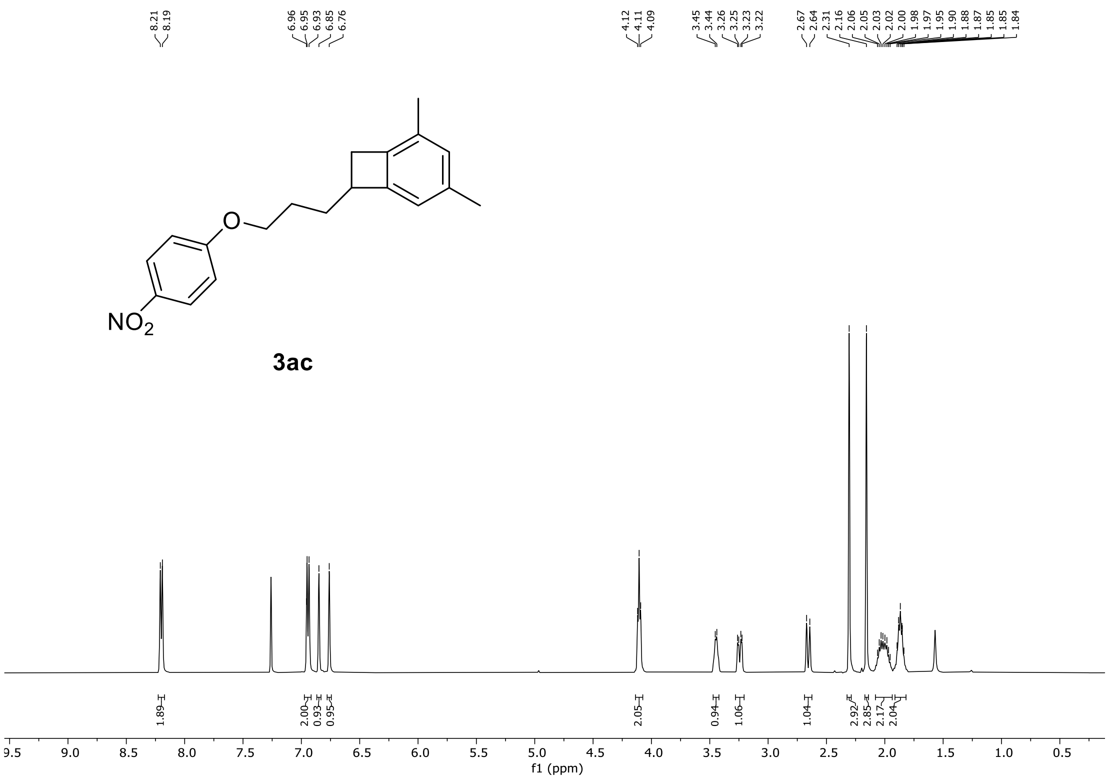

$^1$H NMR (500 MHz, $CDCl_3$) Spectrum of **3ac**

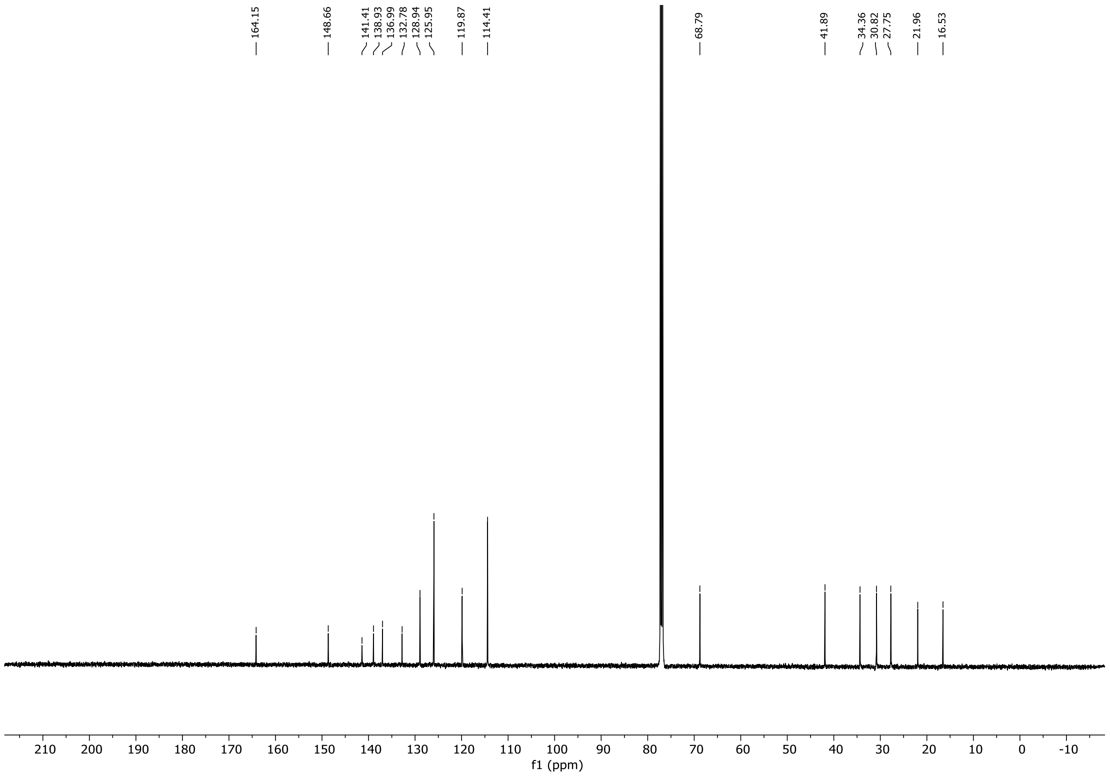

$^{13}$C{$^1$H} NMR (125 MHz, $CDCl_3$) Spectrum of **3ac**

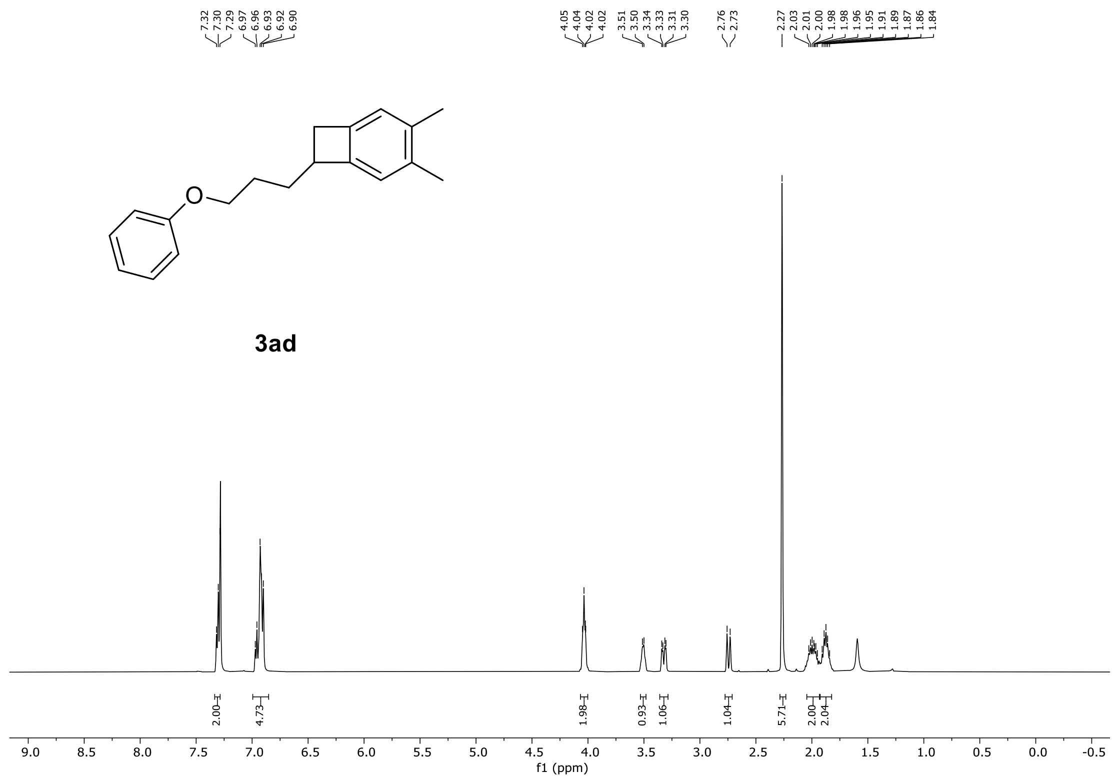


$^{1}$H NMR (500 MHz, $CDCl_3$) Spectrum of **3ad**

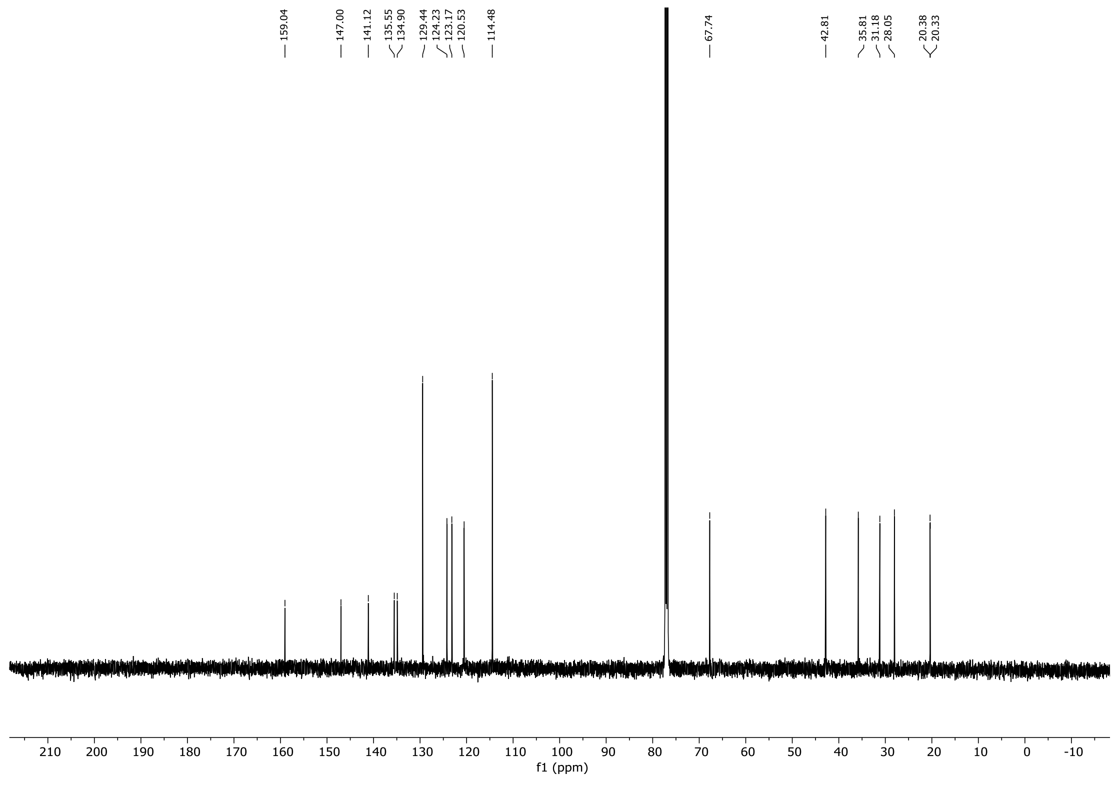


$^{13}$C{$^{1}$H} NMR (125 MHz, $CDCl_3$) Spectrum of **3ad**

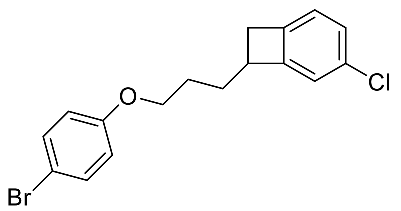


**3ae**

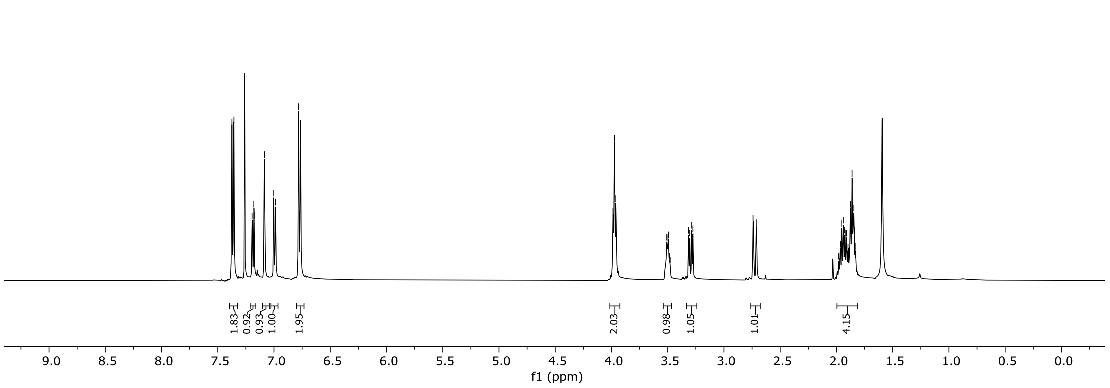

$^{1}$H NMR (500 MHz, CDCl$_3$) Spectrum of **3ae**

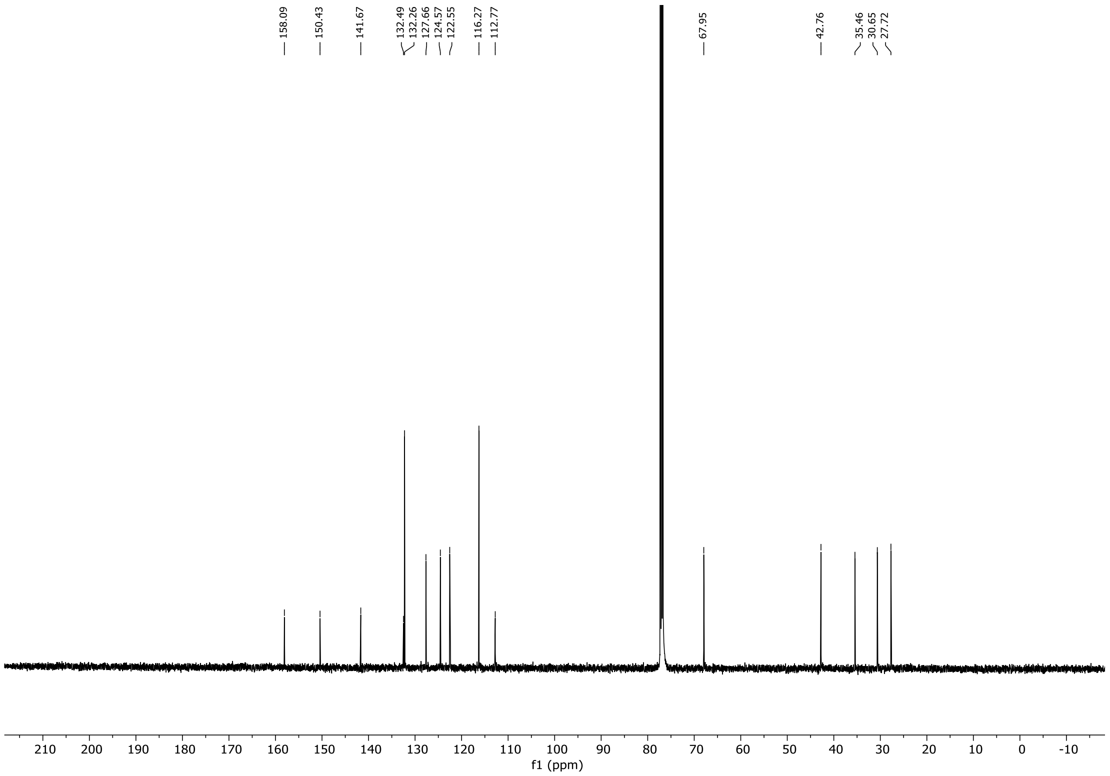

$^{13}$C{$^{1}$H} NMR (125 MHz, CDCl$_3$) Spectrum of **3ae**

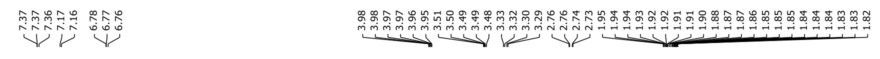


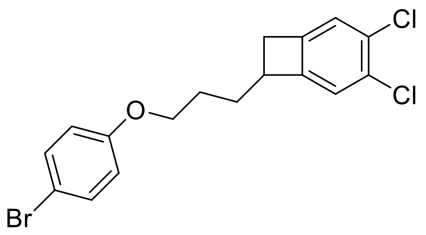


**3af**

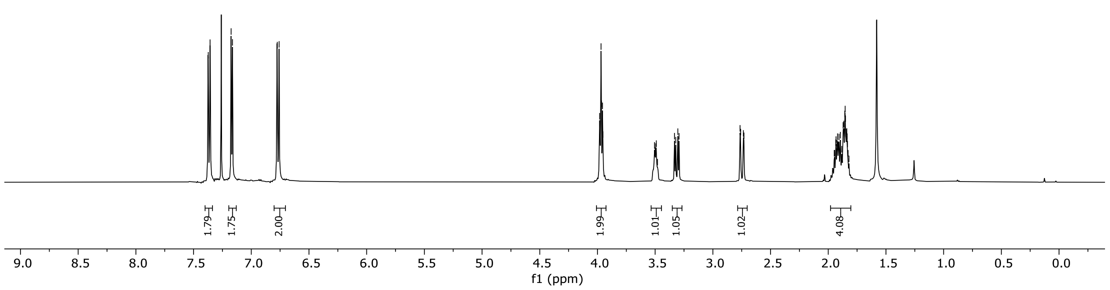


$^{1}$H NMR (500 MHz, $CDCl_3$) Spectrum of **3af**

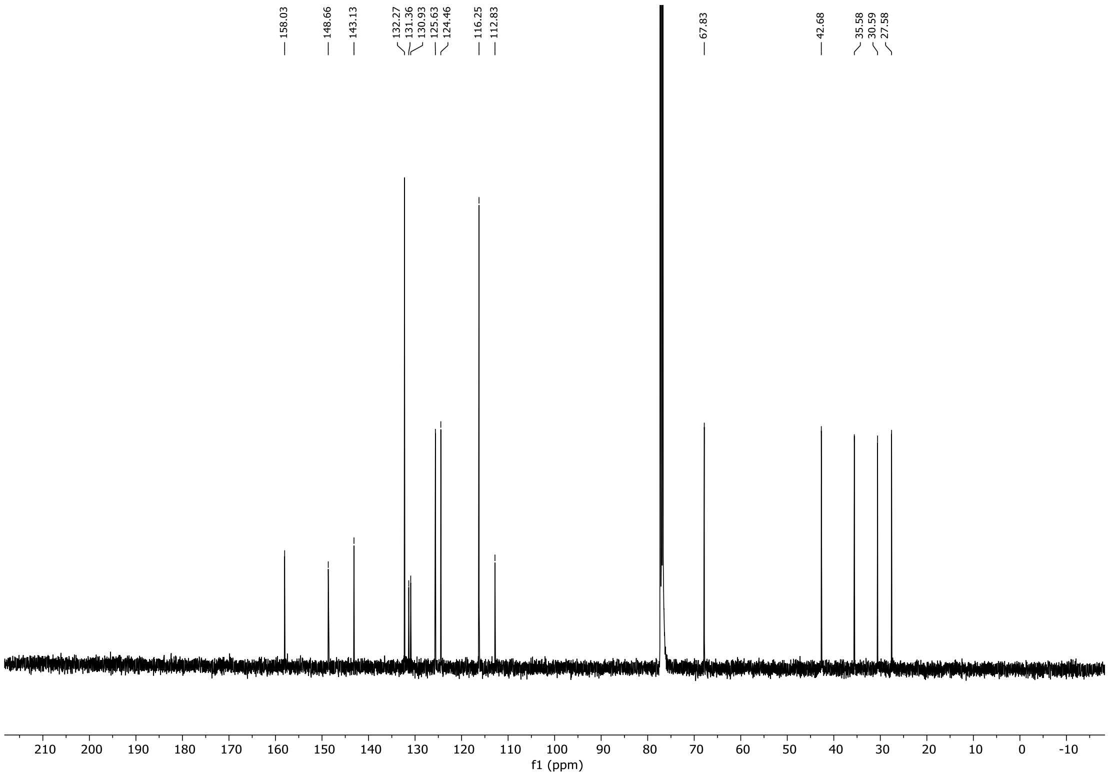


$^{13}$C{$^{1}$H} NMR (125 MHz, $CDCl_3$) Spectrum of **3af**

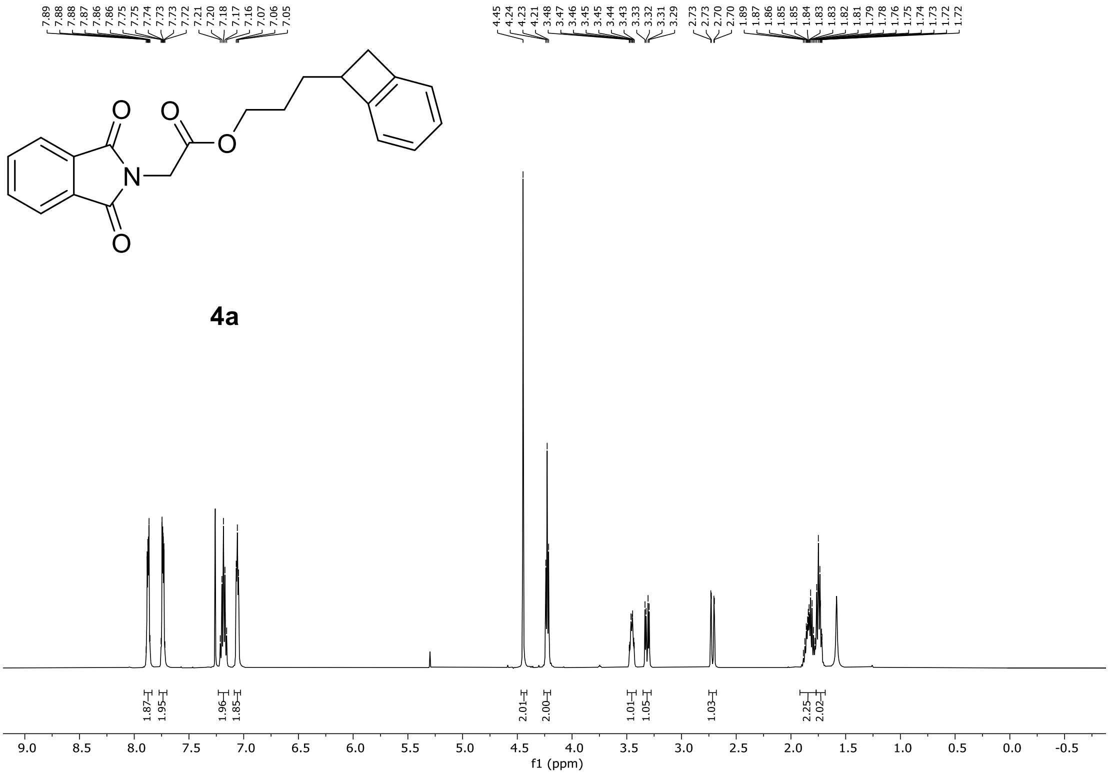


$^{1}H$ NMR (500 MHz, $CDCl_3$) Spectrum of **4a**

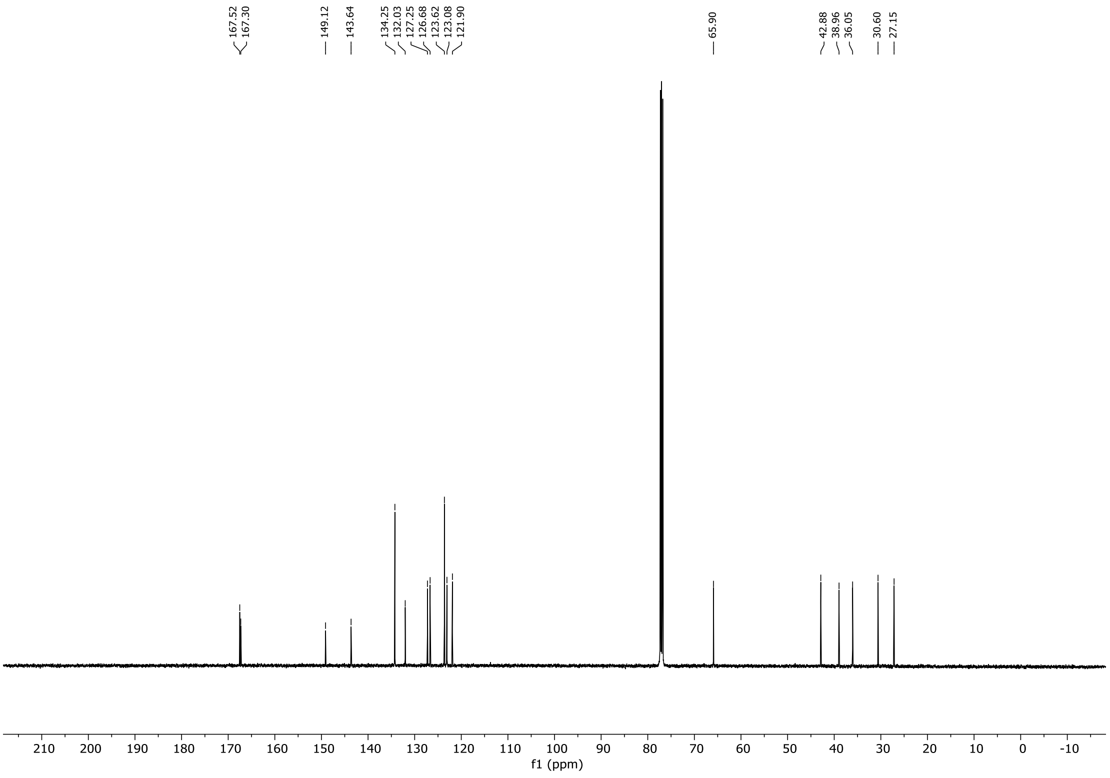


$^{13}C\{^{1}H\}$ NMR (125 MHz, $CDCl_3$) Spectrum of **4a**

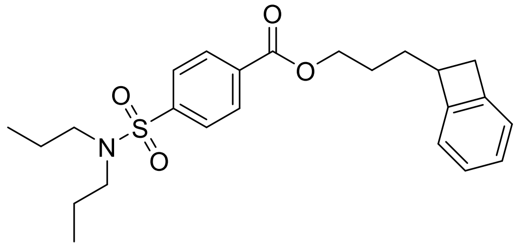


**4b**

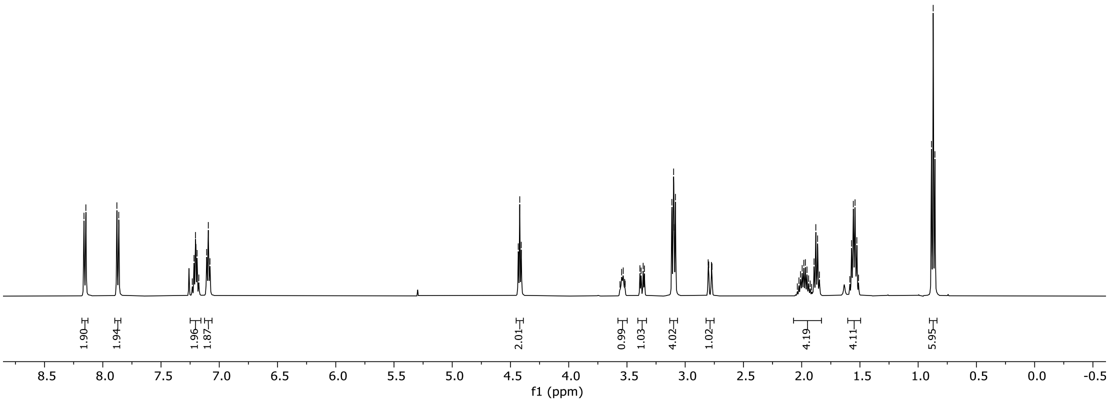


$^{1}H$ NMR (500 MHz, $CDCl_3$) Spectrum of **4b**

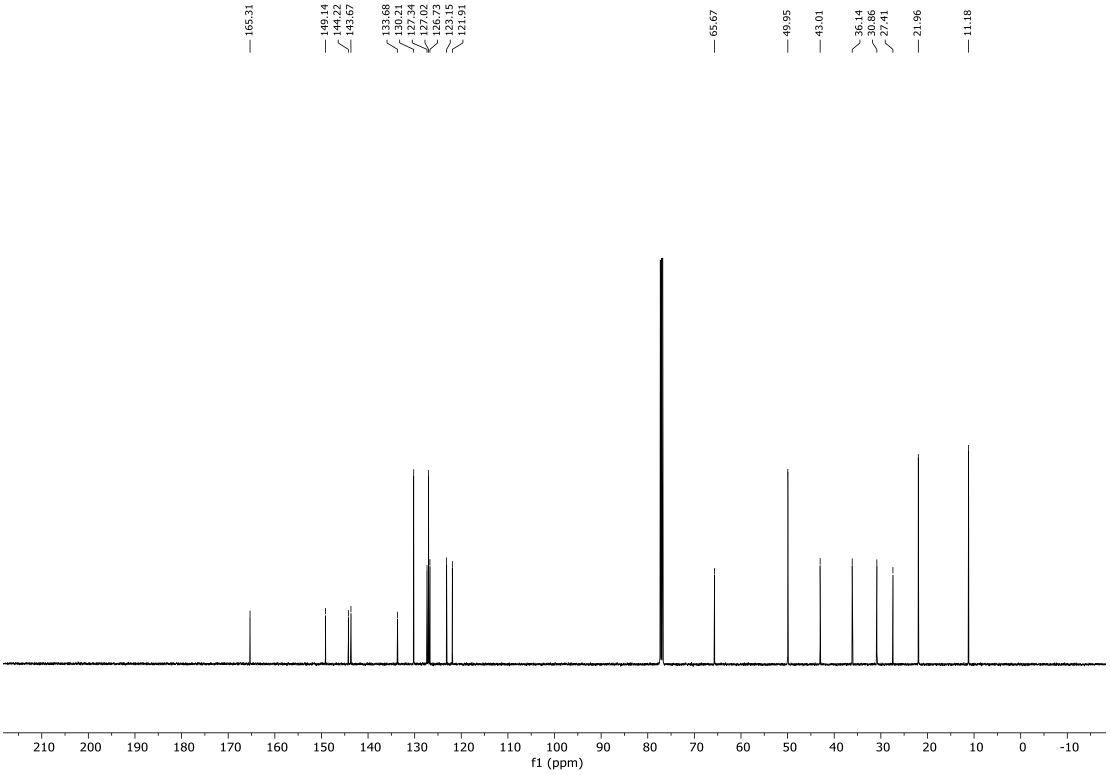


$^{13}C\{^{1}H\}$ NMR (125 MHz, $CDCl_3$) Spectrum of **4b**

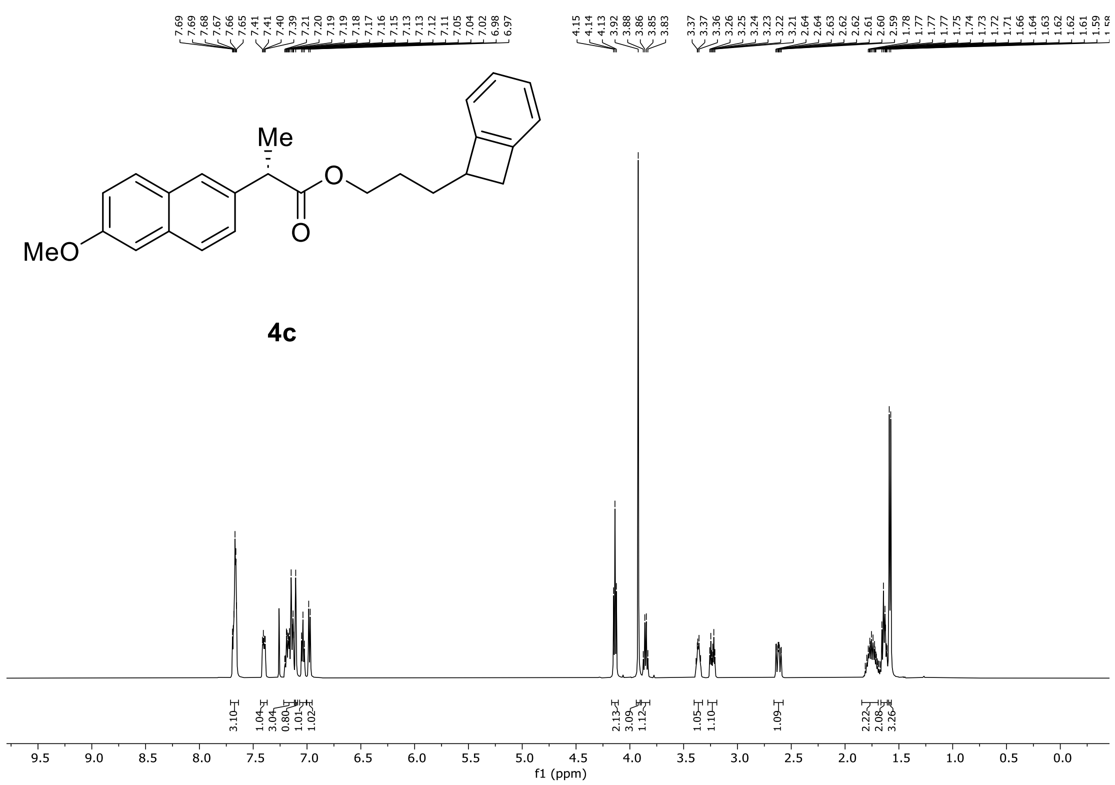


$^{1}H$ NMR (500 MHz, $CDCl_3$) Spectrum of **4c**

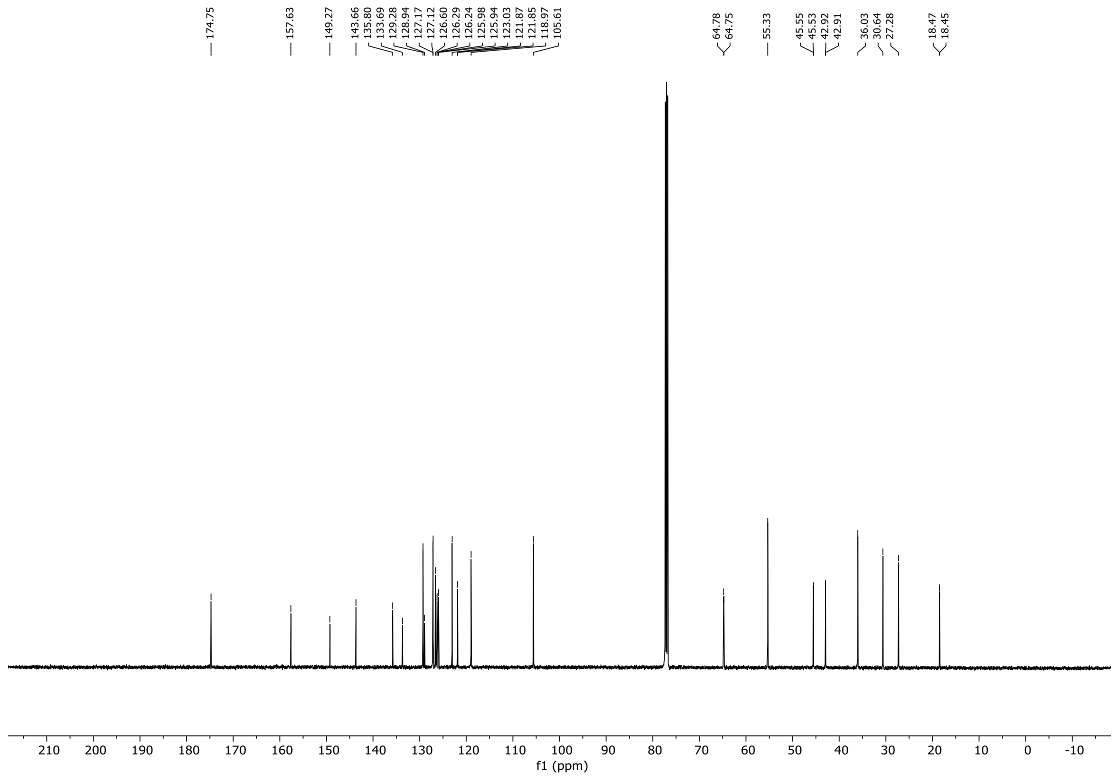


$^{13}C\{^{1}H\}$ NMR (125 MHz, $CDCl_3$) Spectrum of **4c**

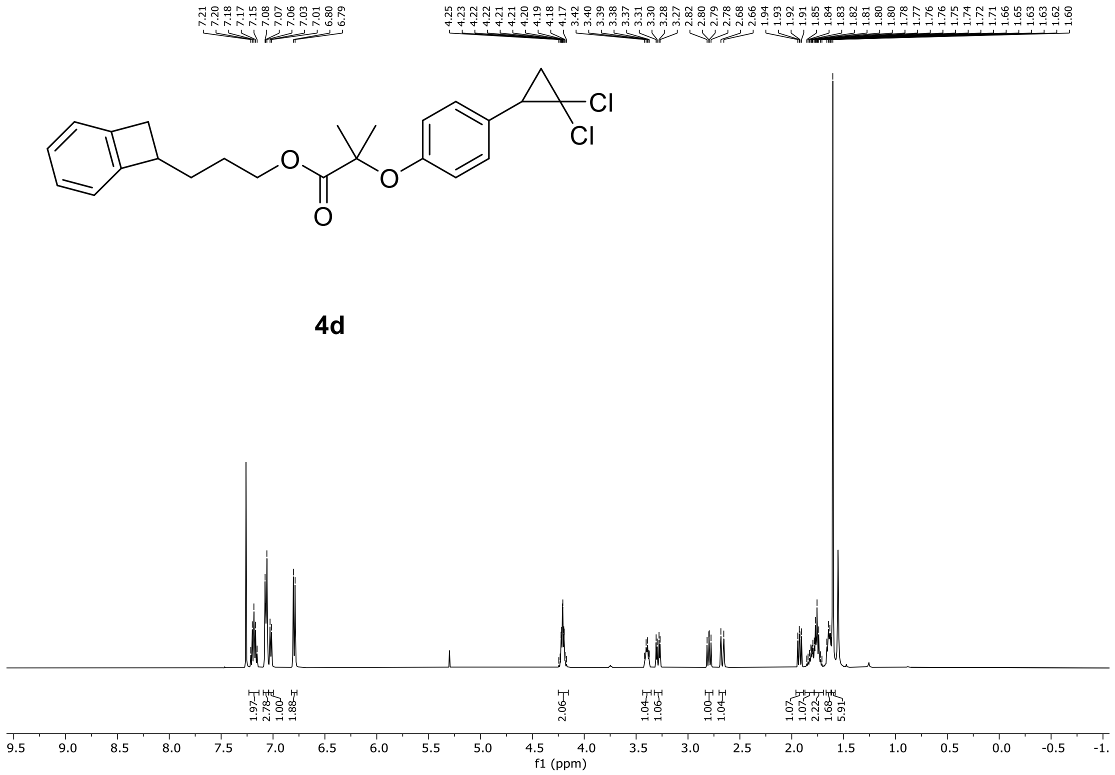


$^{1}H$ NMR (500 MHz, $CDCl_3$) Spectrum of **4d**

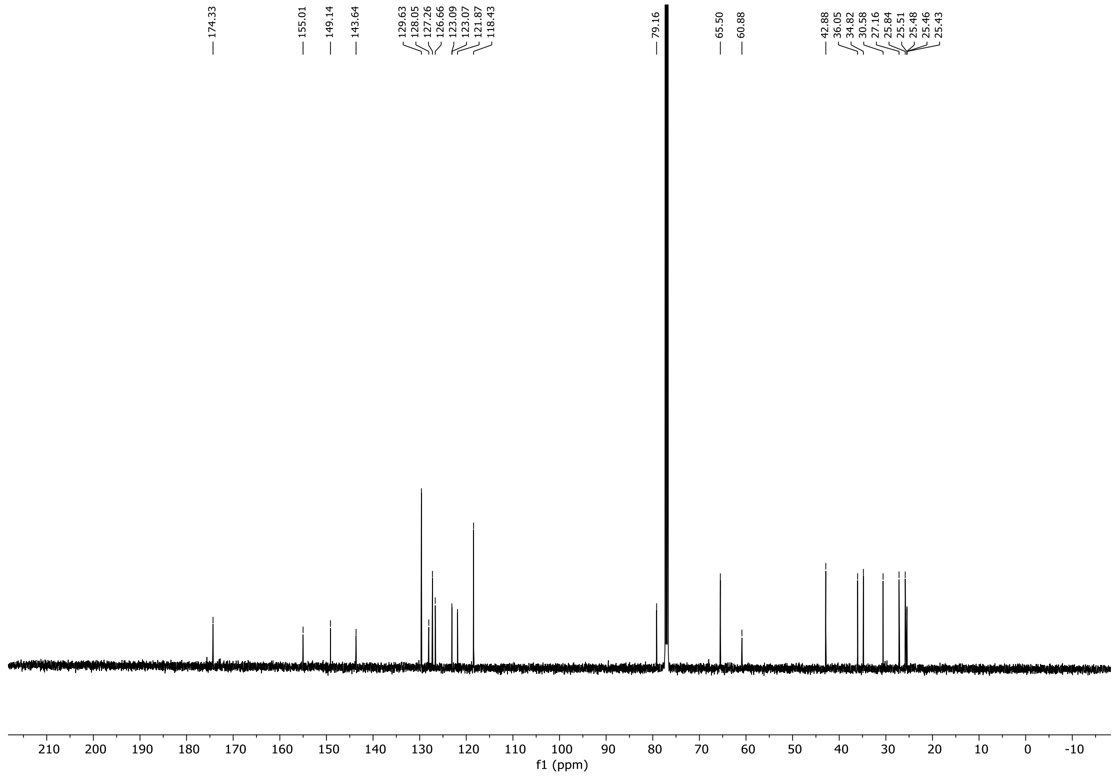


$^{13}C\{^{1}H\}$ NMR (125 MHz, $CDCl_3$) Spectrum of **4d**

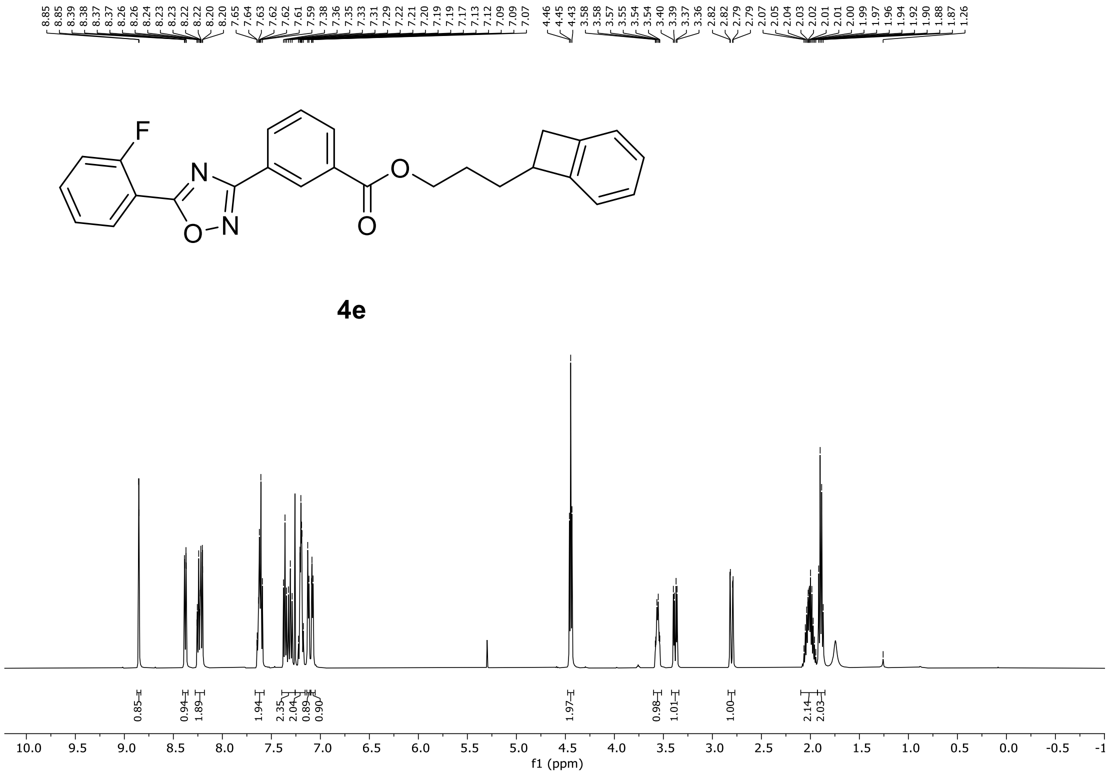


**4e**

$^{1}$H NMR (500 MHz, $CDCl_3$) Spectrum of **4e**

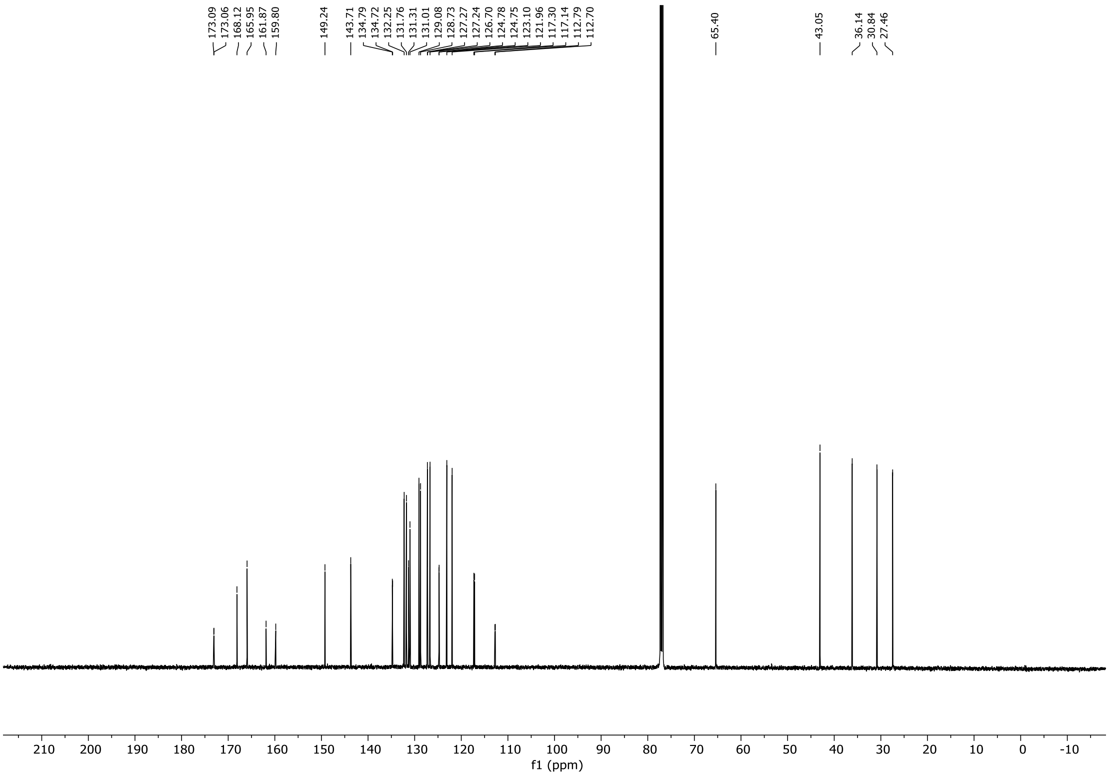


$^{13}$C{[1] H} NMR (125 MHz, $CDCl_3$) Spectrum of **4e**

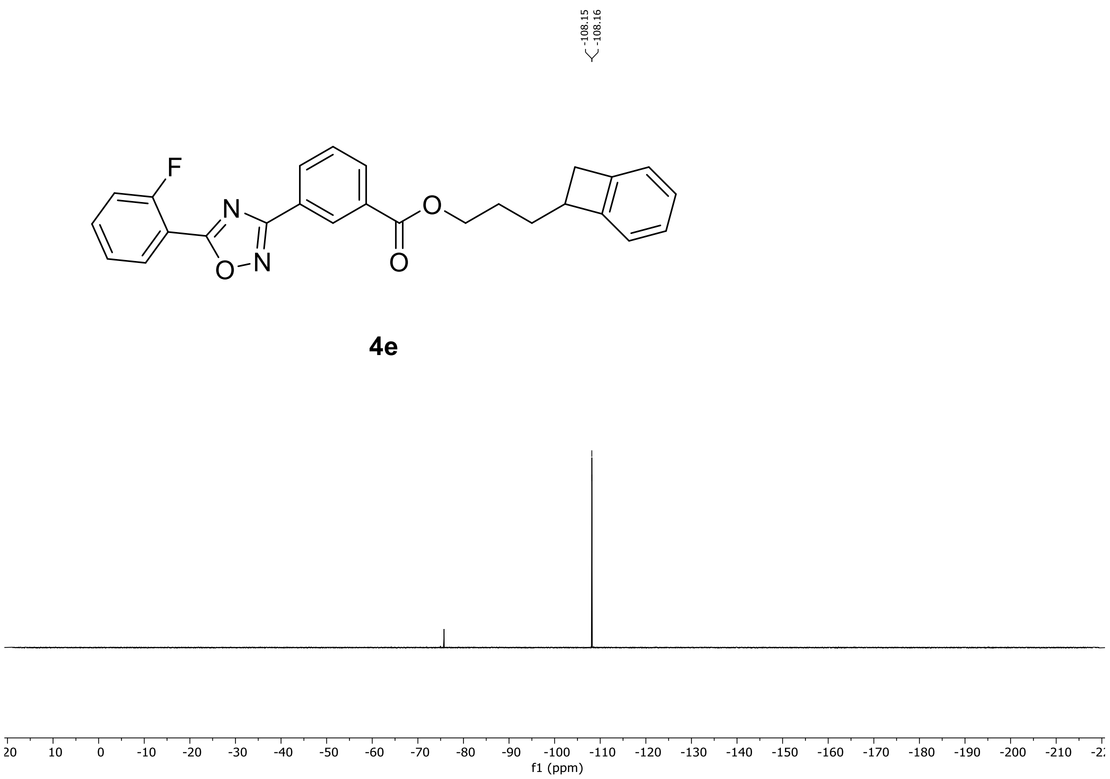


$^{19}$F NMR (471 MHz, $CDCl_3$) Spectrum of **4e**

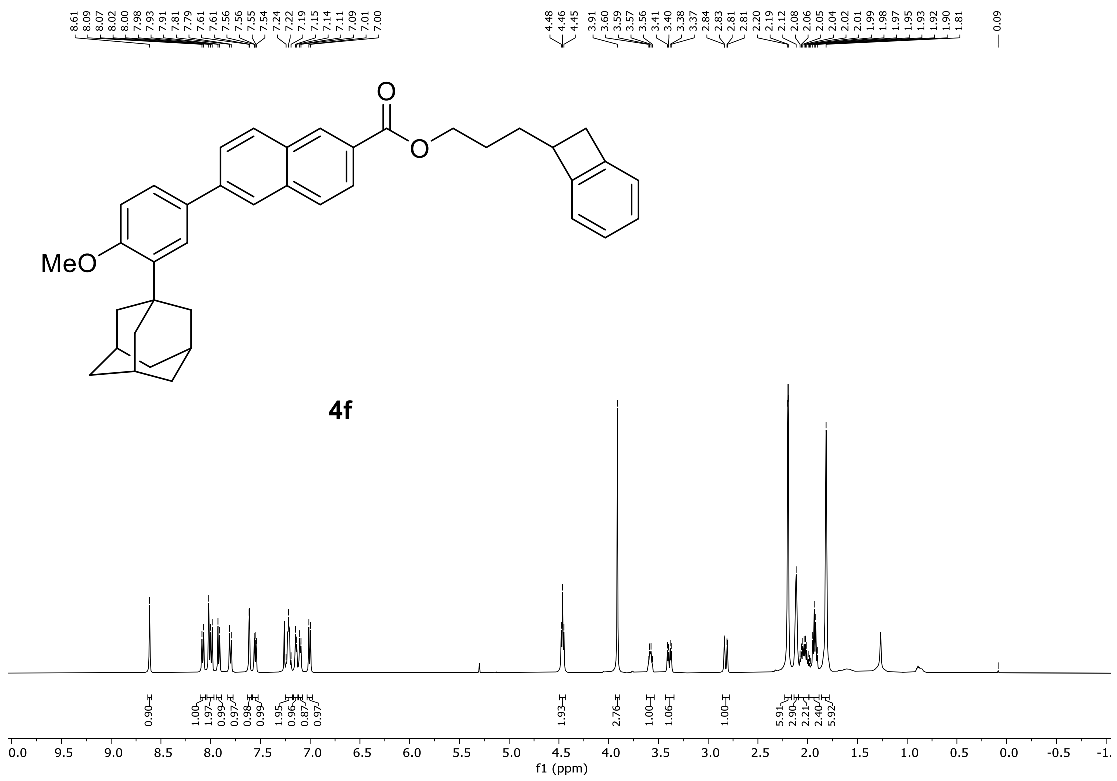


$^{1}$H NMR (500 MHz, $CDCl_3$) Spectrum of **4f**

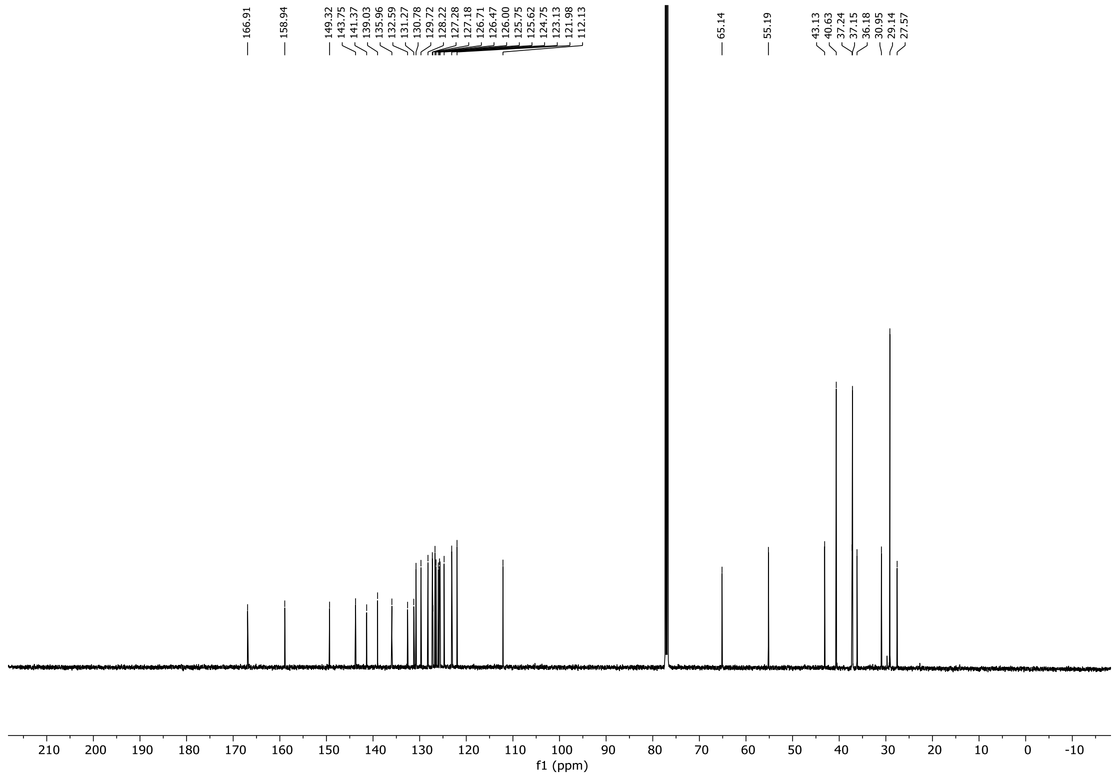


$^{13}C\{^{1}H\}$ NMR (125 MHz, $CDCl_3$) Spectrum of **4f**

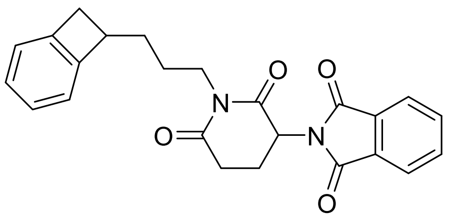

**4g**

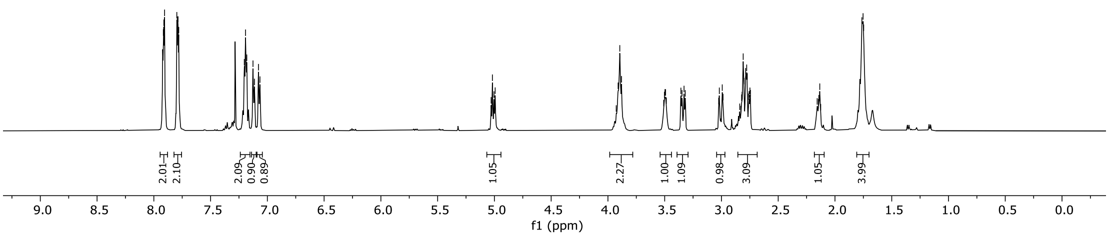


$^{1}H$ NMR (500 MHz, $CDCl_3$) Spectrum of **4g**

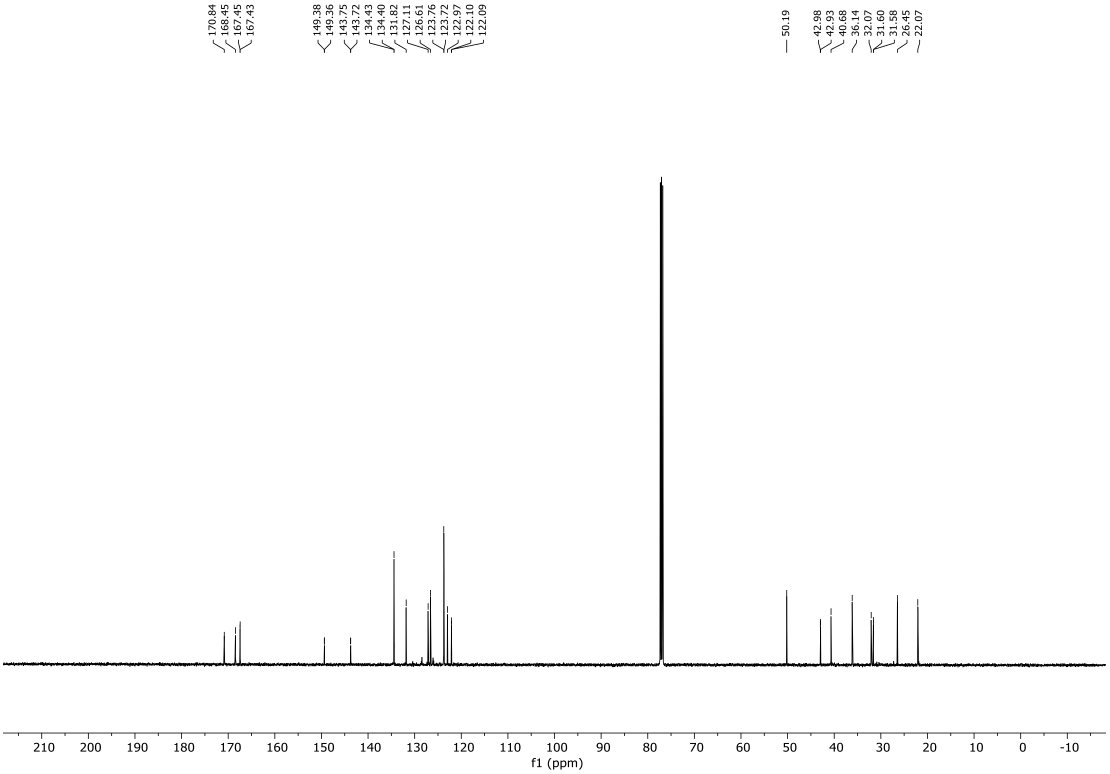


$^{13}C\{^{1}H\}$ NMR (125 MHz, $CDCl_3$) Spectrum of **4g**

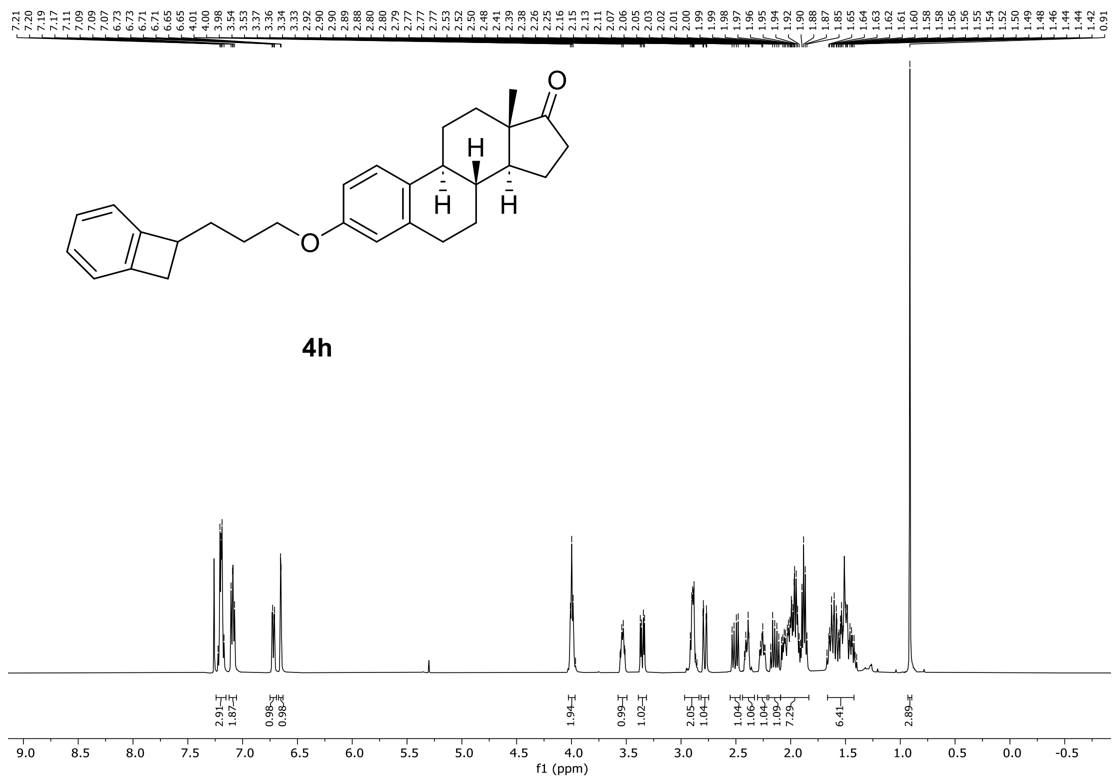

$^{1}H$ NMR (500 MHz, $CDCl_3$) Spectrum of **4h**

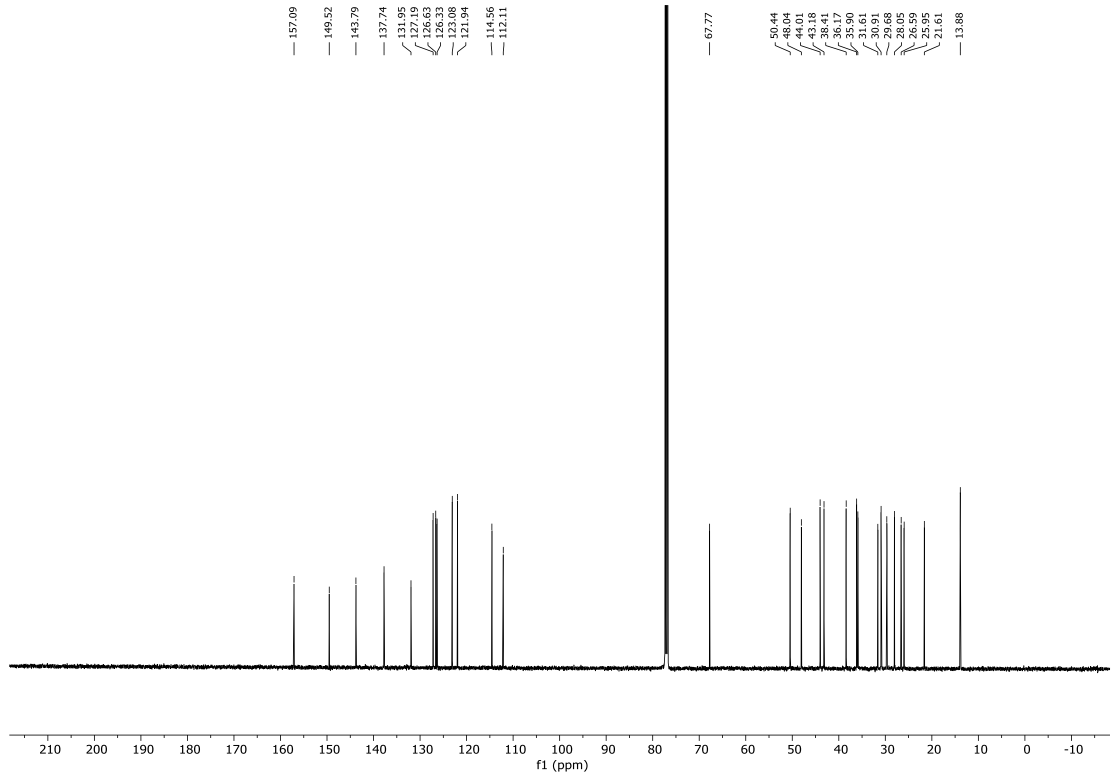

$^{13}C\{^{1}H\}$ NMR (125 MHz, $CDCl_3$) Spectrum of **4h**

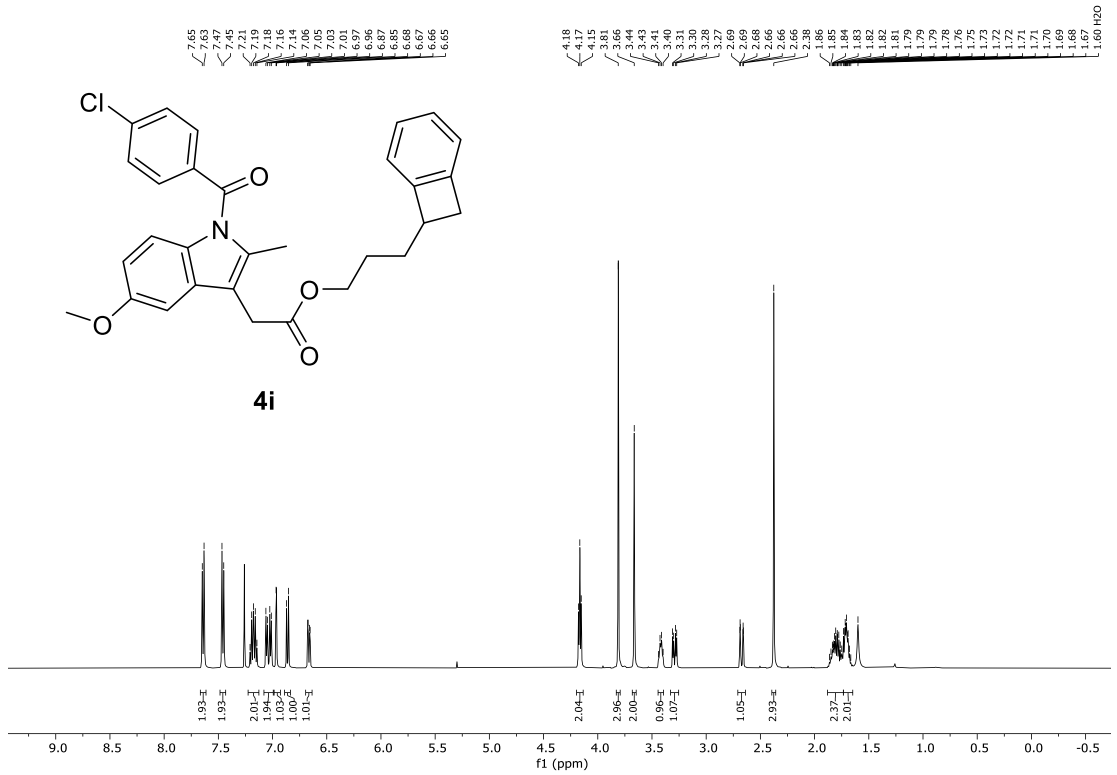


$^{1}$H NMR (500 MHz, $CDCl_3$) Spectrum of **4i**

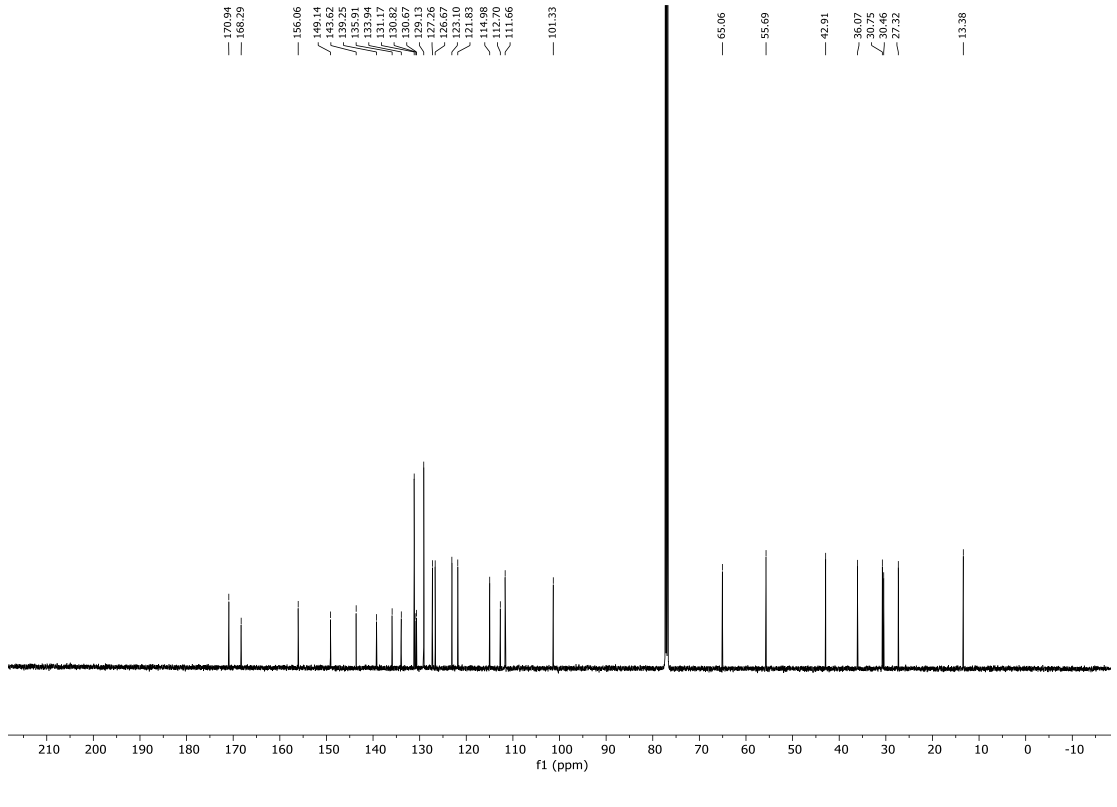


$^{13}C\{^{1}H\}$ NMR (125 MHz, $CDCl_3$) Spectrum of **4i**

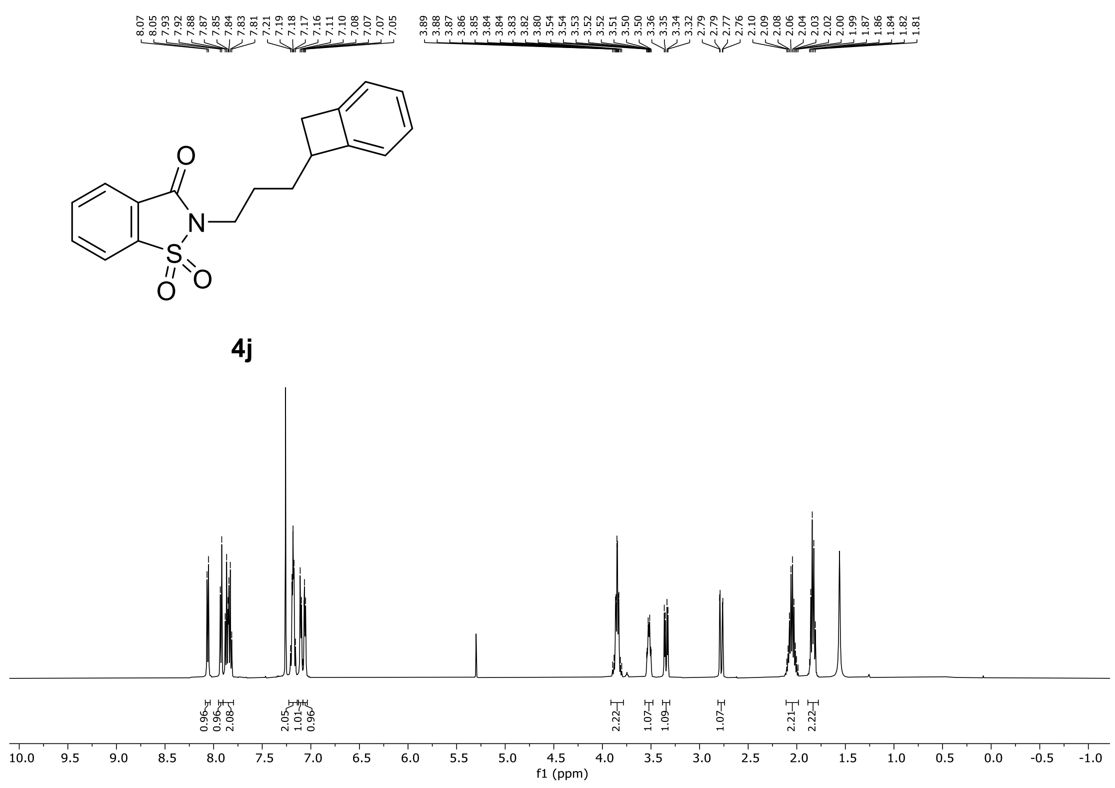


$^{1}$H NMR (500 MHz, $CDCl_3$) Spectrum of **4j**

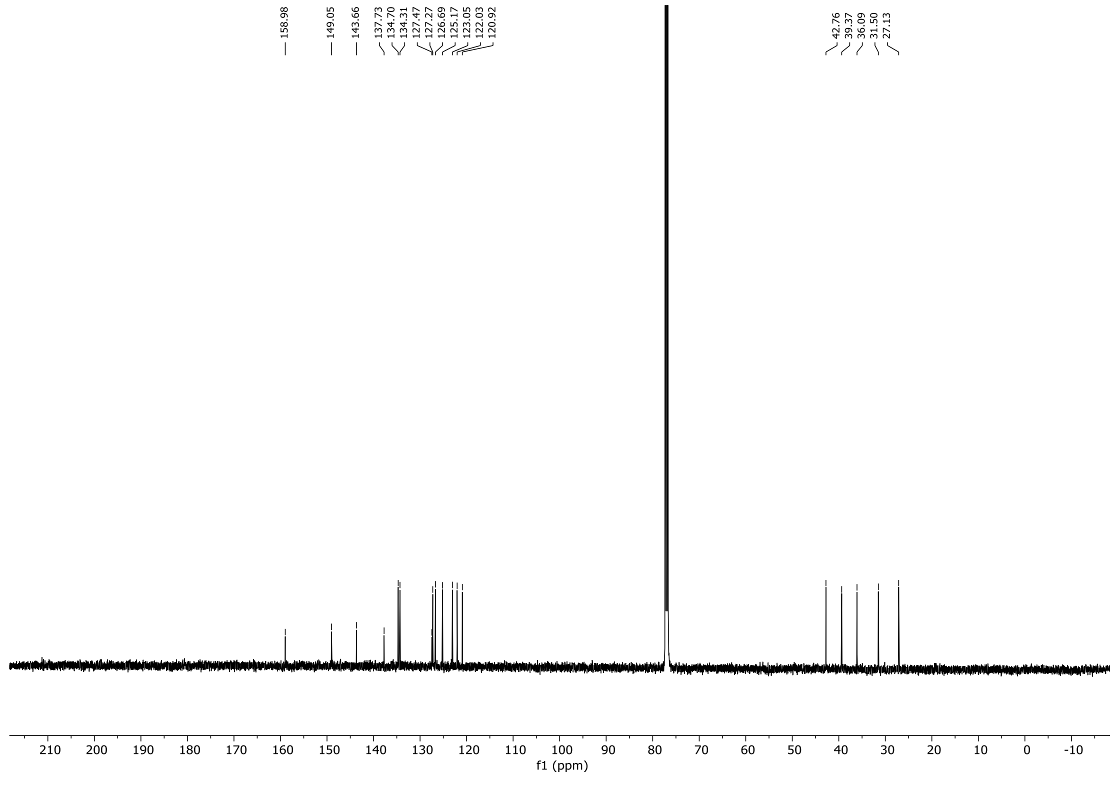


$^{13}$C{$^{1}$H} NMR (125 MHz, $CDCl_3$) Spectrum of **4j**

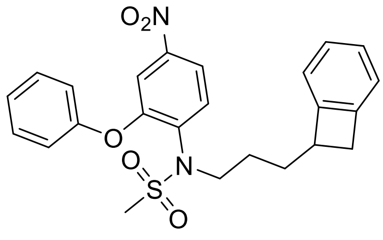


**4k**

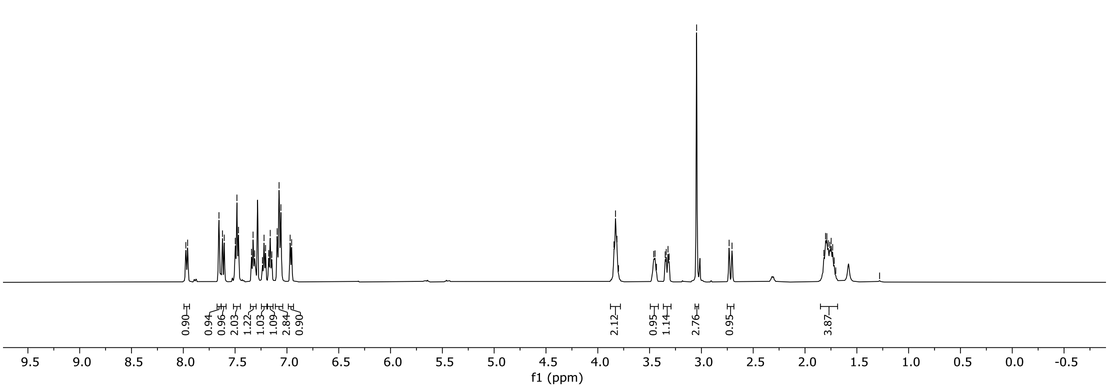


$^{1}$H NMR (500 MHz, $CDCl_3$) Spectrum of **4k**

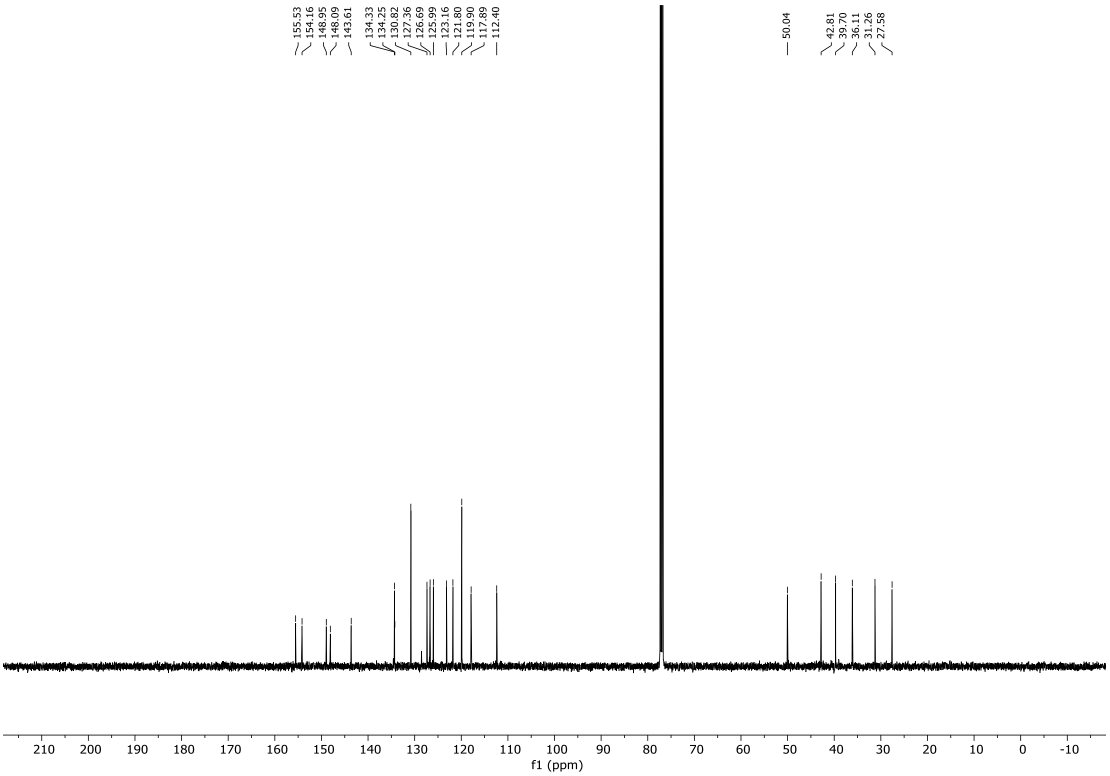


$^{13}$C{[1] H} NMR (125 MHz, $CDCl_3$) Spectrum of **4k**

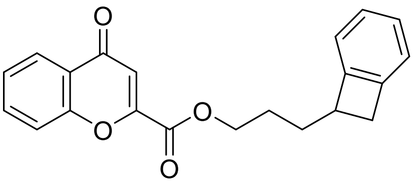

**4l**

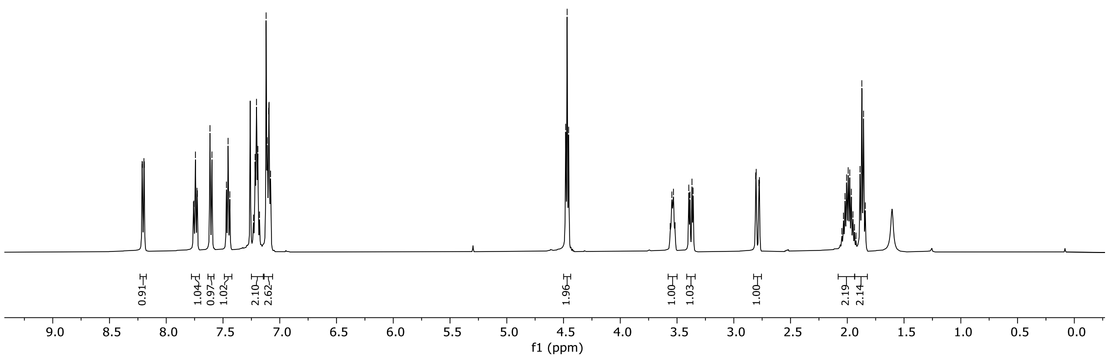


$^{1}$H NMR (500 MHz, $CDCl_3$) Spectrum of **4l**

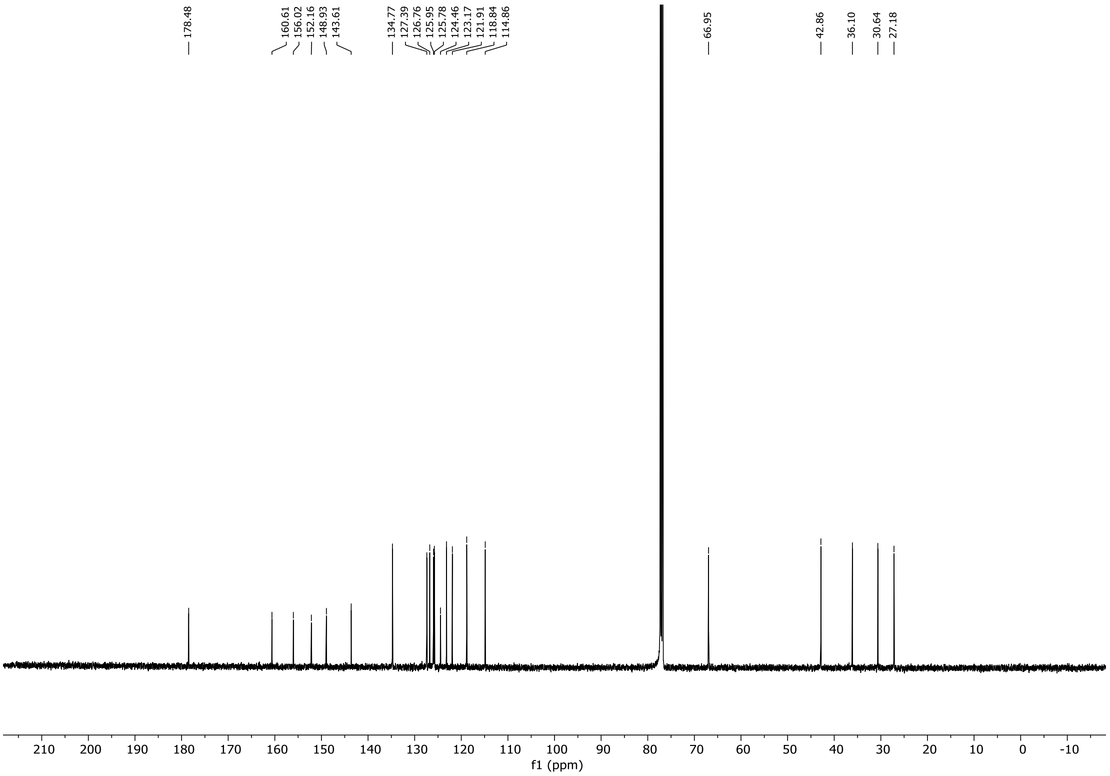


$^{13}C\{^{1}H\}$ NMR (125 MHz, $CDCl_3$) Spectrum of **4l**

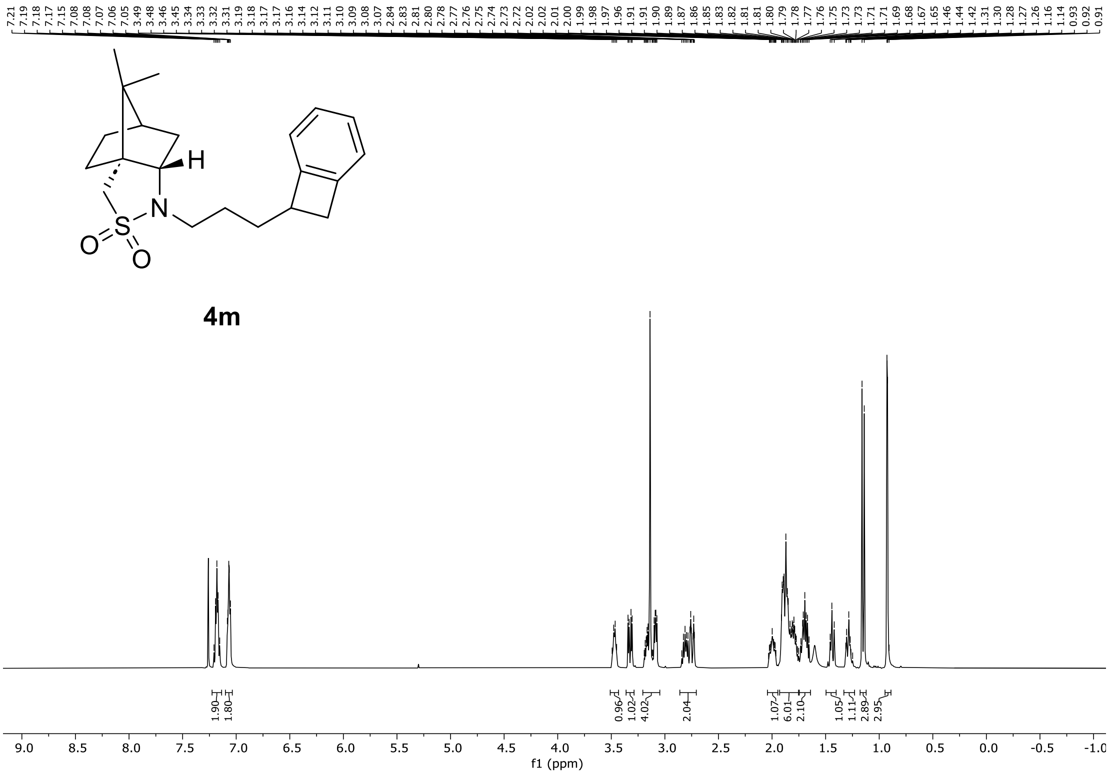


$^{1}$H NMR (500 MHz, $CDCl_3$) Spectrum of **4m**

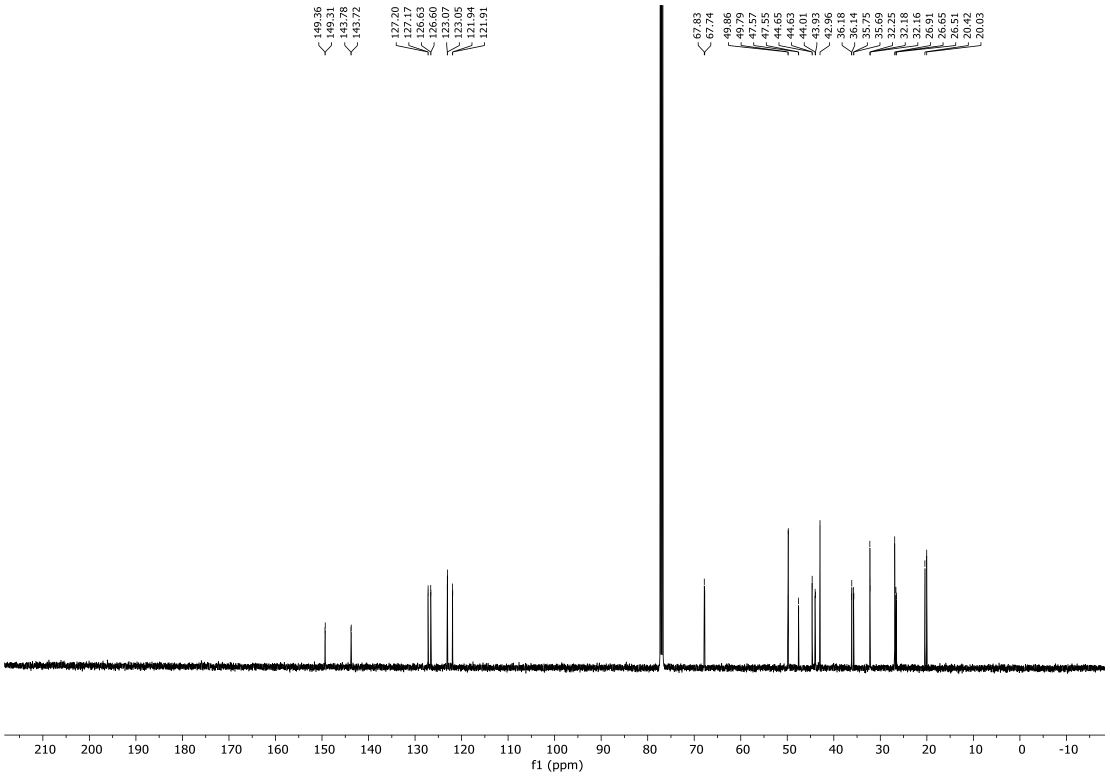


$^{13}C\{^{1}H\}$ NMR (125 MHz, $CDCl_3$) Spectrum of **4m**

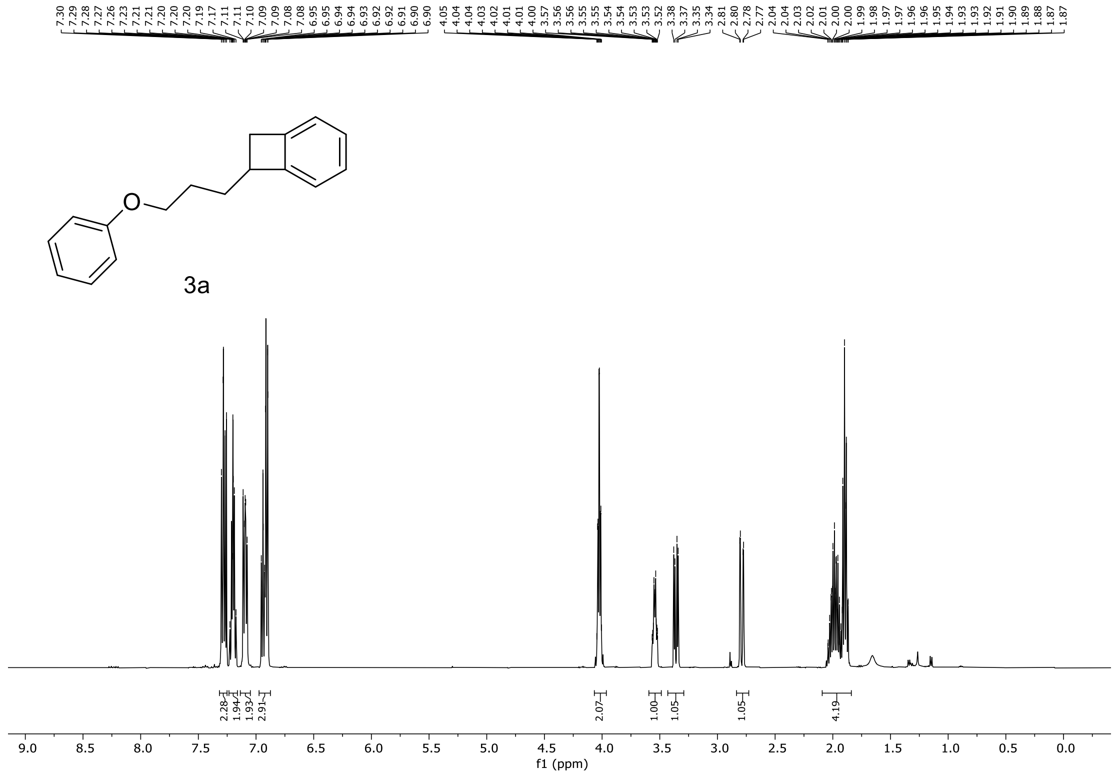


$^{1}$H NMR (500 MHz, $CDCl_3$) Spectrum of **3a** (1 mmol scale)

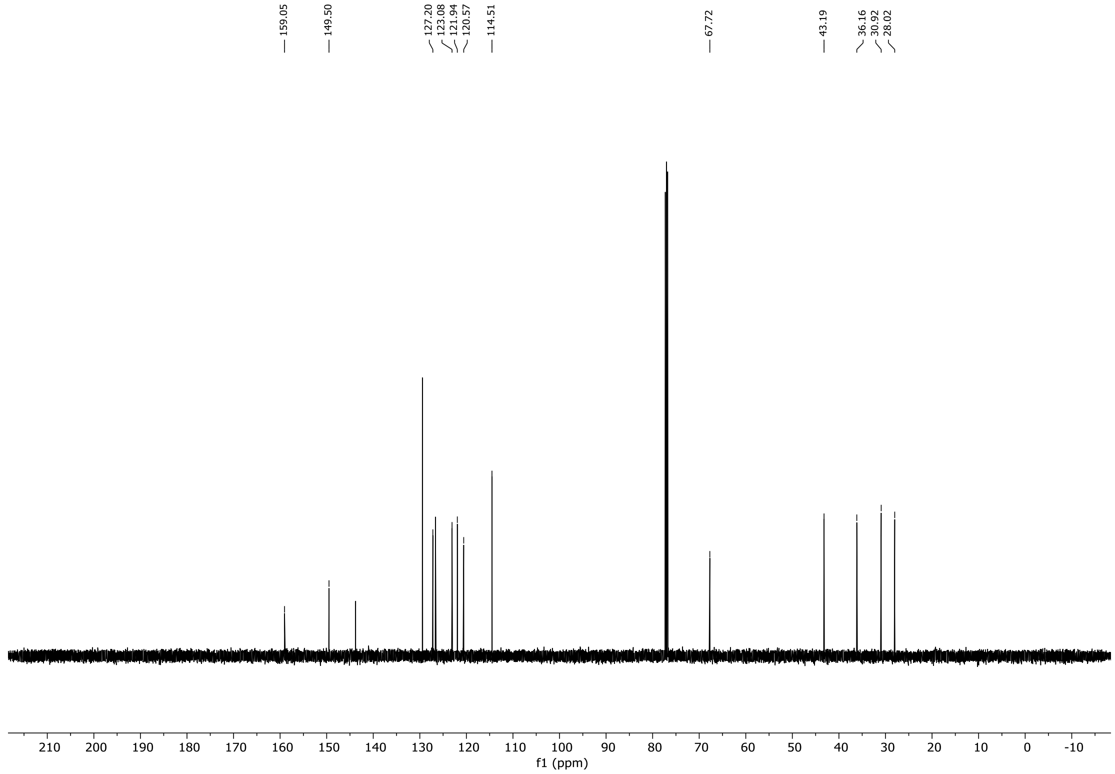


$^{13}$C{$^{1}$H} NMR (125 MHz, $CDCl_3$) Spectrum of **3a** (1 mmol scale)

**Computational Studies**

All DFT calculations were carried out using the Gaussian 09 program suite.[7] Geometry optimizations were performed using the B97D functional[8] with the LANL2DZ effective core potential (ECP) basis set,[9-12] augmented by an *f*-type polarization function for the Au, Ag and I atoms (Au: $\zeta_f = 1.050$, Ag: $\zeta_f = 1.611$, I: $\zeta_d = 0.289$) and the 6-31G(d,p) basis set[13] for all other atoms. The SMD solvation model[14] with dichloroethane as the solvent was applied to account for the solvent effects. Frequency analyses were conducted at the same level of theory to verify that each optimized structure corresponds to either a local minimum (no imaginary frequencies) or a transition state (one imaginary frequency). Intrinsic reaction coordinate (IRC) calculations[15] were conducted to confirm the correct connectivity between transition states and their corresponding reactants and product species. The relative Gibbs energy of each structure was refined by performing single-point energy calculations at the B97D/LANL2DZ-6-311++G(d,p) level of theory in the dichloroethane solvent, based on the optimized geometries. To reduce the overestimation of entropy contribution to Gibbs free energies, an entropy correction of −4.3 (or 4.3) kcal/mol was applied for 2:1 (or 1:2) transformations.[16] Natural bond orbital analyses[17-19] were performed using the NBO 3.1 version contained in Gaussian 09. The non-covalent interaction (NCI) analyses were conducted using the independent gradient model based on Hirshfeld partition (IGMH) within the Multiwfn 3.8 program,[20,21] and the isosurfaces were visualized using VMD 1.9.3 program.[22] The key 3D transition states were generated using CYLview.[23]

## 1. Au(I)-Catalyzed Annulation of Iodobenzene (2a) with Olefin (1a) with the L8 Ligand.

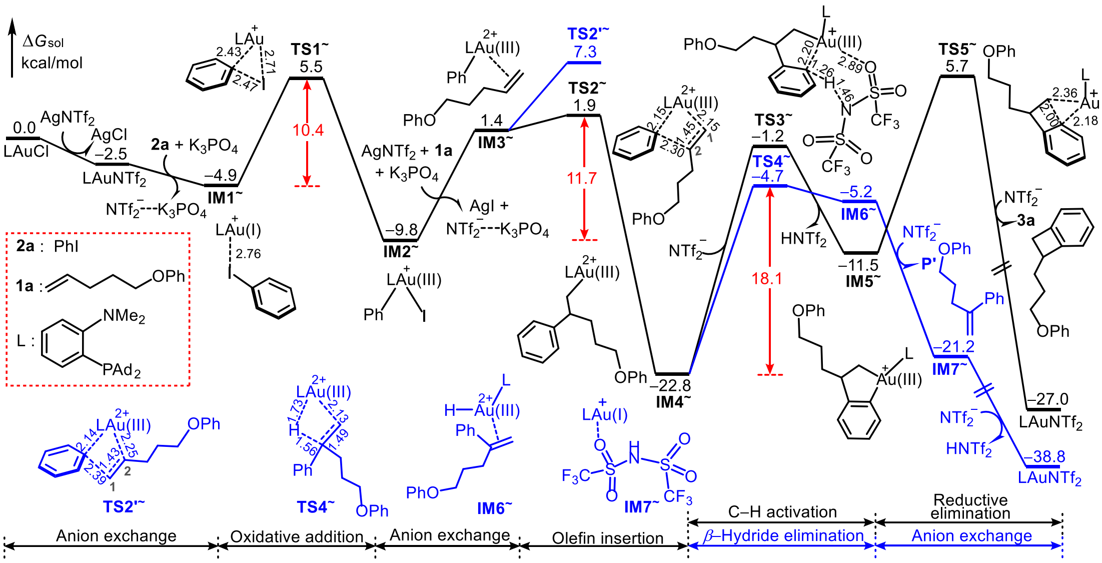

**Fig. S3.** Calculated Gibbs free energy profile for the Au(I)-catalyzed annulation of iodobenzene (**2a**) with olefin (**1a**) with the L8 ligand. Bond distances are given in Å.

## 2. Calculated NPA Charges for LAuCl.

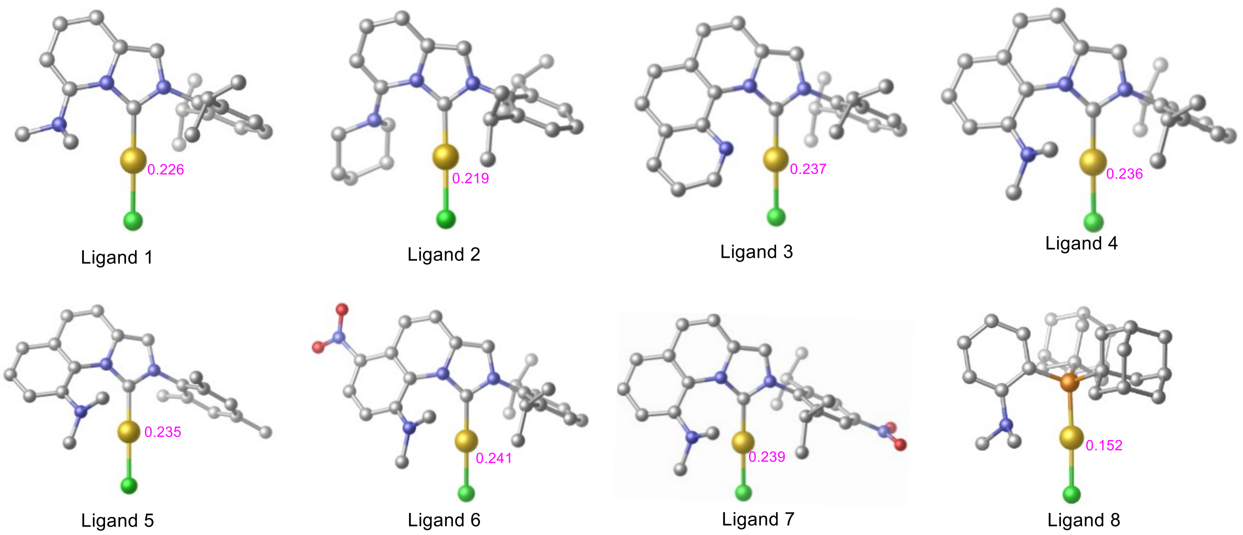


**Fig. S4.** Natural population analysis (NPA) charges derived from Natural Bond Orbital (NBO) analyses (in *e*) on the Au centers of **L1AuCl** – **L8AuCl**.

## 3. Calculated Thermodynamic Data

| **Stationary point** | **Thermal correction to Gibbs free energy at B3LYP-D3/LANL2DZ /6-31G(d,p) (in a.u.) (1)** | **Single-point energy at B3LYP-D3/LANL2DZ /6-311+G(d,p) (in a.u.) (2)** | **Gibbs free energies (G) (in a.u.) (3)=(1)+(2)** | **Imaginary frequencies** |
|---|---|---|---|---|
| (NHC)AuCl | 0.416483 | −1934.392076 | −1933.975593 | |
| (NHC)AuNTf$_2$ | 0.450605 | −3301.123395 | −3300.67279 | |
| **IM1** | 0.494477 | −1716.910338 | −1716.415861 | |

| | | | | |
|---|---|---|---|---|
| **TS1** | 0.498836 | –1716.885832 | –1716.386996 | –96.96 |
| **IM2** | 0.502033 | –1716.896504 | –1716.394471 | |
| **IM3** | 0.709931 | –2207.863937 | –2207.154006 | |
| **TS2** | 0.711985 | –2207.865525 | –2207.15354 | –187.86 |
| **TS2'** | 0.709237 | –2207.856358 | –2207.147121 | –46.92 |
| **IM4** | 0.715 | –2207.896853 | –2207.181853 | |
| **TS3** | 0.739154 | –4035.029278 | –4034.290124 | –842.08 |
| **TS4** | 0.712209 | –2207.879736 | –2207.167527 | –389.11 |
| **IM5** | 0.707862 | –2207.476942 | –2206.76908 | |
| **IM6** | 0.713162 | –2207.883127 | –2207.169965 | |
| **TS5** | 0.701501 | –2207.44653 | –2206.745029 | –228.01 |
| LAuCl | 0.560349 | –2082.788824 | –2082.228475 | |
| $LAuNTf_2$ | 0.596023 | –3449.517661 | –3448.921638 | |
| **IM1˜** | 0.639296 | –1865.310666 | –1864.67137 | |
| **TS1˜** | 0.640816 | –1865.295658 | –1864.654842 | –171.89 |
| **IM2˜** | 0.648909 | –1865.328047 | –1864.679138 | |

| | | | | |
|---|---|---|---|---|
| **IM3˜** | 0.855903 | −2356.282252 | −2355.426349 | |
| **TS2˜** | 0.856872 | −2356.282359 | −2355.425487 | −174.25 |
| **TS2'˜** | 0.857859 | −2356.274747 | −2355.416888 | −127.93 |
| **IM4˜** | 0.859801 | −2356.324582 | −2355.464781 | |
| **TS3˜** | 0.888486 | −4183.436871 | −4182.548385 | −395.89 |
| **TS4˜** | 0.854166 | −2356.290175 | −2355.436009 | −817.86 |
| **IM5˜** | 0.894293 | −4183.459093 | −4182.5648 | |
| **IM6˜** | 0.857187 | −2356.294074 | −2355.436887 | |
| **TS5˜** | 0.840293 | −2355.832808 | −2354.992515 | −274.19 |
| **IM7˜** | 0.603142 | −3449.923773 | −3449.320631 | |
| **1a** | 0.055784 | −242.944479 | −242.888695 | |
| **2a** | 0.178073 | −502.579901 | −502.401828 | |
| AgCl | −0.023664 | −606.331485 | −606.355149 | |
| $AgNTf_2$ | 0.002317 | −1973.046611 | −1973.044294 | |
| $NTf_2^-$ | 0.006794 | −1827.131659 | −1827.124865 | |
| $K_3PO_4$ | −0.021975 | −2442.501183 | −2442.523158 | |

## 4. Cartesian Coordinates of All Structures Involved in This Work

Cartesian coordinates are available in the Source Data associated with this paper.

## References


1. Lücking, U., Chen, J., Rudkevich, D. M. & Rebek, J. A self-folding metallocavitand. *J. Am. Chem. Soc.* **123**, 9929–9934 (2001).
2. Danjo, T., Fujiwara, K., Nishikawa, T., Nakajima, T., Otsubo, N. & Seike, T. Heterocyclic compounds and methods for treating cancer. *Eur. Patent Application* EP 3401309 A1 (2018).
3. Gao, P., Xu, J., Zhou, T., Liu, Y., Bisz, E., Dziuk, B., Lalancette, R., Szostak, R., Zhang, D. & Szostak, M. L-shaped heterobidentate imidazo[1,5-a]pyridin-3-ylidene (N,C)-ligands for oxidant-free Au(I)/Au(III) catalysis. *Angew. Chem. Int. Ed.* **62**, e202218427 (2023).
4. Szostak, M. & Gao, P. Heterobidentate imidazo[1,5-a]pyridine and imidazo[1,5-a]quinoline N-heterocyclic carbene ligands, catalyst complexes thereof, and methods using same. *U.S. Patent Application* US 2024/0383894 A1 (2024).
5. Zhang, S., Wang, C., Ye, X. & Shi, X. Intermolecular Alkene Difunctionalization via Gold-Catalyzed Oxyarylation. *Angew. Chemie Int. Ed.* **59**, 20470–20474 (2020).
6. Scott, S. C., Cadge, J. A., Boden, G. K., Bower, J. F. & Russell, C. A. A hemilabile NHC–gold complex and its application to the redox-neutral 1,2-oxyarylation of feedstock alkenes. *Angew. Chem. Int. Ed.* **62**, e202301526 (2023).
7. Frisch, M. J.; T, G. W.; Schlegel, H. B.; Scuseria, G. E.; Robb, M. A.; Cheeseman, J. R.; Scalmani, G.; Barone, V.; Mennucci, B.; Petersson, G. A.; Nakatsuji, H.; Caricato, M.; Li, X.; Hratchian, H. P.; Izmaylov, A. F.; Bloino, J.; Zheng, G.; Sonnenberg, J. L.; Hada, M.; Ehara, M.; Toyota, K.; Fukuda, R.; Hasegawa, J.; Ishida, M.; Nakajima, T.; Honda, Y.; Kitao, O.; Nakai, H.; Vreven, T.; Montgomery, J. A., Jr.; Peralta, J. E.; Ogliaro, F.; Bearpark, M.; Heyd, J. J.; Brothers, E.; Kudin, K. N.; Staroverov, V. N.; Kobayashi, R.; Normand, J.; Raghavachari, K.; Rendell, A.; Burant, J. C.; Iyengar, S. S.; Tomasi, J.; Cossi, M.; Rega, N.; Millam, N. J.; Klene, M.; Knox, J. E.; Cross, J. B.; Bakken, V.; Adamo, C.; Jaramillo, J.; Gomperts, R.; Stratmann, R. E.; Yazyev, O.; Austin, A. J.; Cammi, R.; Pomelli, C.; Ochterski, J. W.; Martin, R. L.; Morokuma, K.; Zakrzewski, V. G.; Voth, G. A.; Salvador, P.; Dannenberg, J. J.; Dapprich, S.; Daniels, A. D.; Farkas, O.; Foresman, J. B.; Ortiz, J. V.; Cioslowski, J.; Fox, D. J., *Gaussian 09, revision b.01*; Gaussian, Inc.: Wallingford, CT, 2010.
8. Grimme, S. Semiempirical GGA-type density functional constructed with a long-range

dispersion correction. *J. Comput. Chem*. **2006**, *27*, 1787−1799.

9. Hay, P. J.; Wadt, W. R. Ab initio effective core potentials for molecular calculations. Potentials for the transition metal atoms Sc to Hg. *J. Chem. Phys*. **1985**, *82*, 270−283.
10. Wadt, W. R.; Hay, P. J. Ab initio effective core potentials for molecular calculations. Potentials for main group elements Na to Bi. *J. Chem. Phys*. **1985**, *82*, 284−298.
11. Hay, P. J.; Wadt, W. R. Ab initio effective core potentials for molecular calculations. Potentials for K to Au including the outermost core orbitals. *J. Chem. Phys*. **1985**, *82*, 299−310.
12. Ehlers, A.; Böhme, M.; Dapprich, S.; Gobbi, A.; Höllwarth, A.; Jonas, V.; Köhler, K.; Stegmann, R.; Veldkamp, A.; Frenking, G. A set of f-polarization functions for pseudo-potential basis sets of the transition metals Sc-Cu. Y-Ag and La-Au. *Chem. Phys. Lett*. **1993**, *208*, 111−114.
13. Huzinaga, S. Basis sets for molecular calculations. *Computer Physics Reports* **1985**, *2*, 281−339.
14. Marenich, A. V.; Cramer, C. J.; Truhlar, D. G. Universal Solvation Model Based on Solute Electron Density and on a Continuum Model of the Solvent Defined by the Bulk Dielectric Constant and Atomic Surface Tensions. *J. Phys. Chem. B* **2009**, *113*, 6378−6396.
15. Fukui, K. The path of chemical reactions-the IRC approach. *Acc. Chem. Res*. **1981**, *14*, 363−368.
16. Martin, R. L.; Hay, P. J.; Pratt, L. R. Hydrolysis of ferric ion in water and conformational equilibrium. *J. Phys. Chem. A* **1998**, *102*, 3565−3573.
17. Reed, A. E.; Weinstock, R. B.; Weinhold, F. Natural Population Analysis. *J. Chem. Phys*. **1985**, *83*, 735−746.
18. Reed, A. E.; Curtiss, L. A.; Weinhold, F. Intermolecular Interactions from a Natural Bond Orbital, Donor-Acceptor Viewpoint. *Chem. Rev*. **1988**, *88*, 899−926.
19. Glendening, E. D.; Reed, A. E.; Carpenter, J. E.; Weinhold, F. NBO, version 3.1; Gaussian Inc., 2003
20. Lu, T.; Chen, F. W. Multiwfn: A multifunctional wavefunction analyzer. *J. Comput. Chem*. **2012**, *33*, 580−592
21. Lu, T.; Chen, Q. Independent gradient model based on Hirshfeld partition: A new method

for visual study of interactions in chemical systems. *J. Comput. Chem*. **2022**, *43*, 539−555.

22. Humphrey, W.; Dalke, A.; Schulten, K. VMD: Visual Molecular Dynamics. *J. Mol. Graph*. **1996**, *14*, 33−38.
23. Legault, C. Y. CYLview, 1.0b, Université de Sherbrooke, Canada, **2009**, http://www.cylview.org.
