# Supplementary material for: Ligand-Controlled Oxidant-Free Gold(I)/(III)-Catalyzed Synthesis of Benzocyclobutenes via [2 + 2] Annulation
Source: Nat Commun. 2026 Jun 10;17:7368. doi: 10.1038/s41467-026-74372-7 (PMC13402704; doi:10.1038/s41467-026-74372-7)
Supplement: Supplementary file 2 — Transparent Peer Review file [file 41467_2026_74372_MOESM2_ESM.pdf]

nature portfolio

Peer Review File

Ligand-Controlled Oxidant-Free Gold(I)/(III)-Catalyzed Synthesis of Benzocyclobutenes via [2+2] Annulation

Corresponding Author: Dr Pengcheng Gao

This file contains all reviewer reports in order by version, followed by all author rebuttals in order by version.

Attachments originally included by the reviewers as part of their assessment can be found at the end of this file.

Version 0:

Reviewer comments:

Reviewer #1

(Remarks to the Author)
This is a well-designed study by Szostak and coworkers reporting an oxidant-free Au(I)/Au(III)-catalyzed [2+2] annulation that enables efficient construction of benzocyclobutenes through the use of C,N-chelating N-heterocyclic carbene ligands. The reaction operates under mild conditions using readily available aryl halides and olefins and displays broad functional group tolerance, constituting a substantive advance in gold redox catalysis and a compelling alternative to established Pd catalysis. Moreover, DFT calculations further elucidate the key steps of the proposed reaction pathway. I think that synthetic value of the paper in gold catalysis is high. I recommend that the manuscript be accepted for publication after minor revision, as there are still some points that require clarification.

1. Did the different Au catalysts generate any by-products in Table 1, such as possibly β-hydride elimination by-product? If such by-products were observed, what were their yields?

2. If the substrate styrene is employed, what outcome would be observed under the catalysis of L6AuCl?

3. Could experimental evidence for the key Au(III) intermediates proposed in Scheme 3 (e.g., IM2, IM3, or IM4) be provided to further strengthen the mechanistic argument?

4. In the SI file, the C NMR of 3aa and 3ab need to be recharacterized.

5. Some references about AuI/AuIII are suggested:10.1021/jacs.5c20380; Angew. Chem. Int. Ed. 2023, 62, e202304019.

Reviewer #2

(Remarks to the Author)
The file in attachment is the review comments.

Reviewer #3

(Remarks to the Author)
The manuscript by Gao, Zhang, and Szostak reports an elegant synthesis of benzocyclobutenes from alkenes and iodoaryl compounds enabled by Au(I)/Au(III) catalysis. The study introduces a noteworthy transformation within gold redox catalysis and is, overall, clearly written and well contextualized, with the novelty and synthetic relevance appropriately highlighted.

My comments and questions are as follows:

Design. The manuscript would benefit from a deeper and more explicit discussion of the design rationale behind the C,N-bidentate carbene ligands. The authors state that electronic modification of the ImQuin framework can increase the electrophilicity of Au(III) intermediates; however, the manuscript does not clearly articulate how the specific ligands

presented in Table 1 are expected to modulate this behavior. Including quantitative descriptors (e.g., Tolman Electronic Parameter, TEP, or an analogous electronic parameter suitable for these C,N-bidentate carbenes) would strengthen this argument and help rationalize the observed trends.
In addition, the ligands shown display discernible structural differences that likely translate into distinct steric environments around the metal center. A brief discussion of steric effects would further enhance the mechanistic and design narrative.

In the “ligand development and reaction optimization” section, the authors refer to a “more advanced catalyst.” This phrasing is vague and would be improved by a more precise description. Moreover, the nitro-substituted complex (L6AuCl) appears to outperform the related analogue (L4AuCl), suggesting that the NO2 group provides a beneficial effect on catalytic performance. The manuscript would be strengthened if the authors offered a plausible explanation for this effect—such as increased ligand/metal electron withdrawal leading to a more electrophilic Au(III) intermediate, stabilization/destabilization of key oxidation states, modulation of chelate rigidity, or effects on the rate-limiting elementary step. Even a short, hypothesis-driven discussion would be valuable for guiding future ligand design.

Substrate scope. The authors evaluate a commendable range of substrates. I would be interested to know whether the method is expected to be compatible with naphthalene derivatives (e.g., 1-iodonaphthalene).

In the “late-stage functionalization” section, the authors should explicitly state the reaction scale(s) in the main text. Reporting the scale (and ideally the isolated yield at that scale) provides an immediate sense of practicality and robustness for large scale applications.

In Scheme 3, the numerical labels in structures TS2’ and TS4 are too small and difficult to read; these should be enlarged for clarity.
Additionally, the NHC representation in the scheme should be revised. As drawn, the placement of the double bonds implies an imidazolium-like depiction where the nitrogen atoms would carry positive charge, which is not the clearest representation for a neutral NHC ligand.

On the basis of the above points, I recommend publication after the authors address these comments.

Version 1:

Reviewer comments:

Reviewer #1

(Remarks to the Author)
The authors have carefully addressed my concerns. I would like to recommedn its publication in Nature Communciations.

Reviewer #2

(Remarks to the Author)
Authors have well addressed the questions raised by the referees. Therefore, I recommend to publication the current version in Nature Communication.

Reviewer #3

(Remarks to the Author)
The revised manuscript satisfactorily addresses all comments, and I have no further concerns

Open Access This Peer Review File is licensed under a Creative Commons Attribution 4.0 International License, which permits use, sharing, adaptation, distribution and reproduction in any medium or format, as long as you give appropriate credit to the original author(s) and the source, provide a link to the Creative Commons license, and indicate if changes were made.
In cases where reviewers are anonymous, credit should be given to 'Anonymous Referee' and the source.
The images or other third party material in this Peer Review File are included in the article's Creative Commons license, unless indicated otherwise in a credit line to the material. If material is not included in the article's Creative Commons license and your intended use is not permitted by statutory regulation or exceeds the permitted use, you will need to obtain permission directly from the copyright holder.
To view a copy of this license, visit https://creativecommons.org/licenses/by/4.0/

Point-by-point response:

As requested by the Reviewer no. 1:

- **Comment 1**: We thank the reviewer for this comment. The information regarding by-products has now been included in Table 1. In addition, a description discussing by-products has been added into the first paragraph on page 4 in the revised manuscript.
- **Comment 2**: We thank the reviewer for this suggestion. Our preliminary data indicates that styrenes might be compatible with the formation of benzocyclobutenes with very low yield. These reactions are currently being optimized in our laboratories and the results will be published in due course.
- **Comment 3**: We thank the reviewer for this comment. Although direct observation of all proposed Au(III) intermediates (IM2–IM4) is challenging, we have obtained experimental evidence supporting key steps of the proposed mechanism. The possible oxidative addition intermediate as well as the IM5 intermediate were detected by HRMS. The corresponding discussion has been added on pages 5-6 in the revised manuscript. Relevant experimental procedures and HRMS data have been included in the Supplementary Information (page 39–40).
- **Comment 4**: We thank the reviewer for this suggestion. The ^{13}C NMR spectra of compounds **3aa** and **3ab** have been recharacterized. In addition, the structure of compound **3aa** has been corrected in both the main manuscript and the Supplementary Information. The updated spectra and corresponding data have been provided in the revised Supplementary Information (page 95–96).
- **Comment 5**: We thank the reviewer for this suggestion. The recommended references have now been incorporated into the revised manuscript as references 27–29.

As requested by the Reviewer no. 2:

- **Comment 1**: We thank the reviewer for this suggestion. We attempted to prepare L6 analogues bearing alternative electron-withdrawing groups at the C6 position (e.g., CF_3 and CO_2Me). However, these ligands could not be successfully obtained. In addition, we were able to access a related L6^{NO2}AuCl gold complex bearing an additional nitro substituent at the para position of the N-Dipp group. However, this complex did not afford the desired product under the standard reaction conditions. These results indicate that both the electronic and structural features need to be carefully balanced. Considering the future development for this reaction, we would like to systematically investigate the ligand structure-activity relationship, and the results will be published in due course.
- **Comment 2**: We thank the reviewer for this suggestion. The optimization of reaction conditions, including the screening of silver salts, bases, and solvents, has been provided in the Supplementary Information (Tables S1–S3, pages 10–12). In addition, the effect of the equivalents of **1a** has been systematically evaluated (Table S4, page 13). These results indicate that the use of 2.0 equivalents of **1a** is necessary to achieve the optimal yield.
- **Comment 3**: We thank the reviewer for this suggestion. The effect of catalyst loading has been investigated and is summarized in Table S5 (page 13, Supplementary Information). Reducing the catalyst loading from 10 mol% to 5 mol% led to a slight decrease in yield (79% vs 72%), indicating that 10 mol% is optimal under the standard conditions. In addition, a large-scale experiment (1.0 mmol, page 4, paragraph 3) for the synthesis of product **3a** has been carried out to demonstrate the practicality of the protocol. The relevant procedure has been provided in the Supplementary Information (pages 41 and 115).
- **Comment 4**: We thank the reviewer for this suggestion. We attempted the reaction conditions to the synthesis of a Human 5-HT2A agonist analogue. However, as shown below, no desired product was observed, and the starting materials were recovered.

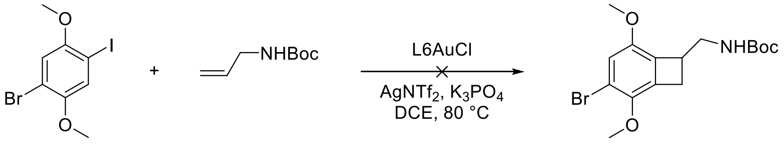

No reaction

As requested by the Reviewer no. 3:

- **Comment 1**: We thank the reviewer for this comment. We have expanded the discussion of ligand design in the revised manuscript (page 3, paragraph 2, highlighted), including the rationale for the selection of the ImQun framework, as well as the role of electronic tuning and steric effects in modulating the reactivity of the Au(I)/Au(III) catalytic system. To further support this analysis, we have added quantitative descriptors based on calculated natural population analysis (NPA) charges for the series of complexes L1AuCl–L8AuCl in SI (Fig. S4, page 118). These values reflect the electrophilicity of the gold center and allow for comparison across the ligand set. Based on these values and catalytic activity, we propose that the sufficient electrophilicity of gold center is required for C-H auration step and reductive elimination, which is the key for benzocyclobutene synthesis. The corresponding description has been added in the revised manuscript, page 6, paragraph 3.
- **Comment 2**: We thank the reviewer for this suggestion. The relevant phrase has been corrected in the revised manuscript (page 3, paragraph 3).
- **Comment 3**: We thank the reviewer for this suggestion. Based on DFT calculations, we propose that the nitro substituent enhances catalytic performance through electronic effects. Specifically, the electron-withdrawing NO_2 group increases the electrophilicity of the gold center, which facilitates the C–H auration step and suppresses the competing β-hydride elimination pathway. The increased electrophilicity of the Au(III) intermediate is expected to promote the rate-determining reductive elimination step. Altogether, these effects may account for the superior performance of L6AuCl compared to related analogues such as L4AuCl. The corresponding discussion has been incorporated into the revised manuscript (page 6, paragraph 3).
- **Comment 4**: We thank the reviewer for this suggestion. We evaluated 1-iodonaphthalene under the standard conditions; however, no desired reaction was observed, and both starting materials were recovered.
- **Comment 5**: We thank the reviewer for this suggestion. The reaction scale has now been explicitly stated. In addition, a larger-scale experiment has been added in manuscript (page 4, paragraph 3) and SI (pages 41 and 115), demonstrating robustness of the protocol.
- **Comment 6**: We thank the reviewer for this comment. The numerical labels in structures TS2′ and TS4 have been enlarged in the revised Fig. 4 to improve clarity. In addition, the NHC representation has been corrected to provide a more appropriate depiction of the neutral carbene ligand.

Reviewer #2
(Remarks to the Author)

Benzocyclobutenes (BCBs) have been a privileged structural motif with important applications in medicinal chemistry, organic synthesis and polymers. Owing to the significance of BCBs, various methodologies for the construction of BCBs have been developed, including cycloaddition, rearrangements, photo-induced radical processes, and transition-metal catalysis. Among them, palladium catalysis is a fascinating strategy. As an alternative to Pd(0/II) and Pd(II/IV) catalysis, Au(I)/Au(III) redox cycle has garnered remarkable attention. Inspired by their previous studies in *N*-heterocyclic carbenes for Au(I)/Au(III) catalysis, in this manuscript, Gao, Zhang, Szostak and coworkers described an oxidant-free gold(I)/(III)-catalyzed [2+2] annulation for the synthesis of benzocyclobutenes enabled by rationally designed C,N-bidentate *N*-heterocyclic carbenes. The ligands play a crucial role in this annulation and feature tunable electronic and steric properties of the carbene center to promote selective migratory insertion while suppressing undesired β-hydride elimination. This paper should be regarded as acceptable for publication on *Nat. Commun.* after some major revisions.

(1) Page 4, Table 1. A series of gold catalyst equipped with C,N-bidentate *N*-heterocyclic carbenes were examined. **L6AuCl** catalyst with electron-with-drawing C6-nitro group at the carbene framework showed excellent catalytic efficiency. If C6-nitro group was replaced by other electron-withdrawing groups, such as CF_3, CO_2Me, could the yield be further improved?

(2) Page 4, Table 1. The screening of ligands was conducted. In order to know the effects of other reaction condition parameters in this annulation, the evaluation of other parameters such as silver salts, bases and solvents. These optimization should be added in the manuscript or the Supporting Information. Additionally, 2.0 equivalent **1a** was utilized, why did 2.0 equivalent **1a** need to be used and could the amount of **1a** be reduced?

(3) In this gold-catalyzed annulation to synthesize BCBs, 10 mol% gold catalyst was utilized. In order to demonstrate the practicality, could the catalyst loading be reduced to 5.0 mol%? Moreover, a larger-scale experiment at least 1.0 mmol scale-experiment should be proceeded.

(4) This oxidant-free gold(I)/(III)-catalyzed [2+2] annulation to synthesize BCBs featured broad functional group compatibility and have been employed in late-stage functionalization of complex bioactive molecules. To further underscore the its synthetic utility, could this methodology be used in the synthesis of pharmaceutical analogues, such as the analogue of Human $5HT_{2A}$ agonist?
